# Causal Associations of Environmental Pollution and Cardiovascular Disease: A Two-Sample Mendelian Randomization Study

**DOI:** 10.5334/gh.1331

**Published:** 2024-06-18

**Authors:** Hui Gao, Jiahai Li, Qiaoli Ma, Qinghui Zhang, Man Li, Xiaoliang Hu

**Affiliations:** 1 Department of Cardiovascular Medicine, The First People’s Hospital of Shangqiu, Shangqiu 476000, China; 2 Graduate School, Dalian Medical University, Dalian, 116044, China; 3 Department of Cardiovascular Medicine, The First People’s Hospital of Qinzhou, Qinzhou 535000, China; 4 Department of Cardiovascular Medicine, Central Hospital of Zibo, Zibo 255000, China; 5 Department of Hypertension, Henan Provincial People’s Hospital, Zhengzhou 450000, China

**Keywords:** Mendelian randomization, Environmental pollution, Cardiovascular disease

## Abstract

**Background:**

There is growing evidence that concentrations of environmental pollutants are previously associated with cardiovascular disease; however, it is unclear whether this association reflects a causal relationship.

**Methods:**

We utilized a two-sample Mendelian randomization (MR) approach to investigate how environmental pollution affects the likelihood of developing cardiovascular disease. We primarily employed the inverse variance weighted (IVW) method. Additionally, to ensure the robustness of our findings, we conducted several sensitivity analyses using alternative methodologies. These included maximum likelihood, MR-Egger regression, weighted median method and weighted model methods.

**Results:**

Inverse variance weighted estimates suggested that an SD increase in PM2.5 exposure increased the risk of heart failure (OR = 1.40, 95% CI 1.02–1.93, p = 0.0386). We found that an SD increase in PM10 exposure increased the risk of hypertension (OR = 1.45, 95% CI 1.02–2.05, p = 0.03598) and atrial fibrillation (OR = 1.41, 95% CI 1.03–1.94, p = 0.03461). Exposure to chemical or other fumes in a workplace was found to increase the risk of hypertension (OR = 3.08, 95% CI 1.40–6.78, p = 0.005218), coronary artery disease (OR = 1.81, 95% CI 1.00–3.26, p = 0.04861), coronary heart disease (OR = 3.15, 95% CI 1.21–8.16, p = 0.0183) and myocardial infarction (OR = 3.03, 95% CI 1.13–8.17, p = 0.02802).

**Conclusion:**

This study reveals the causal relationship between air pollutants and cardiovascular diseases, providing new insights into the protection of cardiovascular diseases.

## Introduction

Cardiovascular disease (CVD) is the leading cause of morbidity and mortality worldwide [1] and is the most common cause of mortality in rich countries [2], Internationally, China and India have the highest burdens of CVD [3]. There is an increasing prevalence of CVD in China, where it represents the leading cause of mortality In 2015 [4,5], the World Health Organization (WHO) estimated that CVD accounted for more than 17.7 million deaths, representing a total 31% of global deaths [6]. Despite extensive efforts in recent years, the causes of cardiovascular disease remain largely unknown [7].

Although previous studies have shown that several modifiable risk factors are associated with an increased risk of cardiovascular disease [8,9,10,11,12]. In addition to well-established risk factors (such as dyslipidaemia and hypertension) [13,14], exposure to air pollution has attracted a lot of attention in the media for its relationship with coronary ischaemia [15]. In addition, previous epidemiological studies have confirmed noise and air pollution as risk factors for hypertension [33,34].

In line with these observations, the World Health Organization (WHO) estimated that each year approximately 800,000 people die prematurely, which could be attributed to air pollution worldwide [16]. Air pollution is thought to predominantly exacerbate cardiopulmonary disease that causes death [17]. However, the use of portable air cleaners or respirators could significantly reduce fine particulate matter exposures on blood pressure and heart rate variability, which could reduces the risk of hypertension and other cardiovascular diseases [35,36].

In case of long-term exposure, these effects are even more pronounced with a one-year life reduction for 18,000 individuals [18]. Against this background, various epidemiological studies have shown that increased levels of air pollution could augment cardiovascular morbidity and mortality due to is chaemic events, more frequent hospitalisations, worsening of heart failure and (ventricular) arrhythmias [19], and that these effects occur both due to daily changes in air pollutant levels as well as due to lifetime exposure [20]. If air pollution has indeed become a relevant factor in the occurrence of cardiovascular disease, this should have worldwide consequences [21]. Recently, the relationship between environmental pollution and cardiovascular diseases has become the focus of clinicians and epidemiologists [37]. With this study, we aim to establish causal attribution regarding the relationship between air pollution and cardiovascular disease.

In recent years, Mendelian randomization (MR) has provided an effective way to make causal inferences in observational studies [22], especially when it is difficult to conduct clinical trials due to medical ethics, subject selection constraints and poor extrapolation of results [23]. Previous MR studies have validated the relationship between LDL-C and atherosclerotic cardiovascular disease (ASCVD) [24]. Therefore, in this study, we used MR to examine the potential causal inference between environmental pollution and cardiovascular disease.

## Methods

### Study design

In our research, we utilized a two-sample Mendelian randomization (MR) approach to investigate how environmental pollution affects the likelihood of developing cardiovascular disease. We obtained publicly available summary datasets from two genome-wide association studies (GWAS) as our primary data sources. Our study focused on several types of environmental pollutants, including Particulate Matter (PM) 2.5 air pollution, PM10 air pollution, Nitrogen dioxide (NO2) air pollution and Noise pollution. The outcome of interest was the occurrence of cardiovascular disease. To establish instrumental variables (IVs), we carefully selected specific single-nucleotide polymorphisms (SNPs) that exhibited robust associations with the various forms of environmental pollution. The MR framework operates based on three crucial assumptions: (i) The genetic instrumental variables are correlated with the exposure (environmental pollution); (ii) The genetic instrumental variables are independent of potential confounding factors; (iii) The genetic instrumental variables solely influence the outcome (cardiovascular disease) through the exposure (environmental pollution). By adhering to these assumptions, our aim was to investigate the causal relationship between environmental pollution and the risk of cardiovascular disease.

**Figure 1:**
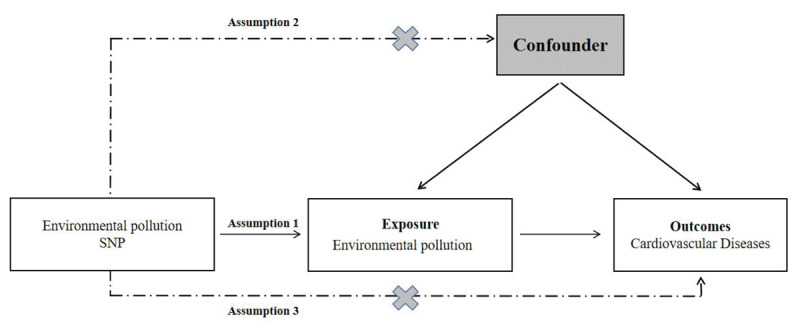
Study design flowchart of the Mendelian randomization study.

Two-sample MR study was conducted to evaluate the causal relationship between environmental pollutants and CVD risk. Single-nucleotide polymorphisms were used as IVs [25], An overview of the research design is presented in Figure 1. The entire process satisfied the three main hypotheses of classical MR analysis: 1. IVs directly affected exposure; 2. IVs were not associated with confounders and 3. IVs influenced the risk of outcomes directly through exposure, not through other pathways. All the original studies obtained ethical approval and informed consent. This study was conducted based on the latest (STROBE-MR) guidelines [26].

### Summary data resources

#### Environmental pollution

For our analysis on PM2.5, PM10, NO2 and Noise pollution exposure, we sourced the summary genetic data from the UK Biobank GWAS. This dataset involved 456,380 participants of European descent and was publicly available through the MRC-IEU Open GWAS data. The specific GWAS-IDs used were ukb-b-10817 for PM2.5 (unit: μg/m^3^), ukb-b-18469 for PM10 (unit: μg/m^3^), ukb-b-9942 for NO2 (unit: μg/m^3^), and ukb-b-19490 for Noise pollution (decibel, dB). Our study was conducted as part of the ESCAPE project (European Study of Cohorts for Air Pollution Effects) and made use of the GWAS pipeline using Phesant-derived variables from the UK Biobank. The primary objective of this project was to examine the impacts of environmental pollution on various health outcomes. The detailed information of each dataset for environmental pollution as showed in the Supplementary Table 1.

#### Cardiovascular disease

Our analysis incorporated a total of 13 cardiovascular diseases with varying case numbers, ranging from 556 (hypertrophic cardiomyopathy) to 122,733 (coronary artery disease). To obtain summary statistics for the associations between the instrumental variables (IVs) and asthma, we extracted data from different consortiums. Specifically, we sourced information about coronary artery disease, and myocardial infarction from the Coronary ARtery DIsease Genome-wide Replication and Meta-analysis plus The Coronary Artery Disease Genetics (CARDIoGRAMplusC4D) consortium for coronary artery disease and myocardial infarction. We sourced information about heart failure from the Heart Failure Molecular Epidemiology for Therapeutic Targets (HERMES) consortium, and information about atrial fibrillation from the Atrial Fibrillation Gen consortium. Additionally, we obtained GWAS summary statistics for the remaining cardiovascular diseases from the FinnGen-R5 consortium. These diseases included hypertension, ischemic heart disease, non-ischemic cardiomyopathy, cardiomyopathy, hypertrophic cardiomyopathy, valvular heart disease and pulmonary heart disease.

### Selection of instrumental variables

To identify genetic predictors associated with environmental pollution characteristics, we implemented rigorous quality control procedures. Initially, we employed a stringent genome-wide significance threshold of P < 5 × 10^–8^ to identify highly associated single-nucleotide polymorphisms (SNPs) linked to both environmental pollution and cardiovascular disease. However, due to a limited number of eligible instrumental variables (IVs), we applied a relatively comprehensive threshold of P < 5 × 10^–6^ to capture a broader set of SNPs for more comprehensive results. To ensure adherence to the assumptions of Mendelian randomization (MR), we conducted a linkage disequilibrium (LD) analysis using data from the European-based 1,000 Genomes Project. Single-nucleotide polymorphisms that did not meet the criteria (R^2^ < 0.001, clumping distance = 10,000 kb) were excluded from further analysis. We also excluded palindromic SNPs due to uncertainties regarding their alignment in the same direction for both exposure and outcome in the cardiovascular disease genome-wide association studies. Additionally, SNPs with a minor allele frequency (MAF) below 0.01 were excluded from the analysis. In cases where SNPs associated with the exposure variable were missing in the outcome GWAS dataset, we selected proxy SNPs with high linkage disequilibrium (r^2^ > 0.80) to ensure comprehensive coverage. To assess the instrument strength, we calculated the F statistic using the formula F = R^2^(n–2)/(1–R2), where R^2^ represents the proportion of variance explained by the instrumental variables, and represents the sample size. A value below 10 for the F statistic indicates a higher likelihood of weak instrument bias, urging caution in result interpretation.

### Statistical analysis

To analyze the Mendelian randomization (MR) data, we primarily employed the inverse variance weighted (IVW) method. Additionally, to ensure the robustness of our findings, we conducted several sensitivity analyses using alternative methodologies. These included maximum likelihood, MR-Egger regression, weighted median method, and weighted model methods. In cases where the IVW method yielded statistically significant results (p < 0.05), we considered the outcome positive, even if other methods did not reach significance, as long as the direction of the beta values remained consistent. To assess the impact on cardiovascular disease, we estimated odds ratios (OR) with their corresponding 95% confidence intervals (CIs), applying a significance threshold of p < 0.05. Heterogeneity was evaluated using Cochran’s Q test for the IVW and MR-Egger estimates. We utilized the MR-Egger regression technique to explore potential pleiotropic bias. To evaluate the stability of our findings, we conducted a systematic ‘leave-one-out’ analysis, sequentially excluding each single-nucleotide polymorphism (SNP) to assess its influence on the overall results. All statistical analyses were performed using the two-Sample MR package (version 0.5.5) within the R software environment (version 4.0.3). These stringent analytical approaches were implemented to ensure the reliability and validity of the study’s outcomes.

## Results

### Selection of genetic instruments

In order to examine the relationship between environmental pollution and the risk of cardiovascular disease, we performed a Mendelian randomization (MR) analysis involving nine environmental pollution traits in relation to cardiovascular disease. Strong genetic instruments (p-values < 5 × 10^–6^) were obtained for environmental pollution traits, ensuring their independence (r^2^ < 0.01) by excluding palindromic single nucleotide polymorphisms (SNPs). Specifically, we identified 52, 27, 73 and 12 SNPs as proxies for a standard deviation increase in PM2.5, PM10, NO2 and Noise pollution levels, respectively. The F-statistics for the instrumental variables were all significantly above 10, indicating the absence of weak instrument bias. The raw data for the instrumental variables is shown in Supplementary Table 2.

### Causal effect of environmental pollution on cardiovascular disease risk

The statistical results of MR are presented in Table 1. Using an IVW approach, we found that an SD (unit: one μg/m^3^) increase in PM2.5 exposure increased the risk of heart failure (OR = 1.40, 95% CI 1.02–1.93, p = 0.0386). We also found positive associations between PM2.5 exposure level and heart failure risk using the maximum likelihood, MR-Egger, weighted median and weighted mode methods. Moreover, we found that an SD (unit: μg/m^3^) increase in PM10 exposure increased the risk of hypertension (OR = 1.45, 95% CI 1.02–2.05, p = 0.03598) and atrial fibrillation (OR = 1.41, 95% CI 1.03–1.94, p = 0.03461), as shown in the results in Table 1. Further positive associations between PM10 exposure levels and hypertension, as well atrial fibrillation risk, were found using the maximum likelihood, MR-Egger, weighted median and weighted mode methods. However, our study did not find that nitrogen dioxide air pollution and noise pollution have causal effect on the increased risk of cardiovascular disease. More details as showed in Supplementary Table 3.

**Table 1:** Significant MR analysis results in the discovery samples.


OUTCOME	EXPOSURE	METHOD	NO.SNP	OR	95%CI		p

					LOWER	UPPER	

Heart failure	Particulate matter air pollution (pm2.5)	IVW	47	1.40	1.02	1.93	0.039

		Maximum likelihood	47	1.42	1.14	1.76	0.002

		MR Egger	47	1.20	0.50	2.88	0.689

		Weighted median	47	1.17	0.85	1.62	0.344

		Weighted mode	47	1.19	0.74	1.94	0.475

Hypertension	Particulate matter air pollution (pm10)	IVW	27	1.45	1.02	2.05	0.036

		Maximum likelihood	27	1.47	1.03	2.11	0.033

		MR Egger	27	1.26	0.61	2.58	0.542

		Weighted median	27	1.45	0.88	2.38	0.142

		Weighted mode	27	1.65	0.67	4.11	0.288

Atrial fibrillation	Particulate matter air pollution (pm10)	IVW	27	1.41	1.03	1.94	0.035

		Maximum likelihood	27	1.44	1.10	1.89	0.008

		MR Egger	27	1.53	0.75	3.13	0.252

		Weighted median	27	1.29	0.87	1.91	0.209

		Weighted mode	27	1.23	0.55	2.75	0.622


### Sensitivity analyses

Sensitivity analyses were shown in Table 2. The estimates of causal effects obtained through the maximum likelihood, MR-Egger regression, weighted median method and weighted model methods were consistent in terms of both magnitude and direction. This consistency strengthens the reliability of the findings. Our analysis provided no substantial evidence of horizontal pleiotropy, suggesting that the instrumental variables used in the study were not influenced by factors other than the exposure being investigated. This was supported by p-values greater than 0.05 when employing the MR-Egger regression intercept approach. Furthermore, the Cochrane Q statistics, used to assess heterogeneity, did not indicate statistically significant differences among the estimates (p > 0.05). This suggests that the underlying genetic variants used as instruments for environmental pollution were not significantly influencing the outcome in different ways and that the causal relationship between air contaminants and CVD risk was not driven by a single SNP (Supplementary Figure S1). Moreover, the leave-one-out analysis, which involved excluding one variant at a time, demonstrated that the effect estimates remained stable and were not unduly influenced by any single variant. This further reinforces the robustness of the results obtained. Overall, these findings indicate a consistent and reliable relationship between environmental pollution and cardiovascular disease with no significant confounding factors or outliers affecting the observed causal effects (see funnel plots shown in Supplementary Figures S2).

**Table 2:** Horizontal pleiotropy test and Heterogeneity test for significant MR analysis results.


OUTCOMES	HORIZONTAL PLEIOTROPY			HETEROGENEITY						
	
	MR-EGGER			MR-EGGER			IVW		
		
	Intercept	SE	P-value	Q	Q_df	Q_P	Q	Q_df	Q_P

Heart failure	0.0024	0.0063	0.706	108	45	4.246E-07	108.4	46	6.044E-07

Hypertension(PM10)	0.003	0.0067	0.66	24.68	25	0.4807	24.87	26	0.5261

Atrial fibrillation	–0.0016	0.0064	0.802	39.41	25	0.0335	39.51	26	0.04353

Hypertension	0.0021	0.0068	0.765	15.91	15	0.3883	16.01	16	0.4526

Coronary artery disease	–0.0037	0.0052	0.482	13.89	15	0.5338	14.41	16	0.5681

Coronary heart disease	–0.0096	0.0085	0.276	17.38	15	0.2969	18.86	16	0.2762

Myocardial infarction	–0.0021	0.0095	0.829	14.99	14	0.3788	15.04	15	0.4484


## Discussion

We used MR for the first time to systematically explore potential causal effects between environmental pollution susceptibility and cardiovascular disease risk. We found a causal relationship between heart failure and heart failure and exposure to PM2.5, with an increased risk of heart failure and heart failure in people exposed to high levels of PM2.5 compared to people exposed to low concentrations. We also found that PM10 exposure levels were positively associated with the risk of developing heart failure, hypertension and atrial fibrillation, i.e. PM10 exposure can increase the risk of developing heart failure, hypertension and atrial fibrillation. In addition, we found that exposure to chemicals or other fumes in the workplace increases the risk of high blood pressure, coronary artery disease, coronary heart disease and myocardial infarction. These results suggest a causal relationship between certain environmental pollutants and cardiovascular disease.

Our findings support previous observational research showing every 10 μg/m^3^ increase in PM2.5 (overall range 2.9–28.0 μg/m^3^) is associated with a 16% increase in ischaemic heart disease mortality [27]. There was also evidence from previous cohort studies indicated that long-term exposures to road traffic noise and ambient air pollution were associated with blood biochemistry [28]. This result provided a possible link between long-term exposures to road traffic noise/air pollution and the increased risk of cardio-metabolic disease. Importantly, in our main analysis, we failed to find a causal relationship between noise pollution and CVD outcomes. The short- and long-term exposure to PM contributes to the development and progression of acute and chronic CV diseases. Particulate matter can easily enter into the respiratory system and contributes to the development of cardiovascular events by inducing a systemic inflammatory condition or affecting the autonomic nervous system [29]. This is consistent with our findings, that exposure to PM2.5 and PM10 increases the risk of heart disease, high blood pressure and atrial fibrillation. Meanwhile, a study in Lanzhou suggests that increased PM10 concentrations may be responsible for increased mortality from cerebrovascular disease and ischemic heart disease. Therefore, reducing environmental particulate matter is of great significance for reducing the incidence of cardiovascular disease.

Our research has several advantages. First, MR analyses of genetic susceptibility to other factors and cardiovascular disease risk have recently been reported [30,31], but no MR studies have analyzed the potential causal relationship between environmental pollutants and cardiovascular disease risk. Secondly, through large-scale GWAS analysis, we conducted MR analyses for nine environmental pollution markers and 13 cardiovascular diseases. The MR design reinforced the causal inference by diminishing residual confounding and other biases. Genetic knowledge of environmental particulate matter exposure and cardiovascular related diseases has been further expanded. These large-scale geographic information systems provide more precise correlation. This magnetic resonance analysis utilizes the latest exposure and outcome GIS datasets to comprehensively investigate the potential relationship between ambient particulate matter and cardiovascular disease, avoiding traditional confounding factors and reverse causation. Third, repeated analysis using a variety of methods to obtain consistent results. Sensitivity analysis and IVS intensity evaluation were used to verify that the results were not biased [32]. We confined the population in the present study to individuals of European ancestry to minimize population structure bias, with the exception for the analysis for coronary artery disease, which might be challenged by bias from ethnicity, based on consortium data where European individuals comprised over 80% of participants. Nevertheless, this population confinement limited the generalizability of our findings to other populations.

## Conclusions

In summary, this two-sample MR study found that a causal relationship between multiple environmental pollutants and cardiovascular diseases. However, the special effect and mechanism of environmental pollutants on cardiovascular diseases still need to be further investigate in future studies.

## Data Accessibility Statement

The data were derived from the following resources available in the public domain: Telomere GWAS.

## Additional File

The additional file for this article can be found as follows:

10.5334/gh.1331.s1 Supplementary File. Supplementary Tables and Supplementary Figures.

## Abbreviations

MR: Mendelian randomization; CVD: Cardiovascular disease; IVW: inverse variance weighted; WHO: World Health Organization; GWAS: genome-wide association studies; SNPS: single-nucleotide polymorphisms.

## References

[1] Nitsa A, Toutouza M, Machairas N, Mariolis A, Philippou A, Koutsilieris M. Vitamin D in cardiovascular disease. In Vivo. 2018; 32(5): 977–981. DOI: 10.21873/invivo.11338 30150419 PMC6199603

[2] Ortega FB, Lavie CJ, Blair SN. Obesity and cardiovascular disease. Circ Res. 2016; 118(11): 1752–70. DOI: 10.1161/CIRCRESAHA.115.306883 27230640

[3] Alaa AM, Bolton T, Di Angelantonio E, Rudd JHF, van der Schaar M. Cardiovascular disease risk prediction using automated machine learning: A prospective study of 423,604 UK Biobank participants. PLoS One. 2019; 14(5): e0213653. DOI: 10.1371/journal.pone.0213653 31091238 PMC6519796

[4] Liu S, Li Y, Zeng X, Wang H, Yin P, Wang L, et al. Burden of cardiovascular diseases in China, 1990–2016: Findings from the 2016 Global Burden of Disease Study. JAMA Cardiol. 2019; 4(4): pp. 342–352. DOI: 10.1001/jamacardio.2019.0295 30865215 PMC6484795

[5] Kario K, Hoshide S, Chia YC, Buranakitjaroen P, Siddique S, Shin J, et al. Guidance on ambulatory blood pressure monitoring: A statement from the HOPE Asia Network. J Clin Hypertens (Greenwich). 2021; 23(3): 411–421. DOI: 10.1111/jch.14128 33319412 PMC8029567

[6] Roth GA, Johnson C, Abajobir A, Abd-Allah F, Abera SF, Abyu G, et al. Global, regional, and national burden of cardiovascular diseases for 10 causes, 1990 to 2015. J Am Coll Cardiol. 2017; 70(1): 1–25. DOI: 10.1016/j.jacc.2017.04.052 28527533 PMC5491406

[7] Chieng D, Canovas R, Segan L, Sugumar H, Voskoboinik A, Prabhu S, et al. The impact of coffee subtypes on incident cardiovascular disease, arrhythmias, and mortality: long-term outcomes from the UK Biobank. Eur J Prev Cardiol. 2022; 29(17): 2240–2249. DOI: 10.1093/eurjpc/zwac189 36162818

[8] Matsushita K, Ballew SH, Wang AY, Kalyesubula R, Schaeffner E, Agarwal R, et al. Epidemiology and risk of cardiovascular disease in populations with chronic kidney disease. Nat Rev Nephrol. 2022; 18(11): 696–707. DOI: 10.1038/s41581-022-00616-6 36104509

[9] An P, Wan S, Luo Y, Luo J, Zhang X, Zhou S, et al. Micronutrient supplementation to reduce cardiovascular risk. J Am Coll Cardiol. 2022; 80(24): 2269–2285. DOI: 10.1016/j.jacc.2022.09.048 36480969

[10] Huh JH, Han KD, Cho YK, Roh E, Kang JG, Lee SJ, et al. Remnant cholesterol and the risk of cardiovascular disease in type 2 diabetes: A nationwide longitudinal cohort study. Cardiovasc Diabetol. 2022; 21(1): 228. DOI: 10.1186/s12933-022-01667-6 36324177 PMC9632127

[11] Borghi C, Agnoletti D, Cicero AFG, Lurbe E, Virdis A. Uric acid and hypertension: A review of evidence and future perspectives for the management of cardiovascular risk. Hypertension. 2022; 79(9): 1927–1936. DOI: 10.1161/HYPERTENSIONAHA.122.17956 35658505

[12] Neves JS, Newman C, Bostrom JA, Buysschaert M, Newman JD, Medina JL, et al. Management of dyslipidemia and atherosclerotic cardiovascular risk in prediabetes. Diabetes Res Clin Pract. 2022; 190: 109980. DOI: 10.1016/j.diabres.2022.109980 35787415

[13] Boren J, Taskinen MR, Björnson E, Packard CJ. Metabolism of triglyceride-rich lipoproteins in health and dyslipidaemia. Nat Rev Cardiol. 2022; 19(9): 577–592. DOI: 10.1038/s41569-022-00676-y 35318466

[14] Savarese G, von Haehling S, Butler J, Cleland JGF, Ponikowski P, Anker SD. Iron deficiency and cardiovascular disease. Eur Heart J. 2023; 44(1): 14–27. DOI: 10.1093/eurheartj/ehac569 36282723 PMC9805408

[15] de Bont J, Jaganathan S, Dahlquist M, Persson Å, Stafoggia M, Ljungman P. Ambient air pollution and cardiovascular diseases: An umbrella review of systematic reviews and meta-analyses. J Intern Med. 2022; 291(6): 779–800. DOI: 10.1111/joim.13467 35138681 PMC9310863

[16] Nwanaji-Enwerem JC, Allen JG, Beamer PI. Another invisible enemy indoors: COVID-19, human health, the home, and United States indoor air policy. J Expo Sci Environ Epidemiol. 2020; 30(5): 773–775. DOI: 10.1038/s41370-020-0247-x 32641763 PMC7341994

[17] Ling SH, van Eeden SF. Particulate matter air pollution exposure: role in the development and exacerbation of chronic obstructive pulmonary disease. Int J Chron Obstruct Pulmon Dis. 2009; 4: 233–43. DOI: 10.2147/COPD.S5098 19554194 PMC2699820

[18] Lou X, Zhang P, Shi N, Ding Z, Xu Z, Liu B, et al. Associations between short-term exposure of ambient particulate matter and hemodialysis patients death: A nationwide, longitudinal case-control study in China. Sci Total Environ. 2022; 852: 158215. DOI: 10.1016/j.scitotenv.2022.158215 36028020

[19] Liu, Q, Huang K, Liang F, Yang X, Li J, Chen J, et al. Long-term exposure to fine particulate matter modifies the association between physical activity and hypertension incidence. J Sport Health Sci. 2022; 11(6): 708–715. DOI: 10.1016/j.jshs.2022.01.004 35065296 PMC9729921

[20] Vega E, Namdeo A, Bramwell L, Miquelajauregui Y, Resendiz-Martinez CG, Jaimes-Palomera M, et al. Changes in air quality in Mexico City, London and Delhi in response to various stages and levels of lockdowns and easing of restrictions during COVID-19 pandemic. Environ Pollut. 2021; 285: 117664. DOI: 10.1016/j.envpol.2021.117664 34380230 PMC8802357

[21] Hayes RB, Lim C, Zhang Y, Cromar K, Shao Y, Reynolds HR, et al. PM2.5 air pollution and cause-specific cardiovascular disease mortality. Int J Epidemiol, 2020. 49(1): pp. 25–35. DOI: 10.1093/ije/dyz114 31289812 PMC7124502

[22] Guo JZ, Wu QJ, Liu FH, Gao C, Gong TT, Li G. Review of mendelian randomization studies on endometrial cancer. Front Endocrinol (Lausanne). 2022; 13: 783150. DOI: 10.3389/fendo.2022.783150 35615721 PMC9124776

[23] Sekula P, Del Greco MF, Pattaro C, Köttgen A. Mendelian randomization as an approach to assess causality using observational data. J Am Soc Nephrol. 2016; 27(11): 3253–3265. DOI: 10.1681/ASN.2016010098 27486138 PMC5084898

[24] Trinder M, Paquette M, Cermakova L, Ban MR, Hegele RA, Baass A, et al. Polygenic contribution to low-density lipoprotein cholesterol levels and cardiovascular risk in monogenic familial hypercholesterolemia. Circ Genom Precis Med. 2020; 13(5): 515–523. DOI: 10.1161/CIRCGEN.120.002919 33079599 PMC7889287

[25] Lawlor DA, Harbord RM, Sterne JA, Timpson N, Davey Smith G. Mendelian randomization: using genes as instruments for making causal inferences in epidemiology. Stat Med. 2008; 27(8): 1133–63. DOI: 10.1002/sim.3034 17886233

[26] Skrivankova VW, Richmond RC, Woolf BAR, Yarmolinsky J, Davies NM, Swanson SA, VanderWeele TJ, et al. Strengthening the reporting of observational studies in epidemiology using mendelian randomization: The STROBE-MR statement. JAMA. 2021; 326(16): 1614–1621. DOI: 10.1001/jama.2021.18236 34698778

[27] Wauters A, Dreyfuss C, Pochet S, Hendrick P, Berkenboom G, van de Borne P, et al. Acute exposure to diesel exhaust impairs nitric oxide-mediated endothelial vasomotor function by increasing endothelial oxidative stress. Hypertension. 2013; 62(2): 352–8. DOI: 10.1161/HYPERTENSIONAHA.111.00991 23798345

[28] Fiordelisi A, Piscitelli P, Trimarco B, Coscioni E, Iaccarino G, Sorriento D. The mechanisms of air pollution and particulate matter in cardiovascular diseases. Heart Fail Rev. 2017; 22(3): 337–347. DOI: 10.1007/s10741-017-9606-7 28303426

[29] Wu T, Ma Y, Wu X, Bai M, Peng Y, Cai W, et al. Association between particulate matter air pollution and cardiovascular disease mortality in Lanzhou, China. Environ Sci Pollut Res Int. 2019; 26(15): 15262–15272. DOI: 10.1007/s11356-019-04742-w 30929170

[30] Wang Y, Eliot MN, Wellenius GA. Short-term changes in ambient particulate matter and risk of stroke: A systematic review and meta-analysis. J Am Heart Assoc. 2014; 3(4). DOI: 10.1161/JAHA.114.000983 PMC4310387 25103204

[31] Yuan S, Mason AM, Carter P, Burgess S, Larsson SC. Homocysteine, B vitamins, and cardiovascular disease: A Mendelian randomization study. BMC Med. 2021; 19(1): 97. DOI: 10.1186/s12916-021-01977-8 33888102 PMC8063383

[32] Birney E. Mendelian randomization. Cold Spring Harb Perspect Med. 2022; 12(4). DOI: 10.1101/cshperspect.a041302 PMC9121891 34872952

[33] Hahad O, Rajagopalan S, Lelieveld J, Sørensen M, Kuntic M, Daiber A, et al. Noise and air pollution as risk factors for hypertension: Part II-Pathophysiologic Insight. Hypertension. 2023; 80(7): 1384–1392. Epub 2023 Apr 19. PMID: 37073733. PMCID: PMC10330112. DOI: 10.1161/HYPERTENSIONAHA.123.20617 37073733 PMC10330112

[34] Hahad O, Rajagopalan S, Lelieveld J, Sørensen M, Frenis K, Daiber A, et al. Noise and air pollution as risk factors for hypertension: part i-epidemiology. Hypertension. 2023; 80(7): 1375–1383. Epub 2023 Apr 19. PMID: 37073726. PMCID: PMC10330192. DOI: 10.1161/HYPERTENSIONAHA.122.18732 37073726 PMC10330192

[35] Faridi S, Brook RD, Yousefian F, Hassanvand MS, Nodehi RN, Shamsipour M, et al. Effects of respirators to reduce fine particulate matter exposures on blood pressure and heart rate variability: A systematic review and meta-analysis. Environ Pollut. 2022; 303: 119109. Epub 2022 Mar 8. PMID: 35271952. PMCID: PMC10411486. DOI: 10.1016/j.envpol.2022.119109 35271952 PMC10411486

[36] Faridi S, Allen RW, Brook RD, Yousefian F, Hassanvand MS, Carlsten C. An updated systematic review and meta-analysis on portable air cleaners and blood pressure: Recommendations for users and manufacturers. Ecotoxicol Environ Saf. 2023; 263: 115227. Epub 2023 Jul 6. PMID: 37421892. DOI: 10.1016/j.ecoenv.2023.115227 37421892

[37] Sagheer U, Al-Kindi, S, Abohashem, S, et al. Environmental pollution and cardiovascular disease: Part 1 of 2: Air Pollution. JACC Adv. 2024, 3(2). DOI: 10.1016/j.jacadv.2023.100805 PMC11198409 38939391

