# Supplementary material for: Causal Associations of Environmental Pollution and Cardiovascular Disease: A Two-Sample Mendelian Randomization Study
Source: Glob Heart. 2024 Jun 18;19(1):52. doi: 10.5334/gh.1331 (PMC11192098; doi:10.5334/gh.1331)
Supplement: Supplementary File. — Supplementary Tables and Supplementary Figures. [file gh-19-1-1331-s1.pdf]

Trait	ID	Cases
Hypertension	finn-b-I9_HYPTENS	55917
Ischemic heart disease	finn-b-I9_ISCHHEART	30952
Non-ischemic cardiomyopathy	finn-b-I9_NONISCHCAR	11400
Cardiomyopathy	finn-b-I9_CARDMYO	3100
Hypertrophic cardiomyopathy	finn-b-I9_HYPERTROCA	556
Valvular heart disease	finn-b-I9_VHD_EXNONE	38209
Pulmonary heart disease	finn-b-I9_PULMHEART	4564
Cardiac arrhytmias	finn-b-CARDIAC_ARRHY	32416
Coronary artery disease	ebi-a-GCST005195	122733
Coronary heart disease	ieu-a-7	60801
Heart failure	ebi-a-GCST009541	47309
Atrial fibrillation	ebi-a-GCST006414	60620
Myocardial infarction	ieu-a-798	43676
Average 24-hour sound level of	ukb-b-19490	NA
Particulate matter air pollution	ukb-b-10817	NA
Particulate matter air pollution	ukb-b-18469	NA
Nitrogen dioxide air pollution	ukb-b-9942	NA
Workplace full of chemical or ot	ukb-d-22610_2	5872
Workplace had a lot of cigarette	ukb-d-22611_2	14941
Worked with materials containi	ukb-d-22612_2	1262
Worked with paints, thinners or	ukb-d-22613_2	3723
Workplace had a lot of diesel ex	ukb-d-22615_2	3483

p-value threshold	5.00E-06
LD Rsq	0.001
Clumping distance (kb)	10000
Use proxies	Minimum LD Rsq value=0.8
Exclude palindromic SNPs	yes
Analysis methods	IVW（main analysis）、Wald ratio、Maximum l

Controls	Sample size	Number of variants	Year
162837	218754	16380466	2021
187840	218792	16380466	2021
175752	187152	16380358	2021
156711	159811	16380196	2021
218236	218792	16380466	2021
180583	218792	16380466	2021
214228	218792	16380466	2021
139739	172155	16380239	2021
424528	547261	7934254	2017
123504	184305	9455779	2015
930014	977323	7773021	2020
970216	1030836	33519037	2018
128199	171875	9289492	2015
NA	456380	9851867	2018
NA	423796	9851867	2018
NA	423796	9851867	2018
NA	456380	9851867	2018
82863	88735	12324484	2018
74862	89803	13566864	2018
75195	76457	9680277	2018
84995	88718	11525313	2018
85621	89104	11409239	2018

ikelihood、MR Egger、Weighted median、Weighted mode

Population	First author	Consortium	PubmedID
European	NA	FinnGen-R5	NA
European	NA	FinnGen-R5	NA
European	NA	FinnGen-R5	NA
European	NA	FinnGen-R5	NA
European	NA	FinnGen-R5	NA
European	NA	FinnGen-R5	NA
European	NA	FinnGen-R5	NA
European	NA	FinnGen-R5	NA
European	van der Harst P	CARDIoGRAMp	29212778
European	Nikpay	CARDIoGRAMp	26343387
European	Shah S	HERMES	31919418
European	Nielsen JB	AFGen	30061737
European	Nikpay	CARDIoGRAMp	26343387
European	Ben Elsworth	MRC-IEU	
European	Ben Elsworth	MRC-IEU	
European	Ben Elsworth	MRC-IEU	
European	Ben Elsworth	MRC-IEU	
European	Neale lab	UK Biobank	
European	Neale lab	UK Biobank	
European	Neale lab	UK Biobank	
European	Neale lab	UK Biobank	
European	Neale lab	UK Biobank	

SNP	CHR	POS	Exposure	samplesize.ex
rs1009402	8	8646438	Nitrogen dioxide air pollution	456380
rs1011627	9	22081397	Nitrogen dioxide air pollution	456380
rs1017229	2	58201202	Nitrogen dioxide air pollution	456380
rs1075045	11	130903582	Nitrogen dioxide air pollution	456380
rs1083851	11	45780039	Nitrogen dioxide air pollution	456380
rs1085260	16	49762462	Nitrogen dioxide air pollution	456380
rs1098373	9	120416066	Nitrogen dioxide air pollution	456380
rs1104924	12	28119596	Nitrogen dioxide air pollution	456380
rs1112882	1	227090717	Nitrogen dioxide air pollution	456380
rs1120476	1	150999737	Nitrogen dioxide air pollution	456380
rs1168101	2	135135102	Nitrogen dioxide air pollution	456380
rs1184458	14	34241004	Nitrogen dioxide air pollution	456380
rs1208981	1	91189933	Nitrogen dioxide air pollution	456380
rs1217106	8	64567670	Nitrogen dioxide air pollution	456380
rs1220359	6	396321	Nitrogen dioxide air pollution	456380
rs1245189	17	2247982	Nitrogen dioxide air pollution	456380
rs1318845	4	153001662	Nitrogen dioxide air pollution	456380
rs1325445	8	40707884	Nitrogen dioxide air pollution	456380
rs1342194	2	6777264	Nitrogen dioxide air pollution	456380
rs1372504	5	103749428	Nitrogen dioxide air pollution	456380
rs1378675	15	25488099	Nitrogen dioxide air pollution	456380
rs1751318	5	53585870	Nitrogen dioxide air pollution	456380
rs2058582	4	13892169	Nitrogen dioxide air pollution	456380
rs27152	5	153734582	Nitrogen dioxide air pollution	456380
rs2838761	21	46481557	Nitrogen dioxide air pollution	456380
rs2865594	2	69152401	Nitrogen dioxide air pollution	456380
rs2870984	2	21685654	Nitrogen dioxide air pollution	456380
rs329177	5	125130444	Nitrogen dioxide air pollution	456380
rs3462373	10	129281256	Nitrogen dioxide air pollution	456380
rs3562135	7	55380566	Nitrogen dioxide air pollution	456380
rs356543	2	164851354	Nitrogen dioxide air pollution	456380
rs3740390	10	104638480	Nitrogen dioxide air pollution	456380
rs3916875	19	45856820	Nitrogen dioxide air pollution	456380
rs4771073	13	27693827	Nitrogen dioxide air pollution	456380
rs4838594	10	49669142	Nitrogen dioxide air pollution	456380
rs533745	1	34419822	Nitrogen dioxide air pollution	456380
rs5564300	12	5773969	Nitrogen dioxide air pollution	456380
rs5597480	7	133771130	Nitrogen dioxide air pollution	456380
rs5611854	5	164465890	Nitrogen dioxide air pollution	456380
rs5729175	2	213625968	Nitrogen dioxide air pollution	456380
rs5882485	5	137976541	Nitrogen dioxide air pollution	456380

rs6174628	12	55968949	Nitrogen dioxide air pollution	456380
rs6177510	1	14054970	Nitrogen dioxide air pollution	456380
rs6206203	17	6254811	Nitrogen dioxide air pollution	456380
rs6211970	19	2279746	Nitrogen dioxide air pollution	456380
rs6245909	7	44268127	Nitrogen dioxide air pollution	456380
rs6667345	1	8197477	Nitrogen dioxide air pollution	456380
rs6749467	2	343517	Nitrogen dioxide air pollution	456380
rs6954825	7	101207035	Nitrogen dioxide air pollution	456380
rs7089175	10	2958311	Nitrogen dioxide air pollution	456380
rs714020	22	40493860	Nitrogen dioxide air pollution	456380
rs724643	7	88669096	Nitrogen dioxide air pollution	456380
rs7264243	18	45920421	Nitrogen dioxide air pollution	456380
rs7265589	13	112082676	Nitrogen dioxide air pollution	456380
rs7279542	16	84606181	Nitrogen dioxide air pollution	456380
rs7308584	7	30740079	Nitrogen dioxide air pollution	456380
rs7514956	1	74019696	Nitrogen dioxide air pollution	456380
rs7559133	15	83627623	Nitrogen dioxide air pollution	456380
rs7615084	3	174719405	Nitrogen dioxide air pollution	456380
rs7657249	4	105641593	Nitrogen dioxide air pollution	456380
rs7720573	8	10153460	Nitrogen dioxide air pollution	456380
rs7758613	8	143137894	Nitrogen dioxide air pollution	456380
rs7760633	6	87297558	Nitrogen dioxide air pollution	456380
rs7776279	6	163999770	Nitrogen dioxide air pollution	456380
rs7838740	16	54206097	Nitrogen dioxide air pollution	456380
rs7853976	12	12490981	Nitrogen dioxide air pollution	456380
rs7910200	10	15981388	Nitrogen dioxide air pollution	456380
rs7947504	6	20992197	Nitrogen dioxide air pollution	456380
rs8012387	3	148486829	Nitrogen dioxide air pollution	456380
rs8033978	8	27818236	Nitrogen dioxide air pollution	456380
rs9368527	6	27679445	Nitrogen dioxide air pollution	456380
rs1084627	12	16211780	Particulate matter air pollution (pm10)	423796
rs1096116	9	13603291	Particulate matter air pollution (pm10)	423796
rs1162153	14	103577789	Particulate matter air pollution (pm10)	423796
rs1171222	9	133311860	Particulate matter air pollution (pm10)	423796
rs1176711	6	159246615	Particulate matter air pollution (pm10)	423796
rs1219295	6	94818099	Particulate matter air pollution (pm10)	423796
rs1220359	6	396321	Particulate matter air pollution (pm10)	423796
rs1257117	10	127771554	Particulate matter air pollution (pm10)	423796
rs1450455	8	342124	Particulate matter air pollution (pm10)	423796
rs1497528	2	33098355	Particulate matter air pollution (pm10)	423796
rs1509937	10	64836267	Particulate matter air pollution (pm10)	423796
rs218514	18	1469513	Particulate matter air pollution (pm10)	423796

rs281223(	13	51264767	Particulate matter air pollution (pm10)	423796
rs347764(	15	57656713	Particulate matter air pollution (pm10)	423796
rs356652(	5	51178554	Particulate matter air pollution (pm10)	423796
rs481513{	20	286487	Particulate matter air pollution (pm10)	423796
rs570482(	7	151623218	Particulate matter air pollution (pm10)	423796
rs614471{	5	101445992	Particulate matter air pollution (pm10)	423796
rs621197(	19	2279746	Particulate matter air pollution (pm10)	423796
rs728195(	5	164643843	Particulate matter air pollution (pm10)	423796
rs732014·	8	11512667	Particulate matter air pollution (pm10)	423796
rs747565:	1	115028382	Particulate matter air pollution (pm10)	423796
rs750517	18	75220892	Particulate matter air pollution (pm10)	423796
rs790372:	13	50507971	Particulate matter air pollution (pm10)	423796
rs802301:	14	48380718	Particulate matter air pollution (pm10)	423796
rs803434	15	68925781	Particulate matter air pollution (pm10)	423796
rs109611(	9	13603291	Particulate matter air pollution (pm2.5)	423796
rs110423:	11	2051631	Particulate matter air pollution (pm2.5)	423796
rs116925:	8	93266536	Particulate matter air pollution (pm2.5)	423796
rs117878:	22	18475815	Particulate matter air pollution (pm2.5)	423796
rs118289:	7	75245569	Particulate matter air pollution (pm2.5)	423796
rs118558:	15	78008843	Particulate matter air pollution (pm2.5)	423796
rs121330(	1	91214714	Particulate matter air pollution (pm2.5)	423796
rs121710(	8	64567670	Particulate matter air pollution (pm2.5)	423796
rs122035!	6	396321	Particulate matter air pollution (pm2.5)	423796
rs128125:	12	62341242	Particulate matter air pollution (pm2.5)	423796
rs131884!	4	153001662	Particulate matter air pollution (pm2.5)	423796
rs137250·	5	103749428	Particulate matter air pollution (pm2.5)	423796
rs153737:	9	22099568	Particulate matter air pollution (pm2.5)	423796
rs169577!	16	82610053	Particulate matter air pollution (pm2.5)	423796
rs171039:	14	76507299	Particulate matter air pollution (pm2.5)	423796
rs176578{	7	17757841	Particulate matter air pollution (pm2.5)	423796
rs214153:	7	9253413	Particulate matter air pollution (pm2.5)	423796
rs229215(	16	49765133	Particulate matter air pollution (pm2.5)	423796
rs27152	5	153734582	Particulate matter air pollution (pm2.5	423796
rs357035	11	61439682	Particulate matter air pollution (pm2.5	423796
rs485452:	2	69051853	Particulate matter air pollution (pm2.5	423796
rs487230	1	55541174	Particulate matter air pollution (pm2.5	423796
rs560686	6	50597378	Particulate matter air pollution (pm2.5	423796
rs588248!	5	137976541	Particulate matter air pollution (pm2.5)	423796
rs613410(	20	11007208	Particulate matter air pollution (pm2.5)	423796
rs621197(	19	2279746	Particulate matter air pollution (pm2.5)	423796
rs621804(	2	200815608	Particulate matter air pollution (pm2.5)	423796
rs623686	5	80861930	Particulate matter air pollution (pm2.5)	423796

rs6248466	7	104872861	Particulate matter air pollution (pm2.5	423796
rs6432776	2	164785611	Particulate matter air pollution (pm2.5	423796
rs6547952	2	22045642	Particulate matter air pollution (pm2.5	423796
rs6749461	2	343517	Particulate matter air pollution (pm2.5	423796
rs6960011	7	90302199	Particulate matter air pollution (pm2.5	423796
rs718059	14	34234984	Particulate matter air pollution (pm2.5	423796
rs7264243	18	45920421	Particulate matter air pollution (pm2.5	423796
rs7265589	13	112082676	Particulate matter air pollution (pm2.5	423796
rs7270000	14	104709808	Particulate matter air pollution (pm2.5	423796
rs7280802	5	164479164	Particulate matter air pollution (pm2.5	423796
rs7514956	1	74019696	Particulate matter air pollution (pm2.5	423796
rs7573056	2	58229428	Particulate matter air pollution (pm2.5	423796
rs7720573	8	10153460	Particulate matter air pollution (pm2.5	423796
rs7725581	6	20833602	Particulate matter air pollution (pm2.5	423796
rs7776279	6	163999770	Particulate matter air pollution (pm2.5	423796
rs7823038	8	35010055	Particulate matter air pollution (pm2.5	423796
rs7853976	12	12490981	Particulate matter air pollution (pm2.5	423796
rs7854601	10	48642432	Particulate matter air pollution (pm2.5	423796
rs8012381	3	148486829	Particulate matter air pollution (pm2.5	423796
rs8614	17	27588806	Particulate matter air pollution (pm2.5	423796
rs9260734	6	29932666	Particulate matter air pollution (pm2.5	423796
rs9644485	8	138230163	Particulate matter air pollution (pm2.5	423796
rs987204	3	94242461	Particulate matter air pollution (pm2.5	423796
rs1030921	19	8779100	Average 24-hour sound level of noise p	456380
rs1175270	8	117910936	Average 24-hour sound level of noise p	456380
rs1433250	19	32674873	Average 24-hour sound level of noise p	456380
rs1443749	7	150414185	Average 24-hour sound level of noise p	456380
rs1502133	1	242381731	Average 24-hour sound level of noise p	456380
rs1759044	3	25466098	Average 24-hour sound level of noise p	456380
rs1891471	6	112596443	Average 24-hour sound level of noise p	456380
rs2714872	7	17938491	Average 24-hour sound level of noise p	456380
rs5762541	22	28666961	Average 24-hour sound level of noise p	456380
rs7147858	11	102269128	Average 24-hour sound level of noise p	456380
rs7241762	18	72116373	Average 24-hour sound level of noise p	456380
rs7492080	13	80597508	Average 24-hour sound level of noise p	456380
rs1009402	8	8646438	Nitrogen dioxide air pollution	456380
rs1011621	9	22081397	Nitrogen dioxide air pollution	456380
rs1017229	2	58201202	Nitrogen dioxide air pollution	456380
rs1075045	11	130903582	Nitrogen dioxide air pollution	456380
rs1083851	11	45780039	Nitrogen dioxide air pollution	456380
rs1085260	16	49762462	Nitrogen dioxide air pollution	456380
rs1098373	9	120416066	Nitrogen dioxide air pollution	456380

rs110492[illegible]	12	28119596	Nitrogen dioxide air pollution	456380
rs111288[illegible]	1	227090717	Nitrogen dioxide air pollution	456380
rs112047[illegible]	1	150999737	Nitrogen dioxide air pollution	456380
rs116810[illegible]	2	135135102	Nitrogen dioxide air pollution	456380
rs118091[illegible]	9	20205067	Nitrogen dioxide air pollution	456380
rs118445[illegible]	14	34241004	Nitrogen dioxide air pollution	456380
rs120898[illegible]	1	91189933	Nitrogen dioxide air pollution	456380
rs121710[illegible]	8	64567670	Nitrogen dioxide air pollution	456380
rs122035[illegible]	6	396321	Nitrogen dioxide air pollution	456380
rs124518[illegible]	17	2247982	Nitrogen dioxide air pollution	456380
rs131884[illegible]	4	153001662	Nitrogen dioxide air pollution	456380
rs132544[illegible]	8	40707884	Nitrogen dioxide air pollution	456380
rs134219[illegible]	2	6777264	Nitrogen dioxide air pollution	456380
rs137250[illegible]	5	103749428	Nitrogen dioxide air pollution	456380
rs137867[illegible]	15	25488099	Nitrogen dioxide air pollution	456380
rs171838[illegible]	18	3100049	Nitrogen dioxide air pollution	456380
rs175131[illegible]	5	53585870	Nitrogen dioxide air pollution	456380
rs205858[illegible]	4	13892169	Nitrogen dioxide air pollution	456380
rs27152	5	153734582	Nitrogen dioxide air pollution	456380
rs283876[illegible]	21	46481557	Nitrogen dioxide air pollution	456380
rs286559[illegible]	2	69152401	Nitrogen dioxide air pollution	456380
rs287098[illegible]	2	21685654	Nitrogen dioxide air pollution	456380
rs329177	5	125130444	Nitrogen dioxide air pollution	456380
rs346237[illegible]	10	129281256	Nitrogen dioxide air pollution	456380
rs356213[illegible]	7	55380566	Nitrogen dioxide air pollution	456380
rs356543	2	164851354	Nitrogen dioxide air pollution	456380
rs374039[illegible]	10	104638480	Nitrogen dioxide air pollution	456380
rs391687[illegible]	19	45856820	Nitrogen dioxide air pollution	456380
rs477107[illegible]	13	27693827	Nitrogen dioxide air pollution	456380
rs483859[illegible]	10	49669142	Nitrogen dioxide air pollution	456380
rs533745	1	34419822	Nitrogen dioxide air pollution	456380
rs556430[illegible]	12	5773969	Nitrogen dioxide air pollution	456380
rs559748[illegible]	7	133771130	Nitrogen dioxide air pollution	456380
rs561185[illegible]	5	164465890	Nitrogen dioxide air pollution	456380
rs572917[illegible]	2	213625968	Nitrogen dioxide air pollution	456380
rs588248[illegible]	5	137976541	Nitrogen dioxide air pollution	456380
rs617462[illegible]	12	55968949	Nitrogen dioxide air pollution	456380
rs617751[illegible]	1	14054970	Nitrogen dioxide air pollution	456380
rs620620[illegible]	17	6254811	Nitrogen dioxide air pollution	456380
rs621197[illegible]	19	2279746	Nitrogen dioxide air pollution	456380
rs624590[illegible]	7	44268127	Nitrogen dioxide air pollution	456380
rs666734[illegible]	1	8197477	Nitrogen dioxide air pollution	456380

rs674946[illegible]	2	343517	Nitrogen dioxide air pollution	456380
rs695482[illegible]	7	101207035	Nitrogen dioxide air pollution	456380
rs708917[illegible]	10	2958311	Nitrogen dioxide air pollution	456380
rs714020	22	40493860	Nitrogen dioxide air pollution	456380
rs724643	7	88669096	Nitrogen dioxide air pollution	456380
rs726424[illegible]	18	45920421	Nitrogen dioxide air pollution	456380
rs726558[illegible]	13	112082676	Nitrogen dioxide air pollution	456380
rs727954[illegible]	16	84606181	Nitrogen dioxide air pollution	456380
rs730858[illegible]	7	30740079	Nitrogen dioxide air pollution	456380
rs751495[illegible]	1	74019696	Nitrogen dioxide air pollution	456380
rs755913[illegible]	15	83627623	Nitrogen dioxide air pollution	456380
rs761508[illegible]	3	174719405	Nitrogen dioxide air pollution	456380
rs765724[illegible]	4	105641593	Nitrogen dioxide air pollution	456380
rs772057[illegible]	8	10153460	Nitrogen dioxide air pollution	456380
rs775861[illegible]	8	143137894	Nitrogen dioxide air pollution	456380
rs776063[illegible]	6	87297558	Nitrogen dioxide air pollution	456380
rs777627[illegible]	6	163999770	Nitrogen dioxide air pollution	456380
rs783874[illegible]	16	54206097	Nitrogen dioxide air pollution	456380
rs785397[illegible]	12	12490981	Nitrogen dioxide air pollution	456380
rs791020[illegible]	10	15981388	Nitrogen dioxide air pollution	456380
rs794750[illegible]	6	20992197	Nitrogen dioxide air pollution	456380
rs801238[illegible]	3	148486829	Nitrogen dioxide air pollution	456380
rs803397[illegible]	8	27818236	Nitrogen dioxide air pollution	456380
rs936852[illegible]	6	27679445	Nitrogen dioxide air pollution	456380
rs109611[illegible]	9	13603291	Particulate matter air pollution (pm2.5	423796
rs110423[illegible]	11	2051631	Particulate matter air pollution (pm2.5	423796
rs116925[illegible]	8	93266536	Particulate matter air pollution (pm2.5	423796
rs117878[illegible]	22	18475815	Particulate matter air pollution (pm2.5	423796
rs118289[illegible]	7	75245569	Particulate matter air pollution (pm2.5	423796
rs118558[illegible]	15	78008843	Particulate matter air pollution (pm2.5	423796
rs121330[illegible]	1	91214714	Particulate matter air pollution (pm2.5	423796
rs121710[illegible]	8	64567670	Particulate matter air pollution (pm2.5	423796
rs122035[illegible]	6	396321	Particulate matter air pollution (pm2.5	423796
rs128125[illegible]	12	62341242	Particulate matter air pollution (pm2.5	423796
rs131884[illegible]	4	153001662	Particulate matter air pollution (pm2.5	423796
rs137250[illegible]	5	103749428	Particulate matter air pollution (pm2.5	423796
rs153737[illegible]	9	22099568	Particulate matter air pollution (pm2.5	423796
rs169577[illegible]	16	82610053	Particulate matter air pollution (pm2.5	423796
rs171039[illegible]	14	76507299	Particulate matter air pollution (pm2.5	423796
rs171838[illegible]	18	3100049	Particulate matter air pollution (pm2.5	423796
rs176578[illegible]	7	17757841	Particulate matter air pollution (pm2.5	423796
rs214153[illegible]	7	9253413	Particulate matter air pollution (pm2.5	423796

rs2292156	16	49765133	Particulate matter air pollution (pm2.5	423796
rs27152	5	153734582	Particulate matter air pollution (pm2.5	423796
rs3570357	11	61439682	Particulate matter air pollution (pm2.5	423796
rs4854523	2	69051853	Particulate matter air pollution (pm2.5	423796
rs487230	1	55541174	Particulate matter air pollution (pm2.5	423796
rs5606867	6	50597378	Particulate matter air pollution (pm2.5	423796
rs5882485	5	137976541	Particulate matter air pollution (pm2.5	423796
rs6134106	20	11007208	Particulate matter air pollution (pm2.5	423796
rs6211970	19	2279746	Particulate matter air pollution (pm2.5	423796
rs6218046	2	200815608	Particulate matter air pollution (pm2.5	423796
rs6236867	5	80861930	Particulate matter air pollution (pm2.5	423796
rs6248466	7	104872861	Particulate matter air pollution (pm2.5	423796
rs6432776	2	164785611	Particulate matter air pollution (pm2.5	423796
rs6547952	2	22045642	Particulate matter air pollution (pm2.5	423796
rs6749467	2	343517	Particulate matter air pollution (pm2.5	423796
rs6960011	7	90302199	Particulate matter air pollution (pm2.5	423796
rs718059	14	34234984	Particulate matter air pollution (pm2.5	423796
rs7264243	18	45920421	Particulate matter air pollution (pm2.5	423796
rs7265589	13	112082676	Particulate matter air pollution (pm2.5	423796
rs7270000	14	104709808	Particulate matter air pollution (pm2.5	423796
rs7280802	5	164479164	Particulate matter air pollution (pm2.5	423796
rs7514956	1	74019696	Particulate matter air pollution (pm2.5	423796
rs7573056	2	58229428	Particulate matter air pollution (pm2.5	423796
rs7720573	8	10153460	Particulate matter air pollution (pm2.5	423796
rs7725581	6	20833602	Particulate matter air pollution (pm2.5	423796
rs7776279	6	163999770	Particulate matter air pollution (pm2.5	423796
rs7823038	8	35010055	Particulate matter air pollution (pm2.5	423796
rs7853976	12	12490981	Particulate matter air pollution (pm2.5	423796
rs7854607	10	48642432	Particulate matter air pollution (pm2.5	423796
rs8012387	3	148486829	Particulate matter air pollution (pm2.5	423796
rs8614	17	27588806	Particulate matter air pollution (pm2.5	423796
rs9260734	6	29932666	Particulate matter air pollution (pm2.5	423796
rs9644485	8	138230163	Particulate matter air pollution (pm2.5	423796
rs987204	3	94242461	Particulate matter air pollution (pm2.5	423796
rs1084627	12	16211780	Particulate matter air pollution (pm10)	423796
rs1096116	9	13603291	Particulate matter air pollution (pm10)	423796
rs1162153	14	103577789	Particulate matter air pollution (pm10)	423796
rs1171222	9	133311860	Particulate matter air pollution (pm10)	423796
rs1176711	6	159246615	Particulate matter air pollution (pm10)	423796
rs1219295	6	94818099	Particulate matter air pollution (pm10)	423796
rs1220359	6	396321	Particulate matter air pollution (pm10)	423796
rs1257117	10	127771554	Particulate matter air pollution (pm10)	423796

rs145045!	8	342124	Particulate matter air pollution (pm10)	423796
rs149130(	11	83722884	Particulate matter air pollution (pm10)	423796
rs149752!	2	33098355	Particulate matter air pollution (pm10)	423796
rs150993:	10	64836267	Particulate matter air pollution (pm10)	423796
rs218514	18	1469513	Particulate matter air pollution (pm10)	423796
rs281223(	13	51264767	Particulate matter air pollution (pm10)	423796
rs347764(	15	57656713	Particulate matter air pollution (pm10)	423796
rs356652(	5	51178554	Particulate matter air pollution (pm10)	423796
rs481513!	20	286487	Particulate matter air pollution (pm10)	423796
rs570482(	7	151623218	Particulate matter air pollution (pm10)	423796
rs614471!	5	101445992	Particulate matter air pollution (pm10)	423796
rs621197(	19	2279746	Particulate matter air pollution (pm10)	423796
rs728195(	5	164643843	Particulate matter air pollution (pm10)	423796
rs732014	8	11512667	Particulate matter air pollution (pm10)	423796
rs747565:	1	115028382	Particulate matter air pollution (pm10)	423796
rs750517:	18	75220892	Particulate matter air pollution (pm10)	423796
rs790372:	13	50507971	Particulate matter air pollution (pm10)	423796
rs802301:	14	48380718	Particulate matter air pollution (pm10)	423796
rs803434:	15	68925781	Particulate matter air pollution (pm10)	423796
rs103092:	19	8779100	Average 24-hour sound level of noise p	456380
rs117527(	8	117910936	Average 24-hour sound level of noise p	456380
rs143325(	19	32674873	Average 24-hour sound level of noise p	456380
rs144374!	7	150414185	Average 24-hour sound level of noise p	456380
rs150213:	1	242381731	Average 24-hour sound level of noise p	456380
rs175904	3	25466098	Average 24-hour sound level of noise p	456380
rs189147:	6	112596443	Average 24-hour sound level of noise p	456380
rs271487:	7	17938491	Average 24-hour sound level of noise p	456380
rs576254:	22	28666961	Average 24-hour sound level of noise p	456380
rs714785!	11	102269128	Average 24-hour sound level of noise p	456380
rs724176:	18	72116373	Average 24-hour sound level of noise p	456380
rs749208(	13	80597508	Average 24-hour sound level of noise p	456380
rs109611(	9	13603291	Particulate matter air pollution (pm2.5	423796
rs110423:	11	2051631	Particulate matter air pollution (pm2.5	423796
rs118289:	7	75245569	Particulate matter air pollution (pm2.5	423796
rs118558:	15	78008843	Particulate matter air pollution (pm2.5	423796
rs121330(	1	91214714	Particulate matter air pollution (pm2.5	423796
rs121710(	8	64567670	Particulate matter air pollution (pm2.5	423796
rs122035!	6	396321	Particulate matter air pollution (pm2.5	423796
rs128125:	12	62341242	Particulate matter air pollution (pm2.5	423796
rs131884!	4	153001662	Particulate matter air pollution (pm2.5	423796
rs137250	5	103749428	Particulate matter air pollution (pm2.5	423796
rs153737:	9	22099568	Particulate matter air pollution (pm2.5	423796

rs1695775	16	82610053	Particulate matter air pollution (pm2.5	423796
rs1710392	14	76507299	Particulate matter air pollution (pm2.5	423796
rs1718385	18	3100049	Particulate matter air pollution (pm2.5	423796
rs1765788	7	17757841	Particulate matter air pollution (pm2.5	423796
rs2141531	7	9253413	Particulate matter air pollution (pm2.5	423796
rs2292156	16	49765133	Particulate matter air pollution (pm2.5	423796
rs27152	5	153734582	Particulate matter air pollution (pm2.5	423796
rs3570357	11	61439682	Particulate matter air pollution (pm2.5	423796
rs4854523	2	69051853	Particulate matter air pollution (pm2.5	423796
rs487230	1	55541174	Particulate matter air pollution (pm2.5	423796
rs5606867	6	50597378	Particulate matter air pollution (pm2.5	423796
rs5882485	5	137976541	Particulate matter air pollution (pm2.5	423796
rs6211970	19	2279746	Particulate matter air pollution (pm2.5	423796
rs6218046	2	200815608	Particulate matter air pollution (pm2.5	423796
rs6236867	5	80861930	Particulate matter air pollution (pm2.5	423796
rs6248466	7	104872861	Particulate matter air pollution (pm2.5	423796
rs6432776	2	164785611	Particulate matter air pollution (pm2.5	423796
rs6547952	2	22045642	Particulate matter air pollution (pm2.5	423796
rs6749467	2	343517	Particulate matter air pollution (pm2.5	423796
rs6960011	7	90302199	Particulate matter air pollution (pm2.5	423796
rs718059	14	34234984	Particulate matter air pollution (pm2.5	423796
rs7264243	18	45920421	Particulate matter air pollution (pm2.5	423796
rs7265589	13	112082676	Particulate matter air pollution (pm2.5	423796
rs7270000	14	104709808	Particulate matter air pollution (pm2.5	423796
rs7280802	5	164479164	Particulate matter air pollution (pm2.5	423796
rs7514956	1	74019696	Particulate matter air pollution (pm2.5	423796
rs7573056	2	58229428	Particulate matter air pollution (pm2.5	423796
rs7720573	8	10153460	Particulate matter air pollution (pm2.5	423796
rs7725581	6	20833602	Particulate matter air pollution (pm2.5	423796
rs7776279	6	163999770	Particulate matter air pollution (pm2.5	423796
rs7853976	12	12490981	Particulate matter air pollution (pm2.5	423796
rs8012387	3	148486829	Particulate matter air pollution (pm2.5	423796
rs8614	17	27588806	Particulate matter air pollution (pm2.5	423796
rs9260734	6	29932666	Particulate matter air pollution (pm2.5	423796
rs9644485	8	138230163	Particulate matter air pollution (pm2.5	423796
rs987204	3	94242461	Particulate matter air pollution (pm2.5	423796
rs1009402	8	8646438	Nitrogen dioxide air pollution	456380
rs1011627	9	22081397	Nitrogen dioxide air pollution	456380
rs1017229	2	58201202	Nitrogen dioxide air pollution	456380
rs1075045	11	130903582	Nitrogen dioxide air pollution	456380
rs1083851	11	45780039	Nitrogen dioxide air pollution	456380
rs1085260	16	49762462	Nitrogen dioxide air pollution	456380

rs109837	9	120416066	Nitrogen dioxide air pollution	456380
rs110492	12	28119596	Nitrogen dioxide air pollution	456380
rs111288	1	227090717	Nitrogen dioxide air pollution	456380
rs112047	1	150999737	Nitrogen dioxide air pollution	456380
rs116810	2	135135102	Nitrogen dioxide air pollution	456380
rs118445	14	34241004	Nitrogen dioxide air pollution	456380
rs120898	1	91189933	Nitrogen dioxide air pollution	456380
rs121710	8	64567670	Nitrogen dioxide air pollution	456380
rs122035	6	396321	Nitrogen dioxide air pollution	456380
rs124518	17	2247982	Nitrogen dioxide air pollution	456380
rs131884	4	153001662	Nitrogen dioxide air pollution	456380
rs132544	8	40707884	Nitrogen dioxide air pollution	456380
rs134219	2	6777264	Nitrogen dioxide air pollution	456380
rs137250	5	103749428	Nitrogen dioxide air pollution	456380
rs137867	15	25488099	Nitrogen dioxide air pollution	456380
rs171838	18	3100049	Nitrogen dioxide air pollution	456380
rs175131	5	53585870	Nitrogen dioxide air pollution	456380
rs205858	4	13892169	Nitrogen dioxide air pollution	456380
rs27152	5	153734582	Nitrogen dioxide air pollution	456380
rs283876	21	46481557	Nitrogen dioxide air pollution	456380
rs286559	2	69152401	Nitrogen dioxide air pollution	456380
rs287098	2	21685654	Nitrogen dioxide air pollution	456380
rs329177	5	125130444	Nitrogen dioxide air pollution	456380
rs346237	10	129281256	Nitrogen dioxide air pollution	456380
rs356213	7	55380566	Nitrogen dioxide air pollution	456380
rs356543	2	164851354	Nitrogen dioxide air pollution	456380
rs374039	10	104638480	Nitrogen dioxide air pollution	456380
rs391687	19	45856820	Nitrogen dioxide air pollution	456380
rs477107	13	27693827	Nitrogen dioxide air pollution	456380
rs483859	10	49669142	Nitrogen dioxide air pollution	456380
rs533745	1	34419822	Nitrogen dioxide air pollution	456380
rs556430	12	5773969	Nitrogen dioxide air pollution	456380
rs559748	7	133771130	Nitrogen dioxide air pollution	456380
rs561185	5	164465890	Nitrogen dioxide air pollution	456380
rs572917	2	213625968	Nitrogen dioxide air pollution	456380
rs588248	5	137976541	Nitrogen dioxide air pollution	456380
rs617462	12	55968949	Nitrogen dioxide air pollution	456380
rs617751	1	14054970	Nitrogen dioxide air pollution	456380
rs620620	17	6254811	Nitrogen dioxide air pollution	456380
rs621197	19	2279746	Nitrogen dioxide air pollution	456380
rs624590	7	44268127	Nitrogen dioxide air pollution	456380
rs666734	1	8197477	Nitrogen dioxide air pollution	456380

rs674946[illegible]	2	343517	Nitrogen dioxide air pollution	456380
rs695482[illegible]	7	101207035	Nitrogen dioxide air pollution	456380
rs708917[illegible]	10	2958311	Nitrogen dioxide air pollution	456380
rs714020	22	40493860	Nitrogen dioxide air pollution	456380
rs724643	7	88669096	Nitrogen dioxide air pollution	456380
rs726424[illegible]	18	45920421	Nitrogen dioxide air pollution	456380
rs726558[illegible]	13	112082676	Nitrogen dioxide air pollution	456380
rs727954[illegible]	16	84606181	Nitrogen dioxide air pollution	456380
rs730858[illegible]	7	30740079	Nitrogen dioxide air pollution	456380
rs751495[illegible]	1	74019696	Nitrogen dioxide air pollution	456380
rs755913[illegible]	15	83627623	Nitrogen dioxide air pollution	456380
rs761508[illegible]	3	174719405	Nitrogen dioxide air pollution	456380
rs765724[illegible]	4	105641593	Nitrogen dioxide air pollution	456380
rs772057[illegible]	8	10153460	Nitrogen dioxide air pollution	456380
rs775861[illegible]	8	143137894	Nitrogen dioxide air pollution	456380
rs776063[illegible]	6	87297558	Nitrogen dioxide air pollution	456380
rs777627[illegible]	6	163999770	Nitrogen dioxide air pollution	456380
rs783874[illegible]	16	54206097	Nitrogen dioxide air pollution	456380
rs785397[illegible]	12	12490981	Nitrogen dioxide air pollution	456380
rs791020[illegible]	10	15981388	Nitrogen dioxide air pollution	456380
rs794750[illegible]	6	20992197	Nitrogen dioxide air pollution	456380
rs801238[illegible]	3	148486829	Nitrogen dioxide air pollution	456380
rs803397[illegible]	8	27818236	Nitrogen dioxide air pollution	456380
rs936852[illegible]	6	27679445	Nitrogen dioxide air pollution	456380
rs103092[illegible]	19	8779100	Average 24-hour sound level of noise [illegible]	456380
rs143325[illegible]	19	32674873	Average 24-hour sound level of noise [illegible]	456380
rs144374[illegible]	7	150434689	Average 24-hour sound level of noise [illegible]	456380
rs150213[illegible]	1	242381731	Average 24-hour sound level of noise [illegible]	456380
rs175904[illegible]	3	25466098	Average 24-hour sound level of noise [illegible]	456380
rs189147[illegible]	6	112596443	Average 24-hour sound level of noise [illegible]	456380
rs271487[illegible]	7	17938491	Average 24-hour sound level of noise [illegible]	456380
rs576254[illegible]	22	28666961	Average 24-hour sound level of noise [illegible]	456380
rs714785[illegible]	11	102269128	Average 24-hour sound level of noise [illegible]	456380
rs724176[illegible]	18	72116373	Average 24-hour sound level of noise [illegible]	456380
rs749208[illegible]	13	80597508	Average 24-hour sound level of noise [illegible]	456380
rs108462[illegible]	12	16211780	Particulate matter air pollution (pm10[illegible]	423796
rs109611[illegible]	9	13603291	Particulate matter air pollution (pm10[illegible]	423796
rs116215[illegible]	14	103577789	Particulate matter air pollution (pm10[illegible]	423796
rs117122[illegible]	9	133311860	Particulate matter air pollution (pm10[illegible]	423796
rs117671[illegible]	6	159246615	Particulate matter air pollution (pm10[illegible]	423796
rs121929[illegible]	6	94818099	Particulate matter air pollution (pm10[illegible]	423796
rs122035[illegible]	6	396321	Particulate matter air pollution (pm10[illegible]	423796

rs1257117	10	127771554	Particulate matter air pollution (pm10)	423796
rs1450455	8	342124	Particulate matter air pollution (pm10)	423796
rs1497528	2	33098355	Particulate matter air pollution (pm10)	423796
rs1509937	10	64836267	Particulate matter air pollution (pm10)	423796
rs218514	18	1469513	Particulate matter air pollution (pm10)	423796
rs2812230	13	51264767	Particulate matter air pollution (pm10)	423796
rs3477646	15	57656713	Particulate matter air pollution (pm10)	423796
rs3566526	5	51178554	Particulate matter air pollution (pm10)	423796
rs4815138	20	286487	Particulate matter air pollution (pm10)	423796
rs5704826	7	151623218	Particulate matter air pollution (pm10)	423796
rs6144718	5	101445992	Particulate matter air pollution (pm10)	423796
rs6211970	19	2279746	Particulate matter air pollution (pm10)	423796
rs7281956	5	164643843	Particulate matter air pollution (pm10)	423796
rs7320144	8	11512667	Particulate matter air pollution (pm10)	423796
rs7505177	18	75220892	Particulate matter air pollution (pm10)	423796
rs7903722	13	50507971	Particulate matter air pollution (pm10)	423796
rs8023013	14	48380718	Particulate matter air pollution (pm10)	423796
rs8034347	15	68925781	Particulate matter air pollution (pm10)	423796
rs1084627	12	16211780	Particulate matter air pollution (pm10)	423796
rs1096116	9	13603291	Particulate matter air pollution (pm10)	423796
rs1162153	14	103577789	Particulate matter air pollution (pm10)	423796
rs1171222	9	133311860	Particulate matter air pollution (pm10)	423796
rs1176711	6	159246615	Particulate matter air pollution (pm10)	423796
rs1219295	6	94818099	Particulate matter air pollution (pm10)	423796
rs1220359	6	396321	Particulate matter air pollution (pm10)	423796
rs1257117	10	127771554	Particulate matter air pollution (pm10)	423796
rs1450455	8	342124	Particulate matter air pollution (pm10)	423796
rs1491306	11	83722884	Particulate matter air pollution (pm10)	423796
rs1497528	2	33098355	Particulate matter air pollution (pm10)	423796
rs1509937	10	64836267	Particulate matter air pollution (pm10)	423796
rs218514	18	1469513	Particulate matter air pollution (pm10)	423796
rs2812230	13	51264767	Particulate matter air pollution (pm10)	423796
rs3477646	15	57656713	Particulate matter air pollution (pm10)	423796
rs3566526	5	51178554	Particulate matter air pollution (pm10)	423796
rs4815138	20	286487	Particulate matter air pollution (pm10)	423796
rs5704826	7	151623218	Particulate matter air pollution (pm10)	423796
rs6144718	5	101445992	Particulate matter air pollution (pm10)	423796
rs6211970	19	2279746	Particulate matter air pollution (pm10)	423796
rs7281956	5	164643843	Particulate matter air pollution (pm10)	423796
rs7320144	8	11512667	Particulate matter air pollution (pm10)	423796
rs7475651	1	115028382	Particulate matter air pollution (pm10)	423796
rs7505177	18	75220892	Particulate matter air pollution (pm10)	423796

rs790372[illegible]	13	50507971	Particulate matter air pollution (pm10[illegible]	423796
rs802301[illegible]	14	48380718	Particulate matter air pollution (pm10[illegible]	423796
rs803434[illegible]	15	68925781	Particulate matter air pollution (pm10[illegible]	423796
rs100940[illegible]	8	8646438	Nitrogen dioxide air pollution	456380
rs101162[illegible]	9	22081397	Nitrogen dioxide air pollution	456380
rs101722[illegible]	2	58201202	Nitrogen dioxide air pollution	456380
rs107504[illegible]	11	130903582	Nitrogen dioxide air pollution	456380
rs108385[illegible]	11	45780039	Nitrogen dioxide air pollution	456380
rs108526[illegible]	16	49762462	Nitrogen dioxide air pollution	456380
rs109837[illegible]	9	120416066	Nitrogen dioxide air pollution	456380
rs110492[illegible]	12	28119596	Nitrogen dioxide air pollution	456380
rs111288[illegible]	1	227090717	Nitrogen dioxide air pollution	456380
rs112047[illegible]	1	150999737	Nitrogen dioxide air pollution	456380
rs116810[illegible]	2	135135102	Nitrogen dioxide air pollution	456380
rs118091[illegible]	9	20205067	Nitrogen dioxide air pollution	456380
rs118445[illegible]	14	34241004	Nitrogen dioxide air pollution	456380
rs120898[illegible]	1	91189933	Nitrogen dioxide air pollution	456380
rs121710[illegible]	8	64567670	Nitrogen dioxide air pollution	456380
rs122035[illegible]	6	396321	Nitrogen dioxide air pollution	456380
rs124518[illegible]	17	2247982	Nitrogen dioxide air pollution	456380
rs131884[illegible]	4	153001662	Nitrogen dioxide air pollution	456380
rs132544[illegible]	8	40707884	Nitrogen dioxide air pollution	456380
rs134219[illegible]	2	6777264	Nitrogen dioxide air pollution	456380
rs137250[illegible]	5	103749428	Nitrogen dioxide air pollution	456380
rs137867[illegible]	15	25488099	Nitrogen dioxide air pollution	456380
rs171838[illegible]	18	3100049	Nitrogen dioxide air pollution	456380
rs175131[illegible]	5	53585870	Nitrogen dioxide air pollution	456380
rs205858[illegible]	4	13892169	Nitrogen dioxide air pollution	456380
rs27152	5	153734582	Nitrogen dioxide air pollution	456380
rs283876[illegible]	21	46481557	Nitrogen dioxide air pollution	456380
rs286559[illegible]	2	69152401	Nitrogen dioxide air pollution	456380
rs287098[illegible]	2	21685654	Nitrogen dioxide air pollution	456380
rs329177	5	125130444	Nitrogen dioxide air pollution	456380
rs346237[illegible]	10	129281256	Nitrogen dioxide air pollution	456380
rs356213[illegible]	7	55380566	Nitrogen dioxide air pollution	456380
rs356543	2	164851354	Nitrogen dioxide air pollution	456380
rs374039[illegible]	10	104638480	Nitrogen dioxide air pollution	456380
rs391687[illegible]	19	45856820	Nitrogen dioxide air pollution	456380
rs477107[illegible]	13	27693827	Nitrogen dioxide air pollution	456380
rs483859[illegible]	10	49669142	Nitrogen dioxide air pollution	456380
rs533745	1	34419822	Nitrogen dioxide air pollution	456380
rs556430[illegible]	12	5773969	Nitrogen dioxide air pollution	456380

rs559748	7	133771130	Nitrogen dioxide air pollution	456380
rs561185	5	164465890	Nitrogen dioxide air pollution	456380
rs572917	2	213625968	Nitrogen dioxide air pollution	456380
rs588248	5	137976541	Nitrogen dioxide air pollution	456380
rs617462	12	55968949	Nitrogen dioxide air pollution	456380
rs617751	1	14054970	Nitrogen dioxide air pollution	456380
rs620620	17	6255977	Nitrogen dioxide air pollution	456380
rs621197	19	2279746	Nitrogen dioxide air pollution	456380
rs624590	7	44268127	Nitrogen dioxide air pollution	456380
rs674946	2	343517	Nitrogen dioxide air pollution	456380
rs695482	7	101207035	Nitrogen dioxide air pollution	456380
rs708917	10	2958311	Nitrogen dioxide air pollution	456380
rs714020	22	40493860	Nitrogen dioxide air pollution	456380
rs724643	7	88669096	Nitrogen dioxide air pollution	456380
rs726424	18	45920421	Nitrogen dioxide air pollution	456380
rs726558	13	112082676	Nitrogen dioxide air pollution	456380
rs727954	16	84606181	Nitrogen dioxide air pollution	456380
rs730858	7	30740079	Nitrogen dioxide air pollution	456380
rs751495	1	74019696	Nitrogen dioxide air pollution	456380
rs755913	15	83627623	Nitrogen dioxide air pollution	456380
rs761508	3	174719405	Nitrogen dioxide air pollution	456380
rs765724	4	105641593	Nitrogen dioxide air pollution	456380
rs772057	8	10153460	Nitrogen dioxide air pollution	456380
rs775861	8	143137894	Nitrogen dioxide air pollution	456380
rs776063	6	87298301	Nitrogen dioxide air pollution	456380
rs777627	6	163999770	Nitrogen dioxide air pollution	456380
rs783874	16	54206097	Nitrogen dioxide air pollution	456380
rs785397	12	12490981	Nitrogen dioxide air pollution	456380
rs791020	10	15981388	Nitrogen dioxide air pollution	456380
rs801238	3	148486829	Nitrogen dioxide air pollution	456380
rs803397	8	27818236	Nitrogen dioxide air pollution	456380
rs936852	6	27679445	Nitrogen dioxide air pollution	456380
rs109611	9	13603291	Particulate matter air pollution (pm2.5	423796
rs116925	8	93266536	Particulate matter air pollution (pm2.5	423796
rs117878	22	18475815	Particulate matter air pollution (pm2.5	423796
rs118289	7	75245569	Particulate matter air pollution (pm2.5	423796
rs118558	15	78012618	Particulate matter air pollution (pm2.5	423796
rs121330	1	91214714	Particulate matter air pollution (pm2.5	423796
rs121710	8	64567670	Particulate matter air pollution (pm2.5	423796
rs122035	6	396321	Particulate matter air pollution (pm2.5	423796
rs131884	4	153001662	Particulate matter air pollution (pm2.5	423796
rs137250	5	103749428	Particulate matter air pollution (pm2.5	423796

rs1537371	9	22099568	Particulate matter air pollution (pm2.5	423796
rs1695775	16	82610053	Particulate matter air pollution (pm2.5	423796
rs1710392	14	76507299	Particulate matter air pollution (pm2.5	423796
rs1718385	18	3100049	Particulate matter air pollution (pm2.5	423796
rs1765788	7	17757841	Particulate matter air pollution (pm2.5	423796
rs2141531	7	9253413	Particulate matter air pollution (pm2.5	423796
rs2292156	16	49765133	Particulate matter air pollution (pm2.5	423796
rs27152	5	153734582	Particulate matter air pollution (pm2.5	423796
rs3570357	11	61439682	Particulate matter air pollution (pm2.5	423796
rs4854523	2	69051853	Particulate matter air pollution (pm2.5	423796
rs487230	1	55541174	Particulate matter air pollution (pm2.5	423796
rs5606867	6	50597378	Particulate matter air pollution (pm2.5	423796
rs5882485	5	137976541	Particulate matter air pollution (pm2.5	423796
rs6134106	20	11007208	Particulate matter air pollution (pm2.5	423796
rs6211970	19	2279746	Particulate matter air pollution (pm2.5	423796
rs6218046	2	200815608	Particulate matter air pollution (pm2.5	423796
rs6236867	5	80861930	Particulate matter air pollution (pm2.5	423796
rs6248466	7	104984561	Particulate matter air pollution (pm2.5	423796
rs6432776	2	164785611	Particulate matter air pollution (pm2.5	423796
rs6547952	2	22045642	Particulate matter air pollution (pm2.5	423796
rs6749467	2	343517	Particulate matter air pollution (pm2.5	423796
rs6960011	7	90302199	Particulate matter air pollution (pm2.5	423796
rs718059	14	34234984	Particulate matter air pollution (pm2.5	423796
rs7264243	18	45920421	Particulate matter air pollution (pm2.5	423796
rs7265589	13	112082676	Particulate matter air pollution (pm2.5	423796
rs7270000	14	104709808	Particulate matter air pollution (pm2.5	423796
rs7280802	5	164479164	Particulate matter air pollution (pm2.5	423796
rs7514956	1	74019696	Particulate matter air pollution (pm2.5	423796
rs7573056	2	58229428	Particulate matter air pollution (pm2.5	423796
rs7720573	8	10153460	Particulate matter air pollution (pm2.5	423796
rs7725581	6	20833602	Particulate matter air pollution (pm2.5	423796
rs7776279	6	163999770	Particulate matter air pollution (pm2.5	423796
rs7823038	8	35010055	Particulate matter air pollution (pm2.5	423796
rs7853976	12	12490981	Particulate matter air pollution (pm2.5	423796
rs8012387	3	148486829	Particulate matter air pollution (pm2.5	423796
rs8614	17	27588806	Particulate matter air pollution (pm2.5	423796
rs9260734	6	29932666	Particulate matter air pollution (pm2.5	423796
rs9644485	8	138230163	Particulate matter air pollution (pm2.5	423796
rs987204	3	94242461	Particulate matter air pollution (pm2.5	423796
rs1030927	19	8779100	Average 24-hour sound level of noise p	456380
rs1175270	8	117910936	Average 24-hour sound level of noise p	456380
rs1433250	19	32674873	Average 24-hour sound level of noise p	456380

rs1443749	7	150414185	Average 24-hour sound level of noise p	456380
rs1502133	1	242381731	Average 24-hour sound level of noise p	456380
rs1759044	3	25466098	Average 24-hour sound level of noise p	456380
rs1891471	6	112596443	Average 24-hour sound level of noise p	456380
rs2714872	7	17938491	Average 24-hour sound level of noise p	456380
rs5762541	22	28666961	Average 24-hour sound level of noise p	456380
rs7147858	11	102269128	Average 24-hour sound level of noise p	456380
rs7241762	18	72116373	Average 24-hour sound level of noise p	456380
rs7492080	13	80597508	Average 24-hour sound level of noise p	456380
rs1009402	8	8646438	Nitrogen dioxide air pollution	456380
rs1011627	9	22081397	Nitrogen dioxide air pollution	456380
rs1017229	2	58201202	Nitrogen dioxide air pollution	456380
rs1075045	11	130903582	Nitrogen dioxide air pollution	456380
rs1083851	11	45780039	Nitrogen dioxide air pollution	456380
rs1085260	16	49762462	Nitrogen dioxide air pollution	456380
rs1098373	9	120416066	Nitrogen dioxide air pollution	456380
rs1104924	12	28119596	Nitrogen dioxide air pollution	456380
rs1112882	1	227090717	Nitrogen dioxide air pollution	456380
rs1120476	1	150999737	Nitrogen dioxide air pollution	456380
rs1168101	2	135135102	Nitrogen dioxide air pollution	456380
rs1180913	9	20205067	Nitrogen dioxide air pollution	456380
rs1184458	14	34241004	Nitrogen dioxide air pollution	456380
rs1208981	1	91189933	Nitrogen dioxide air pollution	456380
rs1217106	8	64567670	Nitrogen dioxide air pollution	456380
rs1220359	6	396321	Nitrogen dioxide air pollution	456380
rs1245189	17	2247982	Nitrogen dioxide air pollution	456380
rs1318845	4	153001662	Nitrogen dioxide air pollution	456380
rs1325445	8	40707884	Nitrogen dioxide air pollution	456380
rs1342194	2	6777264	Nitrogen dioxide air pollution	456380
rs1372504	5	103749428	Nitrogen dioxide air pollution	456380
rs1378675	15	25488099	Nitrogen dioxide air pollution	456380
rs1718385	18	3100049	Nitrogen dioxide air pollution	456380
rs1751318	5	53585870	Nitrogen dioxide air pollution	456380
rs2058582	4	13892169	Nitrogen dioxide air pollution	456380
rs27152	5	153734582	Nitrogen dioxide air pollution	456380
rs2838761	21	46481557	Nitrogen dioxide air pollution	456380
rs2865594	2	69152401	Nitrogen dioxide air pollution	456380
rs2870984	2	21685654	Nitrogen dioxide air pollution	456380
rs329177	5	125130444	Nitrogen dioxide air pollution	456380
rs3462373	10	129281256	Nitrogen dioxide air pollution	456380
rs3562135	7	55380566	Nitrogen dioxide air pollution	456380
rs356543	2	164851354	Nitrogen dioxide air pollution	456380

rs374039	10	104638480	Nitrogen dioxide air pollution	456380
rs391687	19	45856820	Nitrogen dioxide air pollution	456380
rs477107	13	27693827	Nitrogen dioxide air pollution	456380
rs483859	10	49669142	Nitrogen dioxide air pollution	456380
rs533745	1	34419822	Nitrogen dioxide air pollution	456380
rs556430	12	5773969	Nitrogen dioxide air pollution	456380
rs559748	7	133771130	Nitrogen dioxide air pollution	456380
rs561185	5	164465890	Nitrogen dioxide air pollution	456380
rs572917	2	213625968	Nitrogen dioxide air pollution	456380
rs588248	5	137976541	Nitrogen dioxide air pollution	456380
rs617462	12	55968949	Nitrogen dioxide air pollution	456380
rs617751	1	14054970	Nitrogen dioxide air pollution	456380
rs620620	17	6255977	Nitrogen dioxide air pollution	456380
rs621197	19	2279746	Nitrogen dioxide air pollution	456380
rs624590	7	44268127	Nitrogen dioxide air pollution	456380
rs674946	2	343517	Nitrogen dioxide air pollution	456380
rs695482	7	101207035	Nitrogen dioxide air pollution	456380
rs708917	10	2958311	Nitrogen dioxide air pollution	456380
rs714020	22	40493860	Nitrogen dioxide air pollution	456380
rs724643	7	88669096	Nitrogen dioxide air pollution	456380
rs726424	18	45920421	Nitrogen dioxide air pollution	456380
rs726558	13	112082676	Nitrogen dioxide air pollution	456380
rs727954	16	84606181	Nitrogen dioxide air pollution	456380
rs730858	7	30740079	Nitrogen dioxide air pollution	456380
rs751495	1	74019696	Nitrogen dioxide air pollution	456380
rs755913	15	83627623	Nitrogen dioxide air pollution	456380
rs761508	3	174719405	Nitrogen dioxide air pollution	456380
rs765724	4	105641593	Nitrogen dioxide air pollution	456380
rs772057	8	10153460	Nitrogen dioxide air pollution	456380
rs775861	8	143137894	Nitrogen dioxide air pollution	456380
rs776063	6	87298301	Nitrogen dioxide air pollution	456380
rs777627	6	163999770	Nitrogen dioxide air pollution	456380
rs783874	16	54206097	Nitrogen dioxide air pollution	456380
rs785397	12	12490981	Nitrogen dioxide air pollution	456380
rs791020	10	15981388	Nitrogen dioxide air pollution	456380
rs801238	3	148486829	Nitrogen dioxide air pollution	456380
rs803397	8	27818236	Nitrogen dioxide air pollution	456380
rs936852	6	27679445	Nitrogen dioxide air pollution	456380
rs108462	12	16211780	Particulate matter air pollution (pm10	423796
rs109611	9	13603291	Particulate matter air pollution (pm10	423796
rs116215	14	103577789	Particulate matter air pollution (pm10	423796
rs117122	9	133311860	Particulate matter air pollution (pm10	423796

rs117671	6	159246615	Particulate matter air pollution (pm10	423796
rs121929	6	94818099	Particulate matter air pollution (pm10	423796
rs122035	6	396321	Particulate matter air pollution (pm10	423796
rs125711	10	127771554	Particulate matter air pollution (pm10	423796
rs145045	8	342124	Particulate matter air pollution (pm10	423796
rs149130	11	83722884	Particulate matter air pollution (pm10	423796
rs149752	2	33098355	Particulate matter air pollution (pm10	423796
rs150993	10	64836267	Particulate matter air pollution (pm10	423796
rs218514	18	1469513	Particulate matter air pollution (pm10	423796
rs281223	13	51264767	Particulate matter air pollution (pm10	423796
rs347764	15	57656713	Particulate matter air pollution (pm10	423796
rs356652	5	51178554	Particulate matter air pollution (pm10	423796
rs481513	20	286487	Particulate matter air pollution (pm10	423796
rs570482	7	151623218	Particulate matter air pollution (pm10	423796
rs614471	5	101445992	Particulate matter air pollution (pm10	423796
rs621197	19	2279746	Particulate matter air pollution (pm10	423796
rs728195	5	164643843	Particulate matter air pollution (pm10	423796
rs732014	8	11512667	Particulate matter air pollution (pm10	423796
rs747565	1	115028382	Particulate matter air pollution (pm10	423796
rs750517	18	75220892	Particulate matter air pollution (pm10	423796
rs790372	13	50507971	Particulate matter air pollution (pm10	423796
rs802301	14	48380718	Particulate matter air pollution (pm10	423796
rs803434	15	68925781	Particulate matter air pollution (pm10	423796
rs109611	9	13603291	Particulate matter air pollution (pm2.5	423796
rs116925	8	93266536	Particulate matter air pollution (pm2.5	423796
rs117878	22	18475815	Particulate matter air pollution (pm2.5	423796
rs118289	7	75245569	Particulate matter air pollution (pm2.5	423796
rs118558	15	78012618	Particulate matter air pollution (pm2.5	423796
rs121330	1	91214714	Particulate matter air pollution (pm2.5	423796
rs121710	8	64567670	Particulate matter air pollution (pm2.5	423796
rs122035	6	396321	Particulate matter air pollution (pm2.5	423796
rs131884	4	153001662	Particulate matter air pollution (pm2.5	423796
rs137250	5	103749428	Particulate matter air pollution (pm2.5	423796
rs153737	9	22099568	Particulate matter air pollution (pm2.5	423796
rs169577	16	82610053	Particulate matter air pollution (pm2.5	423796
rs171039	14	76507299	Particulate matter air pollution (pm2.5	423796
rs171838	18	3100049	Particulate matter air pollution (pm2.5	423796
rs176578	7	17757841	Particulate matter air pollution (pm2.5	423796
rs214153	7	9253413	Particulate matter air pollution (pm2.5	423796
rs229215	16	49765133	Particulate matter air pollution (pm2.5	423796
rs27152	5	153734582	Particulate matter air pollution (pm2.5	423796
rs357035	11	61439682	Particulate matter air pollution (pm2.5	423796

rs4854523	2	69051853	Particulate matter air pollution (pm2.5	423796
rs487230	1	55541174	Particulate matter air pollution (pm2.5	423796
rs5606867	6	50597378	Particulate matter air pollution (pm2.5	423796
rs5882485	5	137976541	Particulate matter air pollution (pm2.5	423796
rs6134106	20	11007208	Particulate matter air pollution (pm2.5	423796
rs6211970	19	2279746	Particulate matter air pollution (pm2.5	423796
rs6218046	2	200815608	Particulate matter air pollution (pm2.5	423796
rs6236867	5	80861930	Particulate matter air pollution (pm2.5	423796
rs6248466	7	104984561	Particulate matter air pollution (pm2.5	423796
rs6432776	2	164785611	Particulate matter air pollution (pm2.5	423796
rs6547952	2	22045642	Particulate matter air pollution (pm2.5	423796
rs6749467	2	343517	Particulate matter air pollution (pm2.5	423796
rs6960011	7	90302199	Particulate matter air pollution (pm2.5	423796
rs718059	14	34234984	Particulate matter air pollution (pm2.5	423796
rs7264243	18	45920421	Particulate matter air pollution (pm2.5	423796
rs7265589	13	112082676	Particulate matter air pollution (pm2.5	423796
rs7270000	14	104709808	Particulate matter air pollution (pm2.5	423796
rs7280802	5	164479164	Particulate matter air pollution (pm2.5	423796
rs7514956	1	74019696	Particulate matter air pollution (pm2.5	423796
rs7573056	2	58229428	Particulate matter air pollution (pm2.5	423796
rs7720573	8	10153460	Particulate matter air pollution (pm2.5	423796
rs7725581	6	20833602	Particulate matter air pollution (pm2.5	423796
rs7776279	6	163999770	Particulate matter air pollution (pm2.5	423796
rs7823038	8	35010055	Particulate matter air pollution (pm2.5	423796
rs7853976	12	12490981	Particulate matter air pollution (pm2.5	423796
rs8012387	3	148486829	Particulate matter air pollution (pm2.5	423796
rs8614	17	27588806	Particulate matter air pollution (pm2.5	423796
rs9260734	6	29932666	Particulate matter air pollution (pm2.5	423796
rs9644485	8	138230163	Particulate matter air pollution (pm2.5	423796
rs987204	3	94242461	Particulate matter air pollution (pm2.5	423796
rs1030927	19	8779100	Average 24-hour sound level of noise p	456380
rs1175270	8	117910936	Average 24-hour sound level of noise p	456380
rs1433250	19	32674873	Average 24-hour sound level of noise p	456380
rs1443749	7	150414185	Average 24-hour sound level of noise p	456380
rs1502133	1	242381731	Average 24-hour sound level of noise p	456380
rs1759044	3	25466098	Average 24-hour sound level of noise p	456380
rs1891471	6	112596443	Average 24-hour sound level of noise p	456380
rs2714872	7	17938491	Average 24-hour sound level of noise p	456380
rs5762541	22	28666961	Average 24-hour sound level of noise p	456380
rs7147858	11	102269128	Average 24-hour sound level of noise p	456380
rs7241762	18	72116373	Average 24-hour sound level of noise p	456380
rs7492080	13	80597508	Average 24-hour sound level of noise p	456380

rs1009402	8	8646438	Nitrogen dioxide air pollution	456380
rs1011627	9	22081397	Nitrogen dioxide air pollution	456380
rs1017229	2	58201202	Nitrogen dioxide air pollution	456380
rs1075045	11	130903582	Nitrogen dioxide air pollution	456380
rs1083851	11	45780039	Nitrogen dioxide air pollution	456380
rs1085260	16	49762462	Nitrogen dioxide air pollution	456380
rs1098373	9	120416066	Nitrogen dioxide air pollution	456380
rs1104924	12	28119596	Nitrogen dioxide air pollution	456380
rs1112882	1	227090717	Nitrogen dioxide air pollution	456380
rs1120476	1	150999737	Nitrogen dioxide air pollution	456380
rs1168101	2	135135102	Nitrogen dioxide air pollution	456380
rs1180913	9	20205067	Nitrogen dioxide air pollution	456380
rs1184458	14	34241004	Nitrogen dioxide air pollution	456380
rs1208981	1	91189933	Nitrogen dioxide air pollution	456380
rs1217106	8	64567670	Nitrogen dioxide air pollution	456380
rs1220359	6	396321	Nitrogen dioxide air pollution	456380
rs1245189	17	2247982	Nitrogen dioxide air pollution	456380
rs1318845	4	153001662	Nitrogen dioxide air pollution	456380
rs1325445	8	40707884	Nitrogen dioxide air pollution	456380
rs1342194	2	6777264	Nitrogen dioxide air pollution	456380
rs1372504	5	103749428	Nitrogen dioxide air pollution	456380
rs1378675	15	25488099	Nitrogen dioxide air pollution	456380
rs1718385	18	3100049	Nitrogen dioxide air pollution	456380
rs1751318	5	53585870	Nitrogen dioxide air pollution	456380
rs2058582	4	13892169	Nitrogen dioxide air pollution	456380
rs27152	5	153734582	Nitrogen dioxide air pollution	456380
rs2838761	21	46481557	Nitrogen dioxide air pollution	456380
rs2865594	2	69152401	Nitrogen dioxide air pollution	456380
rs2870984	2	21685654	Nitrogen dioxide air pollution	456380
rs329177	5	125130444	Nitrogen dioxide air pollution	456380
rs3462373	10	129281256	Nitrogen dioxide air pollution	456380
rs3562135	7	55380566	Nitrogen dioxide air pollution	456380
rs356543	2	164851354	Nitrogen dioxide air pollution	456380
rs3740390	10	104638480	Nitrogen dioxide air pollution	456380
rs3916875	19	45856820	Nitrogen dioxide air pollution	456380
rs4771073	13	27693827	Nitrogen dioxide air pollution	456380
rs4838594	10	49669142	Nitrogen dioxide air pollution	456380
rs533745	1	34419822	Nitrogen dioxide air pollution	456380
rs5564300	12	5773969	Nitrogen dioxide air pollution	456380
rs5597480	7	133771130	Nitrogen dioxide air pollution	456380
rs5611854	5	164465890	Nitrogen dioxide air pollution	456380
rs5729175	2	213625968	Nitrogen dioxide air pollution	456380

rs588248	5	137976541	Nitrogen dioxide air pollution	456380
rs617462	12	55968949	Nitrogen dioxide air pollution	456380
rs617751	1	14054970	Nitrogen dioxide air pollution	456380
rs620620	17	6255977	Nitrogen dioxide air pollution	456380
rs621197	19	2279746	Nitrogen dioxide air pollution	456380
rs624590	7	44268127	Nitrogen dioxide air pollution	456380
rs674946	2	343517	Nitrogen dioxide air pollution	456380
rs695482	7	101207035	Nitrogen dioxide air pollution	456380
rs708917	10	2958311	Nitrogen dioxide air pollution	456380
rs714020	22	40493860	Nitrogen dioxide air pollution	456380
rs724643	7	88669096	Nitrogen dioxide air pollution	456380
rs726424	18	45920421	Nitrogen dioxide air pollution	456380
rs726558	13	112082676	Nitrogen dioxide air pollution	456380
rs727954	16	84606181	Nitrogen dioxide air pollution	456380
rs730858	7	30740079	Nitrogen dioxide air pollution	456380
rs751495	1	74019696	Nitrogen dioxide air pollution	456380
rs755913	15	83627623	Nitrogen dioxide air pollution	456380
rs761508	3	174719405	Nitrogen dioxide air pollution	456380
rs765724	4	105641593	Nitrogen dioxide air pollution	456380
rs772057	8	10153460	Nitrogen dioxide air pollution	456380
rs775861	8	143137894	Nitrogen dioxide air pollution	456380
rs776063	6	87298301	Nitrogen dioxide air pollution	456380
rs777627	6	163999770	Nitrogen dioxide air pollution	456380
rs783874	16	54206097	Nitrogen dioxide air pollution	456380
rs785397	12	12490981	Nitrogen dioxide air pollution	456380
rs791020	10	15981388	Nitrogen dioxide air pollution	456380
rs801238	3	148486829	Nitrogen dioxide air pollution	456380
rs803397	8	27818236	Nitrogen dioxide air pollution	456380
rs936852	6	27679445	Nitrogen dioxide air pollution	456380
rs109611	9	13603291	Particulate matter air pollution (pm2.5	423796
rs116925	8	93266536	Particulate matter air pollution (pm2.5	423796
rs117878	22	18475815	Particulate matter air pollution (pm2.5	423796
rs118289	7	75245569	Particulate matter air pollution (pm2.5	423796
rs118558	15	78012618	Particulate matter air pollution (pm2.5	423796
rs121330	1	91214714	Particulate matter air pollution (pm2.5	423796
rs121710	8	64567670	Particulate matter air pollution (pm2.5	423796
rs122035	6	396321	Particulate matter air pollution (pm2.5	423796
rs131884	4	153001662	Particulate matter air pollution (pm2.5	423796
rs137250	5	103749428	Particulate matter air pollution (pm2.5	423796
rs153737	9	22099568	Particulate matter air pollution (pm2.5	423796
rs169577	16	82610053	Particulate matter air pollution (pm2.5	423796
rs171039	14	76507299	Particulate matter air pollution (pm2.5	423796

rs171838!	18	3100049	Particulate matter air pollution (pm2.5	423796
rs176578!	7	17757841	Particulate matter air pollution (pm2.5	423796
rs214153:	7	9253413	Particulate matter air pollution (pm2.5	423796
rs229215(	16	49765133	Particulate matter air pollution (pm2.5	423796
rs27152	5	153734582	Particulate matter air pollution (pm2.5	423796
rs357035:	11	61439682	Particulate matter air pollution (pm2.5	423796
rs485452:	2	69051853	Particulate matter air pollution (pm2.5	423796
rs487230	1	55541174	Particulate matter air pollution (pm2.5	423796
rs560686:	6	50597378	Particulate matter air pollution (pm2.5	423796
rs588248!	5	137976541	Particulate matter air pollution (pm2.5	423796
rs613410(	20	11007208	Particulate matter air pollution (pm2.5	423796
rs621197(	19	2279746	Particulate matter air pollution (pm2.5	423796
rs621804(	2	200815608	Particulate matter air pollution (pm2.5	423796
rs623686:	5	80861930	Particulate matter air pollution (pm2.5	423796
rs624846(	7	104984561	Particulate matter air pollution (pm2.5	423796
rs643277(	2	164785611	Particulate matter air pollution (pm2.5	423796
rs654795:	2	22045642	Particulate matter air pollution (pm2.5	423796
rs674946:	2	343517	Particulate matter air pollution (pm2.5	423796
rs696001:	7	90302199	Particulate matter air pollution (pm2.5	423796
rs718059	14	34234984	Particulate matter air pollution (pm2.5	423796
rs726424:	18	45920421	Particulate matter air pollution (pm2.5	423796
rs726558!	13	112082676	Particulate matter air pollution (pm2.5	423796
rs727000(	14	104709808	Particulate matter air pollution (pm2.5	423796
rs728080:	5	164479164	Particulate matter air pollution (pm2.5	423796
rs751495(	1	74019696	Particulate matter air pollution (pm2.5	423796
rs757305(	2	58229428	Particulate matter air pollution (pm2.5	423796
rs772057:	8	10153460	Particulate matter air pollution (pm2.5	423796
rs772558:	6	20833602	Particulate matter air pollution (pm2.5	423796
rs777627!	6	163999770	Particulate matter air pollution (pm2.5	423796
rs782303!	8	35010055	Particulate matter air pollution (pm2.5	423796
rs785397(	12	12490981	Particulate matter air pollution (pm2.5	423796
rs801238:	3	148486829	Particulate matter air pollution (pm2.5	423796
rs8614	17	27588806	Particulate matter air pollution (pm2.5	423796
rs926073‹	6	29932666	Particulate matter air pollution (pm2.5	423796
rs964448!	8	138230163	Particulate matter air pollution (pm2.5	423796
rs987204	3	94242461	Particulate matter air pollution (pm2.5	423796
rs108462:	12	16211780	Particulate matter air pollution (pm10)	423796
rs109611(	9	13603291	Particulate matter air pollution (pm10)	423796
rs116215:	14	103577789	Particulate matter air pollution (pm10)	423796
rs117122:	9	133311860	Particulate matter air pollution (pm10)	423796
rs117671:	6	159246615	Particulate matter air pollution (pm10)	423796
rs121929!	6	94818099	Particulate matter air pollution (pm10)	423796

rs122035	6	396321	Particulate matter air pollution (pm10	423796
rs125711	10	127771554	Particulate matter air pollution (pm10	423796
rs145045	8	342124	Particulate matter air pollution (pm10	423796
rs149130	11	83722884	Particulate matter air pollution (pm10	423796
rs149752	2	33098355	Particulate matter air pollution (pm10	423796
rs150993	10	64836267	Particulate matter air pollution (pm10	423796
rs218514	18	1469513	Particulate matter air pollution (pm10	423796
rs281223	13	51264767	Particulate matter air pollution (pm10	423796
rs347764	15	57656713	Particulate matter air pollution (pm10	423796
rs356652	5	51178554	Particulate matter air pollution (pm10	423796
rs481513	20	286487	Particulate matter air pollution (pm10	423796
rs570482	7	151623218	Particulate matter air pollution (pm10	423796
rs614471	5	101445992	Particulate matter air pollution (pm10	423796
rs621197	19	2279746	Particulate matter air pollution (pm10	423796
rs728195	5	164643843	Particulate matter air pollution (pm10	423796
rs732014	8	11512667	Particulate matter air pollution (pm10	423796
rs747565	1	115028382	Particulate matter air pollution (pm10	423796
rs750517	18	75220892	Particulate matter air pollution (pm10	423796
rs790372	13	50507971	Particulate matter air pollution (pm10	423796
rs802301	14	48380718	Particulate matter air pollution (pm10	423796
rs803434	15	68925781	Particulate matter air pollution (pm10	423796
rs103092	19	8779100	Average 24-hour sound level of noise	456380
rs117527	8	117910936	Average 24-hour sound level of noise	456380
rs143325	19	32674873	Average 24-hour sound level of noise	456380
rs144374	7	150414185	Average 24-hour sound level of noise	456380
rs150213	1	242381731	Average 24-hour sound level of noise	456380
rs175904	3	25466098	Average 24-hour sound level of noise	456380
rs189147	6	112596443	Average 24-hour sound level of noise	456380
rs271487	7	17938491	Average 24-hour sound level of noise	456380
rs576254	22	28666961	Average 24-hour sound level of noise	456380
rs714785	11	102269128	Average 24-hour sound level of noise	456380
rs724176	18	72116373	Average 24-hour sound level of noise	456380
rs749208	13	80597508	Average 24-hour sound level of noise	456380
rs100940	8	8646438	Nitrogen dioxide air pollution	456380
rs101162	9	22081397	Nitrogen dioxide air pollution	456380
rs101722	2	58201202	Nitrogen dioxide air pollution	456380
rs107504	11	130903582	Nitrogen dioxide air pollution	456380
rs108385	11	45780039	Nitrogen dioxide air pollution	456380
rs108526	16	49762462	Nitrogen dioxide air pollution	456380
rs109837	9	120416066	Nitrogen dioxide air pollution	456380
rs110492	12	28119596	Nitrogen dioxide air pollution	456380
rs111288	1	227090717	Nitrogen dioxide air pollution	456380

rs112047	1	150999737	Nitrogen dioxide air pollution	456380
rs116810	2	135135102	Nitrogen dioxide air pollution	456380
rs118091	9	20205067	Nitrogen dioxide air pollution	456380
rs118445	14	34241004	Nitrogen dioxide air pollution	456380
rs120898	1	91189933	Nitrogen dioxide air pollution	456380
rs121710	8	64567670	Nitrogen dioxide air pollution	456380
rs122035	6	396321	Nitrogen dioxide air pollution	456380
rs124518	17	2247982	Nitrogen dioxide air pollution	456380
rs131884	4	153001662	Nitrogen dioxide air pollution	456380
rs132544	8	40707884	Nitrogen dioxide air pollution	456380
rs134219	2	6777264	Nitrogen dioxide air pollution	456380
rs137250	5	103749428	Nitrogen dioxide air pollution	456380
rs137867	15	25488099	Nitrogen dioxide air pollution	456380
rs171838	18	3100049	Nitrogen dioxide air pollution	456380
rs175131	5	53585870	Nitrogen dioxide air pollution	456380
rs205858	4	13892169	Nitrogen dioxide air pollution	456380
rs27152	5	153734582	Nitrogen dioxide air pollution	456380
rs283876	21	46481557	Nitrogen dioxide air pollution	456380
rs286559	2	69152401	Nitrogen dioxide air pollution	456380
rs287098	2	21685654	Nitrogen dioxide air pollution	456380
rs329177	5	125130444	Nitrogen dioxide air pollution	456380
rs346237	10	129281256	Nitrogen dioxide air pollution	456380
rs356213	7	55380566	Nitrogen dioxide air pollution	456380
rs356543	2	164851354	Nitrogen dioxide air pollution	456380
rs374039	10	104638480	Nitrogen dioxide air pollution	456380
rs391687	19	45856820	Nitrogen dioxide air pollution	456380
rs477107	13	27693827	Nitrogen dioxide air pollution	456380
rs483859	10	49669142	Nitrogen dioxide air pollution	456380
rs533745	1	34419822	Nitrogen dioxide air pollution	456380
rs556430	12	5773969	Nitrogen dioxide air pollution	456380
rs559748	7	133771130	Nitrogen dioxide air pollution	456380
rs561185	5	164465890	Nitrogen dioxide air pollution	456380
rs572917	2	213625968	Nitrogen dioxide air pollution	456380
rs588248	5	137976541	Nitrogen dioxide air pollution	456380
rs617462	12	55968949	Nitrogen dioxide air pollution	456380
rs617751	1	14054970	Nitrogen dioxide air pollution	456380
rs620620	17	6255977	Nitrogen dioxide air pollution	456380
rs621197	19	2279746	Nitrogen dioxide air pollution	456380
rs624590	7	44268127	Nitrogen dioxide air pollution	456380
rs674946	2	343517	Nitrogen dioxide air pollution	456380
rs695482	7	101207035	Nitrogen dioxide air pollution	456380
rs708917	10	2958311	Nitrogen dioxide air pollution	456380

rs714020	22	40493860	Nitrogen dioxide air pollution	456380
rs724643	7	88669096	Nitrogen dioxide air pollution	456380
rs726424	18	45920421	Nitrogen dioxide air pollution	456380
rs726558	13	112082676	Nitrogen dioxide air pollution	456380
rs727954	16	84606181	Nitrogen dioxide air pollution	456380
rs730858	7	30740079	Nitrogen dioxide air pollution	456380
rs751495	1	74019696	Nitrogen dioxide air pollution	456380
rs755913	15	83627623	Nitrogen dioxide air pollution	456380
rs761508	3	174719405	Nitrogen dioxide air pollution	456380
rs765724	4	105641593	Nitrogen dioxide air pollution	456380
rs772057	8	10153460	Nitrogen dioxide air pollution	456380
rs775861	8	143137894	Nitrogen dioxide air pollution	456380
rs776063	6	87298301	Nitrogen dioxide air pollution	456380
rs777627	6	163999770	Nitrogen dioxide air pollution	456380
rs783874	16	54206097	Nitrogen dioxide air pollution	456380
rs785397	12	12490981	Nitrogen dioxide air pollution	456380
rs791020	10	15981388	Nitrogen dioxide air pollution	456380
rs801238	3	148486829	Nitrogen dioxide air pollution	456380
rs803397	8	27818236	Nitrogen dioxide air pollution	456380
rs936852	6	27679445	Nitrogen dioxide air pollution	456380
rs109611	9	13603291	Particulate matter air pollution (pm2.5	423796
rs116925	8	93266536	Particulate matter air pollution (pm2.5	423796
rs117878	22	18475815	Particulate matter air pollution (pm2.5	423796
rs118289	7	75245569	Particulate matter air pollution (pm2.5	423796
rs118558	15	78012618	Particulate matter air pollution (pm2.5	423796
rs121330	1	91214714	Particulate matter air pollution (pm2.5	423796
rs121710	8	64567670	Particulate matter air pollution (pm2.5	423796
rs122035	6	396321	Particulate matter air pollution (pm2.5	423796
rs131884	4	153001662	Particulate matter air pollution (pm2.5	423796
rs137250	5	103749428	Particulate matter air pollution (pm2.5	423796
rs153737	9	22099568	Particulate matter air pollution (pm2.5	423796
rs169577	16	82610053	Particulate matter air pollution (pm2.5	423796
rs171039	14	76507299	Particulate matter air pollution (pm2.5	423796
rs171838	18	3100049	Particulate matter air pollution (pm2.5	423796
rs176578	7	17757841	Particulate matter air pollution (pm2.5	423796
rs214153	7	9253413	Particulate matter air pollution (pm2.5	423796
rs229215	16	49765133	Particulate matter air pollution (pm2.5	423796
rs27152	5	153734582	Particulate matter air pollution (pm2.5	423796
rs357035	11	61439682	Particulate matter air pollution (pm2.5	423796
rs485452	2	69051853	Particulate matter air pollution (pm2.5	423796
rs487230	1	55541174	Particulate matter air pollution (pm2.5	423796
rs560686	6	50597378	Particulate matter air pollution (pm2.5	423796

rs588248[illegible]	5	137976541	Particulate matter air pollution (pm2.5	423796
rs613410[illegible]	20	11007208	Particulate matter air pollution (pm2.5	423796
rs621197[illegible]	19	2279746	Particulate matter air pollution (pm2.5	423796
rs621804[illegible]	2	200815608	Particulate matter air pollution (pm2.5	423796
rs623686[illegible]	5	80861930	Particulate matter air pollution (pm2.5	423796
rs624846[illegible]	7	104984561	Particulate matter air pollution (pm2.5	423796
rs643277[illegible]	2	164785611	Particulate matter air pollution (pm2.5	423796
rs654795[illegible]	2	22045642	Particulate matter air pollution (pm2.5	423796
rs674946[illegible]	2	343517	Particulate matter air pollution (pm2.5	423796
rs696001[illegible]	7	90302199	Particulate matter air pollution (pm2.5	423796
rs718059	14	34234984	Particulate matter air pollution (pm2.5	423796
rs726424[illegible]	18	45920421	Particulate matter air pollution (pm2.5	423796
rs726558[illegible]	13	112082676	Particulate matter air pollution (pm2.5	423796
rs727000[illegible]	14	104709808	Particulate matter air pollution (pm2.5	423796
rs728080[illegible]	5	164479164	Particulate matter air pollution (pm2.5	423796
rs751495[illegible]	1	74019696	Particulate matter air pollution (pm2.5	423796
rs757305[illegible]	2	58229428	Particulate matter air pollution (pm2.5	423796
rs772057[illegible]	8	10153460	Particulate matter air pollution (pm2.5	423796
rs772558[illegible]	6	20833602	Particulate matter air pollution (pm2.5	423796
rs777627[illegible]	6	163999770	Particulate matter air pollution (pm2.5	423796
rs782303[illegible]	8	35010055	Particulate matter air pollution (pm2.5	423796
rs785397[illegible]	12	12490981	Particulate matter air pollution (pm2.5	423796
rs801238[illegible]	3	148486829	Particulate matter air pollution (pm2.5	423796
rs8614	17	27588806	Particulate matter air pollution (pm2.5	423796
rs926073[illegible]	6	29932666	Particulate matter air pollution (pm2.5	423796
rs964448[illegible]	8	138230163	Particulate matter air pollution (pm2.5	423796
rs987204	3	94242461	Particulate matter air pollution (pm2.5	423796
rs108462[illegible]	12	16211780	Particulate matter air pollution (pm10)	423796
rs109611[illegible]	9	13603291	Particulate matter air pollution (pm10)	423796
rs116215[illegible]	14	103577789	Particulate matter air pollution (pm10)	423796
rs117122[illegible]	9	133311860	Particulate matter air pollution (pm10)	423796
rs117671[illegible]	6	159246615	Particulate matter air pollution (pm10)	423796
rs121929[illegible]	6	94818099	Particulate matter air pollution (pm10)	423796
rs122035[illegible]	6	396321	Particulate matter air pollution (pm10)	423796
rs125711[illegible]	10	127771554	Particulate matter air pollution (pm10)	423796
rs145045[illegible]	8	342124	Particulate matter air pollution (pm10)	423796
rs149130[illegible]	11	83722884	Particulate matter air pollution (pm10)	423796
rs149752[illegible]	2	33098355	Particulate matter air pollution (pm10)	423796
rs150993[illegible]	10	64836267	Particulate matter air pollution (pm10)	423796
rs218514	18	1469513	Particulate matter air pollution (pm10)	423796
rs281223[illegible]	13	51264767	Particulate matter air pollution (pm10)	423796
rs347764[illegible]	15	57656713	Particulate matter air pollution (pm10)	423796

rs356652(	5	51178554	Particulate matter air pollution (pm10)	423796
rs481513{	20	286487	Particulate matter air pollution (pm10)	423796
rs570482(	7	151623218	Particulate matter air pollution (pm10)	423796
rs614471{	5	101445992	Particulate matter air pollution (pm10)	423796
rs621197(	19	2279746	Particulate matter air pollution (pm10)	423796
rs728195(	5	164643843	Particulate matter air pollution (pm10)	423796
rs732014	8	11512667	Particulate matter air pollution (pm10)	423796
rs747565	1	115028382	Particulate matter air pollution (pm10)	423796
rs750517	18	75220892	Particulate matter air pollution (pm10)	423796
rs790372	13	50507971	Particulate matter air pollution (pm10)	423796
rs802301	14	48380718	Particulate matter air pollution (pm10)	423796
rs803434	15	68925781	Particulate matter air pollution (pm10)	423796
rs103092	19	8779100	Average 24-hour sound level of noise p	456380
rs117527(	8	117910936	Average 24-hour sound level of noise p	456380
rs143325(	19	32674873	Average 24-hour sound level of noise p	456380
rs144374	7	150414185	Average 24-hour sound level of noise p	456380
rs150213	1	242381731	Average 24-hour sound level of noise p	456380
rs175904	3	25466098	Average 24-hour sound level of noise p	456380
rs189147	6	112596443	Average 24-hour sound level of noise p	456380
rs271487	7	17938491	Average 24-hour sound level of noise p	456380
rs576254	22	28666961	Average 24-hour sound level of noise p	456380
rs714785{	11	102269128	Average 24-hour sound level of noise p	456380
rs724176	18	72116373	Average 24-hour sound level of noise p	456380
rs749208(	13	80597508	Average 24-hour sound level of noise p	456380
rs109611(	9	13603291	Particulate matter air pollution (pm2.5	423796
rs116925	8	93266536	Particulate matter air pollution (pm2.5	423796
rs117878	22	18475815	Particulate matter air pollution (pm2.5	423796
rs118289	7	75245569	Particulate matter air pollution (pm2.5	423796
rs118558	15	78012618	Particulate matter air pollution (pm2.5	423796
rs121330(	1	91214714	Particulate matter air pollution (pm2.5	423796
rs121710(	8	64567670	Particulate matter air pollution (pm2.5	423796
rs122035	6	396321	Particulate matter air pollution (pm2.5	423796
rs131884	4	153001662	Particulate matter air pollution (pm2.5	423796
rs137250	5	103749428	Particulate matter air pollution (pm2.5	423796
rs153737	9	22099568	Particulate matter air pollution (pm2.5	423796
rs169577	16	82610053	Particulate matter air pollution (pm2.5	423796
rs171039	14	76507299	Particulate matter air pollution (pm2.5	423796
rs171838	18	3100049	Particulate matter air pollution (pm2.5	423796
rs176578{	7	17757841	Particulate matter air pollution (pm2.5	423796
rs214153	7	9253413	Particulate matter air pollution (pm2.5	423796
rs229215(	16	49765133	Particulate matter air pollution (pm2.5	423796
rs27152	5	153734582	Particulate matter air pollution (pm2.5	423796

rs357035	11	61439682	Particulate matter air pollution (pm2.5	423796
rs485452	2	69051853	Particulate matter air pollution (pm2.5	423796
rs487230	1	55541174	Particulate matter air pollution (pm2.5	423796
rs560686	6	50597378	Particulate matter air pollution (pm2.5	423796
rs588248	5	137976541	Particulate matter air pollution (pm2.5	423796
rs613410	20	11007208	Particulate matter air pollution (pm2.5	423796
rs621197	19	2279746	Particulate matter air pollution (pm2.5	423796
rs621804	2	200815608	Particulate matter air pollution (pm2.5	423796
rs623686	5	80861930	Particulate matter air pollution (pm2.5	423796
rs624846	7	104984561	Particulate matter air pollution (pm2.5	423796
rs643277	2	164785611	Particulate matter air pollution (pm2.5	423796
rs654795	2	22045642	Particulate matter air pollution (pm2.5	423796
rs674946	2	343517	Particulate matter air pollution (pm2.5	423796
rs696001	7	90302199	Particulate matter air pollution (pm2.5	423796
rs718059	14	34234984	Particulate matter air pollution (pm2.5	423796
rs726424	18	45920421	Particulate matter air pollution (pm2.5	423796
rs726558	13	112082676	Particulate matter air pollution (pm2.5	423796
rs727000	14	104709808	Particulate matter air pollution (pm2.5	423796
rs728080	5	164479164	Particulate matter air pollution (pm2.5	423796
rs751495	1	74019696	Particulate matter air pollution (pm2.5	423796
rs757305	2	58229428	Particulate matter air pollution (pm2.5	423796
rs772057	8	10153460	Particulate matter air pollution (pm2.5	423796
rs772558	6	20833602	Particulate matter air pollution (pm2.5	423796
rs777627	6	163999770	Particulate matter air pollution (pm2.5	423796
rs782303	8	35010055	Particulate matter air pollution (pm2.5	423796
rs785397	12	12490981	Particulate matter air pollution (pm2.5	423796
rs801238	3	148486829	Particulate matter air pollution (pm2.5	423796
rs8614	17	27588806	Particulate matter air pollution (pm2.5	423796
rs926073	6	29932666	Particulate matter air pollution (pm2.5	423796
rs964448	8	138230163	Particulate matter air pollution (pm2.5	423796
rs987204	3	94242461	Particulate matter air pollution (pm2.5	423796
rs100940	8	8646438	Nitrogen dioxide air pollution	456380
rs101162	9	22081397	Nitrogen dioxide air pollution	456380
rs101722	2	58201202	Nitrogen dioxide air pollution	456380
rs107504	11	130903582	Nitrogen dioxide air pollution	456380
rs108385	11	45780039	Nitrogen dioxide air pollution	456380
rs108526	16	49762462	Nitrogen dioxide air pollution	456380
rs109837	9	120416066	Nitrogen dioxide air pollution	456380
rs110492	12	28119596	Nitrogen dioxide air pollution	456380
rs111288	1	227090717	Nitrogen dioxide air pollution	456380
rs112047	1	150999737	Nitrogen dioxide air pollution	456380
rs116810	2	135135102	Nitrogen dioxide air pollution	456380

rs1180913	9	20205067	Nitrogen dioxide air pollution	456380
rs1184458	14	34241004	Nitrogen dioxide air pollution	456380
rs1208981	1	91189933	Nitrogen dioxide air pollution	456380
rs1217106	8	64567670	Nitrogen dioxide air pollution	456380
rs1220359	6	396321	Nitrogen dioxide air pollution	456380
rs1245189	17	2247982	Nitrogen dioxide air pollution	456380
rs1318845	4	153001662	Nitrogen dioxide air pollution	456380
rs1325445	8	40707884	Nitrogen dioxide air pollution	456380
rs1342194	2	6777264	Nitrogen dioxide air pollution	456380
rs1372504	5	103749428	Nitrogen dioxide air pollution	456380
rs1378675	15	25488099	Nitrogen dioxide air pollution	456380
rs1718385	18	3100049	Nitrogen dioxide air pollution	456380
rs1751318	5	53585870	Nitrogen dioxide air pollution	456380
rs2058582	4	13892169	Nitrogen dioxide air pollution	456380
rs27152	5	153734582	Nitrogen dioxide air pollution	456380
rs2838761	21	46481557	Nitrogen dioxide air pollution	456380
rs2865594	2	69152401	Nitrogen dioxide air pollution	456380
rs2870984	2	21685654	Nitrogen dioxide air pollution	456380
rs329177	5	125130444	Nitrogen dioxide air pollution	456380
rs3462373	10	129281256	Nitrogen dioxide air pollution	456380
rs3562135	7	55380566	Nitrogen dioxide air pollution	456380
rs356543	2	164851354	Nitrogen dioxide air pollution	456380
rs3740390	10	104638480	Nitrogen dioxide air pollution	456380
rs3916875	19	45856820	Nitrogen dioxide air pollution	456380
rs4771073	13	27693827	Nitrogen dioxide air pollution	456380
rs4838594	10	49669142	Nitrogen dioxide air pollution	456380
rs533745	1	34419822	Nitrogen dioxide air pollution	456380
rs5564300	12	5773969	Nitrogen dioxide air pollution	456380
rs5597480	7	133771130	Nitrogen dioxide air pollution	456380
rs5611854	5	164465890	Nitrogen dioxide air pollution	456380
rs5729175	2	213625968	Nitrogen dioxide air pollution	456380
rs5882485	5	137976541	Nitrogen dioxide air pollution	456380
rs6174628	12	55968949	Nitrogen dioxide air pollution	456380
rs6177510	1	14054970	Nitrogen dioxide air pollution	456380
rs6206203	17	6255977	Nitrogen dioxide air pollution	456380
rs6211970	19	2279746	Nitrogen dioxide air pollution	456380
rs6245909	7	44268127	Nitrogen dioxide air pollution	456380
rs6749467	2	343517	Nitrogen dioxide air pollution	456380
rs6954825	7	101207035	Nitrogen dioxide air pollution	456380
rs7089175	10	2958311	Nitrogen dioxide air pollution	456380
rs714020	22	40493860	Nitrogen dioxide air pollution	456380
rs724643	7	88669096	Nitrogen dioxide air pollution	456380

rs726424	18	45920421	Nitrogen dioxide air pollution	456380
rs726558	13	112082676	Nitrogen dioxide air pollution	456380
rs727954	16	84606181	Nitrogen dioxide air pollution	456380
rs730858	7	30740079	Nitrogen dioxide air pollution	456380
rs751495	1	74019696	Nitrogen dioxide air pollution	456380
rs755913	15	83627623	Nitrogen dioxide air pollution	456380
rs761508	3	174719405	Nitrogen dioxide air pollution	456380
rs765724	4	105641593	Nitrogen dioxide air pollution	456380
rs772057	8	10153460	Nitrogen dioxide air pollution	456380
rs775861	8	143137894	Nitrogen dioxide air pollution	456380
rs776063	6	87298301	Nitrogen dioxide air pollution	456380
rs777627	6	163999770	Nitrogen dioxide air pollution	456380
rs783874	16	54206097	Nitrogen dioxide air pollution	456380
rs785397	12	12490981	Nitrogen dioxide air pollution	456380
rs791020	10	15981388	Nitrogen dioxide air pollution	456380
rs801238	3	148486829	Nitrogen dioxide air pollution	456380
rs803397	8	27818236	Nitrogen dioxide air pollution	456380
rs936852	6	27679445	Nitrogen dioxide air pollution	456380
rs108462	12	16211780	Particulate matter air pollution (pm10)	423796
rs109611	9	13603291	Particulate matter air pollution (pm10)	423796
rs116215	14	103577789	Particulate matter air pollution (pm10)	423796
rs117122	9	133311860	Particulate matter air pollution (pm10)	423796
rs117671	6	159246615	Particulate matter air pollution (pm10)	423796
rs121929	6	94818099	Particulate matter air pollution (pm10)	423796
rs122035	6	396321	Particulate matter air pollution (pm10)	423796
rs125711	10	127771554	Particulate matter air pollution (pm10)	423796
rs145045	8	342124	Particulate matter air pollution (pm10)	423796
rs149130	11	83722884	Particulate matter air pollution (pm10)	423796
rs149752	2	33098355	Particulate matter air pollution (pm10)	423796
rs150993	10	64836267	Particulate matter air pollution (pm10)	423796
rs218514	18	1469513	Particulate matter air pollution (pm10)	423796
rs281223	13	51264767	Particulate matter air pollution (pm10)	423796
rs347764	15	57656713	Particulate matter air pollution (pm10)	423796
rs356652	5	51178554	Particulate matter air pollution (pm10)	423796
rs481513	20	286487	Particulate matter air pollution (pm10)	423796
rs570482	7	151623218	Particulate matter air pollution (pm10)	423796
rs614471	5	101445992	Particulate matter air pollution (pm10)	423796
rs621197	19	2279746	Particulate matter air pollution (pm10)	423796
rs728195	5	164643843	Particulate matter air pollution (pm10)	423796
rs732014	8	11512667	Particulate matter air pollution (pm10)	423796
rs747565	1	115028382	Particulate matter air pollution (pm10)	423796
rs750517	18	75220892	Particulate matter air pollution (pm10)	423796

rs790372	13	50507971	Particulate matter air pollution (pm10)	423796
rs802301	14	48380718	Particulate matter air pollution (pm10)	423796
rs803434	15	68925781	Particulate matter air pollution (pm10)	423796
rs103092	19	8779100	Average 24-hour sound level of noise p	456380
rs117527	8	117910936	Average 24-hour sound level of noise p	456380
rs143325	19	32674873	Average 24-hour sound level of noise p	456380
rs144374	7	150414185	Average 24-hour sound level of noise p	456380
rs150213	1	242381731	Average 24-hour sound level of noise p	456380
rs175904	3	25466098	Average 24-hour sound level of noise p	456380
rs189147	6	112596443	Average 24-hour sound level of noise p	456380
rs271487	7	17938491	Average 24-hour sound level of noise p	456380
rs576254	22	28666961	Average 24-hour sound level of noise p	456380
rs714785	11	102269128	Average 24-hour sound level of noise p	456380
rs724176	18	72116373	Average 24-hour sound level of noise p	456380
rs749208	13	80597508	Average 24-hour sound level of noise p	456380
rs109611	9	13603291	Particulate matter air pollution (pm2.5	423796
rs116925	8	93266536	Particulate matter air pollution (pm2.5	423796
rs117878	22	18475815	Particulate matter air pollution (pm2.5	423796
rs118289	7	75245569	Particulate matter air pollution (pm2.5	423796
rs118558	15	78012618	Particulate matter air pollution (pm2.5	423796
rs121330	1	91214714	Particulate matter air pollution (pm2.5	423796
rs121710	8	64567670	Particulate matter air pollution (pm2.5	423796
rs122035	6	396321	Particulate matter air pollution (pm2.5	423796
rs131884	4	153001662	Particulate matter air pollution (pm2.5	423796
rs137250	5	103749428	Particulate matter air pollution (pm2.5	423796
rs153737	9	22099568	Particulate matter air pollution (pm2.5	423796
rs169577	16	82610053	Particulate matter air pollution (pm2.5	423796
rs171039	14	76507299	Particulate matter air pollution (pm2.5	423796
rs171838	18	3100049	Particulate matter air pollution (pm2.5	423796
rs176578	7	17757841	Particulate matter air pollution (pm2.5	423796
rs214153	7	9253413	Particulate matter air pollution (pm2.5	423796
rs229215	16	49765133	Particulate matter air pollution (pm2.5	423796
rs27152	5	153734582	Particulate matter air pollution (pm2.5	423796
rs357035	11	61439682	Particulate matter air pollution (pm2.5	423796
rs485452	2	69051853	Particulate matter air pollution (pm2.5	423796
rs487230	1	55541174	Particulate matter air pollution (pm2.5	423796
rs560686	6	50597378	Particulate matter air pollution (pm2.5	423796
rs588248	5	137976541	Particulate matter air pollution (pm2.5	423796
rs613410	20	11007208	Particulate matter air pollution (pm2.5	423796
rs621197	19	2279746	Particulate matter air pollution (pm2.5	423796
rs621804	2	200815608	Particulate matter air pollution (pm2.5	423796
rs623686	5	80861930	Particulate matter air pollution (pm2.5	423796

rs6248466	7	104984561	Particulate matter air pollution (pm2.5	423796
rs6432776	2	164785611	Particulate matter air pollution (pm2.5	423796
rs6547952	2	22045642	Particulate matter air pollution (pm2.5	423796
rs6749461	2	343517	Particulate matter air pollution (pm2.5	423796
rs6960011	7	90302199	Particulate matter air pollution (pm2.5	423796
rs718059	14	34234984	Particulate matter air pollution (pm2.5	423796
rs7264243	18	45920421	Particulate matter air pollution (pm2.5	423796
rs7265589	13	112082676	Particulate matter air pollution (pm2.5	423796
rs7270000	14	104709808	Particulate matter air pollution (pm2.5	423796
rs7280802	5	164479164	Particulate matter air pollution (pm2.5	423796
rs7514956	1	74019696	Particulate matter air pollution (pm2.5	423796
rs7573056	2	58229428	Particulate matter air pollution (pm2.5	423796
rs7720573	8	10153460	Particulate matter air pollution (pm2.5	423796
rs7725581	6	20833602	Particulate matter air pollution (pm2.5	423796
rs7776279	6	163999770	Particulate matter air pollution (pm2.5	423796
rs7823038	8	35010055	Particulate matter air pollution (pm2.5	423796
rs7853976	12	12490981	Particulate matter air pollution (pm2.5	423796
rs8012381	3	148486829	Particulate matter air pollution (pm2.5	423796
rs8614	17	27588806	Particulate matter air pollution (pm2.5	423796
rs9260734	6	29932666	Particulate matter air pollution (pm2.5	423796
rs9644485	8	138230163	Particulate matter air pollution (pm2.5	423796
rs987204	3	94242461	Particulate matter air pollution (pm2.5	423796
rs1009402	8	8646438	Nitrogen dioxide air pollution	456380
rs1011621	9	22081397	Nitrogen dioxide air pollution	456380
rs1017229	2	58201202	Nitrogen dioxide air pollution	456380
rs1075045	11	130903582	Nitrogen dioxide air pollution	456380
rs1083851	11	45780039	Nitrogen dioxide air pollution	456380
rs1085260	16	49762462	Nitrogen dioxide air pollution	456380
rs1098373	9	120416066	Nitrogen dioxide air pollution	456380
rs1104924	12	28119596	Nitrogen dioxide air pollution	456380
rs1112882	1	227090717	Nitrogen dioxide air pollution	456380
rs1120476	1	150999737	Nitrogen dioxide air pollution	456380
rs1168101	2	135135102	Nitrogen dioxide air pollution	456380
rs1180913	9	20205067	Nitrogen dioxide air pollution	456380
rs1184458	14	34241004	Nitrogen dioxide air pollution	456380
rs1208981	1	91189933	Nitrogen dioxide air pollution	456380
rs1217106	8	64567670	Nitrogen dioxide air pollution	456380
rs1220359	6	396321	Nitrogen dioxide air pollution	456380
rs1245189	17	2247982	Nitrogen dioxide air pollution	456380
rs1318845	4	153001662	Nitrogen dioxide air pollution	456380
rs1325445	8	40707884	Nitrogen dioxide air pollution	456380
rs1342194	2	6777264	Nitrogen dioxide air pollution	456380

rs1372504	5	103749428	Nitrogen dioxide air pollution	456380
rs1378675	15	25488099	Nitrogen dioxide air pollution	456380
rs1718385	18	3100049	Nitrogen dioxide air pollution	456380
rs1751318	5	53585870	Nitrogen dioxide air pollution	456380
rs2058582	4	13892169	Nitrogen dioxide air pollution	456380
rs27152	5	153734582	Nitrogen dioxide air pollution	456380
rs2838761	21	46481557	Nitrogen dioxide air pollution	456380
rs2865594	2	69152401	Nitrogen dioxide air pollution	456380
rs2870984	2	21685654	Nitrogen dioxide air pollution	456380
rs329177	5	125130444	Nitrogen dioxide air pollution	456380
rs3462373	10	129281256	Nitrogen dioxide air pollution	456380
rs3562135	7	55380566	Nitrogen dioxide air pollution	456380
rs356543	2	164851354	Nitrogen dioxide air pollution	456380
rs3740390	10	104638480	Nitrogen dioxide air pollution	456380
rs3916875	19	45856820	Nitrogen dioxide air pollution	456380
rs4771073	13	27693827	Nitrogen dioxide air pollution	456380
rs4838594	10	49669142	Nitrogen dioxide air pollution	456380
rs533745	1	34419822	Nitrogen dioxide air pollution	456380
rs5564300	12	5773969	Nitrogen dioxide air pollution	456380
rs5597480	7	133771130	Nitrogen dioxide air pollution	456380
rs5611854	5	164465890	Nitrogen dioxide air pollution	456380
rs5729175	2	213625968	Nitrogen dioxide air pollution	456380
rs5882485	5	137976541	Nitrogen dioxide air pollution	456380
rs6174628	12	55968949	Nitrogen dioxide air pollution	456380
rs6177510	1	14054970	Nitrogen dioxide air pollution	456380
rs6206203	17	6255977	Nitrogen dioxide air pollution	456380
rs6211970	19	2279746	Nitrogen dioxide air pollution	456380
rs6245909	7	44268127	Nitrogen dioxide air pollution	456380
rs6749467	2	343517	Nitrogen dioxide air pollution	456380
rs6954825	7	101207035	Nitrogen dioxide air pollution	456380
rs7089175	10	2958311	Nitrogen dioxide air pollution	456380
rs714020	22	40493860	Nitrogen dioxide air pollution	456380
rs724643	7	88669096	Nitrogen dioxide air pollution	456380
rs7264243	18	45920421	Nitrogen dioxide air pollution	456380
rs7265589	13	112082676	Nitrogen dioxide air pollution	456380
rs7279542	16	84606181	Nitrogen dioxide air pollution	456380
rs7308584	7	30740079	Nitrogen dioxide air pollution	456380
rs7514956	1	74019696	Nitrogen dioxide air pollution	456380
rs7559133	15	83627623	Nitrogen dioxide air pollution	456380
rs7615084	3	174719405	Nitrogen dioxide air pollution	456380
rs7657249	4	105641593	Nitrogen dioxide air pollution	456380
rs7720573	8	10153460	Nitrogen dioxide air pollution	456380

rs775861	8	143137894	Nitrogen dioxide air pollution	456380
rs776063	6	87298301	Nitrogen dioxide air pollution	456380
rs777627	6	163999770	Nitrogen dioxide air pollution	456380
rs783874	16	54206097	Nitrogen dioxide air pollution	456380
rs785397	12	12490981	Nitrogen dioxide air pollution	456380
rs791020	10	15981388	Nitrogen dioxide air pollution	456380
rs801238	3	148486829	Nitrogen dioxide air pollution	456380
rs803397	8	27818236	Nitrogen dioxide air pollution	456380
rs936852	6	27679445	Nitrogen dioxide air pollution	456380
rs103092	19	8779100	Average 24-hour sound level of noise	456380
rs117527	8	117910936	Average 24-hour sound level of noise	456380
rs143325	19	32674873	Average 24-hour sound level of noise	456380
rs144374	7	150414185	Average 24-hour sound level of noise	456380
rs150213	1	242381731	Average 24-hour sound level of noise	456380
rs175904	3	25466098	Average 24-hour sound level of noise	456380
rs189147	6	112596443	Average 24-hour sound level of noise	456380
rs271487	7	17938491	Average 24-hour sound level of noise	456380
rs576254	22	28666961	Average 24-hour sound level of noise	456380
rs714785	11	102269128	Average 24-hour sound level of noise	456380
rs724176	18	72116373	Average 24-hour sound level of noise	456380
rs749208	13	80597508	Average 24-hour sound level of noise	456380
rs108462	12	16211780	Particulate matter air pollution (pm10	423796
rs109611	9	13603291	Particulate matter air pollution (pm10	423796
rs116215	14	103577789	Particulate matter air pollution (pm10	423796
rs117122	9	133311860	Particulate matter air pollution (pm10	423796
rs117671	6	159246615	Particulate matter air pollution (pm10	423796
rs121929	6	94818099	Particulate matter air pollution (pm10	423796
rs122035	6	396321	Particulate matter air pollution (pm10	423796
rs125711	10	127771554	Particulate matter air pollution (pm10	423796
rs145045	8	342124	Particulate matter air pollution (pm10	423796
rs149130	11	83722884	Particulate matter air pollution (pm10	423796
rs149752	2	33098355	Particulate matter air pollution (pm10	423796
rs150993	10	64836267	Particulate matter air pollution (pm10	423796
rs218514	18	1469513	Particulate matter air pollution (pm10	423796
rs281223	13	51264767	Particulate matter air pollution (pm10	423796
rs347764	15	57656713	Particulate matter air pollution (pm10	423796
rs356652	5	51178554	Particulate matter air pollution (pm10	423796
rs481513	20	286487	Particulate matter air pollution (pm10	423796
rs570482	7	151623218	Particulate matter air pollution (pm10	423796
rs614471	5	101445992	Particulate matter air pollution (pm10	423796
rs621197	19	2279746	Particulate matter air pollution (pm10	423796
rs728195	5	164643843	Particulate matter air pollution (pm10	423796

rs732014	8	11512667	Particulate matter air pollution (pm10	423796
rs747565	1	115028382	Particulate matter air pollution (pm10	423796
rs750517	18	75220892	Particulate matter air pollution (pm10	423796
rs790372	13	50507971	Particulate matter air pollution (pm10	423796
rs802301	14	48380718	Particulate matter air pollution (pm10	423796
rs803434	15	68925781	Particulate matter air pollution (pm10	423796
rs109611	9	13603291	Particulate matter air pollution (pm2.5	423796
rs116925	8	93266536	Particulate matter air pollution (pm2.5	423796
rs117878	22	18475815	Particulate matter air pollution (pm2.5	423796
rs118289	7	75245569	Particulate matter air pollution (pm2.5	423796
rs118558	15	78012618	Particulate matter air pollution (pm2.5	423796
rs121330	1	91214714	Particulate matter air pollution (pm2.5	423796
rs121710	8	64567670	Particulate matter air pollution (pm2.5	423796
rs122035	6	396321	Particulate matter air pollution (pm2.5	423796
rs131884	4	153001662	Particulate matter air pollution (pm2.5	423796
rs137250	5	103749428	Particulate matter air pollution (pm2.5	423796
rs153737	9	22099568	Particulate matter air pollution (pm2.5	423796
rs169577	16	82610053	Particulate matter air pollution (pm2.5	423796
rs171039	14	76507299	Particulate matter air pollution (pm2.5	423796
rs171838	18	3100049	Particulate matter air pollution (pm2.5	423796
rs176578	7	17757841	Particulate matter air pollution (pm2.5	423796
rs214153	7	9253413	Particulate matter air pollution (pm2.5	423796
rs229215	16	49765133	Particulate matter air pollution (pm2.5	423796
rs27152	5	153734582	Particulate matter air pollution (pm2.5	423796
rs357035	11	61439682	Particulate matter air pollution (pm2.5	423796
rs485452	2	69051853	Particulate matter air pollution (pm2.5	423796
rs487230	1	55541174	Particulate matter air pollution (pm2.5	423796
rs560686	6	50597378	Particulate matter air pollution (pm2.5	423796
rs588248	5	137976541	Particulate matter air pollution (pm2.5	423796
rs613410	20	11007208	Particulate matter air pollution (pm2.5	423796
rs621197	19	2279746	Particulate matter air pollution (pm2.5	423796
rs621804	2	200815608	Particulate matter air pollution (pm2.5	423796
rs623686	5	80861930	Particulate matter air pollution (pm2.5	423796
rs624846	7	104984561	Particulate matter air pollution (pm2.5	423796
rs643277	2	164785611	Particulate matter air pollution (pm2.5	423796
rs654795	2	22045642	Particulate matter air pollution (pm2.5	423796
rs674946	2	343517	Particulate matter air pollution (pm2.5	423796
rs696001	7	90302199	Particulate matter air pollution (pm2.5	423796
rs718059	14	34234984	Particulate matter air pollution (pm2.5	423796
rs726424	18	45920421	Particulate matter air pollution (pm2.5	423796
rs726558	13	112082676	Particulate matter air pollution (pm2.5	423796
rs727000	14	104709808	Particulate matter air pollution (pm2.5	423796

rs728080	5	164479164	Particulate matter air pollution (pm2.5	423796
rs751495	1	74019696	Particulate matter air pollution (pm2.5	423796
rs757305	2	58229428	Particulate matter air pollution (pm2.5	423796
rs772057	8	10153460	Particulate matter air pollution (pm2.5	423796
rs772558	6	20833602	Particulate matter air pollution (pm2.5	423796
rs777627	6	163999770	Particulate matter air pollution (pm2.5	423796
rs782303	8	35010055	Particulate matter air pollution (pm2.5	423796
rs785397	12	12490981	Particulate matter air pollution (pm2.5	423796
rs801238	3	148486829	Particulate matter air pollution (pm2.5	423796
rs8614	17	27588806	Particulate matter air pollution (pm2.5	423796
rs926073	6	29932666	Particulate matter air pollution (pm2.5	423796
rs964448	8	138230163	Particulate matter air pollution (pm2.5	423796
rs987204	3	94242461	Particulate matter air pollution (pm2.5	423796
rs100940	8	8646438	Nitrogen dioxide air pollution	456380
rs101162	9	22081397	Nitrogen dioxide air pollution	456380
rs101722	2	58201202	Nitrogen dioxide air pollution	456380
rs107504	11	130903582	Nitrogen dioxide air pollution	456380
rs108385	11	45780039	Nitrogen dioxide air pollution	456380
rs108526	16	49762462	Nitrogen dioxide air pollution	456380
rs109837	9	120416066	Nitrogen dioxide air pollution	456380
rs110492	12	28119596	Nitrogen dioxide air pollution	456380
rs111288	1	227090717	Nitrogen dioxide air pollution	456380
rs112047	1	150999737	Nitrogen dioxide air pollution	456380
rs116810	2	135135102	Nitrogen dioxide air pollution	456380
rs118091	9	20205067	Nitrogen dioxide air pollution	456380
rs118445	14	34241004	Nitrogen dioxide air pollution	456380
rs120898	1	91189933	Nitrogen dioxide air pollution	456380
rs121710	8	64567670	Nitrogen dioxide air pollution	456380
rs122035	6	396321	Nitrogen dioxide air pollution	456380
rs124518	17	2247982	Nitrogen dioxide air pollution	456380
rs131884	4	153001662	Nitrogen dioxide air pollution	456380
rs132544	8	40707884	Nitrogen dioxide air pollution	456380
rs134219	2	6777264	Nitrogen dioxide air pollution	456380
rs137250	5	103749428	Nitrogen dioxide air pollution	456380
rs137867	15	25488099	Nitrogen dioxide air pollution	456380
rs171838	18	3100049	Nitrogen dioxide air pollution	456380
rs175131	5	53585870	Nitrogen dioxide air pollution	456380
rs205858	4	13892169	Nitrogen dioxide air pollution	456380
rs27152	5	153734582	Nitrogen dioxide air pollution	456380
rs283876	21	46481557	Nitrogen dioxide air pollution	456380
rs286559	2	69152401	Nitrogen dioxide air pollution	456380
rs287098	2	21685654	Nitrogen dioxide air pollution	456380

rs329177	5	125130444	Nitrogen dioxide air pollution	456380
rs3462373	10	129281256	Nitrogen dioxide air pollution	456380
rs3562135	7	55380566	Nitrogen dioxide air pollution	456380
rs356543	2	164851354	Nitrogen dioxide air pollution	456380
rs3740390	10	104638480	Nitrogen dioxide air pollution	456380
rs3916875	19	45856820	Nitrogen dioxide air pollution	456380
rs4771073	13	27693827	Nitrogen dioxide air pollution	456380
rs4838594	10	49669142	Nitrogen dioxide air pollution	456380
rs533745	1	34419822	Nitrogen dioxide air pollution	456380
rs5564300	12	5773969	Nitrogen dioxide air pollution	456380
rs5597480	7	133771130	Nitrogen dioxide air pollution	456380
rs5611854	5	164465890	Nitrogen dioxide air pollution	456380
rs5729175	2	213625968	Nitrogen dioxide air pollution	456380
rs5882485	5	137976541	Nitrogen dioxide air pollution	456380
rs6174628	12	55968949	Nitrogen dioxide air pollution	456380
rs6177510	1	14054970	Nitrogen dioxide air pollution	456380
rs6206203	17	6255977	Nitrogen dioxide air pollution	456380
rs6211970	19	2279746	Nitrogen dioxide air pollution	456380
rs6245909	7	44268127	Nitrogen dioxide air pollution	456380
rs6749467	2	343517	Nitrogen dioxide air pollution	456380
rs6954825	7	101207035	Nitrogen dioxide air pollution	456380
rs7089175	10	2958311	Nitrogen dioxide air pollution	456380
rs714020	22	40493860	Nitrogen dioxide air pollution	456380
rs724643	7	88669096	Nitrogen dioxide air pollution	456380
rs7264243	18	45920421	Nitrogen dioxide air pollution	456380
rs7265589	13	112082676	Nitrogen dioxide air pollution	456380
rs7279542	16	84606181	Nitrogen dioxide air pollution	456380
rs7308584	7	30740079	Nitrogen dioxide air pollution	456380
rs7514956	1	74019696	Nitrogen dioxide air pollution	456380
rs7559133	15	83627623	Nitrogen dioxide air pollution	456380
rs7615084	3	174719405	Nitrogen dioxide air pollution	456380
rs7657249	4	105641593	Nitrogen dioxide air pollution	456380
rs7720573	8	10153460	Nitrogen dioxide air pollution	456380
rs7758613	8	143137894	Nitrogen dioxide air pollution	456380
rs7760633	6	87298301	Nitrogen dioxide air pollution	456380
rs7776279	6	163999770	Nitrogen dioxide air pollution	456380
rs7838740	16	54206097	Nitrogen dioxide air pollution	456380
rs7853976	12	12490981	Nitrogen dioxide air pollution	456380
rs7910200	10	15981388	Nitrogen dioxide air pollution	456380
rs8012387	3	148486829	Nitrogen dioxide air pollution	456380
rs8033978	8	27818236	Nitrogen dioxide air pollution	456380
rs9368527	6	27679445	Nitrogen dioxide air pollution	456380

rs108462	12	16211780	Particulate matter air pollution (pm10	423796
rs109611	9	13603291	Particulate matter air pollution (pm10	423796
rs116215	14	103577789	Particulate matter air pollution (pm10	423796
rs117122	9	133311860	Particulate matter air pollution (pm10	423796
rs117671	6	159246615	Particulate matter air pollution (pm10	423796
rs121929	6	94818099	Particulate matter air pollution (pm10	423796
rs122035	6	396321	Particulate matter air pollution (pm10	423796
rs125711	10	127771554	Particulate matter air pollution (pm10	423796
rs145045	8	342124	Particulate matter air pollution (pm10	423796
rs149130	11	83722884	Particulate matter air pollution (pm10	423796
rs149752	2	33098355	Particulate matter air pollution (pm10	423796
rs150993	10	64836267	Particulate matter air pollution (pm10	423796
rs218514	18	1469513	Particulate matter air pollution (pm10	423796
rs281223	13	51264767	Particulate matter air pollution (pm10	423796
rs347764	15	57656713	Particulate matter air pollution (pm10	423796
rs356652	5	51178554	Particulate matter air pollution (pm10	423796
rs481513	20	286487	Particulate matter air pollution (pm10	423796
rs570482	7	151623218	Particulate matter air pollution (pm10	423796
rs614471	5	101445992	Particulate matter air pollution (pm10	423796
rs621197	19	2279746	Particulate matter air pollution (pm10	423796
rs728195	5	164643843	Particulate matter air pollution (pm10	423796
rs732014	8	11512667	Particulate matter air pollution (pm10	423796
rs747565	1	115028382	Particulate matter air pollution (pm10	423796
rs750517	18	75220892	Particulate matter air pollution (pm10	423796
rs790372	13	50507971	Particulate matter air pollution (pm10	423796
rs802301	14	48380718	Particulate matter air pollution (pm10	423796
rs803434	15	68925781	Particulate matter air pollution (pm10	423796
rs103092	19	8779100	Average 24-hour sound level of noise	456380
rs117527	8	117910936	Average 24-hour sound level of noise	456380
rs143325	19	32674873	Average 24-hour sound level of noise	456380
rs144374	7	150414185	Average 24-hour sound level of noise	456380
rs150213	1	242381731	Average 24-hour sound level of noise	456380
rs175904	3	25466098	Average 24-hour sound level of noise	456380
rs189147	6	112596443	Average 24-hour sound level of noise	456380
rs271487	7	17938491	Average 24-hour sound level of noise	456380
rs576254	22	28666961	Average 24-hour sound level of noise	456380
rs714785	11	102269128	Average 24-hour sound level of noise	456380
rs724176	18	72116373	Average 24-hour sound level of noise	456380
rs749208	13	80597508	Average 24-hour sound level of noise	456380
rs100940	8	8646438	Nitrogen dioxide air pollution	456380
rs101162	9	22081397	Nitrogen dioxide air pollution	456380
rs101722	2	58201202	Nitrogen dioxide air pollution	456380

rs107504[illegible]	11	130903582	Nitrogen dioxide air pollution	456380
rs108385[illegible]	11	45780039	Nitrogen dioxide air pollution	456380
rs108526[illegible]	16	49762462	Nitrogen dioxide air pollution	456380
rs109837[illegible]	9	120416066	Nitrogen dioxide air pollution	456380
rs110492[illegible]	12	28119596	Nitrogen dioxide air pollution	456380
rs111288[illegible]	1	227090717	Nitrogen dioxide air pollution	456380
rs112047[illegible]	1	150999737	Nitrogen dioxide air pollution	456380
rs116810[illegible]	2	135135102	Nitrogen dioxide air pollution	456380
rs118091[illegible]	9	20205067	Nitrogen dioxide air pollution	456380
rs118445[illegible]	14	34241004	Nitrogen dioxide air pollution	456380
rs120898[illegible]	1	91189933	Nitrogen dioxide air pollution	456380
rs121710[illegible]	8	64567670	Nitrogen dioxide air pollution	456380
rs122035[illegible]	6	396321	Nitrogen dioxide air pollution	456380
rs124518[illegible]	17	2247982	Nitrogen dioxide air pollution	456380
rs131884[illegible]	4	153001662	Nitrogen dioxide air pollution	456380
rs132544[illegible]	8	40707884	Nitrogen dioxide air pollution	456380
rs134219[illegible]	2	6777264	Nitrogen dioxide air pollution	456380
rs137250[illegible]	5	103749428	Nitrogen dioxide air pollution	456380
rs137867[illegible]	15	25488099	Nitrogen dioxide air pollution	456380
rs171838[illegible]	18	3100049	Nitrogen dioxide air pollution	456380
rs175131[illegible]	5	53585870	Nitrogen dioxide air pollution	456380
rs205858[illegible]	4	13892169	Nitrogen dioxide air pollution	456380
rs27152	5	153734582	Nitrogen dioxide air pollution	456380
rs283876[illegible]	21	46481557	Nitrogen dioxide air pollution	456380
rs286559[illegible]	2	69152401	Nitrogen dioxide air pollution	456380
rs287098[illegible]	2	21685654	Nitrogen dioxide air pollution	456380
rs329177	5	125130444	Nitrogen dioxide air pollution	456380
rs346237[illegible]	10	129281256	Nitrogen dioxide air pollution	456380
rs356213[illegible]	7	55380566	Nitrogen dioxide air pollution	456380
rs356543	2	164851354	Nitrogen dioxide air pollution	456380
rs374039[illegible]	10	104638480	Nitrogen dioxide air pollution	456380
rs391687[illegible]	19	45856820	Nitrogen dioxide air pollution	456380
rs477107[illegible]	13	27693827	Nitrogen dioxide air pollution	456380
rs483859[illegible]	10	49669142	Nitrogen dioxide air pollution	456380
rs533745	1	34419822	Nitrogen dioxide air pollution	456380
rs556430[illegible]	12	5773969	Nitrogen dioxide air pollution	456380
rs559748[illegible]	7	133771130	Nitrogen dioxide air pollution	456380
rs561185[illegible]	5	164465890	Nitrogen dioxide air pollution	456380
rs572917[illegible]	2	213625968	Nitrogen dioxide air pollution	456380
rs588248[illegible]	5	137976541	Nitrogen dioxide air pollution	456380
rs617462[illegible]	12	55968949	Nitrogen dioxide air pollution	456380
rs617751[illegible]	1	14054970	Nitrogen dioxide air pollution	456380

rs6206203	17	6255977	Nitrogen dioxide air pollution	456380
rs6211970	19	2279746	Nitrogen dioxide air pollution	456380
rs6245909	7	44268127	Nitrogen dioxide air pollution	456380
rs6749461	2	343517	Nitrogen dioxide air pollution	456380
rs6954825	7	101207035	Nitrogen dioxide air pollution	456380
rs7089175	10	2958311	Nitrogen dioxide air pollution	456380
rs714020	22	40493860	Nitrogen dioxide air pollution	456380
rs724643	7	88669096	Nitrogen dioxide air pollution	456380
rs7264243	18	45920421	Nitrogen dioxide air pollution	456380
rs7265589	13	112082676	Nitrogen dioxide air pollution	456380
rs7279542	16	84606181	Nitrogen dioxide air pollution	456380
rs7308584	7	30740079	Nitrogen dioxide air pollution	456380
rs7514956	1	74019696	Nitrogen dioxide air pollution	456380
rs7559133	15	83627623	Nitrogen dioxide air pollution	456380
rs7615084	3	174719405	Nitrogen dioxide air pollution	456380
rs7657249	4	105641593	Nitrogen dioxide air pollution	456380
rs7720573	8	10153460	Nitrogen dioxide air pollution	456380
rs7758613	8	143137894	Nitrogen dioxide air pollution	456380
rs7760633	6	87298301	Nitrogen dioxide air pollution	456380
rs7776279	6	163999770	Nitrogen dioxide air pollution	456380
rs7838740	16	54206097	Nitrogen dioxide air pollution	456380
rs7853976	12	12490981	Nitrogen dioxide air pollution	456380
rs7910200	10	15981388	Nitrogen dioxide air pollution	456380
rs8012387	3	148486829	Nitrogen dioxide air pollution	456380
rs8033978	8	27818236	Nitrogen dioxide air pollution	456380
rs9368527	6	27679445	Nitrogen dioxide air pollution	456380
rs1096116	9	13603291	Particulate matter air pollution (pm2.5	423796
rs1169251	8	93266536	Particulate matter air pollution (pm2.5	423796
rs1178782	22	18475815	Particulate matter air pollution (pm2.5	423796
rs1182891	7	75245569	Particulate matter air pollution (pm2.5	423796
rs1185582	15	78012618	Particulate matter air pollution (pm2.5	423796
rs1213306	1	91214714	Particulate matter air pollution (pm2.5	423796
rs1217106	8	64567670	Particulate matter air pollution (pm2.5	423796
rs1220359	6	396321	Particulate matter air pollution (pm2.5	423796
rs1318845	4	153001662	Particulate matter air pollution (pm2.5	423796
rs1372504	5	103749428	Particulate matter air pollution (pm2.5	423796
rs1537371	9	22099568	Particulate matter air pollution (pm2.5	423796
rs1695775	16	82610053	Particulate matter air pollution (pm2.5	423796
rs1710392	14	76507299	Particulate matter air pollution (pm2.5	423796
rs1718385	18	3100049	Particulate matter air pollution (pm2.5	423796
rs1765788	7	17757841	Particulate matter air pollution (pm2.5	423796
rs2141531	7	9253413	Particulate matter air pollution (pm2.5	423796

rs229215[illegible]	16	49765133	Particulate matter air pollution (pm2.5	423796
rs27152	5	153734582	Particulate matter air pollution (pm2.5	423796
rs357035[illegible]	11	61439682	Particulate matter air pollution (pm2.5	423796
rs485452[illegible]	2	69051853	Particulate matter air pollution (pm2.5	423796
rs487230	1	55541174	Particulate matter air pollution (pm2.5	423796
rs560686[illegible]	6	50597378	Particulate matter air pollution (pm2.5	423796
rs588248[illegible]	5	137976541	Particulate matter air pollution (pm2.5	423796
rs613410[illegible]	20	11007208	Particulate matter air pollution (pm2.5	423796
rs621197[illegible]	19	2279746	Particulate matter air pollution (pm2.5	423796
rs621804[illegible]	2	200815608	Particulate matter air pollution (pm2.5	423796
rs623686[illegible]	5	80861930	Particulate matter air pollution (pm2.5	423796
rs624846[illegible]	7	104984561	Particulate matter air pollution (pm2.5	423796
rs643277[illegible]	2	164785611	Particulate matter air pollution (pm2.5	423796
rs654795[illegible]	2	22045642	Particulate matter air pollution (pm2.5	423796
rs674946[illegible]	2	343517	Particulate matter air pollution (pm2.5	423796
rs696001[illegible]	7	90302199	Particulate matter air pollution (pm2.5	423796
rs718059	14	34234984	Particulate matter air pollution (pm2.5	423796
rs726424[illegible]	18	45920421	Particulate matter air pollution (pm2.5	423796
rs726558[illegible]	13	112082676	Particulate matter air pollution (pm2.5	423796
rs727000[illegible]	14	104709808	Particulate matter air pollution (pm2.5	423796
rs728080[illegible]	5	164479164	Particulate matter air pollution (pm2.5	423796
rs751495[illegible]	1	74019696	Particulate matter air pollution (pm2.5	423796
rs757305[illegible]	2	58229428	Particulate matter air pollution (pm2.5	423796
rs772057[illegible]	8	10153460	Particulate matter air pollution (pm2.5	423796
rs772558[illegible]	6	20833602	Particulate matter air pollution (pm2.5	423796
rs777627[illegible]	6	163999770	Particulate matter air pollution (pm2.5	423796
rs782303[illegible]	8	35010055	Particulate matter air pollution (pm2.5	423796
rs785397[illegible]	12	12490981	Particulate matter air pollution (pm2.5	423796
rs801238[illegible]	3	148486829	Particulate matter air pollution (pm2.5	423796
rs8614	17	27588806	Particulate matter air pollution (pm2.5	423796
rs926073[illegible]	6	29932666	Particulate matter air pollution (pm2.5	423796
rs964448[illegible]	8	138230163	Particulate matter air pollution (pm2.5	423796
rs987204	3	94242461	Particulate matter air pollution (pm2.5	423796
rs108462[illegible]	12	16211780	Particulate matter air pollution (pm10)	423796
rs109611[illegible]	9	13603291	Particulate matter air pollution (pm10)	423796
rs116215[illegible]	14	103577789	Particulate matter air pollution (pm10)	423796
rs117122[illegible]	9	133311860	Particulate matter air pollution (pm10)	423796
rs117671[illegible]	6	159246615	Particulate matter air pollution (pm10)	423796
rs121929[illegible]	6	94818099	Particulate matter air pollution (pm10)	423796
rs122035[illegible]	6	396321	Particulate matter air pollution (pm10)	423796
rs125711[illegible]	10	127771554	Particulate matter air pollution (pm10)	423796
rs145045[illegible]	8	342124	Particulate matter air pollution (pm10)	423796

rs149130	11	83722884	Particulate matter air pollution (pm10	423796
rs149752	2	33098355	Particulate matter air pollution (pm10	423796
rs150993	10	64836267	Particulate matter air pollution (pm10	423796
rs218514	18	1469513	Particulate matter air pollution (pm10	423796
rs281223	13	51264767	Particulate matter air pollution (pm10	423796
rs347764	15	57656713	Particulate matter air pollution (pm10	423796
rs356652	5	51178554	Particulate matter air pollution (pm10	423796
rs481513	20	286487	Particulate matter air pollution (pm10	423796
rs570482	7	151623218	Particulate matter air pollution (pm10	423796
rs614471	5	101445992	Particulate matter air pollution (pm10	423796
rs621197	19	2279746	Particulate matter air pollution (pm10	423796
rs728195	5	164643843	Particulate matter air pollution (pm10	423796
rs732014	8	11512667	Particulate matter air pollution (pm10	423796
rs747565	1	115028382	Particulate matter air pollution (pm10	423796
rs750517	18	75220892	Particulate matter air pollution (pm10	423796
rs790372	13	50507971	Particulate matter air pollution (pm10	423796
rs802301	14	48380718	Particulate matter air pollution (pm10	423796
rs803434	15	68925781	Particulate matter air pollution (pm10	423796
rs103092	19	8779100	Average 24-hour sound level of noise	456380
rs117527	8	117910936	Average 24-hour sound level of noise	456380
rs143325	19	32674873	Average 24-hour sound level of noise	456380
rs144374	7	150414185	Average 24-hour sound level of noise	456380
rs150213	1	242381731	Average 24-hour sound level of noise	456380
rs175904	3	25466098	Average 24-hour sound level of noise	456380
rs189147	6	112596443	Average 24-hour sound level of noise	456380
rs271487	7	17938491	Average 24-hour sound level of noise	456380
rs576254	22	28666961	Average 24-hour sound level of noise	456380
rs714785	11	102269128	Average 24-hour sound level of noise	456380
rs724176	18	72116373	Average 24-hour sound level of noise	456380
rs749208	13	80597508	Average 24-hour sound level of noise	456380
rs100940	8	8646438	Nitrogen dioxide air pollution	456380
rs101162	9	22081397	Nitrogen dioxide air pollution	456380
rs101722	2	58201202	Nitrogen dioxide air pollution	456380
rs107504	11	130903582	Nitrogen dioxide air pollution	456380
rs108385	11	45780039	Nitrogen dioxide air pollution	456380
rs108526	16	49762462	Nitrogen dioxide air pollution	456380
rs109837	9	120416066	Nitrogen dioxide air pollution	456380
rs110492	12	28119596	Nitrogen dioxide air pollution	456380
rs111288	1	227090717	Nitrogen dioxide air pollution	456380
rs112047	1	150999737	Nitrogen dioxide air pollution	456380
rs116810	2	135135102	Nitrogen dioxide air pollution	456380
rs118445	14	34241004	Nitrogen dioxide air pollution	456380

rs120898	1	91189933	Nitrogen dioxide air pollution	456380
rs121710	8	64567670	Nitrogen dioxide air pollution	456380
rs122035	6	396321	Nitrogen dioxide air pollution	456380
rs124518	17	2247982	Nitrogen dioxide air pollution	456380
rs131884	4	153001662	Nitrogen dioxide air pollution	456380
rs132544	8	40707884	Nitrogen dioxide air pollution	456380
rs134219	2	6777264	Nitrogen dioxide air pollution	456380
rs137250	5	103749428	Nitrogen dioxide air pollution	456380
rs137867	15	25488099	Nitrogen dioxide air pollution	456380
rs175131	5	53585870	Nitrogen dioxide air pollution	456380
rs205858	4	13892169	Nitrogen dioxide air pollution	456380
rs27152	5	153734582	Nitrogen dioxide air pollution	456380
rs283876	21	46481557	Nitrogen dioxide air pollution	456380
rs286559	2	69152401	Nitrogen dioxide air pollution	456380
rs287098	2	21685654	Nitrogen dioxide air pollution	456380
rs329177	5	125130444	Nitrogen dioxide air pollution	456380
rs346237	10	129281256	Nitrogen dioxide air pollution	456380
rs356213	7	55380566	Nitrogen dioxide air pollution	456380
rs356543	2	164851354	Nitrogen dioxide air pollution	456380
rs374039	10	104638480	Nitrogen dioxide air pollution	456380
rs391687	19	45856820	Nitrogen dioxide air pollution	456380
rs477107	13	27693827	Nitrogen dioxide air pollution	456380
rs483859	10	49669142	Nitrogen dioxide air pollution	456380
rs533745	1	34419822	Nitrogen dioxide air pollution	456380
rs556430	12	5773969	Nitrogen dioxide air pollution	456380
rs559748	7	133771130	Nitrogen dioxide air pollution	456380
rs561185	5	164465890	Nitrogen dioxide air pollution	456380
rs588248	5	137976541	Nitrogen dioxide air pollution	456380
rs617462	12	55968949	Nitrogen dioxide air pollution	456380
rs617751	1	14054970	Nitrogen dioxide air pollution	456380
rs620620	17	6254811	Nitrogen dioxide air pollution	456380
rs621197	19	2279746	Nitrogen dioxide air pollution	456380
rs624590	7	44268127	Nitrogen dioxide air pollution	456380
rs666734	1	8197477	Nitrogen dioxide air pollution	456380
rs674946	2	343517	Nitrogen dioxide air pollution	456380
rs695482	7	101207035	Nitrogen dioxide air pollution	456380
rs708917	10	2958311	Nitrogen dioxide air pollution	456380
rs714020	22	40493860	Nitrogen dioxide air pollution	456380
rs724643	7	88669096	Nitrogen dioxide air pollution	456380
rs726424	18	45920421	Nitrogen dioxide air pollution	456380
rs726558	13	112082676	Nitrogen dioxide air pollution	456380
rs727954	16	84606181	Nitrogen dioxide air pollution	456380

rs730858	7	30740079	Nitrogen dioxide air pollution	456380
rs751495	1	74019696	Nitrogen dioxide air pollution	456380
rs755913	15	83627623	Nitrogen dioxide air pollution	456380
rs761508	3	174719405	Nitrogen dioxide air pollution	456380
rs765724	4	105641593	Nitrogen dioxide air pollution	456380
rs772057	8	10153460	Nitrogen dioxide air pollution	456380
rs775861	8	143137894	Nitrogen dioxide air pollution	456380
rs776063	6	87297558	Nitrogen dioxide air pollution	456380
rs777627	6	163999770	Nitrogen dioxide air pollution	456380
rs783874	16	54206097	Nitrogen dioxide air pollution	456380
rs785397	12	12490981	Nitrogen dioxide air pollution	456380
rs791020	10	15981388	Nitrogen dioxide air pollution	456380
rs794750	6	20992197	Nitrogen dioxide air pollution	456380
rs801238	3	148486829	Nitrogen dioxide air pollution	456380
rs803397	8	27818236	Nitrogen dioxide air pollution	456380
rs936852	6	27679445	Nitrogen dioxide air pollution	456380
rs108462	12	16211780	Particulate matter air pollution (pm10)	423796
rs109611	9	13603291	Particulate matter air pollution (pm10)	423796
rs116215	14	103577789	Particulate matter air pollution (pm10)	423796
rs117122	9	133311860	Particulate matter air pollution (pm10)	423796
rs117671	6	159246615	Particulate matter air pollution (pm10)	423796
rs121929	6	94818099	Particulate matter air pollution (pm10)	423796
rs122035	6	396321	Particulate matter air pollution (pm10)	423796
rs125711	10	127771554	Particulate matter air pollution (pm10)	423796
rs145045	8	342124	Particulate matter air pollution (pm10)	423796
rs149752	2	33098355	Particulate matter air pollution (pm10)	423796
rs150993	10	64836267	Particulate matter air pollution (pm10)	423796
rs218514	18	1469513	Particulate matter air pollution (pm10)	423796
rs281223	13	51264767	Particulate matter air pollution (pm10)	423796
rs347764	15	57656713	Particulate matter air pollution (pm10)	423796
rs356652	5	51178554	Particulate matter air pollution (pm10)	423796
rs481513	20	286487	Particulate matter air pollution (pm10)	423796
rs570482	7	151623218	Particulate matter air pollution (pm10)	423796
rs614471	5	101445992	Particulate matter air pollution (pm10)	423796
rs621197	19	2279746	Particulate matter air pollution (pm10)	423796
rs728195	5	164643843	Particulate matter air pollution (pm10)	423796
rs732014	8	11512667	Particulate matter air pollution (pm10)	423796
rs747565	1	115028382	Particulate matter air pollution (pm10)	423796
rs750517	18	75220892	Particulate matter air pollution (pm10)	423796
rs790372	13	50507971	Particulate matter air pollution (pm10)	423796
rs802301	14	48380718	Particulate matter air pollution (pm10)	423796
rs803434	15	68925781	Particulate matter air pollution (pm10)	423796

rs103092[illegible]	19	8779100	Average 24-hour sound level of noise p	456380
rs117527[illegible]	8	117910936	Average 24-hour sound level of noise p	456380
rs143325[illegible]	19	32674873	Average 24-hour sound level of noise p	456380
rs144374[illegible]	7	150414185	Average 24-hour sound level of noise p	456380
rs150213[illegible]	1	242381731	Average 24-hour sound level of noise p	456380
rs175904[illegible]	3	25466098	Average 24-hour sound level of noise p	456380
rs189147[illegible]	6	112596443	Average 24-hour sound level of noise p	456380
rs271487[illegible]	7	17938491	Average 24-hour sound level of noise p	456380
rs576254[illegible]	22	28666961	Average 24-hour sound level of noise p	456380
rs714785[illegible]	11	102269128	Average 24-hour sound level of noise p	456380
rs724176[illegible]	18	72116373	Average 24-hour sound level of noise p	456380
rs749208[illegible]	13	80597508	Average 24-hour sound level of noise p	456380
rs109611[illegible]	9	13603291	Particulate matter air pollution (pm2.5	423796
rs110423[illegible]	11	2051631	Particulate matter air pollution (pm2.5	423796
rs116925[illegible]	8	93266536	Particulate matter air pollution (pm2.5	423796
rs117878[illegible]	22	18475815	Particulate matter air pollution (pm2.5	423796
rs118289[illegible]	7	75245569	Particulate matter air pollution (pm2.5	423796
rs118558[illegible]	15	78008843	Particulate matter air pollution (pm2.5	423796
rs121330[illegible]	1	91214714	Particulate matter air pollution (pm2.5	423796
rs121710[illegible]	8	64567670	Particulate matter air pollution (pm2.5	423796
rs122035[illegible]	6	396321	Particulate matter air pollution (pm2.5	423796
rs128125[illegible]	12	62341242	Particulate matter air pollution (pm2.5	423796
rs131884[illegible]	4	153001662	Particulate matter air pollution (pm2.5	423796
rs137250[illegible]	5	103749428	Particulate matter air pollution (pm2.5	423796
rs153737[illegible]	9	22099568	Particulate matter air pollution (pm2.5	423796
rs169577[illegible]	16	82610053	Particulate matter air pollution (pm2.5	423796
rs171039[illegible]	14	76507299	Particulate matter air pollution (pm2.5	423796
rs176578[illegible]	7	17757841	Particulate matter air pollution (pm2.5	423796
rs214153[illegible]	7	9253413	Particulate matter air pollution (pm2.5	423796
rs229215[illegible]	16	49765133	Particulate matter air pollution (pm2.5	423796
rs27152	5	153734582	Particulate matter air pollution (pm2.5	423796
rs357035[illegible]	11	61439682	Particulate matter air pollution (pm2.5	423796
rs485452[illegible]	2	69051853	Particulate matter air pollution (pm2.5	423796
rs487230	1	55541174	Particulate matter air pollution (pm2.5	423796
rs560686[illegible]	6	50597378	Particulate matter air pollution (pm2.5	423796
rs588248[illegible]	5	137976541	Particulate matter air pollution (pm2.5	423796
rs613410[illegible]	20	11007208	Particulate matter air pollution (pm2.5	423796
rs621197[illegible]	19	2279746	Particulate matter air pollution (pm2.5	423796
rs621804[illegible]	2	200815608	Particulate matter air pollution (pm2.5	423796
rs623686[illegible]	5	80861930	Particulate matter air pollution (pm2.5	423796
rs624846[illegible]	7	104872861	Particulate matter air pollution (pm2.5	423796
rs643277[illegible]	2	164785611	Particulate matter air pollution (pm2.5	423796

rs654795	2	22045642	Particulate matter air pollution (pm2.5	423796
rs674946	2	343517	Particulate matter air pollution (pm2.5	423796
rs696001	7	90302199	Particulate matter air pollution (pm2.5	423796
rs718059	14	34234984	Particulate matter air pollution (pm2.5	423796
rs726424	18	45920421	Particulate matter air pollution (pm2.5	423796
rs726558	13	112082676	Particulate matter air pollution (pm2.5	423796
rs727000	14	104709808	Particulate matter air pollution (pm2.5	423796
rs728080	5	164479164	Particulate matter air pollution (pm2.5	423796
rs751495	1	74019696	Particulate matter air pollution (pm2.5	423796
rs757305	2	58229428	Particulate matter air pollution (pm2.5	423796
rs772057	8	10153460	Particulate matter air pollution (pm2.5	423796
rs772558	6	20833602	Particulate matter air pollution (pm2.5	423796
rs777627	6	163999770	Particulate matter air pollution (pm2.5	423796
rs782303	8	35010055	Particulate matter air pollution (pm2.5	423796
rs785397	12	12490981	Particulate matter air pollution (pm2.5	423796
rs785460	10	48642432	Particulate matter air pollution (pm2.5	423796
rs801238	3	148486829	Particulate matter air pollution (pm2.5	423796
rs8614	17	27588806	Particulate matter air pollution (pm2.5	423796
rs964448	8	138230163	Particulate matter air pollution (pm2.5	423796
rs987204	3	94242461	Particulate matter air pollution (pm2.5	423796
rs100940	8	8646438	Nitrogen dioxide air pollution	456380
rs101162	9	22081397	Nitrogen dioxide air pollution	456380
rs101722	2	58201202	Nitrogen dioxide air pollution	456380
rs107504	11	130903582	Nitrogen dioxide air pollution	456380
rs108385	11	45780039	Nitrogen dioxide air pollution	456380
rs108526	16	49762462	Nitrogen dioxide air pollution	456380
rs109837	9	120416066	Nitrogen dioxide air pollution	456380
rs110492	12	28119596	Nitrogen dioxide air pollution	456380
rs111288	1	227090717	Nitrogen dioxide air pollution	456380
rs112047	1	150999737	Nitrogen dioxide air pollution	456380
rs116810	2	135135102	Nitrogen dioxide air pollution	456380
rs118445	14	34241004	Nitrogen dioxide air pollution	456380
rs120898	1	91189933	Nitrogen dioxide air pollution	456380
rs121710	8	64567670	Nitrogen dioxide air pollution	456380
rs122035	6	396321	Nitrogen dioxide air pollution	456380
rs124518	17	2247982	Nitrogen dioxide air pollution	456380
rs131884	4	153001662	Nitrogen dioxide air pollution	456380
rs132544	8	40707884	Nitrogen dioxide air pollution	456380
rs134219	2	6777264	Nitrogen dioxide air pollution	456380
rs137250	5	103749428	Nitrogen dioxide air pollution	456380
rs137867	15	25488099	Nitrogen dioxide air pollution	456380
rs175131	5	53585870	Nitrogen dioxide air pollution	456380

rs2058582	4	13892169	Nitrogen dioxide air pollution	456380
rs27152	5	153734582	Nitrogen dioxide air pollution	456380
rs2838761	21	46481557	Nitrogen dioxide air pollution	456380
rs2865594	2	69152401	Nitrogen dioxide air pollution	456380
rs2870984	2	21685654	Nitrogen dioxide air pollution	456380
rs329177	5	125130444	Nitrogen dioxide air pollution	456380
rs3462373	10	129281256	Nitrogen dioxide air pollution	456380
rs3562135	7	55380566	Nitrogen dioxide air pollution	456380
rs356543	2	164851354	Nitrogen dioxide air pollution	456380
rs3740390	10	104638480	Nitrogen dioxide air pollution	456380
rs3916875	19	45856820	Nitrogen dioxide air pollution	456380
rs4771073	13	27693827	Nitrogen dioxide air pollution	456380
rs4838594	10	49669142	Nitrogen dioxide air pollution	456380
rs533745	1	34419822	Nitrogen dioxide air pollution	456380
rs5564300	12	5773969	Nitrogen dioxide air pollution	456380
rs5597480	7	133771130	Nitrogen dioxide air pollution	456380
rs5611854	5	164465890	Nitrogen dioxide air pollution	456380
rs5882485	5	137976541	Nitrogen dioxide air pollution	456380
rs6174628	12	55968949	Nitrogen dioxide air pollution	456380
rs6177510	1	14054970	Nitrogen dioxide air pollution	456380
rs6206203	17	6254811	Nitrogen dioxide air pollution	456380
rs6211970	19	2279746	Nitrogen dioxide air pollution	456380
rs6245909	7	44268127	Nitrogen dioxide air pollution	456380
rs6667345	1	8197477	Nitrogen dioxide air pollution	456380
rs6749467	2	343517	Nitrogen dioxide air pollution	456380
rs6954825	7	101207035	Nitrogen dioxide air pollution	456380
rs7089175	10	2958311	Nitrogen dioxide air pollution	456380
rs714020	22	40493860	Nitrogen dioxide air pollution	456380
rs724643	7	88669096	Nitrogen dioxide air pollution	456380
rs7264243	18	45920421	Nitrogen dioxide air pollution	456380
rs7265589	13	112082676	Nitrogen dioxide air pollution	456380
rs7279542	16	84606181	Nitrogen dioxide air pollution	456380
rs7308584	7	30740079	Nitrogen dioxide air pollution	456380
rs7514956	1	74019696	Nitrogen dioxide air pollution	456380
rs7559133	15	83627623	Nitrogen dioxide air pollution	456380
rs7615084	3	174719405	Nitrogen dioxide air pollution	456380
rs7657249	4	105641593	Nitrogen dioxide air pollution	456380
rs7720573	8	10153460	Nitrogen dioxide air pollution	456380
rs7758613	8	143137894	Nitrogen dioxide air pollution	456380
rs7760633	6	87297558	Nitrogen dioxide air pollution	456380
rs7776279	6	163999770	Nitrogen dioxide air pollution	456380
rs7838740	16	54206097	Nitrogen dioxide air pollution	456380

rs7853976	12	12490981	Nitrogen dioxide air pollution	456380
rs7910200	10	15981388	Nitrogen dioxide air pollution	456380
rs7947504	6	20992197	Nitrogen dioxide air pollution	456380
rs8012381	3	148486829	Nitrogen dioxide air pollution	456380
rs8033978	8	27818236	Nitrogen dioxide air pollution	456380
rs9368521	6	27679445	Nitrogen dioxide air pollution	456380
rs1084621	12	16211780	Particulate matter air pollution (pm10)	423796
rs1096116	9	13603291	Particulate matter air pollution (pm10)	423796
rs1162153	14	103577789	Particulate matter air pollution (pm10)	423796
rs1171222	9	133311860	Particulate matter air pollution (pm10)	423796
rs1176711	6	159246615	Particulate matter air pollution (pm10)	423796
rs1219295	6	94818099	Particulate matter air pollution (pm10)	423796
rs1220359	6	396321	Particulate matter air pollution (pm10)	423796
rs1257111	10	127771554	Particulate matter air pollution (pm10)	423796
rs1450455	8	342124	Particulate matter air pollution (pm10)	423796
rs1497528	2	33098355	Particulate matter air pollution (pm10)	423796
rs1509931	10	64836267	Particulate matter air pollution (pm10)	423796
rs218514	18	1469513	Particulate matter air pollution (pm10)	423796
rs2812230	13	51264767	Particulate matter air pollution (pm10)	423796
rs3477646	15	57656713	Particulate matter air pollution (pm10)	423796
rs3566526	5	51178554	Particulate matter air pollution (pm10)	423796
rs4815138	20	286487	Particulate matter air pollution (pm10)	423796
rs5704826	7	151623218	Particulate matter air pollution (pm10)	423796
rs6144718	5	101445992	Particulate matter air pollution (pm10)	423796
rs6211970	19	2279746	Particulate matter air pollution (pm10)	423796
rs7281956	5	164643843	Particulate matter air pollution (pm10)	423796
rs7320144	8	11512667	Particulate matter air pollution (pm10)	423796
rs7475651	1	115028382	Particulate matter air pollution (pm10)	423796
rs7505171	18	75220892	Particulate matter air pollution (pm10)	423796
rs7903722	13	50507971	Particulate matter air pollution (pm10)	423796
rs8023013	14	48380718	Particulate matter air pollution (pm10)	423796
rs8034341	15	68925781	Particulate matter air pollution (pm10)	423796
rs1096116	9	13603291	Particulate matter air pollution (pm2.5	423796
rs1104231	11	2051631	Particulate matter air pollution (pm2.5	423796
rs1169251	8	93266536	Particulate matter air pollution (pm2.5	423796
rs1178782	22	18475815	Particulate matter air pollution (pm2.5	423796
rs1182891	7	75245569	Particulate matter air pollution (pm2.5	423796
rs1185582	15	78008843	Particulate matter air pollution (pm2.5	423796
rs1213306	1	91214714	Particulate matter air pollution (pm2.5	423796
rs1217106	8	64567670	Particulate matter air pollution (pm2.5	423796
rs1220359	6	396321	Particulate matter air pollution (pm2.5	423796
rs1281251	12	62341242	Particulate matter air pollution (pm2.5	423796

rs131884[illegible]	4	153001662	Particulate matter air pollution (pm2.5	423796
rs137250[illegible]	5	103749428	Particulate matter air pollution (pm2.5	423796
rs153737[illegible]	9	22099568	Particulate matter air pollution (pm2.5	423796
rs169577[illegible]	16	82610053	Particulate matter air pollution (pm2.5	423796
rs171039[illegible]	14	76507299	Particulate matter air pollution (pm2.5	423796
rs176578[illegible]	7	17757841	Particulate matter air pollution (pm2.5	423796
rs214153[illegible]	7	9253413	Particulate matter air pollution (pm2.5	423796
rs229215[illegible]	16	49765133	Particulate matter air pollution (pm2.5	423796
rs27152	5	153734582	Particulate matter air pollution (pm2.5	423796
rs357035[illegible]	11	61439682	Particulate matter air pollution (pm2.5	423796
rs485452[illegible]	2	69051853	Particulate matter air pollution (pm2.5	423796
rs487230	1	55541174	Particulate matter air pollution (pm2.5	423796
rs560686[illegible]	6	50597378	Particulate matter air pollution (pm2.5	423796
rs588248[illegible]	5	137976541	Particulate matter air pollution (pm2.5	423796
rs613410[illegible]	20	11007208	Particulate matter air pollution (pm2.5	423796
rs621197[illegible]	19	2279746	Particulate matter air pollution (pm2.5	423796
rs621804[illegible]	2	200815608	Particulate matter air pollution (pm2.5	423796
rs623686[illegible]	5	80861930	Particulate matter air pollution (pm2.5	423796
rs624846[illegible]	7	104872861	Particulate matter air pollution (pm2.5	423796
rs643277[illegible]	2	164785611	Particulate matter air pollution (pm2.5	423796
rs654795[illegible]	2	22045642	Particulate matter air pollution (pm2.5	423796
rs674946[illegible]	2	343517	Particulate matter air pollution (pm2.5	423796
rs696001[illegible]	7	90302199	Particulate matter air pollution (pm2.5	423796
rs718059	14	34234984	Particulate matter air pollution (pm2.5	423796
rs726424[illegible]	18	45920421	Particulate matter air pollution (pm2.5	423796
rs726558[illegible]	13	112082676	Particulate matter air pollution (pm2.5	423796
rs727000[illegible]	14	104709808	Particulate matter air pollution (pm2.5	423796
rs728080[illegible]	5	164479164	Particulate matter air pollution (pm2.5	423796
rs751495[illegible]	1	74019696	Particulate matter air pollution (pm2.5	423796
rs757305[illegible]	2	58229428	Particulate matter air pollution (pm2.5	423796
rs772057[illegible]	8	10153460	Particulate matter air pollution (pm2.5	423796
rs772558[illegible]	6	20833602	Particulate matter air pollution (pm2.5	423796
rs777627[illegible]	6	163999770	Particulate matter air pollution (pm2.5	423796
rs785397[illegible]	12	12490981	Particulate matter air pollution (pm2.5	423796
rs785460[illegible]	10	48642432	Particulate matter air pollution (pm2.5	423796
rs801238[illegible]	3	148486829	Particulate matter air pollution (pm2.5	423796
rs8614	17	27588806	Particulate matter air pollution (pm2.5	423796
rs964448[illegible]	8	138230163	Particulate matter air pollution (pm2.5	423796
rs987204	3	94242461	Particulate matter air pollution (pm2.5	423796
rs103092[illegible]	19	8779100	Average 24-hour sound level of noise p	456380
rs117527[illegible]	8	117910936	Average 24-hour sound level of noise p	456380
rs143325[illegible]	19	32674873	Average 24-hour sound level of noise p	456380

rs1443749	7	150434689	Average 24-hour sound level of noise p	456380
rs150213	1	242381731	Average 24-hour sound level of noise p	456380
rs175904	3	25466098	Average 24-hour sound level of noise p	456380
rs189147	6	112596443	Average 24-hour sound level of noise p	456380
rs271487	7	17938491	Average 24-hour sound level of noise p	456380
rs576254	22	28666961	Average 24-hour sound level of noise p	456380
rs714785	11	102269128	Average 24-hour sound level of noise p	456380
rs724176	18	72116373	Average 24-hour sound level of noise p	456380
rs749208	13	80597508	Average 24-hour sound level of noise p	456380

Outcome	samplesize.outcon	effect allele	other allele	Beta	EAF
Coronary artery	547261	A	G	0.0150639	0.130178
Coronary artery	547261	T	G	0.00972771	0.484233
Coronary artery	547261	A	G	-0.0102936	0.416147
Coronary artery	547261	T	C	-0.009853	0.417906
Coronary artery	547261	T	C	0.0180857	0.072984
Coronary artery	547261	A	C	0.0149971	0.16794
Coronary artery	547261	A	G	0.0156804	0.154461
Coronary artery	547261	A	C	0.0124993	0.186886
Coronary artery	547261	T	C	-0.0386416	0.016208
Coronary artery	547261	G	A	-0.0110104	0.2435
Coronary artery	547261	G	A	-0.0098554	0.641994
Coronary artery	547261	A	G	-0.0132439	0.153233
Coronary artery	547261	A	G	-0.0095825	0.548637
Coronary artery	547261	G	A	0.0140134	0.782419
Coronary artery	547261	T	C	0.0196624	0.219436
Coronary artery	547261	C	T	0.00970902	0.380091
Coronary artery	547261	C	T	-0.0130453	0.200795
Coronary artery	547261	C	A	-0.0114495	0.243279
Coronary artery	547261	T	C	0.0154658	0.12919
Coronary artery	547261	A	G	0.0103554	0.373673
Coronary artery	547261	T	C	-0.0384693	0.017336
Coronary artery	547261	T	C	-0.018716	0.083064
Coronary artery	547261	G	A	0.0102993	0.698769
Coronary artery	547261	T	C	-0.0100751	0.663321
Coronary artery	547261	G	A	0.0139755	0.130449
Coronary artery	547261	C	T	0.0201622	0.064993
Coronary artery	547261	G	A	-0.0153369	0.109891
Coronary artery	547261	A	G	0.0107315	0.611738
Coronary artery	547261	T	C	0.0107705	0.33449
Coronary artery	547261	C	T	-0.0350603	0.019349
Coronary artery	547261	G	A	0.0108015	0.745173
Coronary artery	547261	T	C	0.0209748	0.079749
Coronary artery	547261	A	G	-0.0359277	0.018434
Coronary artery	547261	G	A	0.0160831	0.881571
Coronary artery	547261	C	T	0.0113964	0.239895
Coronary artery	547261	A	G	0.00969921	0.387517
Coronary artery	547261	G	T	-0.0204187	0.062116
Coronary artery	547261	T	C	-0.0113675	0.245752
Coronary artery	547261	G	T	-0.0175571	0.15565
Coronary artery	547261	C	A	-0.0300886	0.02677
Coronary artery	547261	C	A	0.0118268	0.365075

Coronary artery	547261	A	G	0.0300981	0.028151
Coronary artery	547261	T	C	-0.0113821	0.23522
Coronary artery	547261	T	C	0.0129274	0.172431
Coronary artery	547261	T	C	0.00992086	0.658049
Coronary artery	547261	T	G	-0.0207931	0.070424
Coronary artery	547261	T	C	0.0120277	0.270243
Coronary artery	547261	A	G	-0.0106399	0.46467
Coronary artery	547261	C	T	0.0134611	0.158973
Coronary artery	547261	T	C	-0.009463	0.51929
Coronary artery	547261	T	C	0.0123158	0.230477
Coronary artery	547261	G	A	0.011581	0.769457
Coronary artery	547261	T	C	0.0946143	0.003807
Coronary artery	547261	G	A	-0.0107992	0.286147
Coronary artery	547261	T	C	-0.0287129	0.029919
Coronary artery	547261	G	T	-0.0331207	0.025544
Coronary artery	547261	C	A	-0.0132066	0.186513
Coronary artery	547261	C	T	-0.0231955	0.046759
Coronary artery	547261	A	G	0.0135202	0.165706
Coronary artery	547261	G	A	0.0159377	0.106329
Coronary artery	547261	T	C	0.0148569	0.274855
Coronary artery	547261	A	G	-0.0154582	0.113504
Coronary artery	547261	T	C	-0.0098524	0.370453
Coronary artery	547261	A	G	0.0118368	0.714735
Coronary artery	547261	A	G	0.0271431	0.031972
Coronary artery	547261	C	T	-0.030081	0.027798
Coronary artery	547261	T	C	-0.0102176	0.424276
Coronary artery	547261	C	T	0.0369793	0.027316
Coronary artery	547261	T	G	-0.0176865	0.091868
Coronary artery	547261	T	C	-0.023713	0.04104
Coronary artery	547261	A	C	-0.0111924	0.368826
Coronary artery	547261	G	A	-0.0162463	0.114428
Coronary artery	547261	A	G	-0.0195322	0.085011
Coronary artery	547261	A	G	-0.0217207	0.063928
Coronary artery	547261	T	C	0.0388327	0.01703
Coronary artery	547261	T	C	-0.0420185	0.017318
Coronary artery	547261	T	C	-0.0387119	0.023291
Coronary artery	547261	T	C	0.0132343	0.212894
Coronary artery	547261	G	T	0.0118316	0.241258
Coronary artery	547261	G	A	-0.0200821	0.069722
Coronary artery	547261	C	T	-0.0287918	0.032879
Coronary artery	547261	T	C	-0.028982	0.032703
Coronary artery	547261	T	C	-0.0120578	0.230187

Coronary artery	547261	C	T	0.0460816	0.011858
Coronary artery	547261	A	G	0.027384	0.036362
Coronary artery	547261	T	C	-0.035513	0.020331
Coronary artery	547261	A	G	0.0117626	0.377409
Coronary artery	547261	C	A	-0.0124891	0.311303
Coronary artery	547261	T	C	-0.0258626	0.040057
Coronary artery	547261	T	C	0.0105209	0.657728
Coronary artery	547261	T	C	-0.0127818	0.190704
Coronary artery	547261	G	A	0.0417032	0.017923
Coronary artery	547261	C	T	-0.0494714	0.010542
Coronary artery	547261	T	G	-0.0107374	0.419106
Coronary artery	547261	A	G	-0.032334	0.028942
Coronary artery	547261	G	A	0.0420186	0.017845
Coronary artery	547261	A	G	-0.0164801	0.109299
Coronary artery	547261	A	G	-0.0193433	0.085011
Coronary artery	547261	A	G	-0.0130416	0.257614
Coronary artery	547261	G	T	0.0533208	0.010465
Coronary artery	547261	G	A	-0.0480395	0.011699
Coronary artery	547261	T	G	0.0127384	0.187616
Coronary artery	547261	A	G	-0.0126249	0.283075
Coronary artery	547261	A	C	0.0104397	0.347049
Coronary artery	547261	G	A	0.012725	0.781731
Coronary artery	547261	T	C	0.0216661	0.212894
Coronary artery	547261	G	A	0.0111409	0.287274
Coronary artery	547261	C	T	-0.0139685	0.201776
Coronary artery	547261	A	G	0.0122914	0.374311
Coronary artery	547261	A	C	0.0123705	0.500143
Coronary artery	547261	C	T	0.0216856	0.064265
Coronary artery	547261	A	C	-0.0139275	0.151277
Coronary artery	547261	C	T	-0.0371072	0.018141
Coronary artery	547261	G	T	-0.0114166	0.298539
Coronary artery	547261	T	G	0.0146809	0.167529
Coronary artery	547261	T	C	-0.0107288	0.665119
Coronary artery	547261	A	G	-0.0246758	0.049304
Coronary artery	547261	G	A	0.0134447	0.171589
Coronary artery	547261	G	A	-0.0116357	0.759827
Coronary artery	547261	T	G	-0.0181851	0.083179
Coronary artery	547261	C	A	0.0120084	0.364496
Coronary artery	547261	A	C	0.0544302	0.008242
Coronary artery	547261	T	C	0.0111347	0.657728
Coronary artery	547261	A	G	-0.0351148	0.021377
Coronary artery	547261	C	T	-0.0181174	0.082788

Coronary artery	547261	A	G	0.0178158	0.097718
Coronary artery	547261	C	T	0.0114362	0.71603
Coronary artery	547261	G	T	-0.0110872	0.645782
Coronary artery	547261	A	G	-0.0123919	0.465814
Coronary artery	547261	G	A	-0.0105236	0.339919
Coronary artery	547261	A	G	-0.0135133	0.166699
Coronary artery	547261	T	C	0.113396	0.003862
Coronary artery	547261	G	A	-0.0113177	0.286053
Coronary artery	547261	C	T	0.02003	0.074366
Coronary artery	547261	C	A	-0.0160909	0.149235
Coronary artery	547261	C	A	-0.0129805	0.186948
Coronary artery	547261	C	A	-0.0114094	0.441655
Coronary artery	547261	T	C	0.0135219	0.273909
Coronary artery	547261	T	C	0.0313937	0.036507
Coronary artery	547261	A	G	0.0109471	0.71427
Coronary artery	547261	G	A	-0.0467891	0.013105
Coronary artery	547261	C	T	-0.0335181	0.027757
Coronary artery	547261	C	T	-0.0317179	0.026758
Coronary artery	547261	T	G	-0.0187398	0.092425
Coronary artery	547261	A	C	0.0130804	0.182153
Coronary artery	547261	A	G	0.0139827	0.151287
Coronary artery	547261	C	T	0.00986771	0.478622
Coronary artery	547261	A	G	0.0102987	0.437758
Coronary artery	547261	C	T	0.00990463	0.425557
Coronary artery	547261	A	G	0.0573849	0.008598
Coronary artery	547261	T	C	-0.0355946	0.018977
Coronary artery	547261	A	G	-0.0405505	0.015591
Coronary artery	547261	A	C	-0.0107806	0.286782
Coronary artery	547261	G	A	0.0289547	0.030344
Coronary artery	547261	T	C	0.0096854	0.565309
Coronary artery	547261	C	T	0.0101453	0.33166
Coronary artery	547261	C	T	-0.0104559	0.327404
Coronary artery	547261	G	A	-0.0268806	0.034508
Coronary artery	547261	T	C	-0.0098819	0.455668
Coronary artery	547261	A	G	0.0113885	0.285878
Atrial fibrillation	1030836	A	G	0.0150639	0.130178
Atrial fibrillation	1030836	T	G	0.00972771	0.484233
Atrial fibrillation	1030836	A	G	-0.0102936	0.416147
Atrial fibrillation	1030836	T	C	-0.009853	0.417906
Atrial fibrillation	1030836	T	C	0.0180857	0.072984
Atrial fibrillation	1030836	A	C	0.0149971	0.16794
Atrial fibrillation	1030836	A	G	0.0156804	0.154461

Atrial fibrillatio	1030836	A	C	0.0124993	0.186886
Atrial fibrillatio	1030836	T	C	-0.0386416	0.016208
Atrial fibrillatio	1030836	G	A	-0.0110104	0.2435
Atrial fibrillatio	1030836	G	A	-0.0098554	0.641994
Atrial fibrillatio	1030836	A	G	0.0867972	0.003383
Atrial fibrillatio	1030836	A	G	-0.0132439	0.153233
Atrial fibrillatio	1030836	A	G	-0.0095825	0.548637
Atrial fibrillatio	1030836	G	A	0.0140134	0.782419
Atrial fibrillatio	1030836	T	C	0.0196624	0.219436
Atrial fibrillatio	1030836	C	T	0.00970902	0.380091
Atrial fibrillatio	1030836	C	T	-0.0130453	0.200795
Atrial fibrillatio	1030836	C	A	-0.0114495	0.243279
Atrial fibrillatio	1030836	T	C	0.0154658	0.12919
Atrial fibrillatio	1030836	A	G	0.0103554	0.373673
Atrial fibrillatio	1030836	T	C	-0.0384693	0.017336
Atrial fibrillatio	1030836	G	T	-0.0429541	0.013342
Atrial fibrillatio	1030836	T	C	-0.018716	0.083064
Atrial fibrillatio	1030836	G	A	0.0102993	0.698769
Atrial fibrillatio	1030836	T	C	-0.0100751	0.663321
Atrial fibrillatio	1030836	G	A	0.0139755	0.130449
Atrial fibrillatio	1030836	C	T	0.0201622	0.064993
Atrial fibrillatio	1030836	G	A	-0.0153369	0.109891
Atrial fibrillatio	1030836	A	G	0.0107315	0.611738
Atrial fibrillatio	1030836	T	C	0.0107705	0.33449
Atrial fibrillatio	1030836	C	T	-0.0350603	0.019349
Atrial fibrillatio	1030836	G	A	0.0108015	0.745173
Atrial fibrillatio	1030836	T	C	0.0209748	0.079749
Atrial fibrillatio	1030836	A	G	-0.0359277	0.018434
Atrial fibrillatio	1030836	G	A	0.0160831	0.881571
Atrial fibrillatio	1030836	C	T	0.0113964	0.239895
Atrial fibrillatio	1030836	A	G	0.00969921	0.387517
Atrial fibrillatio	1030836	G	T	-0.0204187	0.062116
Atrial fibrillatio	1030836	T	C	-0.0113675	0.245752
Atrial fibrillatio	1030836	G	T	-0.0175571	0.15565
Atrial fibrillatio	1030836	C	A	-0.0300886	0.02677
Atrial fibrillatio	1030836	C	A	0.0118268	0.365075
Atrial fibrillatio	1030836	A	G	0.0300981	0.028151
Atrial fibrillatio	1030836	T	C	-0.0113821	0.23522
Atrial fibrillatio	1030836	T	C	0.0129274	0.172431
Atrial fibrillatio	1030836	T	C	0.00992086	0.658049
Atrial fibrillatio	1030836	T	G	-0.0207931	0.070424
Atrial fibrillatio	1030836	T	C	0.0120277	0.270243

Atrial fibrillation	1030836	A	G	-0.0106399	0.46467
Atrial fibrillation	1030836	C	T	0.0134611	0.158973
Atrial fibrillation	1030836	T	C	-0.009463	0.51929
Atrial fibrillation	1030836	T	C	0.0123158	0.230477
Atrial fibrillation	1030836	G	A	0.011581	0.769457
Atrial fibrillation	1030836	T	C	0.0946143	0.003807
Atrial fibrillation	1030836	G	A	-0.0107992	0.286147
Atrial fibrillation	1030836	T	C	-0.0287129	0.029919
Atrial fibrillation	1030836	G	T	-0.0331207	0.025544
Atrial fibrillation	1030836	C	A	-0.0132066	0.186513
Atrial fibrillation	1030836	C	T	-0.0231955	0.046759
Atrial fibrillation	1030836	A	G	0.0135202	0.165706
Atrial fibrillation	1030836	G	A	0.0159377	0.106329
Atrial fibrillation	1030836	T	C	0.0148569	0.274855
Atrial fibrillation	1030836	A	G	-0.0154582	0.113504
Atrial fibrillation	1030836	T	C	-0.0098524	0.370453
Atrial fibrillation	1030836	A	G	0.0118368	0.714735
Atrial fibrillation	1030836	A	G	0.0271431	0.031972
Atrial fibrillation	1030836	C	T	-0.030081	0.027798
Atrial fibrillation	1030836	T	C	-0.0102176	0.424276
Atrial fibrillation	1030836	C	T	0.0369793	0.027316
Atrial fibrillation	1030836	T	G	-0.0176865	0.091868
Atrial fibrillation	1030836	T	C	-0.023713	0.04104
Atrial fibrillation	1030836	A	C	-0.0111924	0.368826
Atrial fibrillation	1030836	A	G	-0.0193433	0.085011
Atrial fibrillation	1030836	A	G	-0.0130416	0.257614
Atrial fibrillation	1030836	G	T	0.0533208	0.010465
Atrial fibrillation	1030836	G	A	-0.0480395	0.011699
Atrial fibrillation	1030836	T	G	0.0127384	0.187616
Atrial fibrillation	1030836	A	G	-0.0126249	0.283075
Atrial fibrillation	1030836	A	C	0.0104397	0.347049
Atrial fibrillation	1030836	G	A	0.012725	0.781731
Atrial fibrillation	1030836	T	C	0.0216661	0.212894
Atrial fibrillation	1030836	G	A	0.0111409	0.287274
Atrial fibrillation	1030836	C	T	-0.0139685	0.201776
Atrial fibrillation	1030836	A	G	0.0122914	0.374311
Atrial fibrillation	1030836	A	C	0.0123705	0.500143
Atrial fibrillation	1030836	C	T	0.0216856	0.064265
Atrial fibrillation	1030836	A	C	-0.0139275	0.151277
Atrial fibrillation	1030836	G	T	-0.0434289	0.013488
Atrial fibrillation	1030836	C	T	-0.0371072	0.018141
Atrial fibrillation	1030836	G	T	-0.0114166	0.298539

Atrial fibrillatio	1030836	T	G	0.0146809	0.167529
Atrial fibrillatio	1030836	T	C	-0.0107288	0.665119
Atrial fibrillatio	1030836	A	G	-0.0246758	0.049304
Atrial fibrillatio	1030836	G	A	0.0134447	0.171589
Atrial fibrillatio	1030836	G	A	-0.0116357	0.759827
Atrial fibrillatio	1030836	T	G	-0.0181851	0.083179
Atrial fibrillatio	1030836	C	A	0.0120084	0.364496
Atrial fibrillatio	1030836	A	C	0.0544302	0.008242
Atrial fibrillatio	1030836	T	C	0.0111347	0.657728
Atrial fibrillatio	1030836	A	G	-0.0351148	0.021377
Atrial fibrillatio	1030836	C	T	-0.0181174	0.082788
Atrial fibrillatio	1030836	A	G	0.0178158	0.097718
Atrial fibrillatio	1030836	C	T	0.0114362	0.71603
Atrial fibrillatio	1030836	G	T	-0.0110872	0.645782
Atrial fibrillatio	1030836	A	G	-0.0123919	0.465814
Atrial fibrillatio	1030836	G	A	-0.0105236	0.339919
Atrial fibrillatio	1030836	A	G	-0.0135133	0.166699
Atrial fibrillatio	1030836	T	C	0.113396	0.003862
Atrial fibrillatio	1030836	G	A	-0.0113177	0.286053
Atrial fibrillatio	1030836	C	T	0.02003	0.074366
Atrial fibrillatio	1030836	C	A	-0.0160909	0.149235
Atrial fibrillatio	1030836	C	A	-0.0129805	0.186948
Atrial fibrillatio	1030836	C	A	-0.0114094	0.441655
Atrial fibrillatio	1030836	T	C	0.0135219	0.273909
Atrial fibrillatio	1030836	T	C	0.0313937	0.036507
Atrial fibrillatio	1030836	A	G	0.0109471	0.71427
Atrial fibrillatio	1030836	G	A	-0.0467891	0.013105
Atrial fibrillatio	1030836	C	T	-0.0335181	0.027757
Atrial fibrillatio	1030836	C	T	-0.0317179	0.026758
Atrial fibrillatio	1030836	T	G	-0.0187398	0.092425
Atrial fibrillatio	1030836	A	C	0.0130804	0.182153
Atrial fibrillatio	1030836	A	G	0.0139827	0.151287
Atrial fibrillatio	1030836	C	T	0.00986771	0.478622
Atrial fibrillatio	1030836	A	G	0.0102987	0.437758
Atrial fibrillatio	1030836	G	A	-0.0162463	0.114428
Atrial fibrillatio	1030836	A	G	-0.0195322	0.085011
Atrial fibrillatio	1030836	A	G	-0.0217207	0.063928
Atrial fibrillatio	1030836	T	C	0.0388327	0.01703
Atrial fibrillatio	1030836	T	C	-0.0420185	0.017318
Atrial fibrillatio	1030836	T	C	-0.0387119	0.023291
Atrial fibrillatio	1030836	T	C	0.0132343	0.212894
Atrial fibrillatio	1030836	G	T	0.0118316	0.241258

Atrial fibrillation	1030836	G	A	-0.0200821	0.069722
Atrial fibrillation	1030836	C	T	-0.0581919	0.010194
Atrial fibrillation	1030836	C	T	-0.0287918	0.032879
Atrial fibrillation	1030836	T	C	-0.028982	0.032703
Atrial fibrillation	1030836	T	C	-0.0120578	0.230187
Atrial fibrillation	1030836	C	T	0.0460816	0.011858
Atrial fibrillation	1030836	A	G	0.027384	0.036362
Atrial fibrillation	1030836	T	C	-0.035513	0.020331
Atrial fibrillation	1030836	A	G	0.0117626	0.377409
Atrial fibrillation	1030836	C	A	-0.0124891	0.311303
Atrial fibrillation	1030836	T	C	-0.0258626	0.040057
Atrial fibrillation	1030836	T	C	0.0105209	0.657728
Atrial fibrillation	1030836	T	C	-0.0127818	0.190704
Atrial fibrillation	1030836	G	A	0.0417032	0.017923
Atrial fibrillation	1030836	C	T	-0.0494714	0.010542
Atrial fibrillation	1030836	T	G	-0.0107374	0.419106
Atrial fibrillation	1030836	A	G	-0.032334	0.028942
Atrial fibrillation	1030836	G	A	0.0420186	0.017845
Atrial fibrillation	1030836	A	G	-0.0164801	0.109299
Atrial fibrillation	1030836	C	T	0.00990463	0.425557
Atrial fibrillation	1030836	A	G	0.0573849	0.008598
Atrial fibrillation	1030836	T	C	-0.0355946	0.018977
Atrial fibrillation	1030836	A	G	-0.0405505	0.015591
Atrial fibrillation	1030836	A	C	-0.0107806	0.286782
Atrial fibrillation	1030836	G	A	0.0289547	0.030344
Atrial fibrillation	1030836	T	C	0.0096854	0.565309
Atrial fibrillation	1030836	C	T	0.0101453	0.33166
Atrial fibrillation	1030836	C	T	-0.0104559	0.327404
Atrial fibrillation	1030836	G	A	-0.0268806	0.034508
Atrial fibrillation	1030836	T	C	-0.0098819	0.455668
Atrial fibrillation	1030836	A	G	0.0113885	0.285878
Heart failure	977323	A	G	-0.0193433	0.085011
Heart failure	977323	A	G	-0.0130416	0.257614
Heart failure	977323	T	G	0.0127384	0.187616
Heart failure	977323	A	G	-0.0126249	0.283075
Heart failure	977323	A	C	0.0104397	0.347049
Heart failure	977323	G	A	0.012725	0.781731
Heart failure	977323	T	C	0.0216661	0.212894
Heart failure	977323	G	A	0.0111409	0.287274
Heart failure	977323	C	T	-0.0139685	0.201776
Heart failure	977323	A	G	0.0122914	0.374311
Heart failure	977323	A	C	0.0123705	0.500143

Heart failure	977323	C	T	0.0216856	0.064265
Heart failure	977323	A	C	-0.0139275	0.151277
Heart failure	977323	G	T	-0.0434289	0.013488
Heart failure	977323	C	T	-0.0371072	0.018141
Heart failure	977323	G	T	-0.0114166	0.298539
Heart failure	977323	T	G	0.0146809	0.167529
Heart failure	977323	T	C	-0.0107288	0.665119
Heart failure	977323	A	G	-0.0246758	0.049304
Heart failure	977323	G	A	0.0134447	0.171589
Heart failure	977323	G	A	-0.0116357	0.759827
Heart failure	977323	T	G	-0.0181851	0.083179
Heart failure	977323	C	A	0.0120084	0.364496
Heart failure	977323	T	C	0.0111347	0.657728
Heart failure	977323	A	G	-0.0351148	0.021377
Heart failure	977323	C	T	-0.0181174	0.082788
Heart failure	977323	A	G	0.0178158	0.097718
Heart failure	977323	C	T	0.0114362	0.71603
Heart failure	977323	G	T	-0.0110872	0.645782
Heart failure	977323	A	G	-0.0123919	0.465814
Heart failure	977323	G	A	-0.0105236	0.339919
Heart failure	977323	A	G	-0.0135133	0.166699
Heart failure	977323	T	C	0.113396	0.003862
Heart failure	977323	G	A	-0.0113177	0.286053
Heart failure	977323	C	T	0.02003	0.074366
Heart failure	977323	C	A	-0.0160909	0.149235
Heart failure	977323	C	A	-0.0129805	0.186948
Heart failure	977323	C	A	-0.0114094	0.441655
Heart failure	977323	T	C	0.0135219	0.273909
Heart failure	977323	T	C	0.0313937	0.036507
Heart failure	977323	A	G	0.0109471	0.71427
Heart failure	977323	C	T	-0.0335181	0.027757
Heart failure	977323	T	G	-0.0187398	0.092425
Heart failure	977323	A	C	0.0130804	0.182153
Heart failure	977323	A	G	0.0139827	0.151287
Heart failure	977323	C	T	0.00986771	0.478622
Heart failure	977323	A	G	0.0102987	0.437758
Heart failure	977323	A	G	0.0150639	0.130178
Heart failure	977323	T	G	0.00972771	0.484233
Heart failure	977323	A	G	-0.0102936	0.416147
Heart failure	977323	T	C	-0.009853	0.417906
Heart failure	977323	T	C	0.0180857	0.072984
Heart failure	977323	A	C	0.0149971	0.16794

Heart failure	977323	A	G	0.0156804	0.154461
Heart failure	977323	A	C	0.0124993	0.186886
Heart failure	977323	T	C	-0.0386416	0.016208
Heart failure	977323	G	A	-0.0110104	0.2435
Heart failure	977323	G	A	-0.0098554	0.641994
Heart failure	977323	A	G	-0.0132439	0.153233
Heart failure	977323	A	G	-0.0095825	0.548637
Heart failure	977323	G	A	0.0140134	0.782419
Heart failure	977323	T	C	0.0196624	0.219436
Heart failure	977323	C	T	0.00970902	0.380091
Heart failure	977323	C	T	-0.0130453	0.200795
Heart failure	977323	C	A	-0.0114495	0.243279
Heart failure	977323	T	C	0.0154658	0.12919
Heart failure	977323	A	G	0.0103554	0.373673
Heart failure	977323	T	C	-0.0384693	0.017336
Heart failure	977323	G	T	-0.0429541	0.013342
Heart failure	977323	T	C	-0.018716	0.083064
Heart failure	977323	G	A	0.0102993	0.698769
Heart failure	977323	T	C	-0.0100751	0.663321
Heart failure	977323	G	A	0.0139755	0.130449
Heart failure	977323	C	T	0.0201622	0.064993
Heart failure	977323	G	A	-0.0153369	0.109891
Heart failure	977323	A	G	0.0107315	0.611738
Heart failure	977323	T	C	0.0107705	0.33449
Heart failure	977323	C	T	-0.0350603	0.019349
Heart failure	977323	G	A	0.0108015	0.745173
Heart failure	977323	T	C	0.0209748	0.079749
Heart failure	977323	A	G	-0.0359277	0.018434
Heart failure	977323	G	A	0.0160831	0.881571
Heart failure	977323	C	T	0.0113964	0.239895
Heart failure	977323	A	G	0.00969921	0.387517
Heart failure	977323	G	T	-0.0204187	0.062116
Heart failure	977323	T	C	-0.0113675	0.245752
Heart failure	977323	G	T	-0.0175571	0.15565
Heart failure	977323	C	A	-0.0300886	0.02677
Heart failure	977323	C	A	0.0118268	0.365075
Heart failure	977323	A	G	0.0300981	0.028151
Heart failure	977323	T	C	-0.0113821	0.23522
Heart failure	977323	T	C	0.0129274	0.172431
Heart failure	977323	T	C	0.00992086	0.658049
Heart failure	977323	T	G	-0.0207931	0.070424
Heart failure	977323	T	C	0.0120277	0.270243

Heart failure	977323	A	G	-0.0106399	0.46467
Heart failure	977323	C	T	0.0134611	0.158973
Heart failure	977323	T	C	-0.009463	0.51929
Heart failure	977323	T	C	0.0123158	0.230477
Heart failure	977323	G	A	0.011581	0.769457
Heart failure	977323	T	C	0.0946143	0.003807
Heart failure	977323	G	A	-0.0107992	0.286147
Heart failure	977323	T	C	-0.0287129	0.029919
Heart failure	977323	G	T	-0.0331207	0.025544
Heart failure	977323	C	A	-0.0132066	0.186513
Heart failure	977323	C	T	-0.0231955	0.046759
Heart failure	977323	A	G	0.0135202	0.165706
Heart failure	977323	G	A	0.0159377	0.106329
Heart failure	977323	T	C	0.0148569	0.274855
Heart failure	977323	A	G	-0.0154582	0.113504
Heart failure	977323	T	C	-0.0098524	0.370453
Heart failure	977323	A	G	0.0118368	0.714735
Heart failure	977323	A	G	0.0271431	0.031972
Heart failure	977323	C	T	-0.030081	0.027798
Heart failure	977323	T	C	-0.0102176	0.424276
Heart failure	977323	C	T	0.0369793	0.027316
Heart failure	977323	T	G	-0.0176865	0.091868
Heart failure	977323	T	C	-0.023713	0.04104
Heart failure	977323	A	C	-0.0111924	0.368826
Heart failure	977323	C	T	0.00990463	0.425557
Heart failure	977323	T	C	-0.0355946	0.018977
Heart failure	977323	A	G	-0.0405505	0.015591
Heart failure	977323	A	C	-0.0107806	0.286782
Heart failure	977323	G	A	0.0289547	0.030344
Heart failure	977323	T	C	0.0096854	0.565309
Heart failure	977323	C	T	0.0101453	0.33166
Heart failure	977323	C	T	-0.0104559	0.327404
Heart failure	977323	G	A	-0.0268806	0.034508
Heart failure	977323	T	C	-0.0098819	0.455668
Heart failure	977323	A	G	0.0113885	0.285878
Heart failure	977323	G	A	-0.0162463	0.114428
Heart failure	977323	A	G	-0.0195322	0.085011
Heart failure	977323	A	G	-0.0217207	0.063928
Heart failure	977323	T	C	0.0388327	0.01703
Heart failure	977323	T	C	-0.0420185	0.017318
Heart failure	977323	T	C	-0.0387119	0.023291
Heart failure	977323	T	C	0.0132343	0.212894

Heart failure	977323	G	T	0.0118316	0.241258
Heart failure	977323	G	A	-0.0200821	0.069722
Heart failure	977323	C	T	-0.0287918	0.032879
Heart failure	977323	T	C	-0.028982	0.032703
Heart failure	977323	T	C	-0.0120578	0.230187
Heart failure	977323	C	T	0.0460816	0.011858
Heart failure	977323	A	G	0.027384	0.036362
Heart failure	977323	T	C	-0.035513	0.020331
Heart failure	977323	A	G	0.0117626	0.377409
Heart failure	977323	C	A	-0.0124891	0.311303
Heart failure	977323	T	C	-0.0258626	0.040057
Heart failure	977323	T	C	0.0105209	0.657728
Heart failure	977323	T	C	-0.0127818	0.190704
Heart failure	977323	G	A	0.0417032	0.017923
Heart failure	977323	T	G	-0.0107374	0.419106
Heart failure	977323	A	G	-0.032334	0.028942
Heart failure	977323	G	A	0.0420186	0.017845
Heart failure	977323	A	G	-0.0164801	0.109299
Cardiac arrhytm	172155	G	A	-0.0162463	0.114428
Cardiac arrhytm	172155	A	G	-0.0195322	0.085011
Cardiac arrhytm	172155	A	G	-0.0217207	0.063928
Cardiac arrhytm	172155	T	C	0.0388327	0.01703
Cardiac arrhytm	172155	T	C	-0.0420185	0.017318
Cardiac arrhytm	172155	T	C	-0.0387119	0.023291
Cardiac arrhytm	172155	T	C	0.0132343	0.212894
Cardiac arrhytm	172155	G	T	0.0118316	0.241258
Cardiac arrhytm	172155	G	A	-0.0200821	0.069722
Cardiac arrhytm	172155	C	T	-0.0581919	0.010194
Cardiac arrhytm	172155	C	T	-0.0287918	0.032879
Cardiac arrhytm	172155	T	C	-0.028982	0.032703
Cardiac arrhytm	172155	T	C	-0.0120578	0.230187
Cardiac arrhytm	172155	C	T	0.0460816	0.011858
Cardiac arrhytm	172155	A	G	0.027384	0.036362
Cardiac arrhytm	172155	T	C	-0.035513	0.020331
Cardiac arrhytm	172155	A	G	0.0117626	0.377409
Cardiac arrhytm	172155	C	A	-0.0124891	0.311303
Cardiac arrhytm	172155	T	C	-0.0258626	0.040057
Cardiac arrhytm	172155	T	C	0.0105209	0.657728
Cardiac arrhytm	172155	T	C	-0.0127818	0.190704
Cardiac arrhytm	172155	G	A	0.0417032	0.017923
Cardiac arrhytm	172155	C	T	-0.0494714	0.010542
Cardiac arrhytm	172155	T	G	-0.0107374	0.419106

Cardiac arrhytm	172155	A	G	-0.032334	0.028942
Cardiac arrhytm	172155	G	A	0.0420186	0.017845
Cardiac arrhytm	172155	A	G	-0.0164801	0.109299
Cardiac arrhytm	172155	A	G	0.0150639	0.130178
Cardiac arrhytm	172155	T	G	0.00972771	0.484233
Cardiac arrhytm	172155	A	G	-0.0102936	0.416147
Cardiac arrhytm	172155	T	C	-0.009853	0.417906
Cardiac arrhytm	172155	T	C	0.0180857	0.072984
Cardiac arrhytm	172155	A	C	0.0149971	0.16794
Cardiac arrhytm	172155	A	G	0.0156804	0.154461
Cardiac arrhytm	172155	A	C	0.0124993	0.186886
Cardiac arrhytm	172155	T	C	-0.0386416	0.016208
Cardiac arrhytm	172155	G	A	-0.0110104	0.2435
Cardiac arrhytm	172155	G	A	-0.0098554	0.641994
Cardiac arrhytm	172155	A	G	0.0867972	0.003383
Cardiac arrhytm	172155	A	G	-0.0132439	0.153233
Cardiac arrhytm	172155	A	G	-0.0095825	0.548637
Cardiac arrhytm	172155	G	A	0.0140134	0.782419
Cardiac arrhytm	172155	T	C	0.0196624	0.219436
Cardiac arrhytm	172155	C	T	0.00970902	0.380091
Cardiac arrhytm	172155	C	T	-0.0130453	0.200795
Cardiac arrhytm	172155	C	A	-0.0114495	0.243279
Cardiac arrhytm	172155	T	C	0.0154658	0.12919
Cardiac arrhytm	172155	A	G	0.0103554	0.373673
Cardiac arrhytm	172155	T	C	-0.0384693	0.017336
Cardiac arrhytm	172155	G	T	-0.0429541	0.013342
Cardiac arrhytm	172155	T	C	-0.018716	0.083064
Cardiac arrhytm	172155	G	A	0.0102993	0.698769
Cardiac arrhytm	172155	T	C	-0.0100751	0.663321
Cardiac arrhytm	172155	G	A	0.0139755	0.130449
Cardiac arrhytm	172155	C	T	0.0201622	0.064993
Cardiac arrhytm	172155	G	A	-0.0153369	0.109891
Cardiac arrhytm	172155	A	G	0.0107315	0.611738
Cardiac arrhytm	172155	T	C	0.0107705	0.33449
Cardiac arrhytm	172155	C	T	-0.0350603	0.019349
Cardiac arrhytm	172155	G	A	0.0108015	0.745173
Cardiac arrhytm	172155	T	C	0.0209748	0.079749
Cardiac arrhytm	172155	A	G	-0.0359277	0.018434
Cardiac arrhytm	172155	G	A	0.0160831	0.881571
Cardiac arrhytm	172155	C	T	0.0113964	0.239895
Cardiac arrhytm	172155	A	G	0.00969921	0.387517
Cardiac arrhytm	172155	G	T	-0.0204187	0.062116

Cardiac arrhytm	172155	T	C	-0.0113675	0.245752
Cardiac arrhytm	172155	G	T	-0.0175571	0.15565
Cardiac arrhytm	172155	C	A	-0.0300886	0.02677
Cardiac arrhytm	172155	C	A	0.0118268	0.365075
Cardiac arrhytm	172155	A	G	0.0300981	0.028151
Cardiac arrhytm	172155	T	C	-0.0113821	0.23522
Cardiac arrhytm	172155	T	C	0.0129274	0.172431
Cardiac arrhytm	172155	T	C	0.00992086	0.658049
Cardiac arrhytm	172155	T	G	-0.0207931	0.070424
Cardiac arrhytm	172155	A	G	-0.0106399	0.46467
Cardiac arrhytm	172155	C	T	0.0134611	0.158973
Cardiac arrhytm	172155	T	C	-0.009463	0.51929
Cardiac arrhytm	172155	T	C	0.0123158	0.230477
Cardiac arrhytm	172155	G	A	0.011581	0.769457
Cardiac arrhytm	172155	T	C	0.0946143	0.003807
Cardiac arrhytm	172155	G	A	-0.0107992	0.286147
Cardiac arrhytm	172155	T	C	-0.0287129	0.029919
Cardiac arrhytm	172155	G	T	-0.0331207	0.025544
Cardiac arrhytm	172155	C	A	-0.0132066	0.186513
Cardiac arrhytm	172155	C	T	-0.0231955	0.046759
Cardiac arrhytm	172155	A	G	0.0135202	0.165706
Cardiac arrhytm	172155	G	A	0.0159377	0.106329
Cardiac arrhytm	172155	T	C	0.0148569	0.274855
Cardiac arrhytm	172155	A	G	-0.0154582	0.113504
Cardiac arrhytm	172155	T	C	-0.0098524	0.370453
Cardiac arrhytm	172155	A	G	0.0118368	0.714735
Cardiac arrhytm	172155	A	G	0.0271431	0.031972
Cardiac arrhytm	172155	C	T	-0.030081	0.027798
Cardiac arrhytm	172155	T	C	-0.0102176	0.424276
Cardiac arrhytm	172155	T	G	-0.0176865	0.091868
Cardiac arrhytm	172155	T	C	-0.023713	0.04104
Cardiac arrhytm	172155	A	C	-0.0111924	0.368826
Cardiac arrhytm	172155	A	G	-0.0193433	0.085011
Cardiac arrhytm	172155	G	T	0.0533208	0.010465
Cardiac arrhytm	172155	G	A	-0.0480395	0.011699
Cardiac arrhytm	172155	T	G	0.0127384	0.187616
Cardiac arrhytm	172155	A	G	-0.0126249	0.283075
Cardiac arrhytm	172155	A	C	0.0104397	0.347049
Cardiac arrhytm	172155	G	A	0.012725	0.781731
Cardiac arrhytm	172155	T	C	0.0216661	0.212894
Cardiac arrhytm	172155	C	T	-0.0139685	0.201776
Cardiac arrhytm	172155	A	G	0.0122914	0.374311

Cardiac arrhytm	172155	A	C	0.0123705	0.500143
Cardiac arrhytm	172155	C	T	0.0216856	0.064265
Cardiac arrhytm	172155	A	C	-0.0139275	0.151277
Cardiac arrhytm	172155	G	T	-0.0434289	0.013488
Cardiac arrhytm	172155	C	T	-0.0371072	0.018141
Cardiac arrhytm	172155	G	T	-0.0114166	0.298539
Cardiac arrhytm	172155	T	G	0.0146809	0.167529
Cardiac arrhytm	172155	T	C	-0.0107288	0.665119
Cardiac arrhytm	172155	A	G	-0.0246758	0.049304
Cardiac arrhytm	172155	G	A	0.0134447	0.171589
Cardiac arrhytm	172155	G	A	-0.0116357	0.759827
Cardiac arrhytm	172155	T	G	-0.0181851	0.083179
Cardiac arrhytm	172155	C	A	0.0120084	0.364496
Cardiac arrhytm	172155	A	C	0.0544302	0.008242
Cardiac arrhytm	172155	T	C	0.0111347	0.657728
Cardiac arrhytm	172155	A	G	-0.0351148	0.021377
Cardiac arrhytm	172155	C	T	-0.0181174	0.082788
Cardiac arrhytm	172155	A	G	0.0178158	0.097718
Cardiac arrhytm	172155	C	T	0.0114362	0.71603
Cardiac arrhytm	172155	G	T	-0.0110872	0.645782
Cardiac arrhytm	172155	A	G	-0.0123919	0.465814
Cardiac arrhytm	172155	G	A	-0.0105236	0.339919
Cardiac arrhytm	172155	A	G	-0.0135133	0.166699
Cardiac arrhytm	172155	T	C	0.113396	0.003862
Cardiac arrhytm	172155	G	A	-0.0113177	0.286053
Cardiac arrhytm	172155	C	T	0.02003	0.074366
Cardiac arrhytm	172155	C	A	-0.0160909	0.149235
Cardiac arrhytm	172155	C	A	-0.0129805	0.186948
Cardiac arrhytm	172155	C	A	-0.0114094	0.441655
Cardiac arrhytm	172155	T	C	0.0135219	0.273909
Cardiac arrhytm	172155	T	C	0.0313937	0.036507
Cardiac arrhytm	172155	A	G	0.0109471	0.71427
Cardiac arrhytm	172155	G	A	-0.0467891	0.013105
Cardiac arrhytm	172155	C	T	-0.0335181	0.027757
Cardiac arrhytm	172155	T	G	-0.0187398	0.092425
Cardiac arrhytm	172155	A	C	0.0130804	0.182153
Cardiac arrhytm	172155	A	G	0.0139827	0.151287
Cardiac arrhytm	172155	C	T	0.00986771	0.478622
Cardiac arrhytm	172155	A	G	0.0102987	0.437758
Cardiac arrhytm	172155	C	T	0.00990463	0.425557
Cardiac arrhytm	172155	A	G	0.0573849	0.008598
Cardiac arrhytm	172155	T	C	-0.0355946	0.018977

Cardiac arrhytm	172155	A	G	-0.0405505	0.015591
Cardiac arrhytm	172155	A	C	-0.0107806	0.286782
Cardiac arrhytm	172155	G	A	0.0289547	0.030344
Cardiac arrhytm	172155	T	C	0.0096854	0.565309
Cardiac arrhytm	172155	C	T	0.0101453	0.33166
Cardiac arrhytm	172155	C	T	-0.0104559	0.327404
Cardiac arrhytm	172155	G	A	-0.0268806	0.034508
Cardiac arrhytm	172155	T	C	-0.0098819	0.455668
Cardiac arrhytm	172155	A	G	0.0113885	0.285878
Cardiomyopath	159811	A	G	0.0150639	0.130178
Cardiomyopath	159811	T	G	0.00972771	0.484233
Cardiomyopath	159811	A	G	-0.0102936	0.416147
Cardiomyopath	159811	T	C	-0.009853	0.417906
Cardiomyopath	159811	T	C	0.0180857	0.072984
Cardiomyopath	159811	A	C	0.0149971	0.16794
Cardiomyopath	159811	A	G	0.0156804	0.154461
Cardiomyopath	159811	A	C	0.0124993	0.186886
Cardiomyopath	159811	T	C	-0.0386416	0.016208
Cardiomyopath	159811	G	A	-0.0110104	0.2435
Cardiomyopath	159811	G	A	-0.0098554	0.641994
Cardiomyopath	159811	A	G	0.0867972	0.003383
Cardiomyopath	159811	A	G	-0.0132439	0.153233
Cardiomyopath	159811	A	G	-0.0095825	0.548637
Cardiomyopath	159811	G	A	0.0140134	0.782419
Cardiomyopath	159811	T	C	0.0196624	0.219436
Cardiomyopath	159811	C	T	0.00970902	0.380091
Cardiomyopath	159811	C	T	-0.0130453	0.200795
Cardiomyopath	159811	C	A	-0.0114495	0.243279
Cardiomyopath	159811	T	C	0.0154658	0.12919
Cardiomyopath	159811	A	G	0.0103554	0.373673
Cardiomyopath	159811	T	C	-0.0384693	0.017336
Cardiomyopath	159811	G	T	-0.0429541	0.013342
Cardiomyopath	159811	T	C	-0.018716	0.083064
Cardiomyopath	159811	G	A	0.0102993	0.698769
Cardiomyopath	159811	T	C	-0.0100751	0.663321
Cardiomyopath	159811	G	A	0.0139755	0.130449
Cardiomyopath	159811	C	T	0.0201622	0.064993
Cardiomyopath	159811	G	A	-0.0153369	0.109891
Cardiomyopath	159811	A	G	0.0107315	0.611738
Cardiomyopath	159811	T	C	0.0107705	0.33449
Cardiomyopath	159811	C	T	-0.0350603	0.019349
Cardiomyopath	159811	G	A	0.0108015	0.745173

Cardiomyopath	159811	T	C	0.0209748	0.079749
Cardiomyopath	159811	A	G	-0.0359277	0.018434
Cardiomyopath	159811	G	A	0.0160831	0.881571
Cardiomyopath	159811	C	T	0.0113964	0.239895
Cardiomyopath	159811	A	G	0.00969921	0.387517
Cardiomyopath	159811	G	T	-0.0204187	0.062116
Cardiomyopath	159811	T	C	-0.0113675	0.245752
Cardiomyopath	159811	G	T	-0.0175571	0.15565
Cardiomyopath	159811	C	A	-0.0300886	0.02677
Cardiomyopath	159811	C	A	0.0118268	0.365075
Cardiomyopath	159811	A	G	0.0300981	0.028151
Cardiomyopath	159811	T	C	-0.0113821	0.23522
Cardiomyopath	159811	T	C	0.0129274	0.172431
Cardiomyopath	159811	T	C	0.00992086	0.658049
Cardiomyopath	159811	T	G	-0.0207931	0.070424
Cardiomyopath	159811	A	G	-0.0106399	0.46467
Cardiomyopath	159811	C	T	0.0134611	0.158973
Cardiomyopath	159811	T	C	-0.009463	0.51929
Cardiomyopath	159811	T	C	0.0123158	0.230477
Cardiomyopath	159811	G	A	0.011581	0.769457
Cardiomyopath	159811	T	C	0.0946143	0.003807
Cardiomyopath	159811	G	A	-0.0107992	0.286147
Cardiomyopath	159811	T	C	-0.0287129	0.029919
Cardiomyopath	159811	G	T	-0.0331207	0.025544
Cardiomyopath	159811	C	A	-0.0132066	0.186513
Cardiomyopath	159811	C	T	-0.0231955	0.046759
Cardiomyopath	159811	A	G	0.0135202	0.165706
Cardiomyopath	159811	G	A	0.0159377	0.106329
Cardiomyopath	159811	T	C	0.0148569	0.274855
Cardiomyopath	159811	A	G	-0.0154582	0.113504
Cardiomyopath	159811	T	C	-0.0098524	0.370453
Cardiomyopath	159811	A	G	0.0118368	0.714735
Cardiomyopath	159811	A	G	0.0271431	0.031972
Cardiomyopath	159811	C	T	-0.030081	0.027798
Cardiomyopath	159811	T	C	-0.0102176	0.424276
Cardiomyopath	159811	T	G	-0.0176865	0.091868
Cardiomyopath	159811	T	C	-0.023713	0.04104
Cardiomyopath	159811	A	C	-0.0111924	0.368826
Cardiomyopath	159811	G	A	-0.0162463	0.114428
Cardiomyopath	159811	A	G	-0.0195322	0.085011
Cardiomyopath	159811	A	G	-0.0217207	0.063928
Cardiomyopath	159811	T	C	0.0388327	0.01703

Cardiomyopath	159811	T	C	-0.0420185	0.017318
Cardiomyopath	159811	T	C	-0.0387119	0.023291
Cardiomyopath	159811	T	C	0.0132343	0.212894
Cardiomyopath	159811	G	T	0.0118316	0.241258
Cardiomyopath	159811	G	A	-0.0200821	0.069722
Cardiomyopath	159811	C	T	-0.0581919	0.010194
Cardiomyopath	159811	C	T	-0.0287918	0.032879
Cardiomyopath	159811	T	C	-0.028982	0.032703
Cardiomyopath	159811	T	C	-0.0120578	0.230187
Cardiomyopath	159811	C	T	0.0460816	0.011858
Cardiomyopath	159811	A	G	0.027384	0.036362
Cardiomyopath	159811	T	C	-0.035513	0.020331
Cardiomyopath	159811	A	G	0.0117626	0.377409
Cardiomyopath	159811	C	A	-0.0124891	0.311303
Cardiomyopath	159811	T	C	-0.0258626	0.040057
Cardiomyopath	159811	T	C	0.0105209	0.657728
Cardiomyopath	159811	T	C	-0.0127818	0.190704
Cardiomyopath	159811	G	A	0.0417032	0.017923
Cardiomyopath	159811	C	T	-0.0494714	0.010542
Cardiomyopath	159811	T	G	-0.0107374	0.419106
Cardiomyopath	159811	A	G	-0.032334	0.028942
Cardiomyopath	159811	G	A	0.0420186	0.017845
Cardiomyopath	159811	A	G	-0.0164801	0.109299
Cardiomyopath	159811	A	G	-0.0193433	0.085011
Cardiomyopath	159811	G	T	0.0533208	0.010465
Cardiomyopath	159811	G	A	-0.0480395	0.011699
Cardiomyopath	159811	T	G	0.0127384	0.187616
Cardiomyopath	159811	A	G	-0.0126249	0.283075
Cardiomyopath	159811	A	C	0.0104397	0.347049
Cardiomyopath	159811	G	A	0.012725	0.781731
Cardiomyopath	159811	T	C	0.0216661	0.212894
Cardiomyopath	159811	C	T	-0.0139685	0.201776
Cardiomyopath	159811	A	G	0.0122914	0.374311
Cardiomyopath	159811	A	C	0.0123705	0.500143
Cardiomyopath	159811	C	T	0.0216856	0.064265
Cardiomyopath	159811	A	C	-0.0139275	0.151277
Cardiomyopath	159811	G	T	-0.0434289	0.013488
Cardiomyopath	159811	C	T	-0.0371072	0.018141
Cardiomyopath	159811	G	T	-0.0114166	0.298539
Cardiomyopath	159811	T	G	0.0146809	0.167529
Cardiomyopath	159811	T	C	-0.0107288	0.665119
Cardiomyopath	159811	A	G	-0.0246758	0.049304

Cardiomyopath	159811 G	A	0.0134447	0.171589
Cardiomyopath	159811 G	A	-0.0116357	0.759827
Cardiomyopath	159811 T	G	-0.0181851	0.083179
Cardiomyopath	159811 C	A	0.0120084	0.364496
Cardiomyopath	159811 A	C	0.0544302	0.008242
Cardiomyopath	159811 T	C	0.0111347	0.657728
Cardiomyopath	159811 A	G	-0.0351148	0.021377
Cardiomyopath	159811 C	T	-0.0181174	0.082788
Cardiomyopath	159811 A	G	0.0178158	0.097718
Cardiomyopath	159811 C	T	0.0114362	0.71603
Cardiomyopath	159811 G	T	-0.0110872	0.645782
Cardiomyopath	159811 A	G	-0.0123919	0.465814
Cardiomyopath	159811 G	A	-0.0105236	0.339919
Cardiomyopath	159811 A	G	-0.0135133	0.166699
Cardiomyopath	159811 T	C	0.113396	0.003862
Cardiomyopath	159811 G	A	-0.0113177	0.286053
Cardiomyopath	159811 C	T	0.02003	0.074366
Cardiomyopath	159811 C	A	-0.0160909	0.149235
Cardiomyopath	159811 C	A	-0.0129805	0.186948
Cardiomyopath	159811 C	A	-0.0114094	0.441655
Cardiomyopath	159811 T	C	0.0135219	0.273909
Cardiomyopath	159811 T	C	0.0313937	0.036507
Cardiomyopath	159811 A	G	0.0109471	0.71427
Cardiomyopath	159811 G	A	-0.0467891	0.013105
Cardiomyopath	159811 C	T	-0.0335181	0.027757
Cardiomyopath	159811 T	G	-0.0187398	0.092425
Cardiomyopath	159811 A	C	0.0130804	0.182153
Cardiomyopath	159811 A	G	0.0139827	0.151287
Cardiomyopath	159811 C	T	0.00986771	0.478622
Cardiomyopath	159811 A	G	0.0102987	0.437758
Cardiomyopath	159811 C	T	0.00990463	0.425557
Cardiomyopath	159811 A	G	0.0573849	0.008598
Cardiomyopath	159811 T	C	-0.0355946	0.018977
Cardiomyopath	159811 A	G	-0.0405505	0.015591
Cardiomyopath	159811 A	C	-0.0107806	0.286782
Cardiomyopath	159811 G	A	0.0289547	0.030344
Cardiomyopath	159811 T	C	0.0096854	0.565309
Cardiomyopath	159811 C	T	0.0101453	0.33166
Cardiomyopath	159811 C	T	-0.0104559	0.327404
Cardiomyopath	159811 G	A	-0.0268806	0.034508
Cardiomyopath	159811 T	C	-0.0098819	0.455668
Cardiomyopath	159811 A	G	0.0113885	0.285878

Hypertrophic ca	218792	A	G	0.0150639	0.130178
Hypertrophic ca	218792	T	G	0.00972771	0.484233
Hypertrophic ca	218792	A	G	-0.0102936	0.416147
Hypertrophic ca	218792	T	C	-0.009853	0.417906
Hypertrophic ca	218792	T	C	0.0180857	0.072984
Hypertrophic ca	218792	A	C	0.0149971	0.16794
Hypertrophic ca	218792	A	G	0.0156804	0.154461
Hypertrophic ca	218792	A	C	0.0124993	0.186886
Hypertrophic ca	218792	T	C	-0.0386416	0.016208
Hypertrophic ca	218792	G	A	-0.0110104	0.2435
Hypertrophic ca	218792	G	A	-0.0098554	0.641994
Hypertrophic ca	218792	A	G	0.0867972	0.003383
Hypertrophic ca	218792	A	G	-0.0132439	0.153233
Hypertrophic ca	218792	A	G	-0.0095825	0.548637
Hypertrophic ca	218792	G	A	0.0140134	0.782419
Hypertrophic ca	218792	T	C	0.0196624	0.219436
Hypertrophic ca	218792	C	T	0.00970902	0.380091
Hypertrophic ca	218792	C	T	-0.0130453	0.200795
Hypertrophic ca	218792	C	A	-0.0114495	0.243279
Hypertrophic ca	218792	T	C	0.0154658	0.12919
Hypertrophic ca	218792	A	G	0.0103554	0.373673
Hypertrophic ca	218792	T	C	-0.0384693	0.017336
Hypertrophic ca	218792	G	T	-0.0429541	0.013342
Hypertrophic ca	218792	T	C	-0.018716	0.083064
Hypertrophic ca	218792	G	A	0.0102993	0.698769
Hypertrophic ca	218792	T	C	-0.0100751	0.663321
Hypertrophic ca	218792	G	A	0.0139755	0.130449
Hypertrophic ca	218792	C	T	0.0201622	0.064993
Hypertrophic ca	218792	G	A	-0.0153369	0.109891
Hypertrophic ca	218792	A	G	0.0107315	0.611738
Hypertrophic ca	218792	T	C	0.0107705	0.33449
Hypertrophic ca	218792	C	T	-0.0350603	0.019349
Hypertrophic ca	218792	G	A	0.0108015	0.745173
Hypertrophic ca	218792	T	C	0.0209748	0.079749
Hypertrophic ca	218792	A	G	-0.0359277	0.018434
Hypertrophic ca	218792	G	A	0.0160831	0.881571
Hypertrophic ca	218792	C	T	0.0113964	0.239895
Hypertrophic ca	218792	A	G	0.00969921	0.387517
Hypertrophic ca	218792	G	T	-0.0204187	0.062116
Hypertrophic ca	218792	T	C	-0.0113675	0.245752
Hypertrophic ca	218792	G	T	-0.0175571	0.15565
Hypertrophic ca	218792	C	A	-0.0300886	0.02677

Hypertrophic ca	218792	C	A	0.0118268	0.365075
Hypertrophic ca	218792	A	G	0.0300981	0.028151
Hypertrophic ca	218792	T	C	-0.0113821	0.23522
Hypertrophic ca	218792	T	C	0.0129274	0.172431
Hypertrophic ca	218792	T	C	0.00992086	0.658049
Hypertrophic ca	218792	T	G	-0.0207931	0.070424
Hypertrophic ca	218792	A	G	-0.0106399	0.46467
Hypertrophic ca	218792	C	T	0.0134611	0.158973
Hypertrophic ca	218792	T	C	-0.009463	0.51929
Hypertrophic ca	218792	T	C	0.0123158	0.230477
Hypertrophic ca	218792	G	A	0.011581	0.769457
Hypertrophic ca	218792	T	C	0.0946143	0.003807
Hypertrophic ca	218792	G	A	-0.0107992	0.286147
Hypertrophic ca	218792	T	C	-0.0287129	0.029919
Hypertrophic ca	218792	G	T	-0.0331207	0.025544
Hypertrophic ca	218792	C	A	-0.0132066	0.186513
Hypertrophic ca	218792	C	T	-0.0231955	0.046759
Hypertrophic ca	218792	A	G	0.0135202	0.165706
Hypertrophic ca	218792	G	A	0.0159377	0.106329
Hypertrophic ca	218792	T	C	0.0148569	0.274855
Hypertrophic ca	218792	A	G	-0.0154582	0.113504
Hypertrophic ca	218792	T	C	-0.0098524	0.370453
Hypertrophic ca	218792	A	G	0.0118368	0.714735
Hypertrophic ca	218792	A	G	0.0271431	0.031972
Hypertrophic ca	218792	C	T	-0.030081	0.027798
Hypertrophic ca	218792	T	C	-0.0102176	0.424276
Hypertrophic ca	218792	T	G	-0.0176865	0.091868
Hypertrophic ca	218792	T	C	-0.023713	0.04104
Hypertrophic ca	218792	A	C	-0.0111924	0.368826
Hypertrophic ca	218792	A	G	-0.0193433	0.085011
Hypertrophic ca	218792	G	T	0.0533208	0.010465
Hypertrophic ca	218792	G	A	-0.0480395	0.011699
Hypertrophic ca	218792	T	G	0.0127384	0.187616
Hypertrophic ca	218792	A	G	-0.0126249	0.283075
Hypertrophic ca	218792	A	C	0.0104397	0.347049
Hypertrophic ca	218792	G	A	0.012725	0.781731
Hypertrophic ca	218792	T	C	0.0216661	0.212894
Hypertrophic ca	218792	C	T	-0.0139685	0.201776
Hypertrophic ca	218792	A	G	0.0122914	0.374311
Hypertrophic ca	218792	A	C	0.0123705	0.500143
Hypertrophic ca	218792	C	T	0.0216856	0.064265
Hypertrophic ca	218792	A	C	-0.0139275	0.151277

Hypertrophic ca	218792	G	T	-0.0434289	0.013488
Hypertrophic ca	218792	C	T	-0.0371072	0.018141
Hypertrophic ca	218792	G	T	-0.0114166	0.298539
Hypertrophic ca	218792	T	G	0.0146809	0.167529
Hypertrophic ca	218792	T	C	-0.0107288	0.665119
Hypertrophic ca	218792	A	G	-0.0246758	0.049304
Hypertrophic ca	218792	G	A	0.0134447	0.171589
Hypertrophic ca	218792	G	A	-0.0116357	0.759827
Hypertrophic ca	218792	T	G	-0.0181851	0.083179
Hypertrophic ca	218792	C	A	0.0120084	0.364496
Hypertrophic ca	218792	A	C	0.0544302	0.008242
Hypertrophic ca	218792	T	C	0.0111347	0.657728
Hypertrophic ca	218792	A	G	-0.0351148	0.021377
Hypertrophic ca	218792	C	T	-0.0181174	0.082788
Hypertrophic ca	218792	A	G	0.0178158	0.097718
Hypertrophic ca	218792	C	T	0.0114362	0.71603
Hypertrophic ca	218792	G	T	-0.0110872	0.645782
Hypertrophic ca	218792	A	G	-0.0123919	0.465814
Hypertrophic ca	218792	G	A	-0.0105236	0.339919
Hypertrophic ca	218792	A	G	-0.0135133	0.166699
Hypertrophic ca	218792	T	C	0.113396	0.003862
Hypertrophic ca	218792	G	A	-0.0113177	0.286053
Hypertrophic ca	218792	C	T	0.02003	0.074366
Hypertrophic ca	218792	C	A	-0.0160909	0.149235
Hypertrophic ca	218792	C	A	-0.0129805	0.186948
Hypertrophic ca	218792	C	A	-0.0114094	0.441655
Hypertrophic ca	218792	T	C	0.0135219	0.273909
Hypertrophic ca	218792	T	C	0.0313937	0.036507
Hypertrophic ca	218792	A	G	0.0109471	0.71427
Hypertrophic ca	218792	G	A	-0.0467891	0.013105
Hypertrophic ca	218792	C	T	-0.0335181	0.027757
Hypertrophic ca	218792	T	G	-0.0187398	0.092425
Hypertrophic ca	218792	A	C	0.0130804	0.182153
Hypertrophic ca	218792	A	G	0.0139827	0.151287
Hypertrophic ca	218792	C	T	0.00986771	0.478622
Hypertrophic ca	218792	A	G	0.0102987	0.437758
Hypertrophic ca	218792	G	A	-0.0162463	0.114428
Hypertrophic ca	218792	A	G	-0.0195322	0.085011
Hypertrophic ca	218792	A	G	-0.0217207	0.063928
Hypertrophic ca	218792	T	C	0.0388327	0.01703
Hypertrophic ca	218792	T	C	-0.0420185	0.017318
Hypertrophic ca	218792	T	C	-0.0387119	0.023291

Hypertrophic ca	218792	T	C	0.0132343	0.212894
Hypertrophic ca	218792	G	T	0.0118316	0.241258
Hypertrophic ca	218792	G	A	-0.0200821	0.069722
Hypertrophic ca	218792	C	T	-0.0581919	0.010194
Hypertrophic ca	218792	C	T	-0.0287918	0.032879
Hypertrophic ca	218792	T	C	-0.028982	0.032703
Hypertrophic ca	218792	T	C	-0.0120578	0.230187
Hypertrophic ca	218792	C	T	0.0460816	0.011858
Hypertrophic ca	218792	A	G	0.027384	0.036362
Hypertrophic ca	218792	T	C	-0.035513	0.020331
Hypertrophic ca	218792	A	G	0.0117626	0.377409
Hypertrophic ca	218792	C	A	-0.0124891	0.311303
Hypertrophic ca	218792	T	C	-0.0258626	0.040057
Hypertrophic ca	218792	T	C	0.0105209	0.657728
Hypertrophic ca	218792	T	C	-0.0127818	0.190704
Hypertrophic ca	218792	G	A	0.0417032	0.017923
Hypertrophic ca	218792	C	T	-0.0494714	0.010542
Hypertrophic ca	218792	T	G	-0.0107374	0.419106
Hypertrophic ca	218792	A	G	-0.032334	0.028942
Hypertrophic ca	218792	G	A	0.0420186	0.017845
Hypertrophic ca	218792	A	G	-0.0164801	0.109299
Hypertrophic ca	218792	C	T	0.00990463	0.425557
Hypertrophic ca	218792	A	G	0.0573849	0.008598
Hypertrophic ca	218792	T	C	-0.0355946	0.018977
Hypertrophic ca	218792	A	G	-0.0405505	0.015591
Hypertrophic ca	218792	A	C	-0.0107806	0.286782
Hypertrophic ca	218792	G	A	0.0289547	0.030344
Hypertrophic ca	218792	T	C	0.0096854	0.565309
Hypertrophic ca	218792	C	T	0.0101453	0.33166
Hypertrophic ca	218792	C	T	-0.0104559	0.327404
Hypertrophic ca	218792	G	A	-0.0268806	0.034508
Hypertrophic ca	218792	T	C	-0.0098819	0.455668
Hypertrophic ca	218792	A	G	0.0113885	0.285878
Hypertension	218754	A	G	0.0150639	0.130178
Hypertension	218754	T	G	0.00972771	0.484233
Hypertension	218754	A	G	-0.0102936	0.416147
Hypertension	218754	T	C	-0.009853	0.417906
Hypertension	218754	T	C	0.0180857	0.072984
Hypertension	218754	A	C	0.0149971	0.16794
Hypertension	218754	A	G	0.0156804	0.154461
Hypertension	218754	A	C	0.0124993	0.186886
Hypertension	218754	T	C	-0.0386416	0.016208

Hypertension	218754	G	A	-0.0110104	0.2435
Hypertension	218754	G	A	-0.0098554	0.641994
Hypertension	218754	A	G	0.0867972	0.003383
Hypertension	218754	A	G	-0.0132439	0.153233
Hypertension	218754	A	G	-0.0095825	0.548637
Hypertension	218754	G	A	0.0140134	0.782419
Hypertension	218754	T	C	0.0196624	0.219436
Hypertension	218754	C	T	0.00970902	0.380091
Hypertension	218754	C	T	-0.0130453	0.200795
Hypertension	218754	C	A	-0.0114495	0.243279
Hypertension	218754	T	C	0.0154658	0.12919
Hypertension	218754	A	G	0.0103554	0.373673
Hypertension	218754	T	C	-0.0384693	0.017336
Hypertension	218754	G	T	-0.0429541	0.013342
Hypertension	218754	T	C	-0.018716	0.083064
Hypertension	218754	G	A	0.0102993	0.698769
Hypertension	218754	T	C	-0.0100751	0.663321
Hypertension	218754	G	A	0.0139755	0.130449
Hypertension	218754	C	T	0.0201622	0.064993
Hypertension	218754	G	A	-0.0153369	0.109891
Hypertension	218754	A	G	0.0107315	0.611738
Hypertension	218754	T	C	0.0107705	0.33449
Hypertension	218754	C	T	-0.0350603	0.019349
Hypertension	218754	G	A	0.0108015	0.745173
Hypertension	218754	T	C	0.0209748	0.079749
Hypertension	218754	A	G	-0.0359277	0.018434
Hypertension	218754	G	A	0.0160831	0.881571
Hypertension	218754	C	T	0.0113964	0.239895
Hypertension	218754	A	G	0.00969921	0.387517
Hypertension	218754	G	T	-0.0204187	0.062116
Hypertension	218754	T	C	-0.0113675	0.245752
Hypertension	218754	G	T	-0.0175571	0.15565
Hypertension	218754	C	A	-0.0300886	0.02677
Hypertension	218754	C	A	0.0118268	0.365075
Hypertension	218754	A	G	0.0300981	0.028151
Hypertension	218754	T	C	-0.0113821	0.23522
Hypertension	218754	T	C	0.0129274	0.172431
Hypertension	218754	T	C	0.00992086	0.658049
Hypertension	218754	T	G	-0.0207931	0.070424
Hypertension	218754	A	G	-0.0106399	0.46467
Hypertension	218754	C	T	0.0134611	0.158973
Hypertension	218754	T	C	-0.009463	0.51929

Hypertension	218754	T	C	0.0123158	0.230477
Hypertension	218754	G	A	0.011581	0.769457
Hypertension	218754	T	C	0.0946143	0.003807
Hypertension	218754	G	A	-0.0107992	0.286147
Hypertension	218754	T	C	-0.0287129	0.029919
Hypertension	218754	G	T	-0.0331207	0.025544
Hypertension	218754	C	A	-0.0132066	0.186513
Hypertension	218754	C	T	-0.0231955	0.046759
Hypertension	218754	A	G	0.0135202	0.165706
Hypertension	218754	G	A	0.0159377	0.106329
Hypertension	218754	T	C	0.0148569	0.274855
Hypertension	218754	A	G	-0.0154582	0.113504
Hypertension	218754	T	C	-0.0098524	0.370453
Hypertension	218754	A	G	0.0118368	0.714735
Hypertension	218754	A	G	0.0271431	0.031972
Hypertension	218754	C	T	-0.030081	0.027798
Hypertension	218754	T	C	-0.0102176	0.424276
Hypertension	218754	T	G	-0.0176865	0.091868
Hypertension	218754	T	C	-0.023713	0.04104
Hypertension	218754	A	C	-0.0111924	0.368826
Hypertension	218754	A	G	-0.0193433	0.085011
Hypertension	218754	G	T	0.0533208	0.010465
Hypertension	218754	G	A	-0.0480395	0.011699
Hypertension	218754	T	G	0.0127384	0.187616
Hypertension	218754	A	G	-0.0126249	0.283075
Hypertension	218754	A	C	0.0104397	0.347049
Hypertension	218754	G	A	0.012725	0.781731
Hypertension	218754	T	C	0.0216661	0.212894
Hypertension	218754	C	T	-0.0139685	0.201776
Hypertension	218754	A	G	0.0122914	0.374311
Hypertension	218754	A	C	0.0123705	0.500143
Hypertension	218754	C	T	0.0216856	0.064265
Hypertension	218754	A	C	-0.0139275	0.151277
Hypertension	218754	G	T	-0.0434289	0.013488
Hypertension	218754	C	T	-0.0371072	0.018141
Hypertension	218754	G	T	-0.0114166	0.298539
Hypertension	218754	T	G	0.0146809	0.167529
Hypertension	218754	T	C	-0.0107288	0.665119
Hypertension	218754	A	G	-0.0246758	0.049304
Hypertension	218754	G	A	0.0134447	0.171589
Hypertension	218754	G	A	-0.0116357	0.759827
Hypertension	218754	T	G	-0.0181851	0.083179

Hypertension	218754	C	A	0.0120084	0.364496
Hypertension	218754	A	C	0.0544302	0.008242
Hypertension	218754	T	C	0.0111347	0.657728
Hypertension	218754	A	G	-0.0351148	0.021377
Hypertension	218754	C	T	-0.0181174	0.082788
Hypertension	218754	A	G	0.0178158	0.097718
Hypertension	218754	C	T	0.0114362	0.71603
Hypertension	218754	G	T	-0.0110872	0.645782
Hypertension	218754	A	G	-0.0123919	0.465814
Hypertension	218754	G	A	-0.0105236	0.339919
Hypertension	218754	A	G	-0.0135133	0.166699
Hypertension	218754	T	C	0.113396	0.003862
Hypertension	218754	G	A	-0.0113177	0.286053
Hypertension	218754	C	T	0.02003	0.074366
Hypertension	218754	C	A	-0.0160909	0.149235
Hypertension	218754	C	A	-0.0129805	0.186948
Hypertension	218754	C	A	-0.0114094	0.441655
Hypertension	218754	T	C	0.0135219	0.273909
Hypertension	218754	T	C	0.0313937	0.036507
Hypertension	218754	A	G	0.0109471	0.71427
Hypertension	218754	G	A	-0.0467891	0.013105
Hypertension	218754	C	T	-0.0335181	0.027757
Hypertension	218754	T	G	-0.0187398	0.092425
Hypertension	218754	A	C	0.0130804	0.182153
Hypertension	218754	A	G	0.0139827	0.151287
Hypertension	218754	C	T	0.00986771	0.478622
Hypertension	218754	A	G	0.0102987	0.437758
Hypertension	218754	G	A	-0.0162463	0.114428
Hypertension	218754	A	G	-0.0195322	0.085011
Hypertension	218754	A	G	-0.0217207	0.063928
Hypertension	218754	T	C	0.0388327	0.01703
Hypertension	218754	T	C	-0.0420185	0.017318
Hypertension	218754	T	C	-0.0387119	0.023291
Hypertension	218754	T	C	0.0132343	0.212894
Hypertension	218754	G	T	0.0118316	0.241258
Hypertension	218754	G	A	-0.0200821	0.069722
Hypertension	218754	C	T	-0.0581919	0.010194
Hypertension	218754	C	T	-0.0287918	0.032879
Hypertension	218754	T	C	-0.028982	0.032703
Hypertension	218754	T	C	-0.0120578	0.230187
Hypertension	218754	C	T	0.0460816	0.011858
Hypertension	218754	A	G	0.027384	0.036362

Hypertension	218754	T	C	-0.035513	0.020331
Hypertension	218754	A	G	0.0117626	0.377409
Hypertension	218754	C	A	-0.0124891	0.311303
Hypertension	218754	T	C	-0.0258626	0.040057
Hypertension	218754	T	C	0.0105209	0.657728
Hypertension	218754	T	C	-0.0127818	0.190704
Hypertension	218754	G	A	0.0417032	0.017923
Hypertension	218754	C	T	-0.0494714	0.010542
Hypertension	218754	T	G	-0.0107374	0.419106
Hypertension	218754	A	G	-0.032334	0.028942
Hypertension	218754	G	A	0.0420186	0.017845
Hypertension	218754	A	G	-0.0164801	0.109299
Hypertension	218754	C	T	0.00990463	0.425557
Hypertension	218754	A	G	0.0573849	0.008598
Hypertension	218754	T	C	-0.0355946	0.018977
Hypertension	218754	A	G	-0.0405505	0.015591
Hypertension	218754	A	C	-0.0107806	0.286782
Hypertension	218754	G	A	0.0289547	0.030344
Hypertension	218754	T	C	0.0096854	0.565309
Hypertension	218754	C	T	0.0101453	0.33166
Hypertension	218754	C	T	-0.0104559	0.327404
Hypertension	218754	G	A	-0.0268806	0.034508
Hypertension	218754	T	C	-0.0098819	0.455668
Hypertension	218754	A	G	0.0113885	0.285878
Ischemic heart	218792	A	G	-0.0193433	0.085011
Ischemic heart	218792	G	T	0.0533208	0.010465
Ischemic heart	218792	G	A	-0.0480395	0.011699
Ischemic heart	218792	T	G	0.0127384	0.187616
Ischemic heart	218792	A	G	-0.0126249	0.283075
Ischemic heart	218792	A	C	0.0104397	0.347049
Ischemic heart	218792	G	A	0.012725	0.781731
Ischemic heart	218792	T	C	0.0216661	0.212894
Ischemic heart	218792	C	T	-0.0139685	0.201776
Ischemic heart	218792	A	G	0.0122914	0.374311
Ischemic heart	218792	A	C	0.0123705	0.500143
Ischemic heart	218792	C	T	0.0216856	0.064265
Ischemic heart	218792	A	C	-0.0139275	0.151277
Ischemic heart	218792	G	T	-0.0434289	0.013488
Ischemic heart	218792	C	T	-0.0371072	0.018141
Ischemic heart	218792	G	T	-0.0114166	0.298539
Ischemic heart	218792	T	G	0.0146809	0.167529
Ischemic heart	218792	T	C	-0.0107288	0.665119

Ischemic heart	218792	A	G	-0.0246758	0.049304
Ischemic heart	218792	G	A	0.0134447	0.171589
Ischemic heart	218792	G	A	-0.0116357	0.759827
Ischemic heart	218792	T	G	-0.0181851	0.083179
Ischemic heart	218792	C	A	0.0120084	0.364496
Ischemic heart	218792	A	C	0.0544302	0.008242
Ischemic heart	218792	T	C	0.0111347	0.657728
Ischemic heart	218792	A	G	-0.0351148	0.021377
Ischemic heart	218792	C	T	-0.0181174	0.082788
Ischemic heart	218792	A	G	0.0178158	0.097718
Ischemic heart	218792	C	T	0.0114362	0.71603
Ischemic heart	218792	G	T	-0.0110872	0.645782
Ischemic heart	218792	A	G	-0.0123919	0.465814
Ischemic heart	218792	G	A	-0.0105236	0.339919
Ischemic heart	218792	A	G	-0.0135133	0.166699
Ischemic heart	218792	T	C	0.113396	0.003862
Ischemic heart	218792	G	A	-0.0113177	0.286053
Ischemic heart	218792	C	T	0.02003	0.074366
Ischemic heart	218792	C	A	-0.0160909	0.149235
Ischemic heart	218792	C	A	-0.0129805	0.186948
Ischemic heart	218792	C	A	-0.0114094	0.441655
Ischemic heart	218792	T	C	0.0135219	0.273909
Ischemic heart	218792	T	C	0.0313937	0.036507
Ischemic heart	218792	A	G	0.0109471	0.71427
Ischemic heart	218792	G	A	-0.0467891	0.013105
Ischemic heart	218792	C	T	-0.0335181	0.027757
Ischemic heart	218792	T	G	-0.0187398	0.092425
Ischemic heart	218792	A	C	0.0130804	0.182153
Ischemic heart	218792	A	G	0.0139827	0.151287
Ischemic heart	218792	C	T	0.00986771	0.478622
Ischemic heart	218792	A	G	0.0102987	0.437758
Ischemic heart	218792	A	G	0.0150639	0.130178
Ischemic heart	218792	T	G	0.00972771	0.484233
Ischemic heart	218792	A	G	-0.0102936	0.416147
Ischemic heart	218792	T	C	-0.009853	0.417906
Ischemic heart	218792	T	C	0.0180857	0.072984
Ischemic heart	218792	A	C	0.0149971	0.16794
Ischemic heart	218792	A	G	0.0156804	0.154461
Ischemic heart	218792	A	C	0.0124993	0.186886
Ischemic heart	218792	T	C	-0.0386416	0.016208
Ischemic heart	218792	G	A	-0.0110104	0.2435
Ischemic heart	218792	G	A	-0.0098554	0.641994

Ischemic heart	218792	A	G	0.0867972	0.003383
Ischemic heart	218792	A	G	-0.0132439	0.153233
Ischemic heart	218792	A	G	-0.0095825	0.548637
Ischemic heart	218792	G	A	0.0140134	0.782419
Ischemic heart	218792	T	C	0.0196624	0.219436
Ischemic heart	218792	C	T	0.00970902	0.380091
Ischemic heart	218792	C	T	-0.0130453	0.200795
Ischemic heart	218792	C	A	-0.0114495	0.243279
Ischemic heart	218792	T	C	0.0154658	0.12919
Ischemic heart	218792	A	G	0.0103554	0.373673
Ischemic heart	218792	T	C	-0.0384693	0.017336
Ischemic heart	218792	G	T	-0.0429541	0.013342
Ischemic heart	218792	T	C	-0.018716	0.083064
Ischemic heart	218792	G	A	0.0102993	0.698769
Ischemic heart	218792	T	C	-0.0100751	0.663321
Ischemic heart	218792	G	A	0.0139755	0.130449
Ischemic heart	218792	C	T	0.0201622	0.064993
Ischemic heart	218792	G	A	-0.0153369	0.109891
Ischemic heart	218792	A	G	0.0107315	0.611738
Ischemic heart	218792	T	C	0.0107705	0.33449
Ischemic heart	218792	C	T	-0.0350603	0.019349
Ischemic heart	218792	G	A	0.0108015	0.745173
Ischemic heart	218792	T	C	0.0209748	0.079749
Ischemic heart	218792	A	G	-0.0359277	0.018434
Ischemic heart	218792	G	A	0.0160831	0.881571
Ischemic heart	218792	C	T	0.0113964	0.239895
Ischemic heart	218792	A	G	0.00969921	0.387517
Ischemic heart	218792	G	T	-0.0204187	0.062116
Ischemic heart	218792	T	C	-0.0113675	0.245752
Ischemic heart	218792	G	T	-0.0175571	0.15565
Ischemic heart	218792	C	A	-0.0300886	0.02677
Ischemic heart	218792	C	A	0.0118268	0.365075
Ischemic heart	218792	A	G	0.0300981	0.028151
Ischemic heart	218792	T	C	-0.0113821	0.23522
Ischemic heart	218792	T	C	0.0129274	0.172431
Ischemic heart	218792	T	C	0.00992086	0.658049
Ischemic heart	218792	T	G	-0.0207931	0.070424
Ischemic heart	218792	A	G	-0.0106399	0.46467
Ischemic heart	218792	C	T	0.0134611	0.158973
Ischemic heart	218792	T	C	-0.009463	0.51929
Ischemic heart	218792	T	C	0.0123158	0.230477
Ischemic heart	218792	G	A	0.011581	0.769457

Ischemic heart	218792	T	C	0.0946143	0.003807
Ischemic heart	218792	G	A	-0.0107992	0.286147
Ischemic heart	218792	T	C	-0.0287129	0.029919
Ischemic heart	218792	G	T	-0.0331207	0.025544
Ischemic heart	218792	C	A	-0.0132066	0.186513
Ischemic heart	218792	C	T	-0.0231955	0.046759
Ischemic heart	218792	A	G	0.0135202	0.165706
Ischemic heart	218792	G	A	0.0159377	0.106329
Ischemic heart	218792	T	C	0.0148569	0.274855
Ischemic heart	218792	A	G	-0.0154582	0.113504
Ischemic heart	218792	T	C	-0.0098524	0.370453
Ischemic heart	218792	A	G	0.0118368	0.714735
Ischemic heart	218792	A	G	0.0271431	0.031972
Ischemic heart	218792	C	T	-0.030081	0.027798
Ischemic heart	218792	T	C	-0.0102176	0.424276
Ischemic heart	218792	T	G	-0.0176865	0.091868
Ischemic heart	218792	T	C	-0.023713	0.04104
Ischemic heart	218792	A	C	-0.0111924	0.368826
Ischemic heart	218792	G	A	-0.0162463	0.114428
Ischemic heart	218792	A	G	-0.0195322	0.085011
Ischemic heart	218792	A	G	-0.0217207	0.063928
Ischemic heart	218792	T	C	0.0388327	0.01703
Ischemic heart	218792	T	C	-0.0420185	0.017318
Ischemic heart	218792	T	C	-0.0387119	0.023291
Ischemic heart	218792	T	C	0.0132343	0.212894
Ischemic heart	218792	G	T	0.0118316	0.241258
Ischemic heart	218792	G	A	-0.0200821	0.069722
Ischemic heart	218792	C	T	-0.0581919	0.010194
Ischemic heart	218792	C	T	-0.0287918	0.032879
Ischemic heart	218792	T	C	-0.028982	0.032703
Ischemic heart	218792	T	C	-0.0120578	0.230187
Ischemic heart	218792	C	T	0.0460816	0.011858
Ischemic heart	218792	A	G	0.027384	0.036362
Ischemic heart	218792	T	C	-0.035513	0.020331
Ischemic heart	218792	A	G	0.0117626	0.377409
Ischemic heart	218792	C	A	-0.0124891	0.311303
Ischemic heart	218792	T	C	-0.0258626	0.040057
Ischemic heart	218792	T	C	0.0105209	0.657728
Ischemic heart	218792	T	C	-0.0127818	0.190704
Ischemic heart	218792	G	A	0.0417032	0.017923
Ischemic heart	218792	C	T	-0.0494714	0.010542
Ischemic heart	218792	T	G	-0.0107374	0.419106

Ischemic heart	218792	A	G	-0.032334	0.028942
Ischemic heart	218792	G	A	0.0420186	0.017845
Ischemic heart	218792	A	G	-0.0164801	0.109299
Ischemic heart	218792	C	T	0.00990463	0.425557
Ischemic heart	218792	A	G	0.0573849	0.008598
Ischemic heart	218792	T	C	-0.0355946	0.018977
Ischemic heart	218792	A	G	-0.0405505	0.015591
Ischemic heart	218792	A	C	-0.0107806	0.286782
Ischemic heart	218792	G	A	0.0289547	0.030344
Ischemic heart	218792	T	C	0.0096854	0.565309
Ischemic heart	218792	C	T	0.0101453	0.33166
Ischemic heart	218792	C	T	-0.0104559	0.327404
Ischemic heart	218792	G	A	-0.0268806	0.034508
Ischemic heart	218792	T	C	-0.0098819	0.455668
Ischemic heart	218792	A	G	0.0113885	0.285878
Non-ischemic c	187152	A	G	-0.0193433	0.085011
Non-ischemic c	187152	G	T	0.0533208	0.010465
Non-ischemic c	187152	G	A	-0.0480395	0.011699
Non-ischemic c	187152	T	G	0.0127384	0.187616
Non-ischemic c	187152	A	G	-0.0126249	0.283075
Non-ischemic c	187152	A	C	0.0104397	0.347049
Non-ischemic c	187152	G	A	0.012725	0.781731
Non-ischemic c	187152	T	C	0.0216661	0.212894
Non-ischemic c	187152	C	T	-0.0139685	0.201776
Non-ischemic c	187152	A	G	0.0122914	0.374311
Non-ischemic c	187152	A	C	0.0123705	0.500143
Non-ischemic c	187152	C	T	0.0216856	0.064265
Non-ischemic c	187152	A	C	-0.0139275	0.151277
Non-ischemic c	187152	G	T	-0.0434289	0.013488
Non-ischemic c	187152	C	T	-0.0371072	0.018141
Non-ischemic c	187152	G	T	-0.0114166	0.298539
Non-ischemic c	187152	T	G	0.0146809	0.167529
Non-ischemic c	187152	T	C	-0.0107288	0.665119
Non-ischemic c	187152	A	G	-0.0246758	0.049304
Non-ischemic c	187152	G	A	0.0134447	0.171589
Non-ischemic c	187152	G	A	-0.0116357	0.759827
Non-ischemic c	187152	T	G	-0.0181851	0.083179
Non-ischemic c	187152	C	A	0.0120084	0.364496
Non-ischemic c	187152	A	C	0.0544302	0.008242
Non-ischemic c	187152	T	C	0.0111347	0.657728
Non-ischemic c	187152	A	G	-0.0351148	0.021377
Non-ischemic c	187152	C	T	-0.0181174	0.082788

Non-ischemic c	187152	A	G	0.0178158	0.097718
Non-ischemic c	187152	C	T	0.0114362	0.71603
Non-ischemic c	187152	G	T	-0.0110872	0.645782
Non-ischemic c	187152	A	G	-0.0123919	0.465814
Non-ischemic c	187152	G	A	-0.0105236	0.339919
Non-ischemic c	187152	A	G	-0.0135133	0.166699
Non-ischemic c	187152	T	C	0.113396	0.003862
Non-ischemic c	187152	G	A	-0.0113177	0.286053
Non-ischemic c	187152	C	T	0.02003	0.074366
Non-ischemic c	187152	C	A	-0.0160909	0.149235
Non-ischemic c	187152	C	A	-0.0129805	0.186948
Non-ischemic c	187152	C	A	-0.0114094	0.441655
Non-ischemic c	187152	T	C	0.0135219	0.273909
Non-ischemic c	187152	T	C	0.0313937	0.036507
Non-ischemic c	187152	A	G	0.0109471	0.71427
Non-ischemic c	187152	G	A	-0.0467891	0.013105
Non-ischemic c	187152	C	T	-0.0335181	0.027757
Non-ischemic c	187152	T	G	-0.0187398	0.092425
Non-ischemic c	187152	A	C	0.0130804	0.182153
Non-ischemic c	187152	A	G	0.0139827	0.151287
Non-ischemic c	187152	C	T	0.00986771	0.478622
Non-ischemic c	187152	A	G	0.0102987	0.437758
Non-ischemic c	187152	A	G	0.0150639	0.130178
Non-ischemic c	187152	T	G	0.00972771	0.484233
Non-ischemic c	187152	A	G	-0.0102936	0.416147
Non-ischemic c	187152	T	C	-0.009853	0.417906
Non-ischemic c	187152	T	C	0.0180857	0.072984
Non-ischemic c	187152	A	C	0.0149971	0.16794
Non-ischemic c	187152	A	G	0.0156804	0.154461
Non-ischemic c	187152	A	C	0.0124993	0.186886
Non-ischemic c	187152	T	C	-0.0386416	0.016208
Non-ischemic c	187152	G	A	-0.0110104	0.2435
Non-ischemic c	187152	G	A	-0.0098554	0.641994
Non-ischemic c	187152	A	G	0.0867972	0.003383
Non-ischemic c	187152	A	G	-0.0132439	0.153233
Non-ischemic c	187152	A	G	-0.0095825	0.548637
Non-ischemic c	187152	G	A	0.0140134	0.782419
Non-ischemic c	187152	T	C	0.0196624	0.219436
Non-ischemic c	187152	C	T	0.00970902	0.380091
Non-ischemic c	187152	C	T	-0.0130453	0.200795
Non-ischemic c	187152	C	A	-0.0114495	0.243279
Non-ischemic c	187152	T	C	0.0154658	0.12919

Non-ischemic c	187152	A	G	0.0103554	0.373673
Non-ischemic c	187152	T	C	-0.0384693	0.017336
Non-ischemic c	187152	G	T	-0.0429541	0.013342
Non-ischemic c	187152	T	C	-0.018716	0.083064
Non-ischemic c	187152	G	A	0.0102993	0.698769
Non-ischemic c	187152	T	C	-0.0100751	0.663321
Non-ischemic c	187152	G	A	0.0139755	0.130449
Non-ischemic c	187152	C	T	0.0201622	0.064993
Non-ischemic c	187152	G	A	-0.0153369	0.109891
Non-ischemic c	187152	A	G	0.0107315	0.611738
Non-ischemic c	187152	T	C	0.0107705	0.33449
Non-ischemic c	187152	C	T	-0.0350603	0.019349
Non-ischemic c	187152	G	A	0.0108015	0.745173
Non-ischemic c	187152	T	C	0.0209748	0.079749
Non-ischemic c	187152	A	G	-0.0359277	0.018434
Non-ischemic c	187152	G	A	0.0160831	0.881571
Non-ischemic c	187152	C	T	0.0113964	0.239895
Non-ischemic c	187152	A	G	0.00969921	0.387517
Non-ischemic c	187152	G	T	-0.0204187	0.062116
Non-ischemic c	187152	T	C	-0.0113675	0.245752
Non-ischemic c	187152	G	T	-0.0175571	0.15565
Non-ischemic c	187152	C	A	-0.0300886	0.02677
Non-ischemic c	187152	C	A	0.0118268	0.365075
Non-ischemic c	187152	A	G	0.0300981	0.028151
Non-ischemic c	187152	T	C	-0.0113821	0.23522
Non-ischemic c	187152	T	C	0.0129274	0.172431
Non-ischemic c	187152	T	C	0.00992086	0.658049
Non-ischemic c	187152	T	G	-0.0207931	0.070424
Non-ischemic c	187152	A	G	-0.0106399	0.46467
Non-ischemic c	187152	C	T	0.0134611	0.158973
Non-ischemic c	187152	T	C	-0.009463	0.51929
Non-ischemic c	187152	T	C	0.0123158	0.230477
Non-ischemic c	187152	G	A	0.011581	0.769457
Non-ischemic c	187152	T	C	0.0946143	0.003807
Non-ischemic c	187152	G	A	-0.0107992	0.286147
Non-ischemic c	187152	T	C	-0.0287129	0.029919
Non-ischemic c	187152	G	T	-0.0331207	0.025544
Non-ischemic c	187152	C	A	-0.0132066	0.186513
Non-ischemic c	187152	C	T	-0.0231955	0.046759
Non-ischemic c	187152	A	G	0.0135202	0.165706
Non-ischemic c	187152	G	A	0.0159377	0.106329
Non-ischemic c	187152	T	C	0.0148569	0.274855

Non-ischemic c	187152	A	G	-0.0154582	0.113504
Non-ischemic c	187152	T	C	-0.0098524	0.370453
Non-ischemic c	187152	A	G	0.0118368	0.714735
Non-ischemic c	187152	A	G	0.0271431	0.031972
Non-ischemic c	187152	C	T	-0.030081	0.027798
Non-ischemic c	187152	T	C	-0.0102176	0.424276
Non-ischemic c	187152	T	G	-0.0176865	0.091868
Non-ischemic c	187152	T	C	-0.023713	0.04104
Non-ischemic c	187152	A	C	-0.0111924	0.368826
Non-ischemic c	187152	C	T	0.00990463	0.425557
Non-ischemic c	187152	A	G	0.0573849	0.008598
Non-ischemic c	187152	T	C	-0.0355946	0.018977
Non-ischemic c	187152	A	G	-0.0405505	0.015591
Non-ischemic c	187152	A	C	-0.0107806	0.286782
Non-ischemic c	187152	G	A	0.0289547	0.030344
Non-ischemic c	187152	T	C	0.0096854	0.565309
Non-ischemic c	187152	C	T	0.0101453	0.33166
Non-ischemic c	187152	C	T	-0.0104559	0.327404
Non-ischemic c	187152	G	A	-0.0268806	0.034508
Non-ischemic c	187152	T	C	-0.0098819	0.455668
Non-ischemic c	187152	A	G	0.0113885	0.285878
Non-ischemic c	187152	G	A	-0.0162463	0.114428
Non-ischemic c	187152	A	G	-0.0195322	0.085011
Non-ischemic c	187152	A	G	-0.0217207	0.063928
Non-ischemic c	187152	T	C	0.0388327	0.01703
Non-ischemic c	187152	T	C	-0.0420185	0.017318
Non-ischemic c	187152	T	C	-0.0387119	0.023291
Non-ischemic c	187152	T	C	0.0132343	0.212894
Non-ischemic c	187152	G	T	0.0118316	0.241258
Non-ischemic c	187152	G	A	-0.0200821	0.069722
Non-ischemic c	187152	C	T	-0.0581919	0.010194
Non-ischemic c	187152	C	T	-0.0287918	0.032879
Non-ischemic c	187152	T	C	-0.028982	0.032703
Non-ischemic c	187152	T	C	-0.0120578	0.230187
Non-ischemic c	187152	C	T	0.0460816	0.011858
Non-ischemic c	187152	A	G	0.027384	0.036362
Non-ischemic c	187152	T	C	-0.035513	0.020331
Non-ischemic c	187152	A	G	0.0117626	0.377409
Non-ischemic c	187152	C	A	-0.0124891	0.311303
Non-ischemic c	187152	T	C	-0.0258626	0.040057
Non-ischemic c	187152	T	C	0.0105209	0.657728
Non-ischemic c	187152	T	C	-0.0127818	0.190704

Non-ischemic c	187152	G	A	0.0417032	0.017923
Non-ischemic c	187152	C	T	-0.0494714	0.010542
Non-ischemic c	187152	T	G	-0.0107374	0.419106
Non-ischemic c	187152	A	G	-0.032334	0.028942
Non-ischemic c	187152	G	A	0.0420186	0.017845
Non-ischemic c	187152	A	G	-0.0164801	0.109299
Pulmonary hea	218792	A	G	-0.0193433	0.085011
Pulmonary hea	218792	G	T	0.0533208	0.010465
Pulmonary hea	218792	G	A	-0.0480395	0.011699
Pulmonary hea	218792	T	G	0.0127384	0.187616
Pulmonary hea	218792	A	G	-0.0126249	0.283075
Pulmonary hea	218792	A	C	0.0104397	0.347049
Pulmonary hea	218792	G	A	0.012725	0.781731
Pulmonary hea	218792	T	C	0.0216661	0.212894
Pulmonary hea	218792	C	T	-0.0139685	0.201776
Pulmonary hea	218792	A	G	0.0122914	0.374311
Pulmonary hea	218792	A	C	0.0123705	0.500143
Pulmonary hea	218792	C	T	0.0216856	0.064265
Pulmonary hea	218792	A	C	-0.0139275	0.151277
Pulmonary hea	218792	G	T	-0.0434289	0.013488
Pulmonary hea	218792	C	T	-0.0371072	0.018141
Pulmonary hea	218792	G	T	-0.0114166	0.298539
Pulmonary hea	218792	T	G	0.0146809	0.167529
Pulmonary hea	218792	T	C	-0.0107288	0.665119
Pulmonary hea	218792	A	G	-0.0246758	0.049304
Pulmonary hea	218792	G	A	0.0134447	0.171589
Pulmonary hea	218792	G	A	-0.0116357	0.759827
Pulmonary hea	218792	T	G	-0.0181851	0.083179
Pulmonary hea	218792	C	A	0.0120084	0.364496
Pulmonary hea	218792	A	C	0.0544302	0.008242
Pulmonary hea	218792	T	C	0.0111347	0.657728
Pulmonary hea	218792	A	G	-0.0351148	0.021377
Pulmonary hea	218792	C	T	-0.0181174	0.082788
Pulmonary hea	218792	A	G	0.0178158	0.097718
Pulmonary hea	218792	C	T	0.0114362	0.71603
Pulmonary hea	218792	G	T	-0.0110872	0.645782
Pulmonary hea	218792	A	G	-0.0123919	0.465814
Pulmonary hea	218792	G	A	-0.0105236	0.339919
Pulmonary hea	218792	A	G	-0.0135133	0.166699
Pulmonary hea	218792	T	C	0.113396	0.003862
Pulmonary hea	218792	G	A	-0.0113177	0.286053
Pulmonary hea	218792	C	T	0.02003	0.074366

Pulmonary hea	218792	C	A	-0.0160909	0.149235
Pulmonary hea	218792	C	A	-0.0129805	0.186948
Pulmonary hea	218792	C	A	-0.0114094	0.441655
Pulmonary hea	218792	T	C	0.0135219	0.273909
Pulmonary hea	218792	T	C	0.0313937	0.036507
Pulmonary hea	218792	A	G	0.0109471	0.71427
Pulmonary hea	218792	G	A	-0.0467891	0.013105
Pulmonary hea	218792	C	T	-0.0335181	0.027757
Pulmonary hea	218792	T	G	-0.0187398	0.092425
Pulmonary hea	218792	A	C	0.0130804	0.182153
Pulmonary hea	218792	A	G	0.0139827	0.151287
Pulmonary hea	218792	C	T	0.00986771	0.478622
Pulmonary hea	218792	A	G	0.0102987	0.437758
Pulmonary hea	218792	A	G	0.0150639	0.130178
Pulmonary hea	218792	T	G	0.00972771	0.484233
Pulmonary hea	218792	A	G	-0.0102936	0.416147
Pulmonary hea	218792	T	C	-0.009853	0.417906
Pulmonary hea	218792	T	C	0.0180857	0.072984
Pulmonary hea	218792	A	C	0.0149971	0.16794
Pulmonary hea	218792	A	G	0.0156804	0.154461
Pulmonary hea	218792	A	C	0.0124993	0.186886
Pulmonary hea	218792	T	C	-0.0386416	0.016208
Pulmonary hea	218792	G	A	-0.0110104	0.2435
Pulmonary hea	218792	G	A	-0.0098554	0.641994
Pulmonary hea	218792	A	G	0.0867972	0.003383
Pulmonary hea	218792	A	G	-0.0132439	0.153233
Pulmonary hea	218792	A	G	-0.0095825	0.548637
Pulmonary hea	218792	G	A	0.0140134	0.782419
Pulmonary hea	218792	T	C	0.0196624	0.219436
Pulmonary hea	218792	C	T	0.00970902	0.380091
Pulmonary hea	218792	C	T	-0.0130453	0.200795
Pulmonary hea	218792	C	A	-0.0114495	0.243279
Pulmonary hea	218792	T	C	0.0154658	0.12919
Pulmonary hea	218792	A	G	0.0103554	0.373673
Pulmonary hea	218792	T	C	-0.0384693	0.017336
Pulmonary hea	218792	G	T	-0.0429541	0.013342
Pulmonary hea	218792	T	C	-0.018716	0.083064
Pulmonary hea	218792	G	A	0.0102993	0.698769
Pulmonary hea	218792	T	C	-0.0100751	0.663321
Pulmonary hea	218792	G	A	0.0139755	0.130449
Pulmonary hea	218792	C	T	0.0201622	0.064993
Pulmonary hea	218792	G	A	-0.0153369	0.109891

Pulmonary hea	218792	A	G	0.0107315	0.611738
Pulmonary hea	218792	T	C	0.0107705	0.33449
Pulmonary hea	218792	C	T	-0.0350603	0.019349
Pulmonary hea	218792	G	A	0.0108015	0.745173
Pulmonary hea	218792	T	C	0.0209748	0.079749
Pulmonary hea	218792	A	G	-0.0359277	0.018434
Pulmonary hea	218792	G	A	0.0160831	0.881571
Pulmonary hea	218792	C	T	0.0113964	0.239895
Pulmonary hea	218792	A	G	0.00969921	0.387517
Pulmonary hea	218792	G	T	-0.0204187	0.062116
Pulmonary hea	218792	T	C	-0.0113675	0.245752
Pulmonary hea	218792	G	T	-0.0175571	0.15565
Pulmonary hea	218792	C	A	-0.0300886	0.02677
Pulmonary hea	218792	C	A	0.0118268	0.365075
Pulmonary hea	218792	A	G	0.0300981	0.028151
Pulmonary hea	218792	T	C	-0.0113821	0.23522
Pulmonary hea	218792	T	C	0.0129274	0.172431
Pulmonary hea	218792	T	C	0.00992086	0.658049
Pulmonary hea	218792	T	G	-0.0207931	0.070424
Pulmonary hea	218792	A	G	-0.0106399	0.46467
Pulmonary hea	218792	C	T	0.0134611	0.158973
Pulmonary hea	218792	T	C	-0.009463	0.51929
Pulmonary hea	218792	T	C	0.0123158	0.230477
Pulmonary hea	218792	G	A	0.011581	0.769457
Pulmonary hea	218792	T	C	0.0946143	0.003807
Pulmonary hea	218792	G	A	-0.0107992	0.286147
Pulmonary hea	218792	T	C	-0.0287129	0.029919
Pulmonary hea	218792	G	T	-0.0331207	0.025544
Pulmonary hea	218792	C	A	-0.0132066	0.186513
Pulmonary hea	218792	C	T	-0.0231955	0.046759
Pulmonary hea	218792	A	G	0.0135202	0.165706
Pulmonary hea	218792	G	A	0.0159377	0.106329
Pulmonary hea	218792	T	C	0.0148569	0.274855
Pulmonary hea	218792	A	G	-0.0154582	0.113504
Pulmonary hea	218792	T	C	-0.0098524	0.370453
Pulmonary hea	218792	A	G	0.0118368	0.714735
Pulmonary hea	218792	A	G	0.0271431	0.031972
Pulmonary hea	218792	C	T	-0.030081	0.027798
Pulmonary hea	218792	T	C	-0.0102176	0.424276
Pulmonary hea	218792	T	G	-0.0176865	0.091868
Pulmonary hea	218792	T	C	-0.023713	0.04104
Pulmonary hea	218792	A	C	-0.0111924	0.368826

Pulmonary hea	218792	G	A	-0.0162463	0.114428
Pulmonary hea	218792	A	G	-0.0195322	0.085011
Pulmonary hea	218792	A	G	-0.0217207	0.063928
Pulmonary hea	218792	T	C	0.0388327	0.01703
Pulmonary hea	218792	T	C	-0.0420185	0.017318
Pulmonary hea	218792	T	C	-0.0387119	0.023291
Pulmonary hea	218792	T	C	0.0132343	0.212894
Pulmonary hea	218792	G	T	0.0118316	0.241258
Pulmonary hea	218792	G	A	-0.0200821	0.069722
Pulmonary hea	218792	C	T	-0.0581919	0.010194
Pulmonary hea	218792	C	T	-0.0287918	0.032879
Pulmonary hea	218792	T	C	-0.028982	0.032703
Pulmonary hea	218792	T	C	-0.0120578	0.230187
Pulmonary hea	218792	C	T	0.0460816	0.011858
Pulmonary hea	218792	A	G	0.027384	0.036362
Pulmonary hea	218792	T	C	-0.035513	0.020331
Pulmonary hea	218792	A	G	0.0117626	0.377409
Pulmonary hea	218792	C	A	-0.0124891	0.311303
Pulmonary hea	218792	T	C	-0.0258626	0.040057
Pulmonary hea	218792	T	C	0.0105209	0.657728
Pulmonary hea	218792	T	C	-0.0127818	0.190704
Pulmonary hea	218792	G	A	0.0417032	0.017923
Pulmonary hea	218792	C	T	-0.0494714	0.010542
Pulmonary hea	218792	T	G	-0.0107374	0.419106
Pulmonary hea	218792	A	G	-0.032334	0.028942
Pulmonary hea	218792	G	A	0.0420186	0.017845
Pulmonary hea	218792	A	G	-0.0164801	0.109299
Pulmonary hea	218792	C	T	0.00990463	0.425557
Pulmonary hea	218792	A	G	0.0573849	0.008598
Pulmonary hea	218792	T	C	-0.0355946	0.018977
Pulmonary hea	218792	A	G	-0.0405505	0.015591
Pulmonary hea	218792	A	C	-0.0107806	0.286782
Pulmonary hea	218792	G	A	0.0289547	0.030344
Pulmonary hea	218792	T	C	0.0096854	0.565309
Pulmonary hea	218792	C	T	0.0101453	0.33166
Pulmonary hea	218792	C	T	-0.0104559	0.327404
Pulmonary hea	218792	G	A	-0.0268806	0.034508
Pulmonary hea	218792	T	C	-0.0098819	0.455668
Pulmonary hea	218792	A	G	0.0113885	0.285878
Valvular heart c	218792	A	G	0.0150639	0.130178
Valvular heart c	218792	T	G	0.00972771	0.484233
Valvular heart c	218792	A	G	-0.0102936	0.416147

Valvular heart c	218792	T	C	-0.009853	0.417906
Valvular heart c	218792	T	C	0.0180857	0.072984
Valvular heart c	218792	A	C	0.0149971	0.16794
Valvular heart c	218792	A	G	0.0156804	0.154461
Valvular heart c	218792	A	C	0.0124993	0.186886
Valvular heart c	218792	T	C	-0.0386416	0.016208
Valvular heart c	218792	G	A	-0.0110104	0.2435
Valvular heart c	218792	G	A	-0.0098554	0.641994
Valvular heart c	218792	A	G	0.0867972	0.003383
Valvular heart c	218792	A	G	-0.0132439	0.153233
Valvular heart c	218792	A	G	-0.0095825	0.548637
Valvular heart c	218792	G	A	0.0140134	0.782419
Valvular heart c	218792	T	C	0.0196624	0.219436
Valvular heart c	218792	C	T	0.00970902	0.380091
Valvular heart c	218792	C	T	-0.0130453	0.200795
Valvular heart c	218792	C	A	-0.0114495	0.243279
Valvular heart c	218792	T	C	0.0154658	0.12919
Valvular heart c	218792	A	G	0.0103554	0.373673
Valvular heart c	218792	T	C	-0.0384693	0.017336
Valvular heart c	218792	G	T	-0.0429541	0.013342
Valvular heart c	218792	T	C	-0.018716	0.083064
Valvular heart c	218792	G	A	0.0102993	0.698769
Valvular heart c	218792	T	C	-0.0100751	0.663321
Valvular heart c	218792	G	A	0.0139755	0.130449
Valvular heart c	218792	C	T	0.0201622	0.064993
Valvular heart c	218792	G	A	-0.0153369	0.109891
Valvular heart c	218792	A	G	0.0107315	0.611738
Valvular heart c	218792	T	C	0.0107705	0.33449
Valvular heart c	218792	C	T	-0.0350603	0.019349
Valvular heart c	218792	G	A	0.0108015	0.745173
Valvular heart c	218792	T	C	0.0209748	0.079749
Valvular heart c	218792	A	G	-0.0359277	0.018434
Valvular heart c	218792	G	A	0.0160831	0.881571
Valvular heart c	218792	C	T	0.0113964	0.239895
Valvular heart c	218792	A	G	0.00969921	0.387517
Valvular heart c	218792	G	T	-0.0204187	0.062116
Valvular heart c	218792	T	C	-0.0113675	0.245752
Valvular heart c	218792	G	T	-0.0175571	0.15565
Valvular heart c	218792	C	A	-0.0300886	0.02677
Valvular heart c	218792	C	A	0.0118268	0.365075
Valvular heart c	218792	A	G	0.0300981	0.028151
Valvular heart c	218792	T	C	-0.0113821	0.23522

Valvular heart c	218792	T	C	0.0129274	0.172431
Valvular heart c	218792	T	C	0.00992086	0.658049
Valvular heart c	218792	T	G	-0.0207931	0.070424
Valvular heart c	218792	A	G	-0.0106399	0.46467
Valvular heart c	218792	C	T	0.0134611	0.158973
Valvular heart c	218792	T	C	-0.009463	0.51929
Valvular heart c	218792	T	C	0.0123158	0.230477
Valvular heart c	218792	G	A	0.011581	0.769457
Valvular heart c	218792	T	C	0.0946143	0.003807
Valvular heart c	218792	G	A	-0.0107992	0.286147
Valvular heart c	218792	T	C	-0.0287129	0.029919
Valvular heart c	218792	G	T	-0.0331207	0.025544
Valvular heart c	218792	C	A	-0.0132066	0.186513
Valvular heart c	218792	C	T	-0.0231955	0.046759
Valvular heart c	218792	A	G	0.0135202	0.165706
Valvular heart c	218792	G	A	0.0159377	0.106329
Valvular heart c	218792	T	C	0.0148569	0.274855
Valvular heart c	218792	A	G	-0.0154582	0.113504
Valvular heart c	218792	T	C	-0.0098524	0.370453
Valvular heart c	218792	A	G	0.0118368	0.714735
Valvular heart c	218792	A	G	0.0271431	0.031972
Valvular heart c	218792	C	T	-0.030081	0.027798
Valvular heart c	218792	T	C	-0.0102176	0.424276
Valvular heart c	218792	T	G	-0.0176865	0.091868
Valvular heart c	218792	T	C	-0.023713	0.04104
Valvular heart c	218792	A	C	-0.0111924	0.368826
Valvular heart c	218792	A	G	-0.0193433	0.085011
Valvular heart c	218792	G	T	0.0533208	0.010465
Valvular heart c	218792	G	A	-0.0480395	0.011699
Valvular heart c	218792	T	G	0.0127384	0.187616
Valvular heart c	218792	A	G	-0.0126249	0.283075
Valvular heart c	218792	A	C	0.0104397	0.347049
Valvular heart c	218792	G	A	0.012725	0.781731
Valvular heart c	218792	T	C	0.0216661	0.212894
Valvular heart c	218792	C	T	-0.0139685	0.201776
Valvular heart c	218792	A	G	0.0122914	0.374311
Valvular heart c	218792	A	C	0.0123705	0.500143
Valvular heart c	218792	C	T	0.0216856	0.064265
Valvular heart c	218792	A	C	-0.0139275	0.151277
Valvular heart c	218792	G	T	-0.0434289	0.013488
Valvular heart c	218792	C	T	-0.0371072	0.018141
Valvular heart c	218792	G	T	-0.0114166	0.298539

Valvular heart c	218792	T	G	0.0146809	0.167529
Valvular heart c	218792	T	C	-0.0107288	0.665119
Valvular heart c	218792	A	G	-0.0246758	0.049304
Valvular heart c	218792	G	A	0.0134447	0.171589
Valvular heart c	218792	G	A	-0.0116357	0.759827
Valvular heart c	218792	T	G	-0.0181851	0.083179
Valvular heart c	218792	C	A	0.0120084	0.364496
Valvular heart c	218792	A	C	0.0544302	0.008242
Valvular heart c	218792	T	C	0.0111347	0.657728
Valvular heart c	218792	A	G	-0.0351148	0.021377
Valvular heart c	218792	C	T	-0.0181174	0.082788
Valvular heart c	218792	A	G	0.0178158	0.097718
Valvular heart c	218792	C	T	0.0114362	0.71603
Valvular heart c	218792	G	T	-0.0110872	0.645782
Valvular heart c	218792	A	G	-0.0123919	0.465814
Valvular heart c	218792	G	A	-0.0105236	0.339919
Valvular heart c	218792	A	G	-0.0135133	0.166699
Valvular heart c	218792	T	C	0.113396	0.003862
Valvular heart c	218792	G	A	-0.0113177	0.286053
Valvular heart c	218792	C	T	0.02003	0.074366
Valvular heart c	218792	C	A	-0.0160909	0.149235
Valvular heart c	218792	C	A	-0.0129805	0.186948
Valvular heart c	218792	C	A	-0.0114094	0.441655
Valvular heart c	218792	T	C	0.0135219	0.273909
Valvular heart c	218792	T	C	0.0313937	0.036507
Valvular heart c	218792	A	G	0.0109471	0.71427
Valvular heart c	218792	G	A	-0.0467891	0.013105
Valvular heart c	218792	C	T	-0.0335181	0.027757
Valvular heart c	218792	T	G	-0.0187398	0.092425
Valvular heart c	218792	A	C	0.0130804	0.182153
Valvular heart c	218792	A	G	0.0139827	0.151287
Valvular heart c	218792	C	T	0.00986771	0.478622
Valvular heart c	218792	A	G	0.0102987	0.437758
Valvular heart c	218792	G	A	-0.0162463	0.114428
Valvular heart c	218792	A	G	-0.0195322	0.085011
Valvular heart c	218792	A	G	-0.0217207	0.063928
Valvular heart c	218792	T	C	0.0388327	0.01703
Valvular heart c	218792	T	C	-0.0420185	0.017318
Valvular heart c	218792	T	C	-0.0387119	0.023291
Valvular heart c	218792	T	C	0.0132343	0.212894
Valvular heart c	218792	G	T	0.0118316	0.241258
Valvular heart c	218792	G	A	-0.0200821	0.069722

Valvular heart c	218792	C	T	-0.0581919	0.010194
Valvular heart c	218792	C	T	-0.0287918	0.032879
Valvular heart c	218792	T	C	-0.028982	0.032703
Valvular heart c	218792	T	C	-0.0120578	0.230187
Valvular heart c	218792	C	T	0.0460816	0.011858
Valvular heart c	218792	A	G	0.027384	0.036362
Valvular heart c	218792	T	C	-0.035513	0.020331
Valvular heart c	218792	A	G	0.0117626	0.377409
Valvular heart c	218792	C	A	-0.0124891	0.311303
Valvular heart c	218792	T	C	-0.0258626	0.040057
Valvular heart c	218792	T	C	0.0105209	0.657728
Valvular heart c	218792	T	C	-0.0127818	0.190704
Valvular heart c	218792	G	A	0.0417032	0.017923
Valvular heart c	218792	C	T	-0.0494714	0.010542
Valvular heart c	218792	T	G	-0.0107374	0.419106
Valvular heart c	218792	A	G	-0.032334	0.028942
Valvular heart c	218792	G	A	0.0420186	0.017845
Valvular heart c	218792	A	G	-0.0164801	0.109299
Valvular heart c	218792	C	T	0.00990463	0.425557
Valvular heart c	218792	A	G	0.0573849	0.008598
Valvular heart c	218792	T	C	-0.0355946	0.018977
Valvular heart c	218792	A	G	-0.0405505	0.015591
Valvular heart c	218792	A	C	-0.0107806	0.286782
Valvular heart c	218792	G	A	0.0289547	0.030344
Valvular heart c	218792	T	C	0.0096854	0.565309
Valvular heart c	218792	C	T	0.0101453	0.33166
Valvular heart c	218792	C	T	-0.0104559	0.327404
Valvular heart c	218792	G	A	-0.0268806	0.034508
Valvular heart c	218792	T	C	-0.0098819	0.455668
Valvular heart c	218792	A	G	0.0113885	0.285878
Coronary heart	184305	A	G	0.0150639	0.130178
Coronary heart	184305	T	G	0.00972771	0.484233
Coronary heart	184305	A	G	-0.0102936	0.416147
Coronary heart	184305	T	C	-0.009853	0.417906
Coronary heart	184305	T	C	0.0180857	0.072984
Coronary heart	184305	A	C	0.0149971	0.16794
Coronary heart	184305	A	G	0.0156804	0.154461
Coronary heart	184305	A	C	0.0124993	0.186886
Coronary heart	184305	T	C	-0.0386416	0.016208
Coronary heart	184305	G	A	-0.0110104	0.2435
Coronary heart	184305	G	A	-0.0098554	0.641994
Coronary heart	184305	A	G	-0.0132439	0.153233

Coronary heart	184305	A	G	-0.0095825	0.548637
Coronary heart	184305	G	A	0.0140134	0.782419
Coronary heart	184305	T	C	0.0196624	0.219436
Coronary heart	184305	C	T	0.00970902	0.380091
Coronary heart	184305	C	T	-0.0130453	0.200795
Coronary heart	184305	C	A	-0.0114495	0.243279
Coronary heart	184305	T	C	0.0154658	0.12919
Coronary heart	184305	A	G	0.0103554	0.373673
Coronary heart	184305	T	C	-0.0384693	0.017336
Coronary heart	184305	T	C	-0.018716	0.083064
Coronary heart	184305	G	A	0.0102993	0.698769
Coronary heart	184305	T	C	-0.0100751	0.663321
Coronary heart	184305	G	A	0.0139755	0.130449
Coronary heart	184305	C	T	0.0201622	0.064993
Coronary heart	184305	G	A	-0.0153369	0.109891
Coronary heart	184305	A	G	0.0107315	0.611738
Coronary heart	184305	T	C	0.0107705	0.33449
Coronary heart	184305	C	T	-0.0350603	0.019349
Coronary heart	184305	G	A	0.0108015	0.745173
Coronary heart	184305	T	C	0.0209748	0.079749
Coronary heart	184305	A	G	-0.0359277	0.018434
Coronary heart	184305	G	A	0.0160831	0.881571
Coronary heart	184305	C	T	0.0113964	0.239895
Coronary heart	184305	A	G	0.00969921	0.387517
Coronary heart	184305	G	T	-0.0204187	0.062116
Coronary heart	184305	T	C	-0.0113675	0.245752
Coronary heart	184305	G	T	-0.0175571	0.15565
Coronary heart	184305	C	A	0.0118268	0.365075
Coronary heart	184305	A	G	0.0300981	0.028151
Coronary heart	184305	T	C	-0.0113821	0.23522
Coronary heart	184305	T	C	0.0129274	0.172431
Coronary heart	184305	T	C	0.00992086	0.658049
Coronary heart	184305	T	G	-0.0207931	0.070424
Coronary heart	184305	T	C	0.0120277	0.270243
Coronary heart	184305	A	G	-0.0106399	0.46467
Coronary heart	184305	C	T	0.0134611	0.158973
Coronary heart	184305	T	C	-0.009463	0.51929
Coronary heart	184305	T	C	0.0123158	0.230477
Coronary heart	184305	G	A	0.011581	0.769457
Coronary heart	184305	T	C	0.0946143	0.003807
Coronary heart	184305	G	A	-0.0107992	0.286147
Coronary heart	184305	T	C	-0.0287129	0.029919

Coronary heart	184305	G	T	-0.0331207	0.025544
Coronary heart	184305	C	A	-0.0132066	0.186513
Coronary heart	184305	C	T	-0.0231955	0.046759
Coronary heart	184305	A	G	0.0135202	0.165706
Coronary heart	184305	G	A	0.0159377	0.106329
Coronary heart	184305	T	C	0.0148569	0.274855
Coronary heart	184305	A	G	-0.0154582	0.113504
Coronary heart	184305	T	C	-0.0098524	0.370453
Coronary heart	184305	A	G	0.0118368	0.714735
Coronary heart	184305	A	G	0.0271431	0.031972
Coronary heart	184305	C	T	-0.030081	0.027798
Coronary heart	184305	T	C	-0.0102176	0.424276
Coronary heart	184305	C	T	0.0369793	0.027316
Coronary heart	184305	T	G	-0.0176865	0.091868
Coronary heart	184305	T	C	-0.023713	0.04104
Coronary heart	184305	A	C	-0.0111924	0.368826
Coronary heart	184305	G	A	-0.0162463	0.114428
Coronary heart	184305	A	G	-0.0195322	0.085011
Coronary heart	184305	A	G	-0.0217207	0.063928
Coronary heart	184305	T	C	0.0388327	0.01703
Coronary heart	184305	T	C	-0.0420185	0.017318
Coronary heart	184305	T	C	-0.0387119	0.023291
Coronary heart	184305	T	C	0.0132343	0.212894
Coronary heart	184305	G	T	0.0118316	0.241258
Coronary heart	184305	G	A	-0.0200821	0.069722
Coronary heart	184305	C	T	-0.0287918	0.032879
Coronary heart	184305	T	C	-0.028982	0.032703
Coronary heart	184305	T	C	-0.0120578	0.230187
Coronary heart	184305	C	T	0.0460816	0.011858
Coronary heart	184305	A	G	0.027384	0.036362
Coronary heart	184305	T	C	-0.035513	0.020331
Coronary heart	184305	A	G	0.0117626	0.377409
Coronary heart	184305	C	A	-0.0124891	0.311303
Coronary heart	184305	T	C	-0.0258626	0.040057
Coronary heart	184305	T	C	0.0105209	0.657728
Coronary heart	184305	T	C	-0.0127818	0.190704
Coronary heart	184305	G	A	0.0417032	0.017923
Coronary heart	184305	C	T	-0.0494714	0.010542
Coronary heart	184305	T	G	-0.0107374	0.419106
Coronary heart	184305	A	G	-0.032334	0.028942
Coronary heart	184305	G	A	0.0420186	0.017845
Coronary heart	184305	A	G	-0.0164801	0.109299

Coronary heart	184305	C	T	0.00990463	0.425557
Coronary heart	184305	A	G	0.0573849	0.008598
Coronary heart	184305	T	C	-0.0355946	0.018977
Coronary heart	184305	A	G	-0.0405505	0.015591
Coronary heart	184305	A	C	-0.0107806	0.286782
Coronary heart	184305	G	A	0.0289547	0.030344
Coronary heart	184305	T	C	0.0096854	0.565309
Coronary heart	184305	C	T	0.0101453	0.33166
Coronary heart	184305	C	T	-0.0104559	0.327404
Coronary heart	184305	G	A	-0.0268806	0.034508
Coronary heart	184305	T	C	-0.0098819	0.455668
Coronary heart	184305	A	G	0.0113885	0.285878
Coronary heart	184305	A	G	-0.0193433	0.085011
Coronary heart	184305	A	G	-0.0130416	0.257614
Coronary heart	184305	G	T	0.0533208	0.010465
Coronary heart	184305	G	A	-0.0480395	0.011699
Coronary heart	184305	T	G	0.0127384	0.187616
Coronary heart	184305	A	G	-0.0126249	0.283075
Coronary heart	184305	A	C	0.0104397	0.347049
Coronary heart	184305	G	A	0.012725	0.781731
Coronary heart	184305	T	C	0.0216661	0.212894
Coronary heart	184305	G	A	0.0111409	0.287274
Coronary heart	184305	C	T	-0.0139685	0.201776
Coronary heart	184305	A	G	0.0122914	0.374311
Coronary heart	184305	A	C	0.0123705	0.500143
Coronary heart	184305	C	T	0.0216856	0.064265
Coronary heart	184305	A	C	-0.0139275	0.151277
Coronary heart	184305	C	T	-0.0371072	0.018141
Coronary heart	184305	G	T	-0.0114166	0.298539
Coronary heart	184305	T	G	0.0146809	0.167529
Coronary heart	184305	T	C	-0.0107288	0.665119
Coronary heart	184305	A	G	-0.0246758	0.049304
Coronary heart	184305	G	A	0.0134447	0.171589
Coronary heart	184305	G	A	-0.0116357	0.759827
Coronary heart	184305	T	G	-0.0181851	0.083179
Coronary heart	184305	C	A	0.0120084	0.364496
Coronary heart	184305	A	C	0.0544302	0.008242
Coronary heart	184305	T	C	0.0111347	0.657728
Coronary heart	184305	A	G	-0.0351148	0.021377
Coronary heart	184305	C	T	-0.0181174	0.082788
Coronary heart	184305	A	G	0.0178158	0.097718
Coronary heart	184305	C	T	0.0114362	0.71603

Coronary heart	184305	G	T	-0.0110872	0.645782
Coronary heart	184305	A	G	-0.0123919	0.465814
Coronary heart	184305	G	A	-0.0105236	0.339919
Coronary heart	184305	A	G	-0.0135133	0.166699
Coronary heart	184305	T	C	0.113396	0.003862
Coronary heart	184305	G	A	-0.0113177	0.286053
Coronary heart	184305	C	T	0.02003	0.074366
Coronary heart	184305	C	A	-0.0160909	0.149235
Coronary heart	184305	C	A	-0.0129805	0.186948
Coronary heart	184305	C	A	-0.0114094	0.441655
Coronary heart	184305	T	C	0.0135219	0.273909
Coronary heart	184305	T	C	0.0313937	0.036507
Coronary heart	184305	A	G	0.0109471	0.71427
Coronary heart	184305	G	A	-0.0467891	0.013105
Coronary heart	184305	C	T	-0.0335181	0.027757
Coronary heart	184305	C	T	-0.0317179	0.026758
Coronary heart	184305	T	G	-0.0187398	0.092425
Coronary heart	184305	A	C	0.0130804	0.182153
Coronary heart	184305	C	T	0.00986771	0.478622
Coronary heart	184305	A	G	0.0102987	0.437758
Myocardial infa	171875	A	G	0.0150639	0.130178
Myocardial infa	171875	T	G	0.00972771	0.484233
Myocardial infa	171875	A	G	-0.0102936	0.416147
Myocardial infa	171875	T	C	-0.009853	0.417906
Myocardial infa	171875	T	C	0.0180857	0.072984
Myocardial infa	171875	A	C	0.0149971	0.16794
Myocardial infa	171875	A	G	0.0156804	0.154461
Myocardial infa	171875	A	C	0.0124993	0.186886
Myocardial infa	171875	T	C	-0.0386416	0.016208
Myocardial infa	171875	G	A	-0.0110104	0.2435
Myocardial infa	171875	G	A	-0.0098554	0.641994
Myocardial infa	171875	A	G	-0.0132439	0.153233
Myocardial infa	171875	A	G	-0.0095825	0.548637
Myocardial infa	171875	G	A	0.0140134	0.782419
Myocardial infa	171875	T	C	0.0196624	0.219436
Myocardial infa	171875	C	T	0.00970902	0.380091
Myocardial infa	171875	C	T	-0.0130453	0.200795
Myocardial infa	171875	C	A	-0.0114495	0.243279
Myocardial infa	171875	T	C	0.0154658	0.12919
Myocardial infa	171875	A	G	0.0103554	0.373673
Myocardial infa	171875	T	C	-0.0384693	0.017336
Myocardial infa	171875	T	C	-0.018716	0.083064

Myocardial infa	171875	G	A	0.0102993	0.698769
Myocardial infa	171875	T	C	-0.0100751	0.663321
Myocardial infa	171875	G	A	0.0139755	0.130449
Myocardial infa	171875	C	T	0.0201622	0.064993
Myocardial infa	171875	G	A	-0.0153369	0.109891
Myocardial infa	171875	A	G	0.0107315	0.611738
Myocardial infa	171875	T	C	0.0107705	0.33449
Myocardial infa	171875	C	T	-0.0350603	0.019349
Myocardial infa	171875	G	A	0.0108015	0.745173
Myocardial infa	171875	T	C	0.0209748	0.079749
Myocardial infa	171875	A	G	-0.0359277	0.018434
Myocardial infa	171875	G	A	0.0160831	0.881571
Myocardial infa	171875	C	T	0.0113964	0.239895
Myocardial infa	171875	A	G	0.00969921	0.387517
Myocardial infa	171875	G	T	-0.0204187	0.062116
Myocardial infa	171875	T	C	-0.0113675	0.245752
Myocardial infa	171875	G	T	-0.0175571	0.15565
Myocardial infa	171875	C	A	0.0118268	0.365075
Myocardial infa	171875	A	G	0.0300981	0.028151
Myocardial infa	171875	T	C	-0.0113821	0.23522
Myocardial infa	171875	T	C	0.0129274	0.172431
Myocardial infa	171875	T	C	0.00992086	0.658049
Myocardial infa	171875	T	G	-0.0207931	0.070424
Myocardial infa	171875	T	C	0.0120277	0.270243
Myocardial infa	171875	A	G	-0.0106399	0.46467
Myocardial infa	171875	C	T	0.0134611	0.158973
Myocardial infa	171875	T	C	-0.009463	0.51929
Myocardial infa	171875	T	C	0.0123158	0.230477
Myocardial infa	171875	G	A	0.011581	0.769457
Myocardial infa	171875	T	C	0.0946143	0.003807
Myocardial infa	171875	G	A	-0.0107992	0.286147
Myocardial infa	171875	T	C	-0.0287129	0.029919
Myocardial infa	171875	G	T	-0.0331207	0.025544
Myocardial infa	171875	C	A	-0.0132066	0.186513
Myocardial infa	171875	C	T	-0.0231955	0.046759
Myocardial infa	171875	A	G	0.0135202	0.165706
Myocardial infa	171875	G	A	0.0159377	0.106329
Myocardial infa	171875	T	C	0.0148569	0.274855
Myocardial infa	171875	A	G	-0.0154582	0.113504
Myocardial infa	171875	T	C	-0.0098524	0.370453
Myocardial infa	171875	A	G	0.0118368	0.714735
Myocardial infa	171875	A	G	0.0271431	0.031972

Myocardial infa	171875	C	T	-0.030081	0.027798
Myocardial infa	171875	T	C	-0.0102176	0.424276
Myocardial infa	171875	C	T	0.0369793	0.027316
Myocardial infa	171875	T	G	-0.0176865	0.091868
Myocardial infa	171875	T	C	-0.023713	0.04104
Myocardial infa	171875	A	C	-0.0111924	0.368826
Myocardial infa	171875	G	A	-0.0162463	0.114428
Myocardial infa	171875	A	G	-0.0195322	0.085011
Myocardial infa	171875	A	G	-0.0217207	0.063928
Myocardial infa	171875	T	C	0.0388327	0.01703
Myocardial infa	171875	T	C	-0.0420185	0.017318
Myocardial infa	171875	T	C	-0.0387119	0.023291
Myocardial infa	171875	T	C	0.0132343	0.212894
Myocardial infa	171875	G	T	0.0118316	0.241258
Myocardial infa	171875	G	A	-0.0200821	0.069722
Myocardial infa	171875	C	T	-0.0287918	0.032879
Myocardial infa	171875	T	C	-0.028982	0.032703
Myocardial infa	171875	T	C	-0.0120578	0.230187
Myocardial infa	171875	C	T	0.0460816	0.011858
Myocardial infa	171875	A	G	0.027384	0.036362
Myocardial infa	171875	T	C	-0.035513	0.020331
Myocardial infa	171875	A	G	0.0117626	0.377409
Myocardial infa	171875	C	A	-0.0124891	0.311303
Myocardial infa	171875	T	C	-0.0258626	0.040057
Myocardial infa	171875	T	C	0.0105209	0.657728
Myocardial infa	171875	T	C	-0.0127818	0.190704
Myocardial infa	171875	G	A	0.0417032	0.017923
Myocardial infa	171875	C	T	-0.0494714	0.010542
Myocardial infa	171875	T	G	-0.0107374	0.419106
Myocardial infa	171875	A	G	-0.032334	0.028942
Myocardial infa	171875	G	A	0.0420186	0.017845
Myocardial infa	171875	A	G	-0.0164801	0.109299
Myocardial infa	171875	A	G	-0.0193433	0.085011
Myocardial infa	171875	A	G	-0.0130416	0.257614
Myocardial infa	171875	G	T	0.0533208	0.010465
Myocardial infa	171875	G	A	-0.0480395	0.011699
Myocardial infa	171875	T	G	0.0127384	0.187616
Myocardial infa	171875	A	G	-0.0126249	0.283075
Myocardial infa	171875	A	C	0.0104397	0.347049
Myocardial infa	171875	G	A	0.012725	0.781731
Myocardial infa	171875	T	C	0.0216661	0.212894
Myocardial infa	171875	G	A	0.0111409	0.287274

Myocardial infa	171875	C	T	-0.0139685	0.201776
Myocardial infa	171875	A	G	0.0122914	0.374311
Myocardial infa	171875	A	C	0.0123705	0.500143
Myocardial infa	171875	C	T	0.0216856	0.064265
Myocardial infa	171875	A	C	-0.0139275	0.151277
Myocardial infa	171875	C	T	-0.0371072	0.018141
Myocardial infa	171875	G	T	-0.0114166	0.298539
Myocardial infa	171875	T	G	0.0146809	0.167529
Myocardial infa	171875	T	C	-0.0107288	0.665119
Myocardial infa	171875	A	G	-0.0246758	0.049304
Myocardial infa	171875	G	A	0.0134447	0.171589
Myocardial infa	171875	G	A	-0.0116357	0.759827
Myocardial infa	171875	T	G	-0.0181851	0.083179
Myocardial infa	171875	C	A	0.0120084	0.364496
Myocardial infa	171875	A	C	0.0544302	0.008242
Myocardial infa	171875	T	C	0.0111347	0.657728
Myocardial infa	171875	A	G	-0.0351148	0.021377
Myocardial infa	171875	C	T	-0.0181174	0.082788
Myocardial infa	171875	A	G	0.0178158	0.097718
Myocardial infa	171875	C	T	0.0114362	0.71603
Myocardial infa	171875	G	T	-0.0110872	0.645782
Myocardial infa	171875	A	G	-0.0123919	0.465814
Myocardial infa	171875	G	A	-0.0105236	0.339919
Myocardial infa	171875	A	G	-0.0135133	0.166699
Myocardial infa	171875	T	C	0.113396	0.003862
Myocardial infa	171875	G	A	-0.0113177	0.286053
Myocardial infa	171875	C	T	0.02003	0.074366
Myocardial infa	171875	C	A	-0.0160909	0.149235
Myocardial infa	171875	C	A	-0.0129805	0.186948
Myocardial infa	171875	C	A	-0.0114094	0.441655
Myocardial infa	171875	T	C	0.0135219	0.273909
Myocardial infa	171875	T	C	0.0313937	0.036507
Myocardial infa	171875	A	G	0.0109471	0.71427
Myocardial infa	171875	C	T	-0.0335181	0.027757
Myocardial infa	171875	C	T	-0.0317179	0.026758
Myocardial infa	171875	T	G	-0.0187398	0.092425
Myocardial infa	171875	A	C	0.0130804	0.182153
Myocardial infa	171875	C	T	0.00986771	0.478622
Myocardial infa	171875	A	G	0.0102987	0.437758
Myocardial infa	171875	C	T	0.00990463	0.425557
Myocardial infa	171875	A	G	0.0573849	0.008598
Myocardial infa	171875	T	C	-0.0355946	0.018977

Myocardial infa	171875	A	G	-0.0405505	0.015591
Myocardial infa	171875	A	C	-0.0107806	0.286782
Myocardial infa	171875	G	A	0.0289547	0.030344
Myocardial infa	171875	T	C	0.0096854	0.565309
Myocardial infa	171875	C	T	0.0101453	0.33166
Myocardial infa	171875	C	T	-0.0104559	0.327404
Myocardial infa	171875	G	A	-0.0268806	0.034508
Myocardial infa	171875	T	C	-0.0098819	0.455668
Myocardial infa	171875	A	G	0.0113885	0.285878

SE	P-value	Proxy-SNP
0.00304795	7.70E-07	-
0.00204522	2.00E-06	-
0.00208179	7.60E-07	-
0.00210846	3.00E-06	-
0.00395544	4.80E-06	-
0.00273431	4.10E-08	-
0.00283547	3.20E-08	-
0.00263194	2.00E-06	-
0.00834142	3.60E-06	-
0.00238685	4.00E-06	-
0.00213843	4.10E-06	-
0.00284617	3.30E-06	-
0.00206171	3.40E-06	-
0.00249759	2.00E-08	-
0.00243611	7.00E-16	-
0.00212158	4.70E-06	-
0.00257421	4.00E-07	-
0.00239699	1.80E-06	-
0.00307844	5.10E-07	-
0.00211393	9.60E-07	-
0.00794035	1.30E-06	-
0.0038227	9.80E-07	-
0.00223922	4.20E-06	-
0.00219189	4.30E-06	-
0.00304505	4.40E-06	-
0.0041442	1.10E-06	-
0.00330989	3.60E-06	-
0.00216058	6.80E-07	-
0.00218214	8.00E-07	-
0.00742849	2.40E-06	-
0.00235154	4.40E-06	-
0.00377883	2.80E-08	-
0.00783716	4.60E-06	-
0.00318004	4.20E-07	-
0.00241321	2.30E-06	-
0.00210349	4.00E-06	-
0.00428631	1.90E-06	-
0.00237872	1.80E-06	-
0.00285444	7.70E-10	-
0.00639175	2.50E-06	-
0.00219366	7.00E-08	-

0.00618418	1.10E-06	-
0.00241176	2.40E-06	-
0.00275083	2.60E-06	-
0.00215778	4.30E-06	-
0.00438464	2.10E-06	-
0.00243539	7.90E-07	-
0.00207826	3.10E-07	-
0.00279712	1.50E-06	-
0.00206188	4.40E-06	-
0.00243998	4.50E-07	-
0.00243102	1.90E-06	-
0.0183509	2.50E-07	-
0.00228306	2.20E-06	-
0.00600813	1.80E-06	-
0.00648004	3.20E-07	-
0.0026301	5.10E-07	-
0.00488467	2.00E-06	-
0.00276029	9.70E-07	-
0.00337812	2.40E-06	-
0.00229517	9.60E-11	-
0.00334203	3.70E-06	-
0.00214122	4.20E-06	-
0.00227307	1.90E-07	-
0.00585868	3.60E-06	-
0.00627021	1.60E-06	-
0.00207188	8.20E-07	-
0.00626977	3.70E-09	-
0.0036924	1.70E-06	-
0.0051799	4.70E-06	-
0.00212721	1.40E-07	-
0.00340904	1.90E-06	-
0.00409857	1.90E-06	-
0.00447256	1.20E-06	-
0.00836887	3.50E-06	-
0.008321	4.40E-07	-
0.00738282	1.60E-07	-
0.00261169	4.00E-07	-
0.00253557	3.10E-06	-
0.0043472	3.80E-06	-
0.00611452	2.50E-06	-
0.00629425	4.10E-06	-
0.00262368	4.30E-06	-

0.0100244	4.30E-06	-
0.00578149	2.20E-06	-
0.00766936	3.60E-06	-
0.0022683	2.20E-07	-
0.00235748	1.20E-07	-
0.00554552	3.10E-06	-
0.00228303	4.10E-06	-
0.00276984	3.90E-06	-
0.00876048	1.90E-06	-
0.0105999	3.10E-06	-
0.00220019	1.10E-06	-
0.00646144	5.60E-07	-
0.00823522	3.40E-07	-
0.00350209	2.50E-06	-
0.00406633	2.00E-06	-
0.00249815	1.80E-07	-
0.0108609	9.10E-07	-
0.0100662	1.80E-06	-
0.0027732	4.40E-06	-
0.00243151	2.10E-07	-
0.00226473	4.00E-06	-
0.00262039	1.20E-06	-
0.00259111	6.20E-17	-
0.00239094	3.20E-06	-
0.0026994	2.30E-07	-
0.00221931	3.10E-08	-
0.00214859	8.50E-09	-
0.00438434	7.60E-07	-
0.00300906	3.70E-06	-
0.00804635	4.00E-06	-
0.00235647	1.30E-06	-
0.0028777	3.40E-07	-
0.00230502	3.20E-06	-
0.00534513	3.90E-06	-
0.0028931	3.40E-06	-
0.00251245	3.60E-06	-
0.00392582	3.60E-06	-
0.00230502	1.90E-07	-
0.011897	4.80E-06	-
0.00226543	8.90E-07	-
0.00762753	4.20E-06	-
0.00392946	4.00E-06	-

0.00362056	8.60E-07	-
0.00238615	1.60E-06	-
0.00228323	1.20E-06	-
0.00218282	1.40E-08	-
0.00227496	3.70E-06	-
0.00294347	4.40E-06	-
0.019135	3.10E-09	-
0.00239801	2.40E-06	-
0.00424579	2.40E-06	-
0.00302016	9.90E-08	-
0.00276012	2.60E-06	-
0.00217008	1.50E-07	-
0.00241312	2.10E-08	-
0.00572778	4.20E-08	-
0.00238522	4.40E-06	-
0.00948467	8.10E-07	-
0.0065901	3.70E-07	-
0.00692319	4.60E-06	-
0.00386726	1.30E-06	-
0.00278984	2.80E-06	-
0.0029999	3.10E-06	-
0.00215925	4.90E-06	-
0.00216741	2.00E-06	-
0.00210992	2.70E-06	-
0.012279	3.00E-06	-
0.00774971	4.40E-06	-
0.00853021	2.00E-06	-
0.00230992	3.10E-06	-
0.00608032	1.90E-06	-
0.00211276	4.60E-06	-
0.00221752	4.80E-06	-
0.00222595	2.60E-06	-
0.00586509	4.60E-06	-
0.00212578	3.30E-06	-
0.00232023	9.20E-07	-
0.00304795	7.70E-07	-
0.00204522	2.00E-06	-
0.00208179	7.60E-07	-
0.00210846	3.00E-06	-
0.00395544	4.80E-06	-
0.00273431	4.10E-08	-
0.00283547	3.20E-08	-

0.00263194	2.00E-06	-
0.00834142	3.60E-06	-
0.00238685	4.00E-06	-
0.00213843	4.10E-06	-
0.0176302	8.50E-07	-
0.00284617	3.30E-06	-
0.00206171	3.40E-06	-
0.00249759	2.00E-08	-
0.00243611	7.00E-16	-
0.00212158	4.70E-06	-
0.00257421	4.00E-07	-
0.00239699	1.80E-06	-
0.00307844	5.10E-07	-
0.00211393	9.60E-07	-
0.00794035	1.30E-06	-
0.00901484	1.90E-06	-
0.0038227	9.80E-07	-
0.00223922	4.20E-06	-
0.00219189	4.30E-06	-
0.00304505	4.40E-06	-
0.0041442	1.10E-06	-
0.00330989	3.60E-06	-
0.00216058	6.80E-07	-
0.00218214	8.00E-07	-
0.00742849	2.40E-06	-
0.00235154	4.40E-06	-
0.00377883	2.80E-08	-
0.00783716	4.60E-06	-
0.00318004	4.20E-07	-
0.00241321	2.30E-06	-
0.00210349	4.00E-06	-
0.00428631	1.90E-06	-
0.00237872	1.80E-06	-
0.00285444	7.70E-10	-
0.00639175	2.50E-06	-
0.00219366	7.00E-08	-
0.00618418	1.10E-06	-
0.00241176	2.40E-06	-
0.00275083	2.60E-06	-
0.00215778	4.30E-06	-
0.00438464	2.10E-06	-
0.00243539	7.90E-07	-

0.00207826	3.10E-07	-
0.00279712	1.50E-06	-
0.00206188	4.40E-06	-
0.00243998	4.50E-07	-
0.00243102	1.90E-06	-
0.0183509	2.50E-07	-
0.00228306	2.20E-06	-
0.00600813	1.80E-06	-
0.00648004	3.20E-07	-
0.0026301	5.10E-07	-
0.00488467	2.00E-06	-
0.00276029	9.70E-07	-
0.00337812	2.40E-06	-
0.00229517	9.60E-11	-
0.00334203	3.70E-06	-
0.00214122	4.20E-06	-
0.00227307	1.90E-07	-
0.00585868	3.60E-06	-
0.00627021	1.60E-06	-
0.00207188	8.20E-07	-
0.00626977	3.70E-09	-
0.0036924	1.70E-06	-
0.0051799	4.70E-06	-
0.00212721	1.40E-07	-
0.00406633	2.00E-06	-
0.00249815	1.80E-07	-
0.0108609	9.10E-07	-
0.0100662	1.80E-06	-
0.0027732	4.40E-06	-
0.00243151	2.10E-07	-
0.00226473	4.00E-06	-
0.00262039	1.20E-06	-
0.00259111	6.20E-17	-
0.00239094	3.20E-06	-
0.0026994	2.30E-07	-
0.00221931	3.10E-08	-
0.00214859	8.50E-09	-
0.00438434	7.60E-07	-
0.00300906	3.70E-06	-
0.00942022	4.00E-06	-
0.00804635	4.00E-06	-
0.00235647	1.30E-06	-

0.0028777	3.40E-07	-
0.00230502	3.20E-06	-
0.00534513	3.90E-06	-
0.0028931	3.40E-06	-
0.00251245	3.60E-06	-
0.00392582	3.60E-06	-
0.00230502	1.90E-07	-
0.011897	4.80E-06	-
0.00226543	8.90E-07	-
0.00762753	4.20E-06	-
0.00392946	4.00E-06	-
0.00362056	8.60E-07	-
0.00238615	1.60E-06	-
0.00228323	1.20E-06	-
0.00218282	1.40E-08	-
0.00227496	3.70E-06	-
0.00294347	4.40E-06	-
0.019135	3.10E-09	-
0.00239801	2.40E-06	-
0.00424579	2.40E-06	-
0.00302016	9.90E-08	-
0.00276012	2.60E-06	-
0.00217008	1.50E-07	-
0.00241312	2.10E-08	-
0.00572778	4.20E-08	-
0.00238522	4.40E-06	-
0.00948467	8.10E-07	-
0.0065901	3.70E-07	-
0.00692319	4.60E-06	-
0.00386726	1.30E-06	-
0.00278984	2.80E-06	-
0.0029999	3.10E-06	-
0.00215925	4.90E-06	-
0.00216741	2.00E-06	-
0.00340904	1.90E-06	-
0.00409857	1.90E-06	-
0.00447256	1.20E-06	-
0.00836887	3.50E-06	-
0.008321	4.40E-07	-
0.00738282	1.60E-07	-
0.00261169	4.00E-07	-
0.00253557	3.10E-06	-

0.0043472	3.80E-06	-
0.0117759	7.70E-07	-
0.00611452	2.50E-06	-
0.00629425	4.10E-06	-
0.00262368	4.30E-06	-
0.0100244	4.30E-06	-
0.00578149	2.20E-06	-
0.00766936	3.60E-06	-
0.0022683	2.20E-07	-
0.00235748	1.20E-07	-
0.00554552	3.10E-06	-
0.00228303	4.10E-06	-
0.00276984	3.90E-06	-
0.00876048	1.90E-06	-
0.0105999	3.10E-06	-
0.00220019	1.10E-06	-
0.00646144	5.60E-07	-
0.00823522	3.40E-07	-
0.00350209	2.50E-06	-
0.00210992	2.70E-06	-
0.012279	3.00E-06	-
0.00774971	4.40E-06	-
0.00853021	2.00E-06	-
0.00230992	3.10E-06	-
0.00608032	1.90E-06	-
0.00211276	4.60E-06	-
0.00221752	4.80E-06	-
0.00222595	2.60E-06	-
0.00586509	4.60E-06	-
0.00212578	3.30E-06	-
0.00232023	9.20E-07	-
0.00406633	2.00E-06	-
0.00249815	1.80E-07	-
0.0027732	4.40E-06	-
0.00243151	2.10E-07	-
0.00226473	4.00E-06	-
0.00262039	1.20E-06	-
0.00259111	6.20E-17	-
0.00239094	3.20E-06	-
0.0026994	2.30E-07	-
0.00221931	3.10E-08	-
0.00214859	8.50E-09	-

0.00438434	7.60E-07	-
0.00300906	3.70E-06	-
0.00942022	4.00E-06	-
0.00804635	4.00E-06	-
0.00235647	1.30E-06	-
0.0028777	3.40E-07	-
0.00230502	3.20E-06	-
0.00534513	3.90E-06	-
0.0028931	3.40E-06	-
0.00251245	3.60E-06	-
0.00392582	3.60E-06	-
0.00230502	1.90E-07	-
0.00226543	8.90E-07	-
0.00762753	4.20E-06	-
0.00392946	4.00E-06	-
0.00362056	8.60E-07	-
0.00238615	1.60E-06	-
0.00228323	1.20E-06	-
0.00218282	1.40E-08	-
0.00227496	3.70E-06	-
0.00294347	4.40E-06	-
0.019135	3.10E-09	-
0.00239801	2.40E-06	-
0.00424579	2.40E-06	-
0.00302016	9.90E-08	-
0.00276012	2.60E-06	-
0.00217008	1.50E-07	-
0.00241312	2.10E-08	-
0.00572778	4.20E-08	-
0.00238522	4.40E-06	-
0.0065901	3.70E-07	-
0.00386726	1.30E-06	-
0.00278984	2.80E-06	-
0.0029999	3.10E-06	-
0.00215925	4.90E-06	-
0.00216741	2.00E-06	-
0.00304795	7.70E-07	-
0.00204522	2.00E-06	-
0.00208179	7.60E-07	-
0.00210846	3.00E-06	-
0.00395544	4.80E-06	-
0.00273431	4.10E-08	-

0.00283547	3.20E-08	-
0.00263194	2.00E-06	-
0.00834142	3.60E-06	-
0.00238685	4.00E-06	-
0.00213843	4.10E-06	-
0.00284617	3.30E-06	-
0.00206171	3.40E-06	-
0.00249759	2.00E-08	-
0.00243611	7.00E-16	-
0.00212158	4.70E-06	-
0.00257421	4.00E-07	-
0.00239699	1.80E-06	-
0.00307844	5.10E-07	-
0.00211393	9.60E-07	-
0.00794035	1.30E-06	-
0.00901484	1.90E-06	-
0.0038227	9.80E-07	-
0.00223922	4.20E-06	-
0.00219189	4.30E-06	-
0.00304505	4.40E-06	-
0.0041442	1.10E-06	-
0.00330989	3.60E-06	-
0.00216058	6.80E-07	-
0.00218214	8.00E-07	-
0.00742849	2.40E-06	-
0.00235154	4.40E-06	-
0.00377883	2.80E-08	-
0.00783716	4.60E-06	-
0.00318004	4.20E-07	-
0.00241321	2.30E-06	-
0.00210349	4.00E-06	-
0.00428631	1.90E-06	-
0.00237872	1.80E-06	-
0.00285444	7.70E-10	-
0.00639175	2.50E-06	-
0.00219366	7.00E-08	-
0.00618418	1.10E-06	-
0.00241176	2.40E-06	-
0.00275083	2.60E-06	-
0.00215778	4.30E-06	-
0.00438464	2.10E-06	-
0.00243539	7.90E-07	-

0.00207826	3.10E-07	-
0.00279712	1.50E-06	-
0.00206188	4.40E-06	-
0.00243998	4.50E-07	-
0.00243102	1.90E-06	-
0.0183509	2.50E-07	-
0.00228306	2.20E-06	-
0.00600813	1.80E-06	-
0.00648004	3.20E-07	-
0.0026301	5.10E-07	-
0.00488467	2.00E-06	-
0.00276029	9.70E-07	-
0.00337812	2.40E-06	-
0.00229517	9.60E-11	-
0.00334203	3.70E-06	-
0.00214122	4.20E-06	-
0.00227307	1.90E-07	-
0.00585868	3.60E-06	-
0.00627021	1.60E-06	-
0.00207188	8.20E-07	-
0.00626977	3.70E-09	-
0.0036924	1.70E-06	-
0.0051799	4.70E-06	-
0.00212721	1.40E-07	-
0.00210992	2.70E-06	-
0.00774971	4.40E-06	-
0.00853021	2.00E-06	rs9657892
0.00230992	3.10E-06	-
0.00608032	1.90E-06	-
0.00211276	4.60E-06	-
0.00221752	4.80E-06	-
0.00222595	2.60E-06	-
0.00586509	4.60E-06	-
0.00212578	3.30E-06	-
0.00232023	9.20E-07	-
0.00340904	1.90E-06	-
0.00409857	1.90E-06	-
0.00447256	1.20E-06	-
0.00836887	3.50E-06	-
0.008321	4.40E-07	-
0.00738282	1.60E-07	-
0.00261169	4.00E-07	-

0.00253557	3.10E-06	-
0.0043472	3.80E-06	-
0.00611452	2.50E-06	-
0.00629425	4.10E-06	-
0.00262368	4.30E-06	-
0.0100244	4.30E-06	-
0.00578149	2.20E-06	-
0.00766936	3.60E-06	-
0.0022683	2.20E-07	-
0.00235748	1.20E-07	-
0.00554552	3.10E-06	-
0.00228303	4.10E-06	-
0.00276984	3.90E-06	-
0.00876048	1.90E-06	-
0.00220019	1.10E-06	-
0.00646144	5.60E-07	-
0.00823522	3.40E-07	-
0.00350209	2.50E-06	-
0.00340904	1.90E-06	-
0.00409857	1.90E-06	-
0.00447256	1.20E-06	-
0.00836887	3.50E-06	-
0.008321	4.40E-07	-
0.00738282	1.60E-07	-
0.00261169	4.00E-07	-
0.00253557	3.10E-06	-
0.0043472	3.80E-06	-
0.0117759	7.70E-07	-
0.00611452	2.50E-06	-
0.00629425	4.10E-06	-
0.00262368	4.30E-06	-
0.0100244	4.30E-06	-
0.00578149	2.20E-06	-
0.00766936	3.60E-06	-
0.0022683	2.20E-07	-
0.00235748	1.20E-07	-
0.00554552	3.10E-06	-
0.00228303	4.10E-06	-
0.00276984	3.90E-06	-
0.00876048	1.90E-06	-
0.0105999	3.10E-06	-
0.00220019	1.10E-06	-

0.00646144	5.60E-07	-
0.00823522	3.40E-07	-
0.00350209	2.50E-06	-
0.00304795	7.70E-07	-
0.00204522	2.00E-06	-
0.00208179	7.60E-07	-
0.00210846	3.00E-06	-
0.00395544	4.80E-06	-
0.00273431	4.10E-08	-
0.00283547	3.20E-08	-
0.00263194	2.00E-06	-
0.00834142	3.60E-06	-
0.00238685	4.00E-06	-
0.00213843	4.10E-06	-
0.0176302	8.50E-07	-
0.00284617	3.30E-06	-
0.00206171	3.40E-06	-
0.00249759	2.00E-08	-
0.00243611	7.00E-16	-
0.00212158	4.70E-06	-
0.00257421	4.00E-07	-
0.00239699	1.80E-06	-
0.00307844	5.10E-07	-
0.00211393	9.60E-07	-
0.00794035	1.30E-06	-
0.00901484	1.90E-06	-
0.0038227	9.80E-07	-
0.00223922	4.20E-06	-
0.00219189	4.30E-06	-
0.00304505	4.40E-06	-
0.0041442	1.10E-06	-
0.00330989	3.60E-06	-
0.00216058	6.80E-07	-
0.00218214	8.00E-07	-
0.00742849	2.40E-06	-
0.00235154	4.40E-06	-
0.00377883	2.80E-08	-
0.00783716	4.60E-06	-
0.00318004	4.20E-07	-
0.00241321	2.30E-06	-
0.00210349	4.00E-06	-
0.00428631	1.90E-06	-

0.00237872	1.80E-06	-
0.00285444	7.70E-10	-
0.00639175	2.50E-06	-
0.00219366	7.00E-08	-
0.00618418	1.10E-06	-
0.00241176	2.40E-06	-
0.00275083	2.60E-06	rs17730540
0.00215778	4.30E-06	-
0.00438464	2.10E-06	-
0.00207826	3.10E-07	-
0.00279712	1.50E-06	-
0.00206188	4.40E-06	-
0.00243998	4.50E-07	-
0.00243102	1.90E-06	-
0.0183509	2.50E-07	-
0.00228306	2.20E-06	-
0.00600813	1.80E-06	-
0.00648004	3.20E-07	-
0.0026301	5.10E-07	-
0.00488467	2.00E-06	-
0.00276029	9.70E-07	-
0.00337812	2.40E-06	-
0.00229517	9.60E-11	-
0.00334203	3.70E-06	-
0.00214122	4.20E-06	rs16877501
0.00227307	1.90E-07	-
0.00585868	3.60E-06	-
0.00627021	1.60E-06	-
0.00207188	8.20E-07	-
0.0036924	1.70E-06	-
0.0051799	4.70E-06	-
0.00212721	1.40E-07	-
0.00406633	2.00E-06	-
0.0108609	9.10E-07	-
0.0100662	1.80E-06	-
0.0027732	4.40E-06	-
0.00243151	2.10E-07	rs11855853
0.00226473	4.00E-06	-
0.00262039	1.20E-06	-
0.00259111	6.20E-17	-
0.0026994	2.30E-07	-
0.00221931	3.10E-08	-

0.00214859	8.50E-09	-
0.00438434	7.60E-07	-
0.00300906	3.70E-06	-
0.00942022	4.00E-06	-
0.00804635	4.00E-06	-
0.00235647	1.30E-06	-
0.0028777	3.40E-07	-
0.00230502	3.20E-06	-
0.00534513	3.90E-06	-
0.0028931	3.40E-06	-
0.00251245	3.60E-06	-
0.00392582	3.60E-06	-
0.00230502	1.90E-07	-
0.011897	4.80E-06	-
0.00226543	8.90E-07	-
0.00762753	4.20E-06	-
0.00392946	4.00E-06	-
0.00362056	8.60E-07	rs62484681
0.00238615	1.60E-06	-
0.00228323	1.20E-06	-
0.00218282	1.40E-08	-
0.00227496	3.70E-06	-
0.00294347	4.40E-06	-
0.019135	3.10E-09	-
0.00239801	2.40E-06	-
0.00424579	2.40E-06	-
0.00302016	9.90E-08	-
0.00276012	2.60E-06	-
0.00217008	1.50E-07	-
0.00241312	2.10E-08	-
0.00572778	4.20E-08	-
0.00238522	4.40E-06	-
0.00948467	8.10E-07	-
0.0065901	3.70E-07	-
0.00386726	1.30E-06	-
0.00278984	2.80E-06	-
0.0029999	3.10E-06	-
0.00215925	4.90E-06	-
0.00216741	2.00E-06	-
0.00210992	2.70E-06	-
0.012279	3.00E-06	-
0.00774971	4.40E-06	-

0.00853021	2.00E-06	-
0.00230992	3.10E-06	-
0.00608032	1.90E-06	-
0.00211276	4.60E-06	-
0.00221752	4.80E-06	-
0.00222595	2.60E-06	-
0.00586509	4.60E-06	-
0.00212578	3.30E-06	-
0.00232023	9.20E-07	-
0.00304795	7.70E-07	-
0.00204522	2.00E-06	-
0.00208179	7.60E-07	-
0.00210846	3.00E-06	-
0.00395544	4.80E-06	-
0.00273431	4.10E-08	-
0.00283547	3.20E-08	-
0.00263194	2.00E-06	-
0.00834142	3.60E-06	-
0.00238685	4.00E-06	-
0.00213843	4.10E-06	-
0.0176302	8.50E-07	-
0.00284617	3.30E-06	-
0.00206171	3.40E-06	-
0.00249759	2.00E-08	-
0.00243611	7.00E-16	-
0.00212158	4.70E-06	-
0.00257421	4.00E-07	-
0.00239699	1.80E-06	-
0.00307844	5.10E-07	-
0.00211393	9.60E-07	-
0.00794035	1.30E-06	-
0.00901484	1.90E-06	-
0.0038227	9.80E-07	-
0.00223922	4.20E-06	-
0.00219189	4.30E-06	-
0.00304505	4.40E-06	-
0.0041442	1.10E-06	-
0.00330989	3.60E-06	-
0.00216058	6.80E-07	-
0.00218214	8.00E-07	-
0.00742849	2.40E-06	-
0.00235154	4.40E-06	-

0.00377883	2.80E-08	-
0.00783716	4.60E-06	-
0.00318004	4.20E-07	-
0.00241321	2.30E-06	-
0.00210349	4.00E-06	-
0.00428631	1.90E-06	-
0.00237872	1.80E-06	-
0.00285444	7.70E-10	-
0.00639175	2.50E-06	-
0.00219366	7.00E-08	-
0.00618418	1.10E-06	-
0.00241176	2.40E-06	-
0.00275083	2.60E-06	rs17730540
0.00215778	4.30E-06	-
0.00438464	2.10E-06	-
0.00207826	3.10E-07	-
0.00279712	1.50E-06	-
0.00206188	4.40E-06	-
0.00243998	4.50E-07	-
0.00243102	1.90E-06	-
0.0183509	2.50E-07	-
0.00228306	2.20E-06	-
0.00600813	1.80E-06	-
0.00648004	3.20E-07	-
0.0026301	5.10E-07	-
0.00488467	2.00E-06	-
0.00276029	9.70E-07	-
0.00337812	2.40E-06	-
0.00229517	9.60E-11	-
0.00334203	3.70E-06	-
0.00214122	4.20E-06	rs16877501
0.00227307	1.90E-07	-
0.00585868	3.60E-06	-
0.00627021	1.60E-06	-
0.00207188	8.20E-07	-
0.0036924	1.70E-06	-
0.0051799	4.70E-06	-
0.00212721	1.40E-07	-
0.00340904	1.90E-06	-
0.00409857	1.90E-06	-
0.00447256	1.20E-06	-
0.00836887	3.50E-06	-

0.008321	4.40E-07	-
0.00738282	1.60E-07	-
0.00261169	4.00E-07	-
0.00253557	3.10E-06	-
0.0043472	3.80E-06	-
0.0117759	7.70E-07	-
0.00611452	2.50E-06	-
0.00629425	4.10E-06	-
0.00262368	4.30E-06	-
0.0100244	4.30E-06	-
0.00578149	2.20E-06	-
0.00766936	3.60E-06	-
0.0022683	2.20E-07	-
0.00235748	1.20E-07	-
0.00554552	3.10E-06	-
0.00228303	4.10E-06	-
0.00276984	3.90E-06	-
0.00876048	1.90E-06	-
0.0105999	3.10E-06	-
0.00220019	1.10E-06	-
0.00646144	5.60E-07	-
0.00823522	3.40E-07	-
0.00350209	2.50E-06	-
0.00406633	2.00E-06	-
0.0108609	9.10E-07	-
0.0100662	1.80E-06	-
0.0027732	4.40E-06	-
0.00243151	2.10E-07	rs11855853
0.00226473	4.00E-06	-
0.00262039	1.20E-06	-
0.00259111	6.20E-17	-
0.0026994	2.30E-07	-
0.00221931	3.10E-08	-
0.00214859	8.50E-09	-
0.00438434	7.60E-07	-
0.00300906	3.70E-06	-
0.00942022	4.00E-06	-
0.00804635	4.00E-06	-
0.00235647	1.30E-06	-
0.0028777	3.40E-07	-
0.00230502	3.20E-06	-
0.00534513	3.90E-06	-

0.0028931	3.40E-06	-
0.00251245	3.60E-06	-
0.00392582	3.60E-06	-
0.00230502	1.90E-07	-
0.011897	4.80E-06	-
0.00226543	8.90E-07	-
0.00762753	4.20E-06	-
0.00392946	4.00E-06	-
0.00362056	8.60E-07	rs62484681
0.00238615	1.60E-06	-
0.00228323	1.20E-06	-
0.00218282	1.40E-08	-
0.00227496	3.70E-06	-
0.00294347	4.40E-06	-
0.019135	3.10E-09	-
0.00239801	2.40E-06	-
0.00424579	2.40E-06	-
0.00302016	9.90E-08	-
0.00276012	2.60E-06	-
0.00217008	1.50E-07	-
0.00241312	2.10E-08	-
0.00572778	4.20E-08	-
0.00238522	4.40E-06	-
0.00948467	8.10E-07	-
0.0065901	3.70E-07	-
0.00386726	1.30E-06	-
0.00278984	2.80E-06	-
0.0029999	3.10E-06	-
0.00215925	4.90E-06	-
0.00216741	2.00E-06	-
0.00210992	2.70E-06	-
0.012279	3.00E-06	-
0.00774971	4.40E-06	-
0.00853021	2.00E-06	-
0.00230992	3.10E-06	-
0.00608032	1.90E-06	-
0.00211276	4.60E-06	-
0.00221752	4.80E-06	-
0.00222595	2.60E-06	-
0.00586509	4.60E-06	-
0.00212578	3.30E-06	-
0.00232023	9.20E-07	-

0.00304795	7.70E-07	-
0.00204522	2.00E-06	-
0.00208179	7.60E-07	-
0.00210846	3.00E-06	-
0.00395544	4.80E-06	-
0.00273431	4.10E-08	-
0.00283547	3.20E-08	-
0.00263194	2.00E-06	-
0.00834142	3.60E-06	-
0.00238685	4.00E-06	-
0.00213843	4.10E-06	-
0.0176302	8.50E-07	-
0.00284617	3.30E-06	-
0.00206171	3.40E-06	-
0.00249759	2.00E-08	-
0.00243611	7.00E-16	-
0.00212158	4.70E-06	-
0.00257421	4.00E-07	-
0.00239699	1.80E-06	-
0.00307844	5.10E-07	-
0.00211393	9.60E-07	-
0.00794035	1.30E-06	-
0.00901484	1.90E-06	-
0.0038227	9.80E-07	-
0.00223922	4.20E-06	-
0.00219189	4.30E-06	-
0.00304505	4.40E-06	-
0.0041442	1.10E-06	-
0.00330989	3.60E-06	-
0.00216058	6.80E-07	-
0.00218214	8.00E-07	-
0.00742849	2.40E-06	-
0.00235154	4.40E-06	-
0.00377883	2.80E-08	-
0.00783716	4.60E-06	-
0.00318004	4.20E-07	-
0.00241321	2.30E-06	-
0.00210349	4.00E-06	-
0.00428631	1.90E-06	-
0.00237872	1.80E-06	-
0.00285444	7.70E-10	-
0.00639175	2.50E-06	-

0.00219366	7.00E-08	-
0.00618418	1.10E-06	-
0.00241176	2.40E-06	-
0.00275083	2.60E-06	rs17730540
0.00215778	4.30E-06	-
0.00438464	2.10E-06	-
0.00207826	3.10E-07	-
0.00279712	1.50E-06	-
0.00206188	4.40E-06	-
0.00243998	4.50E-07	-
0.00243102	1.90E-06	-
0.0183509	2.50E-07	-
0.00228306	2.20E-06	-
0.00600813	1.80E-06	-
0.00648004	3.20E-07	-
0.0026301	5.10E-07	-
0.00488467	2.00E-06	-
0.00276029	9.70E-07	-
0.00337812	2.40E-06	-
0.00229517	9.60E-11	-
0.00334203	3.70E-06	-
0.00214122	4.20E-06	rs16877501
0.00227307	1.90E-07	-
0.00585868	3.60E-06	-
0.00627021	1.60E-06	-
0.00207188	8.20E-07	-
0.0036924	1.70E-06	-
0.0051799	4.70E-06	-
0.00212721	1.40E-07	-
0.00406633	2.00E-06	-
0.0108609	9.10E-07	-
0.0100662	1.80E-06	-
0.0027732	4.40E-06	-
0.00243151	2.10E-07	rs11855853
0.00226473	4.00E-06	-
0.00262039	1.20E-06	-
0.00259111	6.20E-17	-
0.0026994	2.30E-07	-
0.00221931	3.10E-08	-
0.00214859	8.50E-09	-
0.00438434	7.60E-07	-
0.00300906	3.70E-06	-

0.00942022	4.00E-06	-
0.00804635	4.00E-06	-
0.00235647	1.30E-06	-
0.0028777	3.40E-07	-
0.00230502	3.20E-06	-
0.00534513	3.90E-06	-
0.0028931	3.40E-06	-
0.00251245	3.60E-06	-
0.00392582	3.60E-06	-
0.00230502	1.90E-07	-
0.011897	4.80E-06	-
0.00226543	8.90E-07	-
0.00762753	4.20E-06	-
0.00392946	4.00E-06	-
0.00362056	8.60E-07	rs62484681
0.00238615	1.60E-06	-
0.00228323	1.20E-06	-
0.00218282	1.40E-08	-
0.00227496	3.70E-06	-
0.00294347	4.40E-06	-
0.019135	3.10E-09	-
0.00239801	2.40E-06	-
0.00424579	2.40E-06	-
0.00302016	9.90E-08	-
0.00276012	2.60E-06	-
0.00217008	1.50E-07	-
0.00241312	2.10E-08	-
0.00572778	4.20E-08	-
0.00238522	4.40E-06	-
0.00948467	8.10E-07	-
0.0065901	3.70E-07	-
0.00386726	1.30E-06	-
0.00278984	2.80E-06	-
0.0029999	3.10E-06	-
0.00215925	4.90E-06	-
0.00216741	2.00E-06	-
0.00340904	1.90E-06	-
0.00409857	1.90E-06	-
0.00447256	1.20E-06	-
0.00836887	3.50E-06	-
0.008321	4.40E-07	-
0.00738282	1.60E-07	-

0.00261169	4.00E-07	-
0.00253557	3.10E-06	-
0.0043472	3.80E-06	-
0.0117759	7.70E-07	-
0.00611452	2.50E-06	-
0.00629425	4.10E-06	-
0.00262368	4.30E-06	-
0.0100244	4.30E-06	-
0.00578149	2.20E-06	-
0.00766936	3.60E-06	-
0.0022683	2.20E-07	-
0.00235748	1.20E-07	-
0.00554552	3.10E-06	-
0.00228303	4.10E-06	-
0.00276984	3.90E-06	-
0.00876048	1.90E-06	-
0.0105999	3.10E-06	-
0.00220019	1.10E-06	-
0.00646144	5.60E-07	-
0.00823522	3.40E-07	-
0.00350209	2.50E-06	-
0.00210992	2.70E-06	-
0.012279	3.00E-06	-
0.00774971	4.40E-06	-
0.00853021	2.00E-06	-
0.00230992	3.10E-06	-
0.00608032	1.90E-06	-
0.00211276	4.60E-06	-
0.00221752	4.80E-06	-
0.00222595	2.60E-06	-
0.00586509	4.60E-06	-
0.00212578	3.30E-06	-
0.00232023	9.20E-07	-
0.00304795	7.70E-07	-
0.00204522	2.00E-06	-
0.00208179	7.60E-07	-
0.00210846	3.00E-06	-
0.00395544	4.80E-06	-
0.00273431	4.10E-08	-
0.00283547	3.20E-08	-
0.00263194	2.00E-06	-
0.00834142	3.60E-06	-

0.00238685	4.00E-06	-
0.00213843	4.10E-06	-
0.0176302	8.50E-07	-
0.00284617	3.30E-06	-
0.00206171	3.40E-06	-
0.00249759	2.00E-08	-
0.00243611	7.00E-16	-
0.00212158	4.70E-06	-
0.00257421	4.00E-07	-
0.00239699	1.80E-06	-
0.00307844	5.10E-07	-
0.00211393	9.60E-07	-
0.00794035	1.30E-06	-
0.00901484	1.90E-06	-
0.0038227	9.80E-07	-
0.00223922	4.20E-06	-
0.00219189	4.30E-06	-
0.00304505	4.40E-06	-
0.0041442	1.10E-06	-
0.00330989	3.60E-06	-
0.00216058	6.80E-07	-
0.00218214	8.00E-07	-
0.00742849	2.40E-06	-
0.00235154	4.40E-06	-
0.00377883	2.80E-08	-
0.00783716	4.60E-06	-
0.00318004	4.20E-07	-
0.00241321	2.30E-06	-
0.00210349	4.00E-06	-
0.00428631	1.90E-06	-
0.00237872	1.80E-06	-
0.00285444	7.70E-10	-
0.00639175	2.50E-06	-
0.00219366	7.00E-08	-
0.00618418	1.10E-06	-
0.00241176	2.40E-06	-
0.00275083	2.60E-06	rs17730540
0.00215778	4.30E-06	-
0.00438464	2.10E-06	-
0.00207826	3.10E-07	-
0.00279712	1.50E-06	-
0.00206188	4.40E-06	-

0.00243998	4.50E-07	-
0.00243102	1.90E-06	-
0.0183509	2.50E-07	-
0.00228306	2.20E-06	-
0.00600813	1.80E-06	-
0.00648004	3.20E-07	-
0.0026301	5.10E-07	-
0.00488467	2.00E-06	-
0.00276029	9.70E-07	-
0.00337812	2.40E-06	-
0.00229517	9.60E-11	-
0.00334203	3.70E-06	-
0.00214122	4.20E-06	rs16877501
0.00227307	1.90E-07	-
0.00585868	3.60E-06	-
0.00627021	1.60E-06	-
0.00207188	8.20E-07	-
0.0036924	1.70E-06	-
0.0051799	4.70E-06	-
0.00212721	1.40E-07	-
0.00406633	2.00E-06	-
0.0108609	9.10E-07	-
0.0100662	1.80E-06	-
0.0027732	4.40E-06	-
0.00243151	2.10E-07	rs11855853
0.00226473	4.00E-06	-
0.00262039	1.20E-06	-
0.00259111	6.20E-17	-
0.0026994	2.30E-07	-
0.00221931	3.10E-08	-
0.00214859	8.50E-09	-
0.00438434	7.60E-07	-
0.00300906	3.70E-06	-
0.00942022	4.00E-06	-
0.00804635	4.00E-06	-
0.00235647	1.30E-06	-
0.0028777	3.40E-07	-
0.00230502	3.20E-06	-
0.00534513	3.90E-06	-
0.0028931	3.40E-06	-
0.00251245	3.60E-06	-
0.00392582	3.60E-06	-

0.00230502	1.90E-07	-
0.011897	4.80E-06	-
0.00226543	8.90E-07	-
0.00762753	4.20E-06	-
0.00392946	4.00E-06	-
0.00362056	8.60E-07	rs62484681
0.00238615	1.60E-06	-
0.00228323	1.20E-06	-
0.00218282	1.40E-08	-
0.00227496	3.70E-06	-
0.00294347	4.40E-06	-
0.019135	3.10E-09	-
0.00239801	2.40E-06	-
0.00424579	2.40E-06	-
0.00302016	9.90E-08	-
0.00276012	2.60E-06	-
0.00217008	1.50E-07	-
0.00241312	2.10E-08	-
0.00572778	4.20E-08	-
0.00238522	4.40E-06	-
0.00948467	8.10E-07	-
0.0065901	3.70E-07	-
0.00386726	1.30E-06	-
0.00278984	2.80E-06	-
0.0029999	3.10E-06	-
0.00215925	4.90E-06	-
0.00216741	2.00E-06	-
0.00340904	1.90E-06	-
0.00409857	1.90E-06	-
0.00447256	1.20E-06	-
0.00836887	3.50E-06	-
0.008321	4.40E-07	-
0.00738282	1.60E-07	-
0.00261169	4.00E-07	-
0.00253557	3.10E-06	-
0.0043472	3.80E-06	-
0.0117759	7.70E-07	-
0.00611452	2.50E-06	-
0.00629425	4.10E-06	-
0.00262368	4.30E-06	-
0.0100244	4.30E-06	-
0.00578149	2.20E-06	-

0.00766936	3.60E-06	-
0.0022683	2.20E-07	-
0.00235748	1.20E-07	-
0.00554552	3.10E-06	-
0.00228303	4.10E-06	-
0.00276984	3.90E-06	-
0.00876048	1.90E-06	-
0.0105999	3.10E-06	-
0.00220019	1.10E-06	-
0.00646144	5.60E-07	-
0.00823522	3.40E-07	-
0.00350209	2.50E-06	-
0.00210992	2.70E-06	-
0.012279	3.00E-06	-
0.00774971	4.40E-06	-
0.00853021	2.00E-06	-
0.00230992	3.10E-06	-
0.00608032	1.90E-06	-
0.00211276	4.60E-06	-
0.00221752	4.80E-06	-
0.00222595	2.60E-06	-
0.00586509	4.60E-06	-
0.00212578	3.30E-06	-
0.00232023	9.20E-07	-
0.00406633	2.00E-06	-
0.0108609	9.10E-07	-
0.0100662	1.80E-06	-
0.0027732	4.40E-06	-
0.00243151	2.10E-07	rs11855853
0.00226473	4.00E-06	-
0.00262039	1.20E-06	-
0.00259111	6.20E-17	-
0.0026994	2.30E-07	-
0.00221931	3.10E-08	-
0.00214859	8.50E-09	-
0.00438434	7.60E-07	-
0.00300906	3.70E-06	-
0.00942022	4.00E-06	-
0.00804635	4.00E-06	-
0.00235647	1.30E-06	-
0.0028777	3.40E-07	-
0.00230502	3.20E-06	-

0.00534513	3.90E-06	-
0.0028931	3.40E-06	-
0.00251245	3.60E-06	-
0.00392582	3.60E-06	-
0.00230502	1.90E-07	-
0.011897	4.80E-06	-
0.00226543	8.90E-07	-
0.00762753	4.20E-06	-
0.00392946	4.00E-06	-
0.00362056	8.60E-07	rs62484681
0.00238615	1.60E-06	-
0.00228323	1.20E-06	-
0.00218282	1.40E-08	-
0.00227496	3.70E-06	-
0.00294347	4.40E-06	-
0.019135	3.10E-09	-
0.00239801	2.40E-06	-
0.00424579	2.40E-06	-
0.00302016	9.90E-08	-
0.00276012	2.60E-06	-
0.00217008	1.50E-07	-
0.00241312	2.10E-08	-
0.00572778	4.20E-08	-
0.00238522	4.40E-06	-
0.00948467	8.10E-07	-
0.0065901	3.70E-07	-
0.00386726	1.30E-06	-
0.00278984	2.80E-06	-
0.0029999	3.10E-06	-
0.00215925	4.90E-06	-
0.00216741	2.00E-06	-
0.00304795	7.70E-07	-
0.00204522	2.00E-06	-
0.00208179	7.60E-07	-
0.00210846	3.00E-06	-
0.00395544	4.80E-06	-
0.00273431	4.10E-08	-
0.00283547	3.20E-08	-
0.00263194	2.00E-06	-
0.00834142	3.60E-06	-
0.00238685	4.00E-06	-
0.00213843	4.10E-06	-

0.0176302	8.50E-07	-
0.00284617	3.30E-06	-
0.00206171	3.40E-06	-
0.00249759	2.00E-08	-
0.00243611	7.00E-16	-
0.00212158	4.70E-06	-
0.00257421	4.00E-07	-
0.00239699	1.80E-06	-
0.00307844	5.10E-07	-
0.00211393	9.60E-07	-
0.00794035	1.30E-06	-
0.00901484	1.90E-06	-
0.0038227	9.80E-07	-
0.00223922	4.20E-06	-
0.00219189	4.30E-06	-
0.00304505	4.40E-06	-
0.0041442	1.10E-06	-
0.00330989	3.60E-06	-
0.00216058	6.80E-07	-
0.00218214	8.00E-07	-
0.00742849	2.40E-06	-
0.00235154	4.40E-06	-
0.00377883	2.80E-08	-
0.00783716	4.60E-06	-
0.00318004	4.20E-07	-
0.00241321	2.30E-06	-
0.00210349	4.00E-06	-
0.00428631	1.90E-06	-
0.00237872	1.80E-06	-
0.00285444	7.70E-10	-
0.00639175	2.50E-06	-
0.00219366	7.00E-08	-
0.00618418	1.10E-06	-
0.00241176	2.40E-06	-
0.00275083	2.60E-06	rs17730540
0.00215778	4.30E-06	-
0.00438464	2.10E-06	-
0.00207826	3.10E-07	-
0.00279712	1.50E-06	-
0.00206188	4.40E-06	-
0.00243998	4.50E-07	-
0.00243102	1.90E-06	-

0.0183509	2.50E-07	-
0.00228306	2.20E-06	-
0.00600813	1.80E-06	-
0.00648004	3.20E-07	-
0.0026301	5.10E-07	-
0.00488467	2.00E-06	-
0.00276029	9.70E-07	-
0.00337812	2.40E-06	-
0.00229517	9.60E-11	-
0.00334203	3.70E-06	-
0.00214122	4.20E-06	rs16877501
0.00227307	1.90E-07	-
0.00585868	3.60E-06	-
0.00627021	1.60E-06	-
0.00207188	8.20E-07	-
0.0036924	1.70E-06	-
0.0051799	4.70E-06	-
0.00212721	1.40E-07	-
0.00340904	1.90E-06	-
0.00409857	1.90E-06	-
0.00447256	1.20E-06	-
0.00836887	3.50E-06	-
0.008321	4.40E-07	-
0.00738282	1.60E-07	-
0.00261169	4.00E-07	-
0.00253557	3.10E-06	-
0.0043472	3.80E-06	-
0.0117759	7.70E-07	-
0.00611452	2.50E-06	-
0.00629425	4.10E-06	-
0.00262368	4.30E-06	-
0.0100244	4.30E-06	-
0.00578149	2.20E-06	-
0.00766936	3.60E-06	-
0.0022683	2.20E-07	-
0.00235748	1.20E-07	-
0.00554552	3.10E-06	-
0.00228303	4.10E-06	-
0.00276984	3.90E-06	-
0.00876048	1.90E-06	-
0.0105999	3.10E-06	-
0.00220019	1.10E-06	-

0.00646144	5.60E-07	-
0.00823522	3.40E-07	-
0.00350209	2.50E-06	-
0.00210992	2.70E-06	-
0.012279	3.00E-06	-
0.00774971	4.40E-06	-
0.00853021	2.00E-06	-
0.00230992	3.10E-06	-
0.00608032	1.90E-06	-
0.00211276	4.60E-06	-
0.00221752	4.80E-06	-
0.00222595	2.60E-06	-
0.00586509	4.60E-06	-
0.00212578	3.30E-06	-
0.00232023	9.20E-07	-
0.00406633	2.00E-06	-
0.0108609	9.10E-07	-
0.0100662	1.80E-06	-
0.0027732	4.40E-06	-
0.00243151	2.10E-07	rs11855853
0.00226473	4.00E-06	-
0.00262039	1.20E-06	-
0.00259111	6.20E-17	-
0.0026994	2.30E-07	-
0.00221931	3.10E-08	-
0.00214859	8.50E-09	-
0.00438434	7.60E-07	-
0.00300906	3.70E-06	-
0.00942022	4.00E-06	-
0.00804635	4.00E-06	-
0.00235647	1.30E-06	-
0.0028777	3.40E-07	-
0.00230502	3.20E-06	-
0.00534513	3.90E-06	-
0.0028931	3.40E-06	-
0.00251245	3.60E-06	-
0.00392582	3.60E-06	-
0.00230502	1.90E-07	-
0.011897	4.80E-06	-
0.00226543	8.90E-07	-
0.00762753	4.20E-06	-
0.00392946	4.00E-06	-

0.00362056	8.60E-07	rs62484681
0.00238615	1.60E-06	-
0.00228323	1.20E-06	-
0.00218282	1.40E-08	-
0.00227496	3.70E-06	-
0.00294347	4.40E-06	-
0.019135	3.10E-09	-
0.00239801	2.40E-06	-
0.00424579	2.40E-06	-
0.00302016	9.90E-08	-
0.00276012	2.60E-06	-
0.00217008	1.50E-07	-
0.00241312	2.10E-08	-
0.00572778	4.20E-08	-
0.00238522	4.40E-06	-
0.00948467	8.10E-07	-
0.0065901	3.70E-07	-
0.00386726	1.30E-06	-
0.00278984	2.80E-06	-
0.0029999	3.10E-06	-
0.00215925	4.90E-06	-
0.00216741	2.00E-06	-
0.00304795	7.70E-07	-
0.00204522	2.00E-06	-
0.00208179	7.60E-07	-
0.00210846	3.00E-06	-
0.00395544	4.80E-06	-
0.00273431	4.10E-08	-
0.00283547	3.20E-08	-
0.00263194	2.00E-06	-
0.00834142	3.60E-06	-
0.00238685	4.00E-06	-
0.00213843	4.10E-06	-
0.0176302	8.50E-07	-
0.00284617	3.30E-06	-
0.00206171	3.40E-06	-
0.00249759	2.00E-08	-
0.00243611	7.00E-16	-
0.00212158	4.70E-06	-
0.00257421	4.00E-07	-
0.00239699	1.80E-06	-
0.00307844	5.10E-07	-

0.00211393	9.60E-07	-
0.00794035	1.30E-06	-
0.00901484	1.90E-06	-
0.0038227	9.80E-07	-
0.00223922	4.20E-06	-
0.00219189	4.30E-06	-
0.00304505	4.40E-06	-
0.0041442	1.10E-06	-
0.00330989	3.60E-06	-
0.00216058	6.80E-07	-
0.00218214	8.00E-07	-
0.00742849	2.40E-06	-
0.00235154	4.40E-06	-
0.00377883	2.80E-08	-
0.00783716	4.60E-06	-
0.00318004	4.20E-07	-
0.00241321	2.30E-06	-
0.00210349	4.00E-06	-
0.00428631	1.90E-06	-
0.00237872	1.80E-06	-
0.00285444	7.70E-10	-
0.00639175	2.50E-06	-
0.00219366	7.00E-08	-
0.00618418	1.10E-06	-
0.00241176	2.40E-06	-
0.00275083	2.60E-06	rs17730540
0.00215778	4.30E-06	-
0.00438464	2.10E-06	-
0.00207826	3.10E-07	-
0.00279712	1.50E-06	-
0.00206188	4.40E-06	-
0.00243998	4.50E-07	-
0.00243102	1.90E-06	-
0.0183509	2.50E-07	-
0.00228306	2.20E-06	-
0.00600813	1.80E-06	-
0.00648004	3.20E-07	-
0.0026301	5.10E-07	-
0.00488467	2.00E-06	-
0.00276029	9.70E-07	-
0.00337812	2.40E-06	-
0.00229517	9.60E-11	-

0.00334203	3.70E-06	-
0.00214122	4.20E-06	rs16877501
0.00227307	1.90E-07	-
0.00585868	3.60E-06	-
0.00627021	1.60E-06	-
0.00207188	8.20E-07	-
0.0036924	1.70E-06	-
0.0051799	4.70E-06	-
0.00212721	1.40E-07	-
0.00210992	2.70E-06	-
0.012279	3.00E-06	-
0.00774971	4.40E-06	-
0.00853021	2.00E-06	-
0.00230992	3.10E-06	-
0.00608032	1.90E-06	-
0.00211276	4.60E-06	-
0.00221752	4.80E-06	-
0.00222595	2.60E-06	-
0.00586509	4.60E-06	-
0.00212578	3.30E-06	-
0.00232023	9.20E-07	-
0.00340904	1.90E-06	-
0.00409857	1.90E-06	-
0.00447256	1.20E-06	-
0.00836887	3.50E-06	-
0.008321	4.40E-07	-
0.00738282	1.60E-07	-
0.00261169	4.00E-07	-
0.00253557	3.10E-06	-
0.0043472	3.80E-06	-
0.0117759	7.70E-07	-
0.00611452	2.50E-06	-
0.00629425	4.10E-06	-
0.00262368	4.30E-06	-
0.0100244	4.30E-06	-
0.00578149	2.20E-06	-
0.00766936	3.60E-06	-
0.0022683	2.20E-07	-
0.00235748	1.20E-07	-
0.00554552	3.10E-06	-
0.00228303	4.10E-06	-
0.00276984	3.90E-06	-

0.00876048	1.90E-06	-
0.0105999	3.10E-06	-
0.00220019	1.10E-06	-
0.00646144	5.60E-07	-
0.00823522	3.40E-07	-
0.00350209	2.50E-06	-
0.00406633	2.00E-06	-
0.0108609	9.10E-07	-
0.0100662	1.80E-06	-
0.0027732	4.40E-06	-
0.00243151	2.10E-07	rs11855853
0.00226473	4.00E-06	-
0.00262039	1.20E-06	-
0.00259111	6.20E-17	-
0.0026994	2.30E-07	-
0.00221931	3.10E-08	-
0.00214859	8.50E-09	-
0.00438434	7.60E-07	-
0.00300906	3.70E-06	-
0.00942022	4.00E-06	-
0.00804635	4.00E-06	-
0.00235647	1.30E-06	-
0.0028777	3.40E-07	-
0.00230502	3.20E-06	-
0.00534513	3.90E-06	-
0.0028931	3.40E-06	-
0.00251245	3.60E-06	-
0.00392582	3.60E-06	-
0.00230502	1.90E-07	-
0.011897	4.80E-06	-
0.00226543	8.90E-07	-
0.00762753	4.20E-06	-
0.00392946	4.00E-06	-
0.00362056	8.60E-07	rs62484681
0.00238615	1.60E-06	-
0.00228323	1.20E-06	-
0.00218282	1.40E-08	-
0.00227496	3.70E-06	-
0.00294347	4.40E-06	-
0.019135	3.10E-09	-
0.00239801	2.40E-06	-
0.00424579	2.40E-06	-

0.00302016	9.90E-08	-
0.00276012	2.60E-06	-
0.00217008	1.50E-07	-
0.00241312	2.10E-08	-
0.00572778	4.20E-08	-
0.00238522	4.40E-06	-
0.00948467	8.10E-07	-
0.0065901	3.70E-07	-
0.00386726	1.30E-06	-
0.00278984	2.80E-06	-
0.0029999	3.10E-06	-
0.00215925	4.90E-06	-
0.00216741	2.00E-06	-
0.00304795	7.70E-07	-
0.00204522	2.00E-06	-
0.00208179	7.60E-07	-
0.00210846	3.00E-06	-
0.00395544	4.80E-06	-
0.00273431	4.10E-08	-
0.00283547	3.20E-08	-
0.00263194	2.00E-06	-
0.00834142	3.60E-06	-
0.00238685	4.00E-06	-
0.00213843	4.10E-06	-
0.0176302	8.50E-07	-
0.00284617	3.30E-06	-
0.00206171	3.40E-06	-
0.00249759	2.00E-08	-
0.00243611	7.00E-16	-
0.00212158	4.70E-06	-
0.00257421	4.00E-07	-
0.00239699	1.80E-06	-
0.00307844	5.10E-07	-
0.00211393	9.60E-07	-
0.00794035	1.30E-06	-
0.00901484	1.90E-06	-
0.0038227	9.80E-07	-
0.00223922	4.20E-06	-
0.00219189	4.30E-06	-
0.00304505	4.40E-06	-
0.0041442	1.10E-06	-
0.00330989	3.60E-06	-

0.00216058	6.80E-07	-
0.00218214	8.00E-07	-
0.00742849	2.40E-06	-
0.00235154	4.40E-06	-
0.00377883	2.80E-08	-
0.00783716	4.60E-06	-
0.00318004	4.20E-07	-
0.00241321	2.30E-06	-
0.00210349	4.00E-06	-
0.00428631	1.90E-06	-
0.00237872	1.80E-06	-
0.00285444	7.70E-10	-
0.00639175	2.50E-06	-
0.00219366	7.00E-08	-
0.00618418	1.10E-06	-
0.00241176	2.40E-06	-
0.00275083	2.60E-06	rs17730540
0.00215778	4.30E-06	-
0.00438464	2.10E-06	-
0.00207826	3.10E-07	-
0.00279712	1.50E-06	-
0.00206188	4.40E-06	-
0.00243998	4.50E-07	-
0.00243102	1.90E-06	-
0.0183509	2.50E-07	-
0.00228306	2.20E-06	-
0.00600813	1.80E-06	-
0.00648004	3.20E-07	-
0.0026301	5.10E-07	-
0.00488467	2.00E-06	-
0.00276029	9.70E-07	-
0.00337812	2.40E-06	-
0.00229517	9.60E-11	-
0.00334203	3.70E-06	-
0.00214122	4.20E-06	rs16877501
0.00227307	1.90E-07	-
0.00585868	3.60E-06	-
0.00627021	1.60E-06	-
0.00207188	8.20E-07	-
0.0036924	1.70E-06	-
0.0051799	4.70E-06	-
0.00212721	1.40E-07	-

0.00340904	1.90E-06	-
0.00409857	1.90E-06	-
0.00447256	1.20E-06	-
0.00836887	3.50E-06	-
0.008321	4.40E-07	-
0.00738282	1.60E-07	-
0.00261169	4.00E-07	-
0.00253557	3.10E-06	-
0.0043472	3.80E-06	-
0.0117759	7.70E-07	-
0.00611452	2.50E-06	-
0.00629425	4.10E-06	-
0.00262368	4.30E-06	-
0.0100244	4.30E-06	-
0.00578149	2.20E-06	-
0.00766936	3.60E-06	-
0.0022683	2.20E-07	-
0.00235748	1.20E-07	-
0.00554552	3.10E-06	-
0.00228303	4.10E-06	-
0.00276984	3.90E-06	-
0.00876048	1.90E-06	-
0.0105999	3.10E-06	-
0.00220019	1.10E-06	-
0.00646144	5.60E-07	-
0.00823522	3.40E-07	-
0.00350209	2.50E-06	-
0.00210992	2.70E-06	-
0.012279	3.00E-06	-
0.00774971	4.40E-06	-
0.00853021	2.00E-06	-
0.00230992	3.10E-06	-
0.00608032	1.90E-06	-
0.00211276	4.60E-06	-
0.00221752	4.80E-06	-
0.00222595	2.60E-06	-
0.00586509	4.60E-06	-
0.00212578	3.30E-06	-
0.00232023	9.20E-07	-
0.00304795	7.70E-07	-
0.00204522	2.00E-06	-
0.00208179	7.60E-07	-

0.00210846	3.00E-06	-
0.00395544	4.80E-06	-
0.00273431	4.10E-08	-
0.00283547	3.20E-08	-
0.00263194	2.00E-06	-
0.00834142	3.60E-06	-
0.00238685	4.00E-06	-
0.00213843	4.10E-06	-
0.0176302	8.50E-07	-
0.00284617	3.30E-06	-
0.00206171	3.40E-06	-
0.00249759	2.00E-08	-
0.00243611	7.00E-16	-
0.00212158	4.70E-06	-
0.00257421	4.00E-07	-
0.00239699	1.80E-06	-
0.00307844	5.10E-07	-
0.00211393	9.60E-07	-
0.00794035	1.30E-06	-
0.00901484	1.90E-06	-
0.0038227	9.80E-07	-
0.00223922	4.20E-06	-
0.00219189	4.30E-06	-
0.00304505	4.40E-06	-
0.0041442	1.10E-06	-
0.00330989	3.60E-06	-
0.00216058	6.80E-07	-
0.00218214	8.00E-07	-
0.00742849	2.40E-06	-
0.00235154	4.40E-06	-
0.00377883	2.80E-08	-
0.00783716	4.60E-06	-
0.00318004	4.20E-07	-
0.00241321	2.30E-06	-
0.00210349	4.00E-06	-
0.00428631	1.90E-06	-
0.00237872	1.80E-06	-
0.00285444	7.70E-10	-
0.00639175	2.50E-06	-
0.00219366	7.00E-08	-
0.00618418	1.10E-06	-
0.00241176	2.40E-06	-

0.00275083	2.60E-06	rs17730540
0.00215778	4.30E-06	-
0.00438464	2.10E-06	-
0.00207826	3.10E-07	-
0.00279712	1.50E-06	-
0.00206188	4.40E-06	-
0.00243998	4.50E-07	-
0.00243102	1.90E-06	-
0.0183509	2.50E-07	-
0.00228306	2.20E-06	-
0.00600813	1.80E-06	-
0.00648004	3.20E-07	-
0.0026301	5.10E-07	-
0.00488467	2.00E-06	-
0.00276029	9.70E-07	-
0.00337812	2.40E-06	-
0.00229517	9.60E-11	-
0.00334203	3.70E-06	-
0.00214122	4.20E-06	rs16877501
0.00227307	1.90E-07	-
0.00585868	3.60E-06	-
0.00627021	1.60E-06	-
0.00207188	8.20E-07	-
0.0036924	1.70E-06	-
0.0051799	4.70E-06	-
0.00212721	1.40E-07	-
0.00406633	2.00E-06	-
0.0108609	9.10E-07	-
0.0100662	1.80E-06	-
0.0027732	4.40E-06	-
0.00243151	2.10E-07	rs11855853
0.00226473	4.00E-06	-
0.00262039	1.20E-06	-
0.00259111	6.20E-17	-
0.0026994	2.30E-07	-
0.00221931	3.10E-08	-
0.00214859	8.50E-09	-
0.00438434	7.60E-07	-
0.00300906	3.70E-06	-
0.00942022	4.00E-06	-
0.00804635	4.00E-06	-
0.00235647	1.30E-06	-

0.0028777	3.40E-07	-
0.00230502	3.20E-06	-
0.00534513	3.90E-06	-
0.0028931	3.40E-06	-
0.00251245	3.60E-06	-
0.00392582	3.60E-06	-
0.00230502	1.90E-07	-
0.011897	4.80E-06	-
0.00226543	8.90E-07	-
0.00762753	4.20E-06	-
0.00392946	4.00E-06	-
0.00362056	8.60E-07	rs62484681
0.00238615	1.60E-06	-
0.00228323	1.20E-06	-
0.00218282	1.40E-08	-
0.00227496	3.70E-06	-
0.00294347	4.40E-06	-
0.019135	3.10E-09	-
0.00239801	2.40E-06	-
0.00424579	2.40E-06	-
0.00302016	9.90E-08	-
0.00276012	2.60E-06	-
0.00217008	1.50E-07	-
0.00241312	2.10E-08	-
0.00572778	4.20E-08	-
0.00238522	4.40E-06	-
0.00948467	8.10E-07	-
0.0065901	3.70E-07	-
0.00386726	1.30E-06	-
0.00278984	2.80E-06	-
0.0029999	3.10E-06	-
0.00215925	4.90E-06	-
0.00216741	2.00E-06	-
0.00340904	1.90E-06	-
0.00409857	1.90E-06	-
0.00447256	1.20E-06	-
0.00836887	3.50E-06	-
0.008321	4.40E-07	-
0.00738282	1.60E-07	-
0.00261169	4.00E-07	-
0.00253557	3.10E-06	-
0.0043472	3.80E-06	-

0.0117759	7.70E-07	-
0.00611452	2.50E-06	-
0.00629425	4.10E-06	-
0.00262368	4.30E-06	-
0.0100244	4.30E-06	-
0.00578149	2.20E-06	-
0.00766936	3.60E-06	-
0.0022683	2.20E-07	-
0.00235748	1.20E-07	-
0.00554552	3.10E-06	-
0.00228303	4.10E-06	-
0.00276984	3.90E-06	-
0.00876048	1.90E-06	-
0.0105999	3.10E-06	-
0.00220019	1.10E-06	-
0.00646144	5.60E-07	-
0.00823522	3.40E-07	-
0.00350209	2.50E-06	-
0.00210992	2.70E-06	-
0.012279	3.00E-06	-
0.00774971	4.40E-06	-
0.00853021	2.00E-06	-
0.00230992	3.10E-06	-
0.00608032	1.90E-06	-
0.00211276	4.60E-06	-
0.00221752	4.80E-06	-
0.00222595	2.60E-06	-
0.00586509	4.60E-06	-
0.00212578	3.30E-06	-
0.00232023	9.20E-07	-
0.00304795	7.70E-07	-
0.00204522	2.00E-06	-
0.00208179	7.60E-07	-
0.00210846	3.00E-06	-
0.00395544	4.80E-06	-
0.00273431	4.10E-08	-
0.00283547	3.20E-08	-
0.00263194	2.00E-06	-
0.00834142	3.60E-06	-
0.00238685	4.00E-06	-
0.00213843	4.10E-06	-
0.00284617	3.30E-06	-

0.00206171	3.40E-06	-
0.00249759	2.00E-08	-
0.00243611	7.00E-16	-
0.00212158	4.70E-06	-
0.00257421	4.00E-07	-
0.00239699	1.80E-06	-
0.00307844	5.10E-07	-
0.00211393	9.60E-07	-
0.00794035	1.30E-06	-
0.0038227	9.80E-07	-
0.00223922	4.20E-06	-
0.00219189	4.30E-06	-
0.00304505	4.40E-06	-
0.0041442	1.10E-06	-
0.00330989	3.60E-06	-
0.00216058	6.80E-07	-
0.00218214	8.00E-07	-
0.00742849	2.40E-06	-
0.00235154	4.40E-06	-
0.00377883	2.80E-08	-
0.00783716	4.60E-06	-
0.00318004	4.20E-07	-
0.00241321	2.30E-06	-
0.00210349	4.00E-06	-
0.00428631	1.90E-06	-
0.00237872	1.80E-06	-
0.00285444	7.70E-10	-
0.00219366	7.00E-08	-
0.00618418	1.10E-06	-
0.00241176	2.40E-06	-
0.00275083	2.60E-06	-
0.00215778	4.30E-06	-
0.00438464	2.10E-06	-
0.00243539	7.90E-07	-
0.00207826	3.10E-07	-
0.00279712	1.50E-06	-
0.00206188	4.40E-06	-
0.00243998	4.50E-07	-
0.00243102	1.90E-06	-
0.0183509	2.50E-07	-
0.00228306	2.20E-06	-
0.00600813	1.80E-06	-

0.00648004	3.20E-07	-
0.0026301	5.10E-07	-
0.00488467	2.00E-06	-
0.00276029	9.70E-07	-
0.00337812	2.40E-06	-
0.00229517	9.60E-11	-
0.00334203	3.70E-06	-
0.00214122	4.20E-06	-
0.00227307	1.90E-07	-
0.00585868	3.60E-06	-
0.00627021	1.60E-06	-
0.00207188	8.20E-07	-
0.00626977	3.70E-09	-
0.0036924	1.70E-06	-
0.0051799	4.70E-06	-
0.00212721	1.40E-07	-
0.00340904	1.90E-06	-
0.00409857	1.90E-06	-
0.00447256	1.20E-06	-
0.00836887	3.50E-06	-
0.008321	4.40E-07	-
0.00738282	1.60E-07	-
0.00261169	4.00E-07	-
0.00253557	3.10E-06	-
0.0043472	3.80E-06	-
0.00611452	2.50E-06	-
0.00629425	4.10E-06	-
0.00262368	4.30E-06	-
0.0100244	4.30E-06	-
0.00578149	2.20E-06	-
0.00766936	3.60E-06	-
0.0022683	2.20E-07	-
0.00235748	1.20E-07	-
0.00554552	3.10E-06	-
0.00228303	4.10E-06	-
0.00276984	3.90E-06	-
0.00876048	1.90E-06	-
0.0105999	3.10E-06	-
0.00220019	1.10E-06	-
0.00646144	5.60E-07	-
0.00823522	3.40E-07	-
0.00350209	2.50E-06	-

0.00210992	2.70E-06	-
0.012279	3.00E-06	-
0.00774971	4.40E-06	-
0.00853021	2.00E-06	-
0.00230992	3.10E-06	-
0.00608032	1.90E-06	-
0.00211276	4.60E-06	-
0.00221752	4.80E-06	-
0.00222595	2.60E-06	-
0.00586509	4.60E-06	-
0.00212578	3.30E-06	-
0.00232023	9.20E-07	-
0.00406633	2.00E-06	-
0.00249815	1.80E-07	-
0.0108609	9.10E-07	-
0.0100662	1.80E-06	-
0.0027732	4.40E-06	-
0.00243151	2.10E-07	-
0.00226473	4.00E-06	-
0.00262039	1.20E-06	-
0.00259111	6.20E-17	-
0.00239094	3.20E-06	-
0.0026994	2.30E-07	-
0.00221931	3.10E-08	-
0.00214859	8.50E-09	-
0.00438434	7.60E-07	-
0.00300906	3.70E-06	-
0.00804635	4.00E-06	-
0.00235647	1.30E-06	-
0.0028777	3.40E-07	-
0.00230502	3.20E-06	-
0.00534513	3.90E-06	-
0.0028931	3.40E-06	-
0.00251245	3.60E-06	-
0.00392582	3.60E-06	-
0.00230502	1.90E-07	-
0.011897	4.80E-06	-
0.00226543	8.90E-07	-
0.00762753	4.20E-06	-
0.00392946	4.00E-06	-
0.00362056	8.60E-07	-
0.00238615	1.60E-06	-

0.00228323	1.20E-06	-
0.00218282	1.40E-08	-
0.00227496	3.70E-06	-
0.00294347	4.40E-06	-
0.019135	3.10E-09	-
0.00239801	2.40E-06	-
0.00424579	2.40E-06	-
0.00302016	9.90E-08	-
0.00276012	2.60E-06	-
0.00217008	1.50E-07	-
0.00241312	2.10E-08	-
0.00572778	4.20E-08	-
0.00238522	4.40E-06	-
0.00948467	8.10E-07	-
0.0065901	3.70E-07	-
0.00692319	4.60E-06	-
0.00386726	1.30E-06	-
0.00278984	2.80E-06	-
0.00215925	4.90E-06	-
0.00216741	2.00E-06	-
0.00304795	7.70E-07	-
0.00204522	2.00E-06	-
0.00208179	7.60E-07	-
0.00210846	3.00E-06	-
0.00395544	4.80E-06	-
0.00273431	4.10E-08	-
0.00283547	3.20E-08	-
0.00263194	2.00E-06	-
0.00834142	3.60E-06	-
0.00238685	4.00E-06	-
0.00213843	4.10E-06	-
0.00284617	3.30E-06	-
0.00206171	3.40E-06	-
0.00249759	2.00E-08	-
0.00243611	7.00E-16	-
0.00212158	4.70E-06	-
0.00257421	4.00E-07	-
0.00239699	1.80E-06	-
0.00307844	5.10E-07	-
0.00211393	9.60E-07	-
0.00794035	1.30E-06	-
0.0038227	9.80E-07	-

0.00223922	4.20E-06	-
0.00219189	4.30E-06	-
0.00304505	4.40E-06	-
0.0041442	1.10E-06	-
0.00330989	3.60E-06	-
0.00216058	6.80E-07	-
0.00218214	8.00E-07	-
0.00742849	2.40E-06	-
0.00235154	4.40E-06	-
0.00377883	2.80E-08	-
0.00783716	4.60E-06	-
0.00318004	4.20E-07	-
0.00241321	2.30E-06	-
0.00210349	4.00E-06	-
0.00428631	1.90E-06	-
0.00237872	1.80E-06	-
0.00285444	7.70E-10	-
0.00219366	7.00E-08	-
0.00618418	1.10E-06	-
0.00241176	2.40E-06	-
0.00275083	2.60E-06	-
0.00215778	4.30E-06	-
0.00438464	2.10E-06	-
0.00243539	7.90E-07	-
0.00207826	3.10E-07	-
0.00279712	1.50E-06	-
0.00206188	4.40E-06	-
0.00243998	4.50E-07	-
0.00243102	1.90E-06	-
0.0183509	2.50E-07	-
0.00228306	2.20E-06	-
0.00600813	1.80E-06	-
0.00648004	3.20E-07	-
0.0026301	5.10E-07	-
0.00488467	2.00E-06	-
0.00276029	9.70E-07	-
0.00337812	2.40E-06	-
0.00229517	9.60E-11	-
0.00334203	3.70E-06	-
0.00214122	4.20E-06	-
0.00227307	1.90E-07	-
0.00585868	3.60E-06	-

0.00627021	1.60E-06	-
0.00207188	8.20E-07	-
0.00626977	3.70E-09	-
0.0036924	1.70E-06	-
0.0051799	4.70E-06	-
0.00212721	1.40E-07	-
0.00340904	1.90E-06	-
0.00409857	1.90E-06	-
0.00447256	1.20E-06	-
0.00836887	3.50E-06	-
0.008321	4.40E-07	-
0.00738282	1.60E-07	-
0.00261169	4.00E-07	-
0.00253557	3.10E-06	-
0.0043472	3.80E-06	-
0.00611452	2.50E-06	-
0.00629425	4.10E-06	-
0.00262368	4.30E-06	-
0.0100244	4.30E-06	-
0.00578149	2.20E-06	-
0.00766936	3.60E-06	-
0.0022683	2.20E-07	-
0.00235748	1.20E-07	-
0.00554552	3.10E-06	-
0.00228303	4.10E-06	-
0.00276984	3.90E-06	-
0.00876048	1.90E-06	-
0.0105999	3.10E-06	-
0.00220019	1.10E-06	-
0.00646144	5.60E-07	-
0.00823522	3.40E-07	-
0.00350209	2.50E-06	-
0.00406633	2.00E-06	-
0.00249815	1.80E-07	-
0.0108609	9.10E-07	-
0.0100662	1.80E-06	-
0.0027732	4.40E-06	-
0.00243151	2.10E-07	-
0.00226473	4.00E-06	-
0.00262039	1.20E-06	-
0.00259111	6.20E-17	-
0.00239094	3.20E-06	-

0.0026994	2.30E-07	-
0.00221931	3.10E-08	-
0.00214859	8.50E-09	-
0.00438434	7.60E-07	-
0.00300906	3.70E-06	-
0.00804635	4.00E-06	-
0.00235647	1.30E-06	-
0.0028777	3.40E-07	-
0.00230502	3.20E-06	-
0.00534513	3.90E-06	-
0.0028931	3.40E-06	-
0.00251245	3.60E-06	-
0.00392582	3.60E-06	-
0.00230502	1.90E-07	-
0.011897	4.80E-06	-
0.00226543	8.90E-07	-
0.00762753	4.20E-06	-
0.00392946	4.00E-06	-
0.00362056	8.60E-07	-
0.00238615	1.60E-06	-
0.00228323	1.20E-06	-
0.00218282	1.40E-08	-
0.00227496	3.70E-06	-
0.00294347	4.40E-06	-
0.019135	3.10E-09	-
0.00239801	2.40E-06	-
0.00424579	2.40E-06	-
0.00302016	9.90E-08	-
0.00276012	2.60E-06	-
0.00217008	1.50E-07	-
0.00241312	2.10E-08	-
0.00572778	4.20E-08	-
0.00238522	4.40E-06	-
0.0065901	3.70E-07	-
0.00692319	4.60E-06	-
0.00386726	1.30E-06	-
0.00278984	2.80E-06	-
0.00215925	4.90E-06	-
0.00216741	2.00E-06	-
0.00210992	2.70E-06	-
0.012279	3.00E-06	-
0.00774971	4.40E-06	-

0.00853021	2.00E-06	rs9657892
0.00230992	3.10E-06	-
0.00608032	1.90E-06	-
0.00211276	4.60E-06	-
0.00221752	4.80E-06	-
0.00222595	2.60E-06	-
0.00586509	4.60E-06	-
0.00212578	3.30E-06	-
0.00232023	9.20E-07	-

Exposure	Outcome
Particulate matter air pollution (pm2.5)	Coronary artery disease
Particulate matter air pollution (pm2.5)	Coronary artery disease
Particulate matter air pollution (pm2.5)	Coronary artery disease
Particulate matter air pollution (pm2.5)	Coronary artery disease
Particulate matter air pollution (pm2.5)	Coronary artery disease
Particulate matter air pollution (pm2.5)	Atrial fibrillation
Particulate matter air pollution (pm2.5)	Atrial fibrillation
Particulate matter air pollution (pm2.5)	Atrial fibrillation
Particulate matter air pollution (pm2.5)	Atrial fibrillation
Particulate matter air pollution (pm2.5)	Atrial fibrillation
Particulate matter air pollution (pm2.5)	Heart failure
Particulate matter air pollution (pm2.5)	Heart failure
Particulate matter air pollution (pm2.5)	Heart failure
Particulate matter air pollution (pm2.5)	Heart failure
Particulate matter air pollution (pm2.5)	Heart failure
Particulate matter air pollution (pm2.5)	Cardiac arrhytmias
Particulate matter air pollution (pm2.5)	Cardiac arrhytmias
Particulate matter air pollution (pm2.5)	Cardiac arrhytmias
Particulate matter air pollution (pm2.5)	Cardiac arrhytmias
Particulate matter air pollution (pm2.5)	Cardiac arrhytmias
Particulate matter air pollution (pm2.5)	Cardiomyopathy
Particulate matter air pollution (pm2.5)	Cardiomyopathy
Particulate matter air pollution (pm2.5)	Cardiomyopathy
Particulate matter air pollution (pm2.5)	Cardiomyopathy
Particulate matter air pollution (pm2.5)	Cardiomyopathy
Particulate matter air pollution (pm2.5)	Hypertrophic cardiomyopathy
Particulate matter air pollution (pm2.5)	Hypertrophic cardiomyopathy
Particulate matter air pollution (pm2.5)	Hypertrophic cardiomyopathy
Particulate matter air pollution (pm2.5)	Hypertrophic cardiomyopathy
Particulate matter air pollution (pm2.5)	Hypertrophic cardiomyopathy
Particulate matter air pollution (pm2.5)	Hypertension
Particulate matter air pollution (pm2.5)	Hypertension
Particulate matter air pollution (pm2.5)	Hypertension
Particulate matter air pollution (pm2.5)	Hypertension
Particulate matter air pollution (pm2.5)	Hypertension
Particulate matter air pollution (pm2.5)	Ischemic heart diseases
Particulate matter air pollution (pm2.5)	Ischemic heart diseases
Particulate matter air pollution (pm2.5)	Ischemic heart diseases
Particulate matter air pollution (pm2.5)	Ischemic heart diseases
Particulate matter air pollution (pm2.5)	Ischemic heart diseases
Particulate matter air pollution (pm2.5)	Non-ischemic cardiomyopathy

Particulate matter air pollution (pm2.5)	Non-ischemic cardiomyopathy
Particulate matter air pollution (pm2.5)	Non-ischemic cardiomyopathy
Particulate matter air pollution (pm2.5)	Non-ischemic cardiomyopathy
Particulate matter air pollution (pm2.5)	Non-ischemic cardiomyopathy
Particulate matter air pollution (pm2.5)	Pulmonary heart disease
Particulate matter air pollution (pm2.5)	Pulmonary heart disease
Particulate matter air pollution (pm2.5)	Pulmonary heart disease
Particulate matter air pollution (pm2.5)	Pulmonary heart disease
Particulate matter air pollution (pm2.5)	Pulmonary heart disease
Particulate matter air pollution (pm2.5)	Valvular heart disease
Particulate matter air pollution (pm2.5)	Valvular heart disease
Particulate matter air pollution (pm2.5)	Valvular heart disease
Particulate matter air pollution (pm2.5)	Valvular heart disease
Particulate matter air pollution (pm2.5)	Valvular heart disease
Particulate matter air pollution (pm2.5)	Coronary heart disease
Particulate matter air pollution (pm2.5)	Coronary heart disease
Particulate matter air pollution (pm2.5)	Coronary heart disease
Particulate matter air pollution (pm2.5)	Coronary heart disease
Particulate matter air pollution (pm2.5)	Coronary heart disease
Particulate matter air pollution (pm2.5)	Myocardial infarction
Particulate matter air pollution (pm2.5)	Myocardial infarction
Particulate matter air pollution (pm2.5)	Myocardial infarction
Particulate matter air pollution (pm2.5)	Myocardial infarction
Particulate matter air pollution (pm2.5)	Myocardial infarction
Particulate matter air pollution (pm10)	Coronary artery disease
Particulate matter air pollution (pm10)	Coronary artery disease
Particulate matter air pollution (pm10)	Coronary artery disease
Particulate matter air pollution (pm10)	Coronary artery disease
Particulate matter air pollution (pm10)	Coronary artery disease
Particulate matter air pollution (pm10)	Atrial fibrillation
Particulate matter air pollution (pm10)	Atrial fibrillation
Particulate matter air pollution (pm10)	Atrial fibrillation
Particulate matter air pollution (pm10)	Atrial fibrillation
Particulate matter air pollution (pm10)	Atrial fibrillation
Particulate matter air pollution (pm10)	Heart failure
Particulate matter air pollution (pm10)	Heart failure
Particulate matter air pollution (pm10)	Heart failure
Particulate matter air pollution (pm10)	Heart failure
Particulate matter air pollution (pm10)	Heart failure
Particulate matter air pollution (pm10)	Cardiac arrhytmias
Particulate matter air pollution (pm10)	Cardiac arrhytmias
Particulate matter air pollution (pm10)	Cardiac arrhytmias

Particulate matter air pollution (pm10)	Cardiac arrhytmias
Particulate matter air pollution (pm10)	Cardiac arrhytmias
Particulate matter air pollution (pm10)	Cardiomyopathy
Particulate matter air pollution (pm10)	Cardiomyopathy
Particulate matter air pollution (pm10)	Cardiomyopathy
Particulate matter air pollution (pm10)	Cardiomyopathy
Particulate matter air pollution (pm10)	Cardiomyopathy
Particulate matter air pollution (pm10)	Hypertrophic cardiomyopathy
Particulate matter air pollution (pm10)	Hypertrophic cardiomyopathy
Particulate matter air pollution (pm10)	Hypertrophic cardiomyopathy
Particulate matter air pollution (pm10)	Hypertrophic cardiomyopathy
Particulate matter air pollution (pm10)	Hypertrophic cardiomyopathy
Particulate matter air pollution (pm10)	Hypertension
Particulate matter air pollution (pm10)	Hypertension
Particulate matter air pollution (pm10)	Hypertension
Particulate matter air pollution (pm10)	Hypertension
Particulate matter air pollution (pm10)	Hypertension
Particulate matter air pollution (pm10)	Ischemic heart diseases
Particulate matter air pollution (pm10)	Ischemic heart diseases
Particulate matter air pollution (pm10)	Ischemic heart diseases
Particulate matter air pollution (pm10)	Ischemic heart diseases
Particulate matter air pollution (pm10)	Ischemic heart diseases
Particulate matter air pollution (pm10)	Non-ischemic cardiomyopathy
Particulate matter air pollution (pm10)	Non-ischemic cardiomyopathy
Particulate matter air pollution (pm10)	Non-ischemic cardiomyopathy
Particulate matter air pollution (pm10)	Non-ischemic cardiomyopathy
Particulate matter air pollution (pm10)	Non-ischemic cardiomyopathy
Particulate matter air pollution (pm10)	Pulmonary heart disease
Particulate matter air pollution (pm10)	Pulmonary heart disease
Particulate matter air pollution (pm10)	Pulmonary heart disease
Particulate matter air pollution (pm10)	Pulmonary heart disease
Particulate matter air pollution (pm10)	Pulmonary heart disease
Particulate matter air pollution (pm10)	Valvular heart disease
Particulate matter air pollution (pm10)	Valvular heart disease
Particulate matter air pollution (pm10)	Valvular heart disease
Particulate matter air pollution (pm10)	Valvular heart disease
Particulate matter air pollution (pm10)	Valvular heart disease
Particulate matter air pollution (pm10)	Coronary heart disease
Particulate matter air pollution (pm10)	Coronary heart disease
Particulate matter air pollution (pm10)	Coronary heart disease
Particulate matter air pollution (pm10)	Coronary heart disease
Particulate matter air pollution (pm10)	Coronary heart disease

Particulate matter air pollution (pm10)	Myocardial infarction
Particulate matter air pollution (pm10)	Myocardial infarction
Particulate matter air pollution (pm10)	Myocardial infarction
Particulate matter air pollution (pm10)	Myocardial infarction
Particulate matter air pollution (pm10)	Myocardial infarction
Average 24-hour sound level of noise pollution	Coronary artery disease
Average 24-hour sound level of noise pollution	Coronary artery disease
Average 24-hour sound level of noise pollution	Coronary artery disease
Average 24-hour sound level of noise pollution	Coronary artery disease
Average 24-hour sound level of noise pollution	Coronary artery disease
Average 24-hour sound level of noise pollution	Atrial fibrillation
Average 24-hour sound level of noise pollution	Atrial fibrillation
Average 24-hour sound level of noise pollution	Atrial fibrillation
Average 24-hour sound level of noise pollution	Atrial fibrillation
Average 24-hour sound level of noise pollution	Atrial fibrillation
Average 24-hour sound level of noise pollution	Heart failure
Average 24-hour sound level of noise pollution	Heart failure
Average 24-hour sound level of noise pollution	Heart failure
Average 24-hour sound level of noise pollution	Heart failure
Average 24-hour sound level of noise pollution	Heart failure
Average 24-hour sound level of noise pollution	Cardiac arrhytmias
Average 24-hour sound level of noise pollution	Cardiac arrhytmias
Average 24-hour sound level of noise pollution	Cardiac arrhytmias
Average 24-hour sound level of noise pollution	Cardiac arrhytmias
Average 24-hour sound level of noise pollution	Cardiac arrhytmias
Average 24-hour sound level of noise pollution	Cardiomyopathy
Average 24-hour sound level of noise pollution	Cardiomyopathy
Average 24-hour sound level of noise pollution	Cardiomyopathy
Average 24-hour sound level of noise pollution	Cardiomyopathy
Average 24-hour sound level of noise pollution	Cardiomyopathy
Average 24-hour sound level of noise pollution	Hypertrophic cardiomyopathy
Average 24-hour sound level of noise pollution	Hypertrophic cardiomyopathy
Average 24-hour sound level of noise pollution	Hypertrophic cardiomyopathy
Average 24-hour sound level of noise pollution	Hypertrophic cardiomyopathy
Average 24-hour sound level of noise pollution	Hypertrophic cardiomyopathy
Average 24-hour sound level of noise pollution	Hypertension
Average 24-hour sound level of noise pollution	Hypertension
Average 24-hour sound level of noise pollution	Hypertension
Average 24-hour sound level of noise pollution	Hypertension
Average 24-hour sound level of noise pollution	Hypertension
Average 24-hour sound level of noise pollution	Ischemic heart diseases
Average 24-hour sound level of noise pollution	Ischemic heart diseases

Average 24-hour sound level of noise pollution	Ischemic heart diseases
Average 24-hour sound level of noise pollution	Ischemic heart diseases
Average 24-hour sound level of noise pollution	Ischemic heart diseases
Average 24-hour sound level of noise pollution	Non-ischemic cardiomyopathy
Average 24-hour sound level of noise pollution	Non-ischemic cardiomyopathy
Average 24-hour sound level of noise pollution	Non-ischemic cardiomyopathy
Average 24-hour sound level of noise pollution	Non-ischemic cardiomyopathy
Average 24-hour sound level of noise pollution	Non-ischemic cardiomyopathy
Average 24-hour sound level of noise pollution	Pulmonary heart disease
Average 24-hour sound level of noise pollution	Pulmonary heart disease
Average 24-hour sound level of noise pollution	Pulmonary heart disease
Average 24-hour sound level of noise pollution	Pulmonary heart disease
Average 24-hour sound level of noise pollution	Pulmonary heart disease
Average 24-hour sound level of noise pollution	Valvular heart disease
Average 24-hour sound level of noise pollution	Valvular heart disease
Average 24-hour sound level of noise pollution	Valvular heart disease
Average 24-hour sound level of noise pollution	Valvular heart disease
Average 24-hour sound level of noise pollution	Valvular heart disease
Average 24-hour sound level of noise pollution	Coronary heart disease
Average 24-hour sound level of noise pollution	Coronary heart disease
Average 24-hour sound level of noise pollution	Coronary heart disease
Average 24-hour sound level of noise pollution	Coronary heart disease
Average 24-hour sound level of noise pollution	Coronary heart disease
Average 24-hour sound level of noise pollution	Myocardial infarction
Average 24-hour sound level of noise pollution	Myocardial infarction
Average 24-hour sound level of noise pollution	Myocardial infarction
Average 24-hour sound level of noise pollution	Myocardial infarction
Average 24-hour sound level of noise pollution	Myocardial infarction
Nitrogen dioxide air pollution	Coronary artery disease
Nitrogen dioxide air pollution	Coronary artery disease
Nitrogen dioxide air pollution	Coronary artery disease
Nitrogen dioxide air pollution	Coronary artery disease
Nitrogen dioxide air pollution	Coronary artery disease
Nitrogen dioxide air pollution	Atrial fibrillation
Nitrogen dioxide air pollution	Atrial fibrillation
Nitrogen dioxide air pollution	Atrial fibrillation
Nitrogen dioxide air pollution	Atrial fibrillation
Nitrogen dioxide air pollution	Atrial fibrillation
Nitrogen dioxide air pollution	Heart failure
Nitrogen dioxide air pollution	Heart failure
Nitrogen dioxide air pollution	Heart failure
Nitrogen dioxide air pollution	Heart failure

Nitrogen dioxide air pollution	Heart failure
Nitrogen dioxide air pollution	Cardiac arrhytmias
Nitrogen dioxide air pollution	Cardiac arrhytmias
Nitrogen dioxide air pollution	Cardiac arrhytmias
Nitrogen dioxide air pollution	Cardiac arrhytmias
Nitrogen dioxide air pollution	Cardiac arrhytmias
Nitrogen dioxide air pollution	Cardiomyopathy
Nitrogen dioxide air pollution	Cardiomyopathy
Nitrogen dioxide air pollution	Cardiomyopathy
Nitrogen dioxide air pollution	Cardiomyopathy
Nitrogen dioxide air pollution	Cardiomyopathy
Nitrogen dioxide air pollution	Hypertrophic cardiomyopathy
Nitrogen dioxide air pollution	Hypertrophic cardiomyopathy
Nitrogen dioxide air pollution	Hypertrophic cardiomyopathy
Nitrogen dioxide air pollution	Hypertrophic cardiomyopathy
Nitrogen dioxide air pollution	Hypertrophic cardiomyopathy
Nitrogen dioxide air pollution	Hypertension
Nitrogen dioxide air pollution	Hypertension
Nitrogen dioxide air pollution	Hypertension
Nitrogen dioxide air pollution	Hypertension
Nitrogen dioxide air pollution	Hypertension
Nitrogen dioxide air pollution	Ischemic heart diseases
Nitrogen dioxide air pollution	Ischemic heart diseases
Nitrogen dioxide air pollution	Ischemic heart diseases
Nitrogen dioxide air pollution	Ischemic heart diseases
Nitrogen dioxide air pollution	Ischemic heart diseases
Nitrogen dioxide air pollution	Non-ischemic cardiomyopathy
Nitrogen dioxide air pollution	Non-ischemic cardiomyopathy
Nitrogen dioxide air pollution	Non-ischemic cardiomyopathy
Nitrogen dioxide air pollution	Non-ischemic cardiomyopathy
Nitrogen dioxide air pollution	Non-ischemic cardiomyopathy
Nitrogen dioxide air pollution	Pulmonary heart disease
Nitrogen dioxide air pollution	Pulmonary heart disease
Nitrogen dioxide air pollution	Pulmonary heart disease
Nitrogen dioxide air pollution	Pulmonary heart disease
Nitrogen dioxide air pollution	Pulmonary heart disease
Nitrogen dioxide air pollution	Valvular heart disease
Nitrogen dioxide air pollution	Valvular heart disease
Nitrogen dioxide air pollution	Valvular heart disease
Nitrogen dioxide air pollution	Valvular heart disease
Nitrogen dioxide air pollution	Valvular heart disease
Nitrogen dioxide air pollution	Coronary heart disease

Nitrogen dioxide air pollution	Coronary heart disease
Nitrogen dioxide air pollution	Coronary heart disease
Nitrogen dioxide air pollution	Coronary heart disease
Nitrogen dioxide air pollution	Coronary heart disease
Nitrogen dioxide air pollution	Myocardial infarction
Nitrogen dioxide air pollution	Myocardial infarction
Nitrogen dioxide air pollution	Myocardial infarction
Nitrogen dioxide air pollution	Myocardial infarction
Nitrogen dioxide air pollution	Myocardial infarction

Method	No. of snp	Beta	se
Maximum likelihood	51	0.8801	0.1239
MR Egger	51	0.3295	0.8397
Weighted median	51	0.271	0.1373
IVW	51	0.5626	0.3225
Weighted mode	51	0.3411	0.2651
Maximum likelihood	52	0.1091	0.08918
MR Egger	52	0.1515	0.2431
Weighted median	52	0.05861	0.1392
IVW	52	0.1063	0.09872
Weighted mode	52	0.1899	0.2209
Maximum likelihood	47	0.3476	0.1121
MR Egger	47	0.1799	0.4472
Weighted median	47	0.1564	0.1653
IVW	47	0.3378	0.1633
Weighted mode	47	0.1778	0.2468
Maximum likelihood	49	0.06782	0.12
MR Egger	49	0.01837	0.2227
Weighted median	49	-0.02683	0.2008
IVW	49	0.06528	0.1348
Weighted mode	49	-0.008061	0.1761
Maximum likelihood	49	0.4964	0.3132
MR Egger	49	0.1779	0.4922
Weighted median	49	-0.02195	0.4659
IVW	49	0.4615	0.3002
Weighted mode	49	-0.1004	0.478
Maximum likelihood	49	0.265	0.6655
MR Egger	49	0.2645	1.063
Weighted median	49	-0.2615	1.014
IVW	49	0.248	0.6543
Weighted mode	49	-0.3795	1.067
Maximum likelihood	49	0.1917	0.1026
MR Egger	49	0.1464	0.1875
Weighted median	49	0.137	0.1602
IVW	49	0.1866	0.1132
Weighted mode	49	0.1563	0.1511
Maximum likelihood	49	0.3885	0.1496
MR Egger	49	-0.1957	0.4923
Weighted median	49	-0.07323	0.2011
IVW	49	0.3438	0.3036
Weighted mode	49	0.001135	0.171
Maximum likelihood	49	-0.1243	0.1636

MR Egger	49	-0.04964	0.2883
Weighted median	49	-0.1686	0.2731
IVW	49	-0.1203	0.174
Weighted mode	49	-0.2004	0.2422
Maximum likelihood	49	0.1251	0.2433
MR Egger	49	0.175	0.3937
Weighted median	49	0.0827	0.3986
IVW	49	0.1216	0.2398
Weighted mode	49	0.003006	0.3767
Maximum likelihood	49	0.1916	0.09826
MR Egger	49	0.1013	0.1814
Weighted median	49	0.144	0.1641
IVW	49	0.1867	0.1098
Weighted mode	49	0.1098	0.1404
Maximum likelihood	50	0.2346	0.1129
MR Egger	50	0.02101	0.3696
Weighted median	50	0.1043	0.1709
IVW	50	0.2281	0.2239
Weighted mode	50	0.1365	0.1621
Maximum likelihood	49	0.4263	0.1504
MR Egger	49	-0.06788	0.5281
Weighted median	49	0.06943	0.1925
IVW	49	0.364	0.3168
Weighted mode	49	0.1207	0.1725
Maximum likelihood	26	0.2391	0.1355
MR Egger	26	0.3886	0.3264
Weighted median	26	0.1404	0.1959
IVW	26	0.2266	0.1447
Weighted mode	26	0.01432	0.3834
Maximum likelihood	27	0.3651	0.1379
MR Egger	27	0.4267	0.364
Weighted median	27	0.2532	0.2016
IVW	27	0.3444	0.163
Weighted mode	27	0.2052	0.4113
Maximum likelihood	25	0.329	0.1645
MR Egger	25	0.3112	0.451
Weighted median	25	0.3981	0.2221
IVW	25	0.3154	0.1919
Weighted mode	25	0.4007	0.4056
Maximum likelihood	27	0.1551	0.2117
MR Egger	27	0.4777	0.432
Weighted median	27	-0.2538	0.2831

IVW	27	0.1499	0.2079
Weighted mode	27	-0.2535	0.5417
Maximum likelihood	27	0.7362	0.5364
MR Egger	27	0.9863	1.168
Weighted median	27	0.01582	0.7394
IVW	27	0.7054	0.5517
Weighted mode	27	0.1608	1.087
Maximum likelihood	27	0.7933	1.204
MR Egger	27	1.381	2.617
Weighted median	27	-1.553	1.699
IVW	27	0.7634	1.239
Weighted mode	27	-2.614	3.336
Maximum likelihood	27	0.3885	0.1818
MR Egger	27	0.2277	0.3681
Weighted median	27	0.3704	0.2525
IVW	27	0.3712	0.177
Weighted mode	27	0.5037	0.4647
Maximum likelihood	27	0.1956	0.2216
MR Egger	27	0.4553	0.5777
Weighted median	27	0.3846	0.3162
IVW	27	0.1856	0.2741
Weighted mode	27	0.9853	0.5492
Maximum likelihood	27	0.4866	0.2962
MR Egger	27	-0.2272	0.7335
Weighted median	27	0.5477	0.4191
IVW	27	0.4634	0.3534
Weighted mode	27	0.521	0.7513
Maximum likelihood	27	-0.1986	0.4319
MR Egger	27	-1.521	0.8794
Weighted median	27	0.09142	0.5955
IVW	27	-0.1945	0.4232
Weighted mode	27	0.3173	1.084
Maximum likelihood	27	0.02144	0.1721
MR Egger	27	-0.02752	0.3519
Weighted median	27	-0.06689	0.2383
IVW	27	0.02073	0.1692
Weighted mode	27	-0.09348	0.3815
Maximum likelihood	26	0.005038	0.2081
MR Egger	26	-0.1865	0.4511
Weighted median	26	0.1447	0.2798
IVW	26	0.004988	0.2053
Weighted mode	26	0.1119	0.4627

Maximum likelihood	26	0.1704	0.2296
MR Egger	26	0.1004	0.4962
Weighted median	26	0.2294	0.2948
IVW	26	0.1648	0.2268
Weighted mode	26	0.2772	0.505
Maximum likelihood	12	-0.06552	0.2149
MR Egger	12	-0.5308	0.6134
Weighted median	12	-0.1568	0.3092
IVW	12	-0.06588	0.2699
Weighted mode	12	-0.6399	0.5061
Maximum likelihood	12	0.07938	0.2133
MR Egger	12	-0.06348	0.5028
Weighted median	12	0.2709	0.2786
IVW	12	0.0765	0.2089
Weighted mode	12	0.4087	0.5094
Maximum likelihood	11	-0.2084	0.2552
MR Egger	11	-0.2877	0.6545
Weighted median	11	-0.2659	0.3512
IVW	11	-0.2032	0.2504
Weighted mode	11	-0.4422	0.5355
Maximum likelihood	12	0.1008	0.2911
MR Egger	12	-0.9208	0.5319
Weighted median	12	-0.4878	0.3968
IVW	12	0.09615	0.2866
Weighted mode	12	-0.4835	0.4717
Maximum likelihood	12	-0.07199	0.7326
MR Egger	12	-1.681	1.594
Weighted median	12	0.175	1.083
IVW	12	-0.07056	0.8625
Weighted mode	12	0.004088	1.281
Maximum likelihood	12	-3.417	1.616
MR Egger	12	-8.186	3.003
Weighted median	12	-2.832	2.28
IVW	12	-3.357	1.593
Weighted mode	12	-3.956	3.221
Maximum likelihood	12	0.1741	0.2503
MR Egger	12	-0.4905	0.6012
Weighted median	12	-0.1619	0.3552
IVW	12	0.1608	0.3299
Weighted mode	12	-0.2893	0.3957
Maximum likelihood	12	-0.4068	0.3157
MR Egger	12	0.4865	0.8023

Weighted median	12	0.4984	0.442
IVW	12	-0.3783	0.4408
Weighted mode	12	0.5172	0.4939
Maximum likelihood	12	0.5656	0.4043
MR Egger	12	-0.02899	0.7552
Weighted median	12	0.316	0.5423
IVW	12	0.5564	0.3974
Weighted mode	12	0.1393	0.653
Maximum likelihood	12	-0.1986	0.5898
MR Egger	12	-0.8708	1.115
Weighted median	12	-0.6671	0.7995
IVW	12	-0.2047	0.5794
Weighted mode	12	-0.6164	0.983
Maximum likelihood	12	0.251	0.2369
MR Egger	12	-0.4038	0.4346
Weighted median	12	0.02494	0.3233
IVW	12	0.2411	0.2421
Weighted mode	12	0.03568	0.3761
Maximum likelihood	12	-0.5274	0.3102
MR Egger	12	-0.2494	0.8291
Weighted median	12	-0.4919	0.4367
IVW	12	-0.5292	0.3168
Weighted mode	12	-0.2714	0.6643
Maximum likelihood	12	-0.2303	0.3391
MR Egger	12	-0.7077	0.8239
Weighted median	12	-0.232	0.4502
IVW	12	-0.2319	0.3332
Weighted mode	12	0.5282	0.7597
Maximum likelihood	71	0.3329	0.09953
MR Egger	71	-0.6471	0.6804
Weighted median	71	0.2523	0.1283
IVW	71	0.2657	0.2405
Weighted mode	71	0.3871	0.2827
Maximum likelihood	73	0.07591	0.07954
MR Egger	73	-0.09986	0.2316
Weighted median	73	-0.004788	0.1177
IVW	73	0.07294	0.08684
Weighted mode	73	-0.04109	0.2482
Maximum likelihood	72	0.1175	0.09585
MR Egger	72	-0.453	0.36
Weighted median	72	0.1232	0.1376
IVW	72	0.1134	0.134

Weighted mode	72	0.1764	0.2973
Maximum likelihood	71	-0.00318	0.1075
MR Egger	71	-0.1835	0.2068
Weighted median	71	-0.05932	0.1921
IVW	71	-0.003137	0.1149
Weighted mode	71	-0.1005	0.1834
Maximum likelihood	71	0.5104	0.2767
MR Egger	71	-0.08387	0.4882
Weighted median	71	0.05657	0.4299
IVW	71	0.4842	0.2739
Weighted mode	71	0.01856	0.5045
Maximum likelihood	71	-0.08653	0.6006
MR Egger	71	-0.8086	1.091
Weighted median	71	-0.3262	1.057
IVW	71	-0.08654	0.6074
Weighted mode	71	0.1036	1.002
Maximum likelihood	71	-0.1442	0.09485
MR Egger	71	-0.1927	0.2284
Weighted median	71	0.00791	0.1357
IVW	71	-0.1372	0.1258
Weighted mode	71	0.06493	0.1577
Maximum likelihood	71	-0.01006	0.116
MR Egger	71	-0.3489	0.3955
Weighted median	71	-0.1176	0.1817
IVW	71	-0.009426	0.2196
Weighted mode	71	-0.1455	0.1989
Maximum likelihood	71	-0.1481	0.1472
MR Egger	71	-0.2868	0.2835
Weighted median	71	-0.2479	0.2481
IVW	71	-0.1444	0.1564
Weighted mode	71	-0.2616	0.2737
Maximum likelihood	71	-0.1466	0.2201
MR Egger	71	0.1555	0.3987
Weighted median	71	-0.0213	0.3563
IVW	71	-0.1432	0.2202
Weighted mode	71	-0.1342	0.3726
Maximum likelihood	71	0.05953	0.08754
MR Egger	71	0.02881	0.1676
Weighted median	71	0.06639	0.1415
IVW	71	0.05807	0.09216
Weighted mode	71	0.07565	0.1636
Maximum likelihood	70	0.05587	0.1071

MR Egger	70	-0.2323	0.3715
Weighted median	70	0.1229	0.1935
IVW	70	0.05326	0.1871
Weighted mode	70	0.1431	0.2065
Maximum likelihood	70	0.2239	0.1276
MR Egger	70	-0.4954	0.4875
Weighted median	70	0.05412	0.2228
IVW	70	0.2037	0.2469
Weighted mode	70	0.02633	0.2052

p-value
1.21E-12
0.6965
0.0484
0.08107
0.2041
0.221
0.536
0.6737
0.2815
0.394
0.001931
0.6893
0.344
0.0386
0.4749
0.572
0.9346
0.8937
0.6281
0.9637
0.113
0.7194
0.9624
0.1242
0.8346
0.6905
0.8046
0.7964
0.7047
0.7237
0.06161
0.4387
0.3926
0.0993
0.3062
0.00942
0.6927
0.7157
0.2575
0.9947
0.4475

0.8641
0.537
0.4894
0.412
0.607
0.6587
0.8357
0.6121
0.9937
0.05118
0.5793
0.3802
0.08925
0.438
0.03778
0.9549
0.5419
0.3084
0.4039
0.004607
0.8983
0.7183
0.2506
0.4875
0.07767
0.2455
0.4735
0.1173
0.9705
0.008081
0.2521
0.209
0.03461
0.6221
0.04558
0.497
0.07311
0.1002
0.3331
0.4639
0.2794
0.3701

0.4707
0.6437
0.1699
0.4065
0.9829
0.201
0.8836
0.51
0.6025
0.3606
0.5377
0.4404
0.03263
0.5418
0.1423
0.03598
0.2884
0.3774
0.438
0.2239
0.4983
0.08446
0.1004
0.7593
0.1913
0.1897
0.4942
0.6456
0.09607
0.878
0.6457
0.7721
0.9009
0.9383
0.779
0.9025
0.8084
0.9807
0.6829
0.6051
0.9806
0.8109

0.4581
0.8414
0.4366
0.4675
0.5879
0.7605
0.4071
0.6121
0.8072
0.2322
0.7098
0.902
0.3308
0.7142
0.4393
0.414
0.6705
0.4489
0.417
0.4282
0.7293
0.1141
0.2189
0.7372
0.3274
0.9217
0.3163
0.8716
0.9348
0.9975
0.03446
0.02135
0.2141
0.03508
0.2451
0.4868
0.4336
0.6485
0.626
0.48
0.1975
0.5578

0.2594
0.3908
0.3175
0.1618
0.9701
0.5601
0.1615
0.8349
0.7363
0.4529
0.404
0.7238
0.5434
0.2892
0.3748
0.9385
0.3193
0.9261
0.08911
0.7697
0.26
0.09481
0.6907
0.497
0.4105
0.6063
0.4864
0.5013
0.0008242
0.3449
0.04929
0.2692
0.1752
0.3399
0.6676
0.9676
0.401
0.869
0.2202
0.2125
0.3706
0.3973

0.5547
0.9764
0.378
0.7575
0.9782
0.5853
0.06507
0.8641
0.8953
0.07705
0.9708
0.8854
0.4611
0.7575
0.8867
0.9179
0.1284
0.4018
0.9535
0.2755
0.6818
0.9309
0.3807
0.5173
0.9658
0.4668
0.3141
0.3153
0.3177
0.3557
0.3424
0.5056
0.6977
0.9523
0.5156
0.7198
0.4965
0.864
0.6391
0.5286
0.6453
0.6018

0.5339
0.5254
0.7759
0.4905
0.07934
0.3131
0.808
0.4093
0.8983

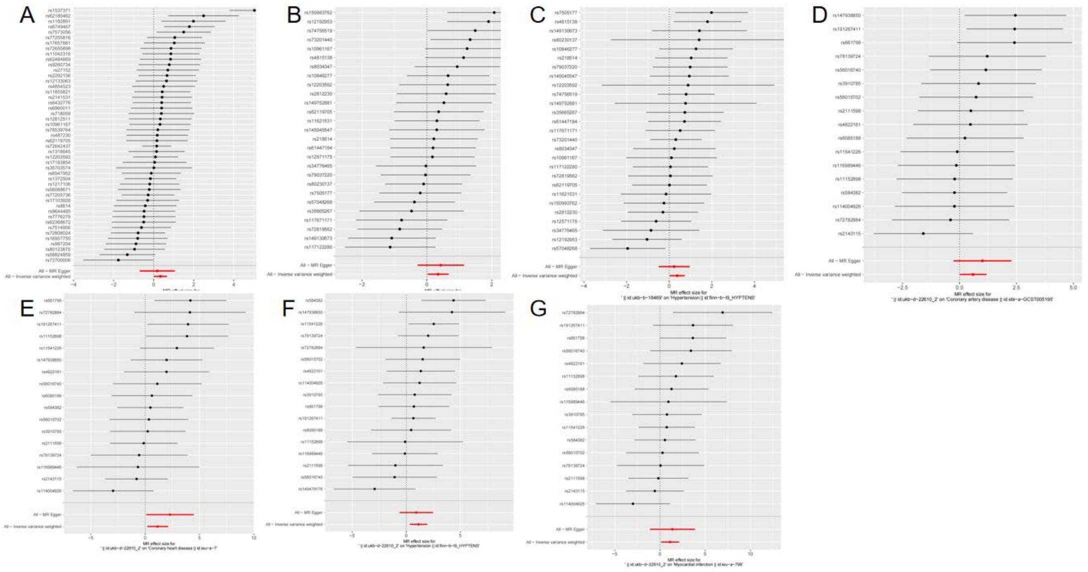

Supplementary Figure 1 sensitivity analysis: (A) Heart failure; (B) Hypertension; (C) Atrial fibrillation; (D) Hypertension; (E) Coronary artery disease; (F) Coronary heart disease; (G)Myocardial infarction

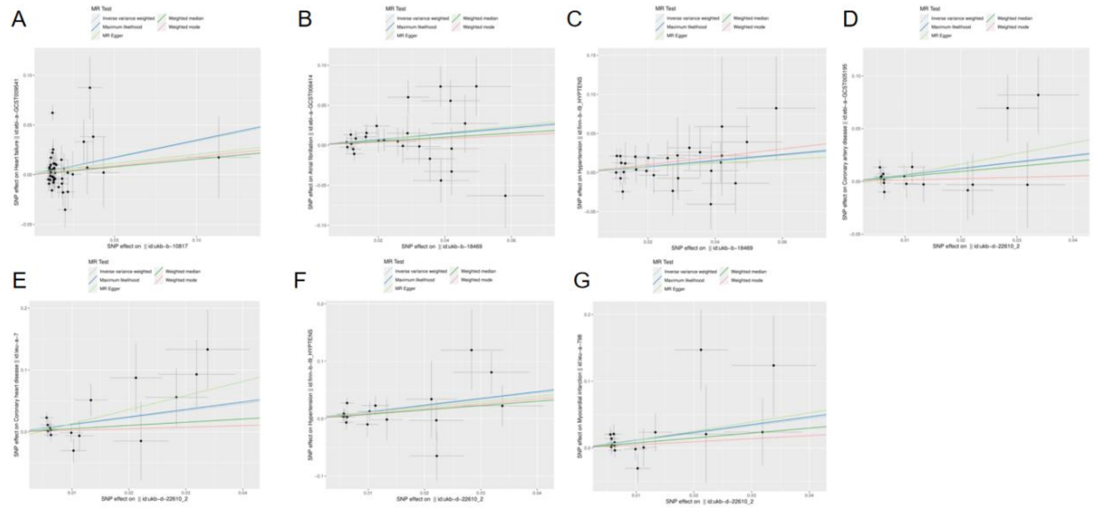

Supplementary Figure 2 Funnel plot: (A) Heart failure; (B) Hypertension; (C) Atrial fibrillation; (D) Hypertension; (E) Coronary artery disease; (F) Coronary heart disease; (G)Myocardial infarction
